# Hypervalent iodine-mediated cyclization of bishomoallylamides to prolinols

**DOI:** 10.3762/bjoc.20.209

**Published:** 2024-09-30

**Authors:** Smaher E Butt, Konrad Kepski, Jean-Marc Sotiropoulos, Wesley J Moran

**Affiliations:** 1 Department of Physical and Life Sciences, University of Huddersfield, Queensgate, Huddersfield HD1 3DH, United Kingdom; 2 Université de Pau et des Pays de l’Adour, IPREM (CNRS-UMR 5254), Technopole Hélioparc, 2 Avenue du Président Pierre Angot, 64053 Pau Cedex 09, France; 3 School of Pharmacy and Bioengineering, Keele University, Keele, Staffordshire ST5 5JX, United Kingdom

**Keywords:** cyclization, DFT, hypervalent iodine, mechanism, proline

## Abstract

A change in mechanism was observed in the hypervalent iodine-mediated cyclization of *N*-alkenylamides when the carbon chain between the alkene and the amide increased from two to three atoms. In the latter case, cyclization at the amide nitrogen to form the pyrrolidine ring was favored over cyclization at the amide oxygen. A DFT study was undertaken to rationalize the change in mechanism of this cyclization process. In addition, reaction conditions were developed, and the scope of this cyclization studied.

## Introduction

**Figure 1:**
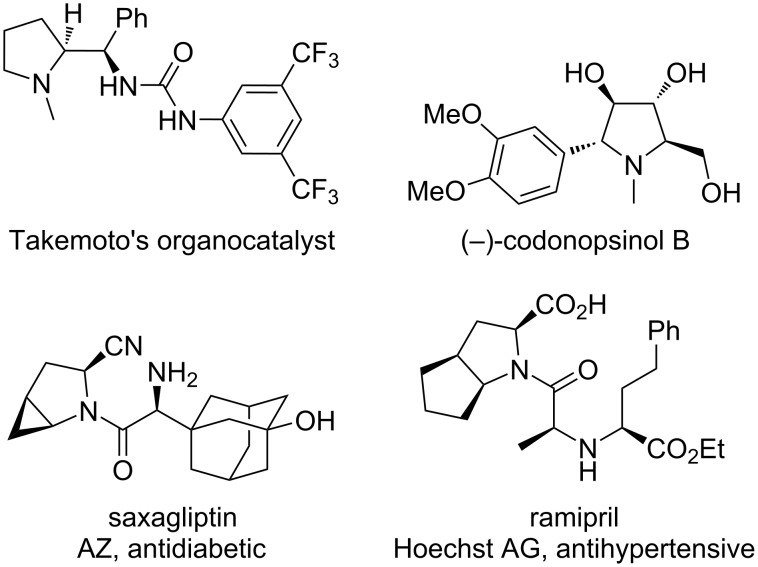
Functional molecules containing a substituted pyrrolidine core.

Proline is one of the 20 DNA-encoded proteinogenic amino acids that are essential to life [1–2]. In addition, the pyrrolidine core is present in many organocatalysts [3–5], natural products (e.g., the potent α-glucosidase inhibitor (−)-codonopsinol B) [6–7], and pharmaceutical drug molecules such as saxagliptin and ramipril (Figure 1) [8]. Accordingly, the development of methods to access substituted prolines and pyrrolidines is an important area of study as this ring system is prevalent in many useful molecules. Typical literature procedures include multistep derivations of proline itself, e.g., the destruction of the stereocenter and then its reinstallation by an enantioselective conjugate addition [9]. Other methods include the enantioselective conjugate addition to α,β-unsaturated pyroglutamic acid derivatives followed by deoxygenation [10], and the enantioselective organocatalytic reaction between 2-acylaminomalonates and α,β-unsaturated aldehydes [11–12].

**Scheme 1:**
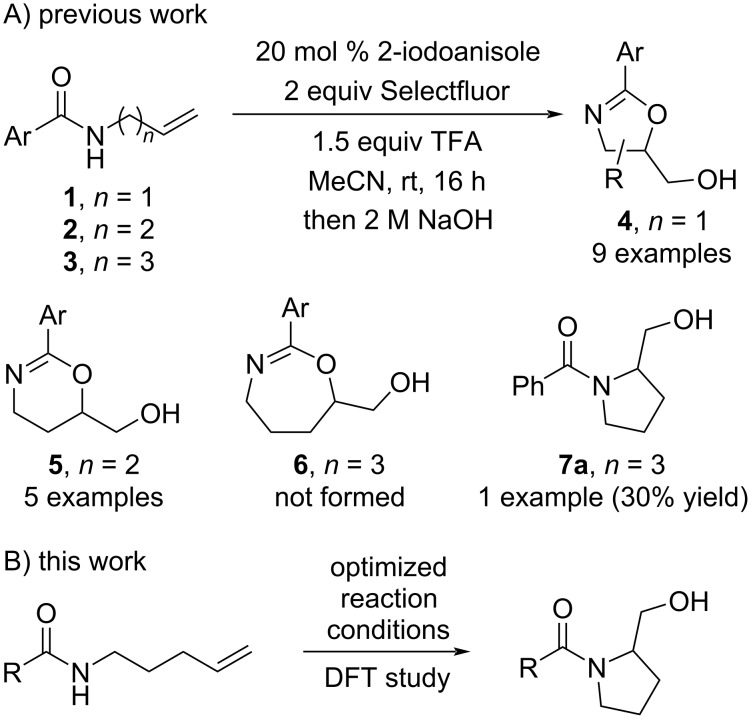
A) Our previous report on *N*-alkenylamide cyclizations. B) An overview of the present work.

The development of new synthetic methods using hypervalent iodine reagents has become increasingly popular in recent years probably due to their useful reactivity, ease of handling, and low toxicity [13]. In particular, hypervalent iodine compounds have been shown to be effective reagents and catalysts for a range of cyclization reactions [14]. In 2015, we reported the iodoarene-catalyzed cyclization of *N*-allylamides **1** and *N*-homoallylamides **2** to 2-oxazolines **4** and dihydrooxazines **5**, respectively (Scheme 1A) [15]. We also reported that an *N*-bishomoallylamide **3** (*n* = 3) was cyclized under the reaction conditions, but in just 30% yield. It turned out that the product of this reaction was the five-membered prolinol **7a** rather than the initially assigned isomeric seven-membered tetrahydrooxazepine **6** [16]. Subsequently, we set out to understand the *O*- versus *N*-chemoselectivity by DFT modelling, and to develop an effective synthetic protocol for the preparation of prolinols **7** in high yield (Scheme 1B). Notably, we are unaware of any reported method to achieve this specific transformation in the literature. Although, Tellitu and co-workers have reported a related preparation of indoline derivatives mediated by bis(trifluoroacetoxy)iodobenzene (PIFA) [17].

## Results and Discussion

**Scheme 2:**
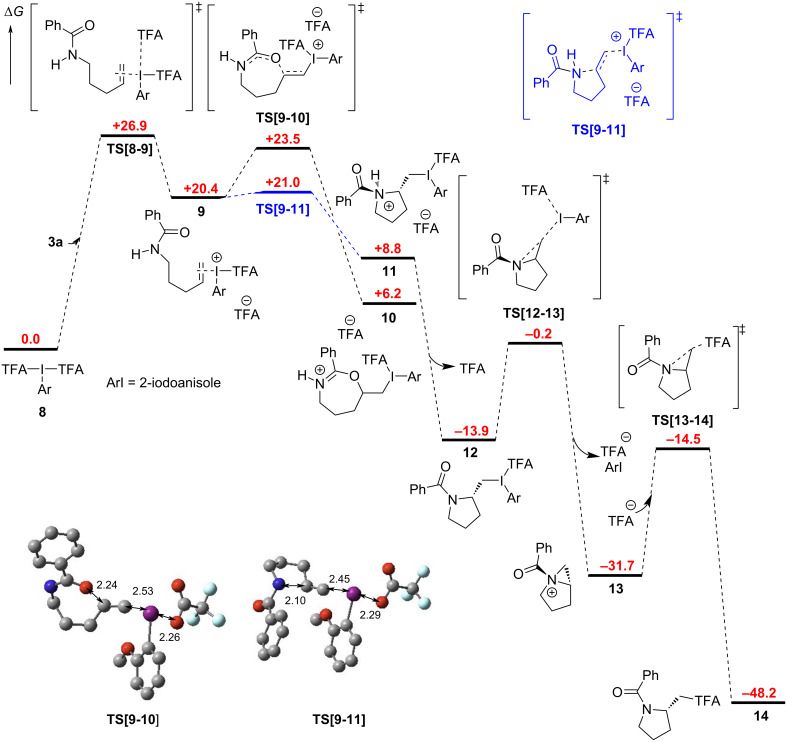
Calculated mechanism for the cyclization of amide **3a** optimized at the B3LYP/ 6-31+G(d,p) level of theory with SDDall used for iodine. Solvent effects were considered using the CPCM model. All Δ*G* values are in kcal·mol^−1^. Optimized structures are shown for the cyclization transition states (hydrogen atoms are omitted for clarity and bond lengths are given in Å).

In 2019, we reported our DFT study on the cyclization of *N*-allylbenzamide (**1a**) to the 2-oxazoline **4a**, i.e., where *n* = 1 and Ar = Ph [18]. This work indicated that the alkene is activated by the iodine(III) species and that this triggers cyclization. Intrigued by the change in mechanism from *O*- to *N*-cyclization onto the alkene when *n* = 3, we modelled this reaction using DFT calculations (Scheme 2). Similarly, we concluded that the present reaction commences with activation of the olefin in **3a** by the hypervalent iodine species **8**, which is generated under the reaction conditions. The activation occurs via an associative pathway where one of the TFA ligands dissociates from **8** upon approaching the substrate and forms the intermediate **9**. The calculated Δ*G*^‡^ value is quite high here, which could explain the low yield obtained after 16 hours. However, for the cyclization of *N*-allylbenzamide (**1a**), we found that the transition state was stabilized by 4.1 kcal·mol^−1^ by an extra molecule of trifluoroacetic acid. A similar stabilizing interaction was not identified in this case with **3a**, despite significant effort, but it cannot be ruled out. The cyclization of **9** was shown to be possible by attack of the amide at both the oxygen and the nitrogen, however, the Δ*G*^‡^ value for the latter was lower by 2.5 kcal·mol^−1^. This demonstrates a clear kinetic preference for formation of the five-membered ring over the seven-membered one [19]. Subsequent deprotonation of **11** leads to tertiary amide **12**.

Upon cyclization, the iodane moiety in **12** is eliminated by an intramolecular attack by the amide nitrogen to form the aziridinium **13**. Finally, ring-opening by S_N_2 attack of trifluoroacetate leads to the final product **14** [20]. In this case, the kinetic pyrrolidine product is obtained due to the electron-withdrawing benzoyl group on the nitrogen atom preventing equilibration to the thermodynamic piperidine product [21]. Basic workup hydrolyzes the trifluoroacetoxy ester in **14** to alcohol **7a**. Consideration of the literature NMR data for the three possible isomeric products (i.e., pyrrolidine, piperidine, and tetrahydrooxazepine) as well as DEPT, HSQC, and HMBC data for **7a** support the assignment of the pyrrolidine structure.

The next stage of the project was to improve the yield of the reaction. We initiated our study by using our initially developed conditions using 20 mol % 2-iodoanisole and found that the reaction outcome led to variable yields of product in the range of 10–25%. Increasing the quantity of 2-iodoanisole to 150 mol % provided a reproducible 30% yield of **7a** (Table 1, entry 1). We then varied the iodoarene to see the impact on the reaction outcome. Using iodobenzene, 2-iodobiphenyl, and 3-iodotoluene provided slight improvements in yield (Table 1, entries 2–4). The more electron-rich 2-iodo-1,3-dimethoxybenzene led to a further increase in yield to 44% (Table 1, entry 5). 1,2-Diiodobenzene has been reported to be a superior precatalyst in intermolecular C–H aminations of arenes but only provided 40% yield in the present case [22]. 1-Iodonaphthalene led to an increase in yield to 49% (Table 1, entry 7). 1-Iodo-2,4-dimethoxybenzene afforded the highest yield of all with 59% of tertiary amide being isolated (Table 1, entry 8). Leaving the reaction to stir for an extended period led to a further increase in yield to 68% (Table 1, entry 9). Using the even more electron-rich 2-iodo-1,3,5-trimethoxybenzene only gave 45% yield (Table 1, entry 10). Our previous studies have shown that the oxidized form of 2-iodo-1,3,5-trimethoxybenzene is unstable in solution and decomposes destructively [18]. Iodoethane was also shown to be an effective reagent furnishing the product in up to 56% upon heating to 40 °C (Table 1, entries 11 and 12). It was envisaged that oxidation of iodoethane led to formation of oxidized forms of iodide by C–I-bond cleavage, therefore tetrabutylammonium iodide was utilized to see if the result could be replicated and it was (Table 1, entry 13). Finally, the reaction was shown to occur using PIFA (bistrifluoroacetoxyiodobenzene), which is envisaged to be produced in the reaction mixture when using iodobenzene as reagent, and a similar yield was obtained (Table 1, entry 14 vs entry 2). The cyclization of amide **3a** did not occur in the absence of an iodine source. Other oxidants, solvents, and acids were screened but superior conditions were not discovered.

**Table 1:** Optimization of cyclization reaction.

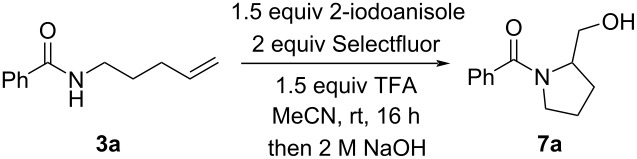		

Entry	Deviation from conditions	Yield [%]^a^

1	none	30
2	iodobenzene	37
3	2-iodobiphenyl	37
4	3-iodotoluene	39
5	2-iodo-1,3-dimethoxybenzene	44
6	1,2-diiodobenzene	40
7	1-iodonaphthalene	49
8	1-iodo-2,4-dimethoxybenzene	59
9	1-iodo-2,4-dimethoxybenzene, 48 h	68
10	2-iodo-1,3,5-trimethoxybenzene	45
11	iodoethane	38
12	iodoethane, 40 °C	56
13	Bu_4_NI	40
14	PIFA, no selectfluor, no TFA	40

^a^The yields are for isolated compounds. TFA = trifluoroacetic acid. PIFA = bis(trifluoroacetoxy)iodobenzene.

**Scheme 3:**
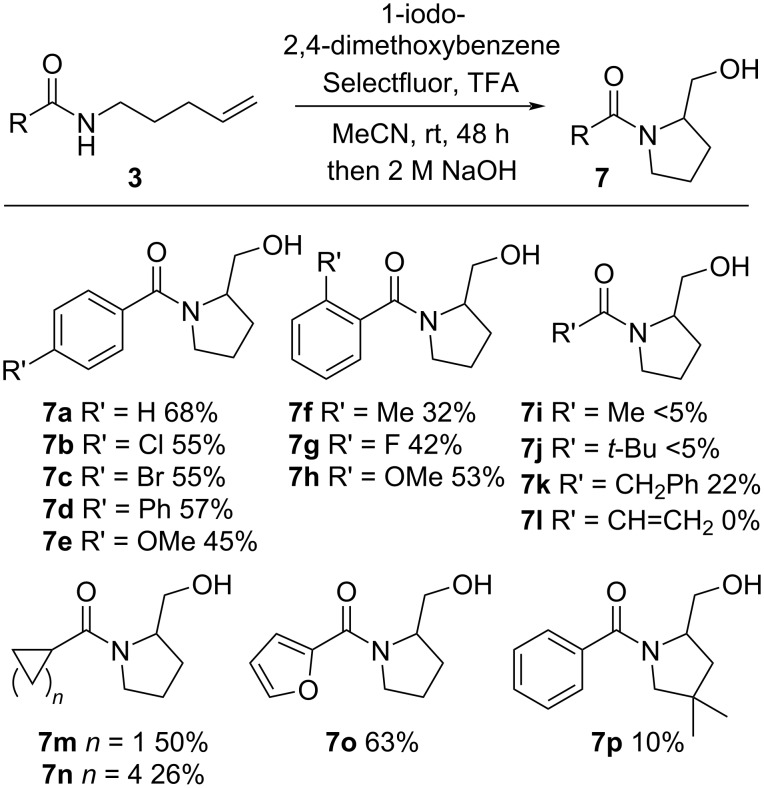
Scope of cyclization reaction.

With the optimized conditions in hand, the scope of the cyclization was investigated (Scheme 3). We examined the cyclization of *para*-substituted benzamides and chloro- (**7b**), bromo- (**7c**), phenyl- (**7d**), and methoxy- (**7e**) derivatives were all isolated in moderate yields. The *ortho*-substituted derivatives **7f**, **7g**, and **7h** were also successfully prepared. Alkylamides were found to be ambiguous substrates as the acetamide **7i** and pivalamide **7j** were formed in trace quantities whereas the benzyl derivative **7k** was isolable. The enamide **7l** was not observed. Intriguingly, cyclopropyl and cyclohexyl derivatives **7m** and **7n**, respectively, were formed and isolated in moderate yields. Furyl derivative **7o** was isolated in 63% yield. Installing a geminal dimethyl group on the alkyl linker was anticipated to lead to an improvement in cyclization, however, very low conversion was observed and the product **7p** was isolated in 10% yield.

**Scheme 4:**
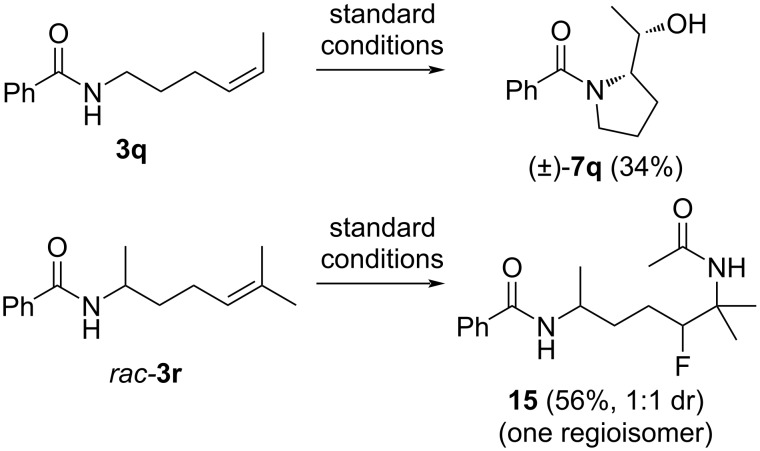
Reactions of di- and trisubstituted alkene substrates.

We then investigated the cyclization of *cis*-disubstituted alkene **3q** and were delighted to observe that only one diastereomer of **7q** was formed (Scheme 4). This result is in accordance with the calculated mechanism. The more electron-rich trisubstituted alkene **3r** reacted directly with Selectfluor leading to a tertiary carbocation which was trapped by acetonitrile in a Ritter-type process to generate bisamide **15** [23]. As would be expected, one regioisomer and a 1:1 mixture of diastereomers was formed.

## Conclusion

We have demonstrated that a change in mechanism occurs in the cyclization of *N*-alkenylamides when increasing the chain length between the amide and the alkene. When there are one or two carbon atoms separating the functional groups, cyclization at the amide oxygen occurs to generate five- and six-membered rings, respectively. However, when there is a three-carbon atom link, the corresponding seven-membered ring is not formed. Instead, cyclization at nitrogen occurs to generate a five-membered ring. We have performed DFT calculations to support the proposed change in mechanism and developed superior reaction conditions to effect this transformation. Finally, we have explored the substrate scope of this cyclization.

## Supporting Information

File 1 Experimental procedures, compound characterization data, copies of NMR spectra, cartesian coordinates and energies of calculated structures.

## Data Availability

The data that supports the findings of this study is available from the corresponding author upon reasonable request.
