# Supplementary material for: Hypervalent iodine-mediated cyclization of bishomoallylamides to prolinols
Source: Beilstein J Org Chem. 2024 Sep 30;20:2455–60. doi: 10.3762/bjoc.20.209 (PMC11457059; doi:10.3762/bjoc.20.209)
Supplement: File 1 — Experimental procedures, compound characterization data, copies of NMR spectra, cartesian coordinates and energies of calculated structures. [file Beilstein_J_Org_Chem-20-2455-s001.pdf]

BEILSTEIN JOURNAL OF ORGANIC CHEMISTRY

Supporting Information

for

Hypervalent iodine-mediated cyclization of bishomoallylamides to prolinols

Smaher E. Butt, Konrad Kepski, Jean-Marc Sotiropoulos and Wesley J. Moran

Beilstein J. Org. Chem. **2024,** *20,* 2455–2460. doi:10.3762/bjoc.20.209

Experimental procedures, compound characterization data, copies of NMR spectra, cartesian coordinates and energies of calculated structures

License and Terms: This is a supporting information file under the terms of the Creative Commons Attribution License (https://creativecommons.org/licenses/by/4.0). Please note that the reuse, redistribution and reproduction in particular requires that the author(s) and source are credited and that individual graphics may be subject to special legal provisions.
The license is subject to the *Beilstein Journal of Organic Chemistry* terms and conditions: (https://www.beilstein-journals.org/bjoc/terms)

Table of contents

General information	S2
Further optimization studies	S3
Experimental procedures and compound characterization data	S4
Cartesian coordinates and energies of calculated structures	S21
Copies of NMR spectra	S46
References	S110

General information

Chemicals were purchased from Sigma Aldrich, Fisher Scientific, or Fluorochem and were used as received without purification or drying. Solvents were used as received without drying. Thin-layer chromatography (TLC) was performed on precoated aluminum sheets of Merck silica gel 60 F254 (0.20 mm) and visualized by UV radiation (254 nm). Automated column chromatography was performed on a Biotage® Isolera Four using Biotage® SNAP Ultra cartridges. Melting points were obtained by DSC analysis. ^{1}H NMR and ^{13}C NMR spectra were measured on Bruker AV III 400 or Bruker Neo 600 apparatus and were referenced to the solvent peak. Chemical shifts δ are given in ppm and the multiplicity of the signals are reported as: s = singlet, s_{br} = broad singlet, d = doublet, t = triplet, q = quartet, sept = septet, dd = doublet of doublets, dt = doublet of triplets, dq = doublet of quartets, qd = quartet of doublets, m = multiplet. The coupling constants (*J*) are given in hertz. Mass spectrometric measurements were performed at Innovative Physical Organic Solutions (IPOS), University of Huddersfield on an Agilent 1290 HPLC + 6530 QTOF instrument. Ions were generated by electrospray ionization (ESI) and only the mass ions are reported. Spectral data for previously reported compounds are in good agreement with the literature values.

Quantum chemical calculations were performed using Gaussian 16,[1] and GaussView was used for molecular modelling.[2] The geometry optimizations were performed in the gas phase under standard conditions using DFT[3] in combination with the 6-31+G(d,p)[4] basis set for all atoms except iodine, for which the SDD (Stuttgart/Dresden) effective core potential was used.[5] All the bond lengths are given in Ångstroms. Vibrational frequency calculations were performed to determine the imaginary frequencies for the respective molecules. The imaginary frequencies verified whether the stationary points are minima, having no imaginary frequencies, or transition states, possessing one imaginary frequency. The connectivity of the transition states was confirmed by computing IRC (intrinsic reaction coordinate) from the transition-state geometry towards both the reactant and product.[6]

Solvent effects were considered using the conductor-like polarizable continuum model (CPCM).[7] Single-point calculations on the gas-phase optimized geometries were performed to estimate the change in energy in the presence of the solvent, acetonitrile. The triple-zeta quality 6-311++G(d,p) basis set along with SDD for I was used to account for the solvent effects. The Gibbs free energy values provided in the text are Gibbs energy in solution, G_{sol}, which was calculated by adding the thermochemistry corrections, $G-E$, to the refined single point energies, E_{sol}, i.e., $G_{sol} = E_{sol} + G - E$. The sums of the electronic and thermal free energies (G) for reactants and transition states were obtained by the standard procedure in the framework of the harmonic approximation. The $\Delta G^{\ddagger}$ of the reactions was calculated from the differences in the G values of the transition states and the reactants.

Further optimization studies

In addition to those described in the manuscript, the following reaction conditions were investigated:

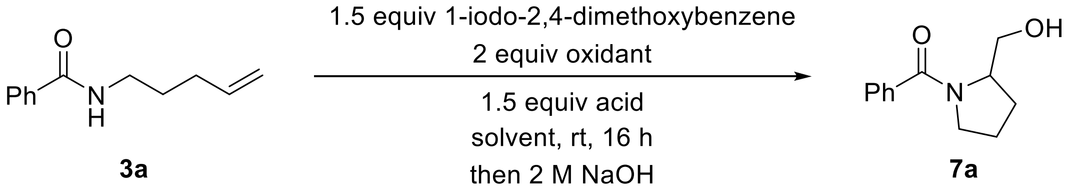

Entry	Oxidant	Acid	Solvent	Yield (%)
1	*m*-CPBA	TFA	MeCN	41
2	Selectfluor	TfOH	MeCN	27
3	Selectfluor	TFA	EtOAc	8
4	Selectfluor	TFA	DCM	4
5	Selectfluor	TFA	DCM/TFE 1:1	10
6	Selectfluor	TFA	DCM/HFIP 1:1	7

Preparation of cyclization substrates 3

Substrates were prepared in three steps using the strategy below:

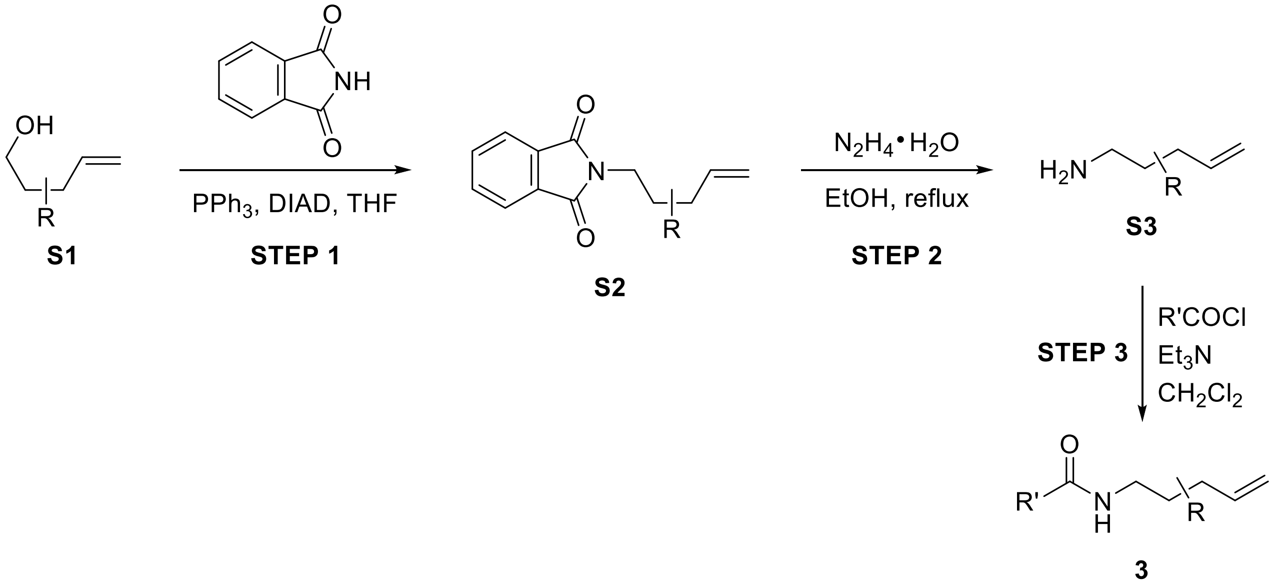

General procedure for STEP 1. Synthesis of 2-(pent-4-en-1-yl)-1*H*-isoindole-1,3(2*H*)-dione (S2a).

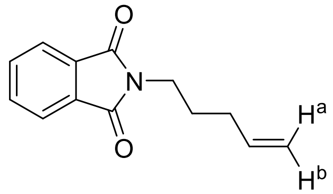

Prepared using a modified version of the procedure reported by White *et al.*[8] PPh_3 (17.5 g, 67 mmol, 1.0 equiv) was added to an oven-dried flask and purged with N_2. THF (180 mL) was added and the mixture cooled to 0 °C. DIAD (14.5 mL, 74 mmol, 1.1 equiv) was added dropwise followed by the addition of 4-penten-1-ol (6.9 mL, 67 mmol, 1.0 equiv). After 5 minutes, phthalimide (9.83 g, 67 mmol, 1.0 equiv) was added. The reaction vessel was raised out of the cooling bath and left stirring at rt overnight. All volatiles were removed under vacuum and a mixture of petroleum ether/EtOAc (10:1, 125 mL) was added. The suspension was filtered, and the filtrate was concentrated under vacuum. The crude was purified using flash chromatography (silica gel, 95:5 petrol/EtOAc) to afford the product **S2a** as a pale-yellow oil (12.12 g, 84% yield). Data matched the literature values.[1]
IR: 716 (s), 884 (m), 1047 (w), 1071 (w), 1335 (m), 1367 (m), 1393 (s), 1437 (m), 1467 (w), 1640 (w), 1703 (s), 2937 (w) cm^{-1}.
^{1}H NMR ($CDCl_3$, 600 MHz): δ 1.77 (2H, quint, J = 7.4 Hz, CH_2), 2.10 (2H, q, J = 7.3 Hz, CH_2), 3.68 (2H, t, J = 7.3 Hz, CH_2), 4.96 (1H, dq, J = 10.3, 1.5 Hz, H^b), 5.04 (1H, dq, J = 17.1, 1.7 Hz, H^a), 5.80 (1H, ddt, J = 17.2, 10.4, 6.6 Hz, CH), 7.69 (2H, dd, J = 5.5, 3.0 Hz, Ar), 7.82 (2H, dd, J = 5.5, 3.0 Hz, Ar).
^{13}C NMR ($CDCl_3$, 150 MHz): δ 27.7, 31.1, 37.7, 115.4, 123.3, 132.3, 134.0, 137.4, 168.5.
HRMS (ESI-TOF) m/z: $[M+H]^+$ Cald for $C_{13}H_{14}NO_2^+$ 216.1019; Found 216.1024.

Synthesis of 2-((4*Z*)-hex-4-en-1-yl)-1*H*-isoindole-1,3(2*H*)-dione (S2o).[9]

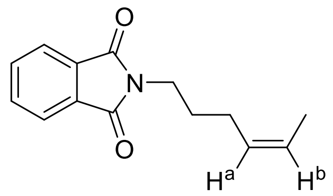

Prepared according to the general procedure for step 1, at half the scale, using *cis*-4-hexen-1-ol (3.9 mL, 33 mmol, 1.0 equiv). The crude was purified using flash chromatography (silica gel, 95:5 petrol/EtOAc) to afford the product **S2o** as a pale-yellow oil (6.26 g, 82% yield).

IR: 712 (s), 883 (w), 1017 (m), 1072 (m), 1334 (m), 1365 (m), 1392 (s), 1436 (m), 1466 (w), 1614 (w), 1703 (s), 2937 (w) cm^{-1}.

^{1}H NMR ($CDCl_3$, 400 MHz): δ 1.59 (3H, d, *J* = 6.5 Hz, Me), 1.75 (2H, quint, *J* = 7.5 Hz, CH_2), 2.11 (2H, q, *J* = 7.4 Hz, CH_2), 3.69 (2H, t, *J* = 7.5 Hz, CH_2), 5.34-5.52 (2H, m, H^a+H^b), 7.70 (2H, dd, *J* = 5.6, 3.1 Hz, Ar), 7.84 (2H, dd, *J* = 5.5, 3.1 Hz, Ar).

^{13}C NMR ($CDCl_3$, 100 MHz): δ 12.9, 24.4, 28.5, 37.9, 123.3, 125.0, 129.2, 132.3, 134.0, 168.6.

HRMS (ESI-TOF) m/z: $[M+H]^+$ Cald for $C_{14}H_{16}NO_2^+$ 230.1176; Found 230.1181.

Synthesis of 2-(6-methylhept-5-en-2-yl)-1*H*-isoindole-1,3(2*H*)-dione (S2p).

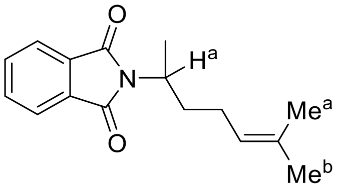

Prepared according to the general procedure for step 1, at half the scale, using 6-methyl-5-hepten-2-ol (5.1 mL, 33 mmol, 1.0 equiv). The crude was purified using flash chromatography (silica gel, 98:2 petrol/EtOAc) to afford the product **S2p** as a colorless oil (6.48 g, 75% yield).

IR: 717 (s), 879 (m), 1039 (m), 1085 (w), 1332 (m), 1355 (s), 1392 (m), 1451 (w), 1466 (w), 1613 (w), 1702 (s), 2929 (w) cm^{-1}.

^{1}H NMR ($CDCl_3$, 400 MHz): δ 1.46 (3H, d, *J* = 6.9 Hz, Me), 1.49 (3H, s, Me^a), 1.54 (3H, d, *J* = 1.0 Hz, Me^b), 1.75 (1H, ddt, *J* = 13.8, 7.5, 6.0 Hz, CH_2), 1.97 (2H, q, *J* = 7.3 Hz, CH_2), 2.16 (1H, ddt, *J* = 14.7, 9.6, 7.3 Hz, CH_2), 4.36 (1H, ddt, *J* = 14.6, 9.6, 6.9 Hz, H^a), 5.04 (1H, tt, *J* = 7.1, 1.4 Hz, CH), 7.69 (2H, dd, *J* = 5.6, 3.0 Hz, Ar), 7.81 (2H, dd, *J* = 5.6, 3.0 Hz, Ar).

^{13}C NMR ($CDCl_3$, 100 MHz): δ 17.8, 19.0, 25.6, 25.7, 33.6, 47.4, 123.1, 123.5, 132.2, 132.3, 133.9, 168.7.

HRMS (ESI-TOF) m/z: $[M+H]^+$ Cald for $C_{16}H_{20}NO_2^+$ 258.1489; Found 258.1494.

General procedure for STEP 2. Synthesis of pent-4-en-1-amine (S3a).

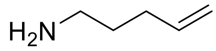

Prepared using a modified version of the procedure reported by Xu *et al.*[10] Phthalimide **S2a** (3.0 g, 14 mmol, 1.0 equiv), hydrazine monohydrate (1.0 mL, 21 mmol, 1.5 equiv) and styrene (3.2 mL, 28 mmol, 2.0 equiv) were all dissolved in ethanol (70 mL) and stirred at

reflux for 4 hours. The suspension was cooled to rt and filtered. The filtrate was acidified using HCl (1 M, 20 mL), filtered and transferred to a separating funnel. The organic layer was extracted with Et_2O (50 mL) and then discarded. The aqueous layer was basified using NaOH (4 M, 20 mL) and extracted three times with DCM (3 × 50 mL). The organic extracts were combined, dried using Na_2SO_4, and filtered. The filtrate was reduced under vacuum (20 °C, 500 mbar) to ≈ 30 mL and immediately used in the next step.

Synthesis of (*Z*)-hex-4-en-1-amine (S3o).

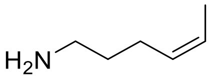

Prepared according to the general procedure for step 2 using phthalimide **S2o** (3.19 g, 14 mmol, 1.0 equiv). The crude amine was used directly in the next step.

Synthesis of 6-methylhept-5-en-2-amine (S3p).

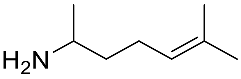

Prepared according to the general procedure for step 2 using phthalimide **S29** (2.99 g, 12 mmol, 1.0 equiv). The crude amine was used directly in the next step.

General procedure for STEP 3. Synthesis of *N*-(pent-4-en-1-yl)benzamide (3a)

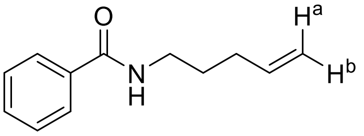

Prepared using the procedure reported by Gilmour *et al.*[3] Pent-4-en-1-amine (**S3a**, <14 mmol, 1.0 equiv) dissolved in DCM (30 mL) which was taken directly from STEP 2, was purged with N_2 and cooled to 0 °C. Et_3N (3.9 mL, 28 mmol, 2.0 equiv) was added followed by dropwise addition of benzoyl chloride (1.6 mL, 14 mmol, 1.0 equiv). The reaction vessel was raised out of the cooling bath and left stirring at rt overnight. All volatiles were removed under vacuum and Et_2O (50 mL) was added. The suspension was filtered and the filtrate was basified using NaOH (2 M, 10 mL). The resulting mixture was transferred to a separating funnel and extracted three times with Et_2O (3 × 50 mL). The organic extracts were combined, dried using Na_2SO_4, filtered and reduced under vacuum. The crude was purified by flash chromatography (silica gel, 85:15 petrol/EtOAc) to afford the product **3a** as a colorless oil (1.74 g, 66% yield). Data matched literature values.[3]
IR: 692 (s, b), 910 (m), 1204 (w), 1366 (w), 1435 (m), 1489 (m), 1603 (m), 1634 (s), 2931 (w), 3074 (w), 3307 (w, b) cm^{-1}.
^{1}H NMR ($CDCl_3$, 400 MHz): δ 1.72 (2H, quint, *J* = 7.4 Hz, CH_2), 2.16 (2H, q, *J* = 7.2 Hz, CH_2), 3.47 (2H, q, *J* = 6.7 Hz, CH_2), 5.00 (1H, dd, *J* = 10.2, 1.0 Hz, H^b), 5.06 (1H, dd, *J* = 17.2, 1.5 Hz, H^a), 5.83 (1H, ddt, *J* = 17.2, 10.4, 6.6 Hz, CH), 6.28 (1H, br s, NH), 7.41 (2H, t, *J* = 7.4 Hz, Ar), 7.48 (1H, t, *J* = 7.2 Hz, Ar), 7.75 (2H, d, *J* = 8.0 Hz, Ar).
^{13}C NMR ($CDCl_3$, 100 MHz): δ 28.9, 31.4, 39.8, 115.4, 126.9, 128.7, 131.5, 134.9, 138.0, 167.6.
HRMS (ESI-TOF) m/z: $[M+H]^+$ Cald for $C_{12}H_{16}NO^+$ 190.1226; Found 190.1238.

Synthesis of 4-chloro-*N*-(pent-4-en-1-yl)benzamide (3b)

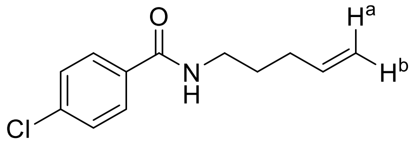

Prepared according to the general procedure for step 3 using pent-4-en-1-amine (**S3a**, 12 mmol, 1.0 equiv) in DCM (30 mL). Et_3N (3.4 mL, 24 mmol, 2.0 equiv). 4-Chlorobenzoyl chloride (1.6 mL, 12 mmol, 1.0 equiv). The crude was purified using flash chromatography (silica gel, 90:10 petrol/EtOAc) to afford the product **3b** as a white powder (1.28 g, 47% yield).

M.p.: 48-51 °C.

IR: 664 (s, b), 758 (m), 917 (s), 1210 (m), 1374 (m), 1435 (m), 1460 (m), 1483 (m), 1595 (m), 1630 (s), 2979 (m), 3074 (w), 3301 (m, br) cm^{-1}.

^1H NMR ($CDCl_3$, 400 MHz): δ 1.72 (2H, quint, *J* = 7.4 Hz, CH_2), 2.15 (2H, q, *J* = 7.2 Hz, CH_2), 3.46 (2H, q, *J* = 6.7 Hz, CH_2), 5.01 (1H, dd, *J* = 10.2, 1.0 Hz, H^b), 5.06 (1H, dd, *J* = 17.2, 1.5 Hz, H^a), 5.83 (1H, ddt, *J* = 17.2, 10.3, 6.6 Hz, CH), 6.23 (1H, br s, NH), 7.39 (2H, d, *J* = 8.5 Hz, Ar), 7.69 (2H, d, *J* = 8.5 Hz, Ar).

^{13}C NMR ($CDCl_3$, 100 MHz): δ 28.8, 31.4, 39.9, 115.5, 128.4, 128.9, 133.2, 137.7, 137.9, 166.6.

HRMS (ESI-TOF) m/z: $[M+H]^+$ Cald for $C_{12}H_{15}ClNO^+$ 224.0837; Found 224.0846.

Synthesis of 4-bromo-*N*-(pent-4-en-1-yl)benzamide (3c)

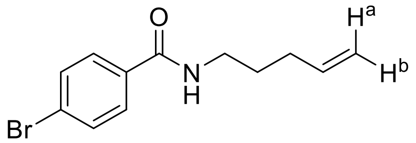

Prepared according to the general procedure for step 3 using pent-4-en-1-amine (**S3a**, 14 mmol, 1.0 equiv) in DCM (30 mL). Et_3N (3.9 mL, 28 mmol, 2.0 equiv) and 4-bromobenzoyl chloride (3.05 g, 14 mmol, 1.0 equiv). The crude was purified using flash chromatography (silica gel, 89:11 petrol/EtOAc) to afford the product as a white solid (0.45 g, 12% yield).

M.p.: 67-70 °C.

IR: 641 (m, b), 756 (m), 919 (s), 1263 (m), 1333 (m), 1436 (m), 1479 (m), 1589 (m), 1630 (s), 2979 (s), 3077 (w), 3301 (m, b) cm^{-1}.

^1H NMR ($CDCl_3$, 400 MHz): δ 1.73 (2H, quint, *J* = 7.3 Hz, CH_2), 2.16 (2H, q, *J* = 7.1 Hz, CH_2), 3.46 (2H, q, *J* = 6.7 Hz, CH_2), 5.01 (1H, dd, *J* = 10.2, 1.5 Hz, H^b), 5.07 (1H, dq, *J* = 17.2, 1.7 Hz, H^a), 5.84 (1H, ddt, *J* = 17.2, 10.3, 6.7 Hz, CH), 6.16 (1H, br s, NH), 7.56 (2H, d, *J* = 8.7 Hz, Ar), 7.62 (2H, d, *J* = 8.7 Hz, Ar).

^{13}C NMR ($CDCl_3$, 100 MHz): δ 28.8, 31.4, 39.9, 115.5, 126.1, 128.6, 131.9, 133.7, 137.9, 166.6.

HRMS (ESI-TOF) m/z: $[M+H]^+$ Cald for $C_{12}H_{15}BrNO^+$ 268.0346; Found 268.0336.

Synthesis of *N*-(pent-4-en-1-yl)[1,1'-biphenyl]-4-carboxamide (3d)

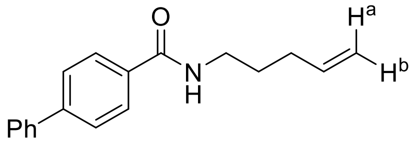

Prepared according to the general procedure for step 3 using pent-4-en-1-amine (**S3a**, 14 mmol, 1.0 equiv) in DCM (30 mL). Et_3N (3.9 mL, 28 mmol, 2.0 equiv). Biphenyl-4-carbonyl chloride (3.01 g, 13.9 mmol, 1.0 equiv). The crude was purified using flash chromatography (silica gel, 85:15 petrol/EtOAc) to afford the product as a white powder (0.46 g, 12% yield).
M.p.: 141-144 °C.
IR: 654 (m, b), 742 (s), 912 (m), 1448 (m), 1483 (m), 1608 (w), 1630 (s), 2979 (w), 3325 (w, b) cm^{-1}.
^1H NMR ($CDCl_3$, 400 MHz): δ 1.76 (2H, quint, *J* = 7.3 Hz, CH_2), 2.19 (2H, q, *J* = 7.2 Hz, CH_2), 3.51 (2H, q, *J* = 6.7 Hz, CH_2), 5.02 (1H, dd, *J* = 10.2, 1.5 Hz, H^b), 5.09 (1H, dq, *J* = 17.2, 1.7 Hz, H^a), 5.86 (1H, ddt, *J* = 17.2, 10.3, 6.6 Hz, CH), 6.21 (1H, br s, NH), 7.38 (1H, t, *J* = 7.3 Hz, Ar), 7.46 (2H, t, *J* = 7.5 Hz, Ar), 7.61 (2H, d, *J* = 7.8 Hz, Ar), 7.65 (2H, d, *J* = 8.3 Hz, Ar), 7.83 (2H, d, *J* = 8.3 Hz, Ar).
^{13}C NMR ($CDCl_3$, 100 MHz): δ 29.0, 31.4, 39.8, 115.5, 127.3, 127.4, 127.5, 128.1, 129.1, 133.6, 138.0, 140.2, 144.3, 167.3.
HRMS (ESI-TOF) m/z: $[M+H]^+$ Cald for $C_{18}H_{20}NO^+$ 266.1539; Found 266.1551.

Synthesis of 4-methoxy-*N*-(pent-4-en-1-yl)benzamide (3e)

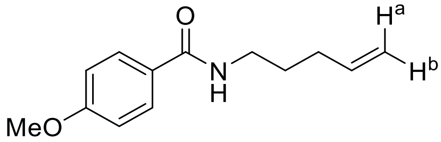

Prepared according to the general procedure for step 3 using pent-4-en-1-amine (**S3a**, 14 mmol, 1.0 equiv) in DCM (30 mL). Et_3N (3.9 mL, 28 mmol, 2.0 equiv) and 4-methoxybenzoyl chloride (1.9 mL, 14 mmol, 1.0 equiv). The crude was purified using flash chromatography (silica gel, 80:20 petrol/EtOAc) to afford the product as a white solid (0.92 g, 30% yield). Data matches the literature values.[11]
M.p.: 48-51 °C.
IR: 652 (m, b), 762 (s), 911 (m), 1250 (m), 1366 (w), 1440 (m), 1452 (m), 1462 (m), 1603 (s), 1628 (s), 2924 (w), 3079 (w), 3323 (m, b) cm^{-1}.
^1H NMR ($CDCl_3$, 400 MHz): δ 1.64 (2H, quint, *J* = 7.3 Hz, CH_2), 2.06 (2H, q, *J* = 7.3 Hz, CH_2), 3.36 (2H, q, *J* = 6.6 Hz, CH_2), 3.76 (3H, s, OMe), 4.92 (1H, dd, *J* = 10.2, 1.5 Hz, H^b), 4.98 (1H, dq, *J* = 17.2, 1.7 Hz, H^a), 5.75 (1H, ddt, *J* = 17.2, 10.3, 6.6 Hz, CH), 6.82 (2H, d, *J* = 8.9 Hz, Ar), 6.85 (1H, br s, NH), 7.73 (2H, d, *J* = 8.9 Hz, Ar).
^{13}C NMR ($CDCl_3$, 100 MHz): δ 28.8, 31.2, 39.6, 55.3, 113.5, 115.0, 127.0, 128.0, 137.9, 161.9, 167.2.
HRMS (ESI-TOF) m/z: $[M+H]^+$ Cald for $C_{13}H_{18}O_2^+$ 220.1332; Found 220.1341.

Synthesis of 2-methyl-*N*-(pent-4-en-1-yl)benzamide (3f)

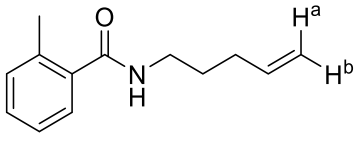

Prepared according to the general procedure for step 3 using pent-4-en-1-amine (**S3a**, 12 mmol, 1.0 equiv) in DCM (30 mL). Et_3N (3.4 mL, 24 mmol, 2.0 equiv) and *o*-toluoyl chloride (1.6 mL, 12 mmol, 1.0 equiv). The crude was purified using flash chromatography (silica gel, 90:10 petrol/EtOAc to afford the product as a colorless oil (0.84 g, 34% yield).
IR: 693 (m, b), 727 (m), 909 (m), 1205 (w), 1378 (w), 1435 (m), 1486 (m), 1600 (m), 1634 (s), 2927 (w), 3073 (w), 3271 (w, b) cm^{-1}.
^1H NMR ($CDCl_3$, 400 MHz): δ 1.71 (2H, quint, *J* = 7.3 Hz, CH_2), 2.16 (2H, q, *J* = 7.3 Hz, CH_2), 2.43 (3H, s, Me), 3.44 (2H, q, *J* = 6.7 Hz, CH_2), 5.00 (1H, dd, *J* = 10.2, 1.5 Hz, H^b), 5.06 (1H, dq, *J* = 17.2, 1.7 Hz, H^a), 5.77-5.89 (2H, m, CH+NH), 7.15-7.22 (2H, m, Ar), 7.25-7.35 (2H, m, Ar).
^{13}C NMR ($CDCl_3$, 100 MHz): δ 19.8, 29.0, 31.3, 39.4, 115.5, 125.8, 126.7, 129.8, 131.1, 136.0, 136.8, 137.8, 170.2.
HRMS (ESI-TOF) m/z: $[M+H]^+$ Cald for $C_{13}H_{18}NO^+$ 204.1383; Found 204.1374.

Synthesis of 2-fluoro-*N*-(pent-4-en-1-yl)benzamide (3g)

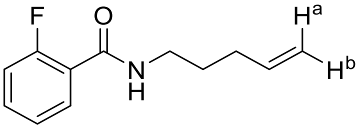

Prepared according to the general procedure for step 3 using pent-4-en-1-amine (**S3a**, 14 mmol, 1.0 equiv) in DCM (30 mL). Et_3N (3.9 mL, 28 mmol, 2.0 equivand 2-fluorobenzoyl chloride (1.7 mL, 14mmol, 1.0 equiv. The crude was purified using flash chromatography (silica gel, 90:10 petrol/EtOAc) to afford the product as a yellow oil (1.76 g, 61% yield).
IR: 912 (m), 1223 (m), 1366 (w), 1451 (m), 1480 (m), 1614 (m), 1639 (s), 2932 (w), 3078 (w), 3297 (w, b) cm^{-1}.
^1H NMR ($CDCl_3$, 400 MHz): δ 1.72 (2H, quint, *J* = 7.3 Hz, CH_2), 2.15 (2H, q, *J* = 7.3 Hz, CH_2), 3.48 (2H, q, *J* = 6.7 Hz, CH_2), 4.99 (1H, dq, *J* = 10.2, 1.5 Hz, H^b), 5.05 (1H, dq, *J* = 17.2, 1.7 Hz, H^a), 5.82 (1H, ddt, *J* = 17.2, 10.3, 6.7 Hz, CH), 6.78 (1H, br s, NH), 7.08 (1H, ddd, *J* = 12.3, 8.3, 1.0 Hz, Ar), 7.23 (1H, td, *J* = 7.6, 1.1 Hz, Ar), 7.39-7.47 (1H, m, Ar), 8.06 (1H, td, *J* = 7.9, 1.9 Hz, Ar).
^{13}C NMR ($CDCl_3$, 100 MHz): δ 28.7, 31.3, 39.6, 115.5, 116.1 (d, *J* = 24.9 Hz), 121.3 (d, *J* = 11.8 Hz), 124.9 (d, *J* = 3.4 Hz), 132.2 (d, *J* = 2.2 Hz), 133.3 (d, *J* = 9.2 Hz), 137.8, 160.7 (d, *J* = 247 Hz), 163.4 (d, *J* = 3.2 Hz).
^{19}F ($CDCl_3$, 376 MHz): -114.0.
HRMS (ESI-TOF) m/z: $[M+H]^+$ Cald for $C_{12}H_{15}FNO^+$ 208.1132; Found 208.1132.

Synthesis of 2-methoxy-*N*-(pent-4-en-1-yl)benzamide (3h)

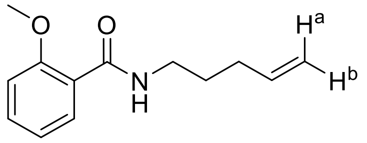

Prepared according to the general procedure for step 3 using pent-4-en-1-amine (**S3a**, 14 mmol, 1.0 equiv) in DCM (30 mL). Et_3N (3.9 mL, 28 mmol, 2.0 equiv) and *o*-methoxybenzoyl chloride (2.0 mL, 14 mmol, 1.0 equiv). The crude was purified using flash chromatography (silica gel, 80:20 petrol/EtOAc) to afford the product as a yellow oil (1.07 g, 35% yield).
IR: 655 (m, b), 753 (s), 909 (m), 1236 (s), 1435 (m), 1464 (m), 1482 (m), 1598 (m), 1642 (s), 2931 (w), 3075 (w), 3404 (w, b) cm^{-1}.
^1H NMR ($CDCl_3$, 400 MHz): δ 1.72 (2H, quint, *J* = 7.2 Hz, CH_2), 2.16 (2H, q, *J* = 7.2 Hz, CH_2), 3.48 (2H, q, *J* = 6.6 Hz, CH_2), 3.96 (3H, s, OMe), 4.99 (1H, dq, *J* = 10.3, 1.5 Hz, H^b), 5.06 (1H, dq, *J* = 17.1, 1.7 Hz, H^a), 5.84 (1H, ddt, *J* = 17.2, 10.3, 6.7 Hz, CH), 6.96 (1H, d, *J* = 8.5 Hz, Ar), 7.07 (1H, td, *J* = 7.6, 0.8 Hz, Ar), 7.40-7.45 (1H, m, Ar), 7.87 (1H, br s, NH), 8.21 (1H, dd, *J* = 7.8, 1.8 Hz, Ar).
^{13}C NMR ($CDCl_3$, 100 MHz): δ 28.9, 31.4, 39.3, 56.0, 111.4, 115.2, 121.4, 121.8, 132.4, 132.7, 138.0, 157.5, 165.3.
HRMS (ESI-TOF) m/z: $[M+Na]^+$ Cald for $C_{13}H_{18}NO_2^+$ 242.1151; Found 242.1157.

Synthesis of *N*-(pent-4-en-1-yl)acetamide (3i)

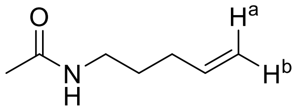

Prepared according to the general procedure for step 3 using pent-4-en-1-amine (**S3a**, 14 mmol, 1.0 equiv) in DCM (30 mL). Et_3N (3.9 mL, 28 mmol, 2.0 equiv) and acetyl chloride (1.0 mL, 14 mmol, 1.0 equiv). The crude was purified using flash chromatography (silica gel, 80:20 petrol/EtOAc) to afford the product as a yellow oil (0.60 g, 34% yield). Data matched the literature values.[4]
IR: 604 (m, b), 724 (w), 910 (m), 1289 (m), 1366 (m), 1640 (m), 2929 (w), 3079 (w), 3287 (w, b) cm^{-1}.
^1H NMR ($CDCl_3$, 400 MHz): δ 1.58 (2H, quint, *J* = 7.3 Hz, CH_2), 1.94 (3H, s, Me), 2.06 (2H, q, *J* = 7.3 Hz, CH_2), 3.22 (2H, q, *J* = 6.6 Hz, CH_2), 4.95 (1H, dq, *J* = 10.3, 1.4 Hz, H^b), 5.00 (1H, dq, *J* = 17.2, 1.7 Hz, H^a), 5.77 (1H, ddt, *J* = 17.0, 10.2, 6.7 Hz, CH), 5.86 (1H, br s, NH).
^{13}C NMR ($CDCl_3$, 100 MHz): δ 23.4, 28.8, 31.2, 39.3, 115.3, 137.9, 170.2.
HRMS (ESI-TOF) m/z: $[M+H]^+$ Cald for $C_7H_{14}NO^+$ 128.1070; Found 128.1076.

Synthesis of 2,2-dimethyl-I-(pent-4-en-1-yl)propanamide (3j)

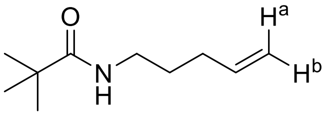

Prepared according to the general procedure for step 3 using pent-4-en-1-amine (**S3a**, 12 mmol, 1.0 equiv) in DCM (30 mL). Et_3N (3.4 mL, 24 mmol, 2.0 equiv) and trimethylacetyl chloride (1.5 mL, 12 mmol, 1.0 equiv). The crude was purified using flash

chromatography (silica gel, 85:15 petrol/EtOAc) to afford the product as a pale-brown oil (0.64 g, 31% yield).
IR: 640 (w, b), 908 (m), 1210 (m), 1366 (w), 1480 (w), 1635 (s), 2963 (w), 3077 (w), 3342 (w, b) cm^{-1}.
^1H NMR ($CDCl_3$, 400 MHz): δ 1.18 (9H, s, Me), 1.60 (2H, quint, *J* = 7.2 Hz, CH_2), 2.08 (2H, q, *J* = 7.2 Hz, CH_2), 3.25 (2H, q, *J* = 6.7 Hz, CH_2), 4.98 (1H, dd, *J* = 10.3, 1.0 Hz, H^b), 5.03 (1H, dq, *J* = 17.2, 1.7 Hz, H^a), 5.67 (1H, br s, NH), 5.80 (1H, ddt, *J* = 17.1, 10.3, 6.7 Hz, CH).
^{13}C NMR ($CDCl_3$, 100 MHz): δ 27.7, 28.8, 31.3, 38.8, 39.2, 115.3, 138.1, 178.5.
HRMS (ESI-TOF) m/z: $[M+H]^+$ Cald for $C_{10}H_{20}NO^+$ 170.1539; Found 170.1547.

Synthesis of *N*-(pent-4-en-1-yl)(phenyl)acetamide (3k)

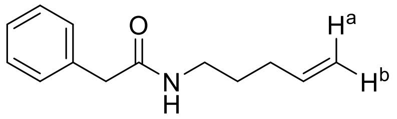

Prepared according to the general procedure for step 3 using pent-4-en-1-amine (**S3a**, 14 mmol, 1.0 equiv) in DCM (30 mL). Et_3N (3.9 mL, 28 mmol, 2.0 equiv) and phenylacetyl chloride (1.8 mL, 14 mmol, 1.0 equiv). The crude was purified using flash chromatography (silica gel, 83:17 petrol/EtOAc) to afford the product as a yellow solid (1.07 g, 38% yield).
M.p.: 39-42 °C.
IR: 692 (s), 754 (m), 909 (m), 1270 (m), 1346 (m), 1453 (m), 1472 (m), 1491 (m), 1626 (m), 1655 (m), 2932 (m), 3063 (m), 3242 (m, b) cm^{-1}.
^1H NMR ($CDCl_3$, 400 MHz): δ 1.52 (2H, quint, *J* = 7.2 Hz, CH_2), 1.99 (2H, q, *J* = 7.2 Hz, CH_2), 3.21 (2H, q, *J* = 6.5 Hz, CH_2), 3.56 (2H, s, $ArCH_2$), 4.88-4.97 (2H, m, H^a+H^b), 5.45 (1H, br s, NH), 5.72 (1H, ddt, *J* = 17.1, 10.2, 6.7 Hz, CH), 7.23-7.39 (5H, m, Ar).
^{13}C NMR ($CDCl_3$, 100 MHz): δ 28.6, 31.2, 39.2, 44.0, 115.3, 127.5, 129.1, 129.6, 135.1, 137.8, 171.0.
HRMS (ESI-TOF) m/z: $[M+H]^+$ Cald for $C_{13}H_{18}NO^+$ 204.1383; Found 204.1388.

Synthesis of *N*-(pent-4-en-1-yl)prop-2-enamide (3l)

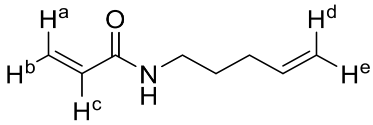

Prepared according to the general procedure for step 3 using pent-4-en-1-amine (**S3a**, 14 mmol, 1.0 equiv) in DCM (30 mL). Et_3N (3.9 mL, 28 mmol, 2.0 equiv) and acryloyl chloride (1.1 mL, 14 mmol, 1.0 equiv). The crude was purified using flash chromatography (silica gel, 75:25 petrol/EtOAc) to afford the product as a pale-yellow oil (0.47 g, 24% yield). Data matched the literature values.[12]
IR: 648 (w, b), 710 (w), 911 (m), 1242 (m), 1655 (m), 2931 (w), 3078 (w), 3284 (w, b) cm^{-1}.
^1H NMR ($CDCl_3$, 400 MHz): δ 1.64 (2H, quint, *J* = 7.3 Hz, CH_2), 2.10 (2H, q, *J* = 7.3 Hz, CH_2), 3.34 (2H, q, *J* = 6.7 Hz, CH_2), 4.98 (1H, dd, *J* = 10.3, 1.1 Hz, H^e), 5.03 (1H, dq, *J* = 17.2, 1.7 Hz, H^d), 5.61 (1H, dd, *J* = 10.3, 1.4 Hz, H^b), 5.74-5.85 (2H, m, CH+NH), 6.08 (1H, dd, *J* = 16.9, 10.3 Hz, H^c), 6.26 (1H, dd, *J* = 17.1, 1.5 Hz, H^a).
^{13}C NMR ($CDCl_3$, 100 MHz): δ 28.8, 31.2, 39.3, 115.4, 126.3, 131.1, 137.8, 165.7.
HRMS (ESI-TOF) m/z: $[M+H]^+$ Cald for $C_8H_{14}NO^+$ 140.1070; Found 140.1073.

Synthesis of *N*-(pent-4-en-1-yl)cyclopropanecarboxamide (3m)

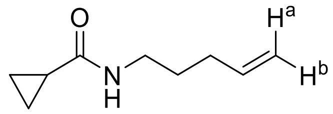

Prepared according to the general procedure for step 3 using pent-4-en-1-amine (**S3a**, 14 mmol, 1.0 equiv) in DCM (30 mL). Et_3N (3.9 mL, 28 mmol, 2.0 equiv) and cyclopropanecarbonyl chloride (1.3 mL, 14 mmol, 1.0 equiv). The crude was purified using flash chromatography (silica gel, 85:15 petrol/EtOAc) to afford the product as a pale-yellow oil (0.93 g, 44% yield).

^{1}H NMR ($CDCl_3$, 400 MHz): δ 0.65-0.73 (2H, m, cycloprop), 0.88-0.95 (2H, m, cycloprop), 1.28-1.37 (1H, m, cyclopropCH), 1.59 (2H, quint, *J* = 7.2 Hz, CH_2), 2.07 (2H, q, *J* = 7.3 Hz, CH_2), 3.25 (2H, q, *J* = 6.8 Hz, CH_2), 4.96 (1H, dd, *J* = 10.3, 1.1 Hz, H^b), 5.01 (1H, dd, *J* = 17.0, 1.6, Hz H^a), 5.79 (1H, ddt, *J* = 17.2, 10.4, 6.5 Hz, CH), 5.94 (1H, br s, NH).

^{13}C NMR ($CDCl_3$, 100 MHz): δ 7.0, 14.8, 29.0, 31.2, 39.3, 115.2, 137.9, 173.6.

IR: 640 (w, b), 910 (m), 1242 (m), 1364 (w), 1640 (s), 2932 (w), 3080 (w), 3290 (w, b) cm^{-1}.

HRMS (ESI-TOF) m/z: $[M+H]^+$ Cald for $C_9H_{20}NO^+$ 154.1226; Found 154.1229.

Synthesis of *N*-(pent-4-en-1-yl)cyclohexanecarboxamide (3n)

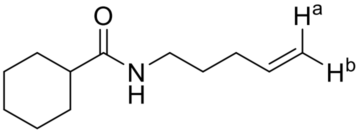

Prepared according to the general procedure for step 3 using pent-4-en-1-amine (**S3a**, 14 mmol, 1.0 equiv) in DCM (30 mL). Et_3N (3.9 mL, 28 mmol, 2.0 equiv) and cyclohexanecarbonyl chloride (1.9 mL, 14 mmol, 1.0 equiv). The crude was purified using flash chromatography (silica gel, 86:14 petrol/EtOAc) to afford the product as a white solid (0.77 g, 28% yield).

M.p.: 39-42 °C.

IR: 697 (m, b), 747 (w), 911 (m), 1256 (m), 1392 (m), 1636 (s), 2928 (s), 3093 (w), 3286 (m, b) cm^{-1}.

^{1}H NMR ($CDCl_3$, 400 MHz): δ 1.19-1.31 (3H, m, cyclohex), 1.34-1.47 (2H, m, cyclohex), 1.53-1.68 (3H, m, cyclohexCH+CH_2), 1.72-1.87 (4H, m, cyclohex), 2.03-2.10 (3H, m, CH_2), 3.23 (2H, q, *J* = 6.7 Hz, CH_2), 4.95 (1H, dd, *J* = 10.2, 1.5, Hz, H^b), 5.01 (1H, dq, *J* = 17.2, 1.7 Hz, H^a), 5.63 (1H, br s, NH), 5.78 (1H, ddt, *J* = 17.1, 10.2, 6.7 Hz, CH).

^{13}C NMR ($CDCl_3$, 100 MHz): δ 25.9, 28.9, 29.8, 31.2, 38.9, 45.7, 115.2, 138.0, 176.2.

HRMS (ESI-TOF) m/z: $[M+H]^+$ Cald for $C_{12}H_{22}NO^+$ 196.1696; Found 196.1702.

Synthesis of *N*-(pent-4-en-1-yl)furan-2-carboxamide (3o)

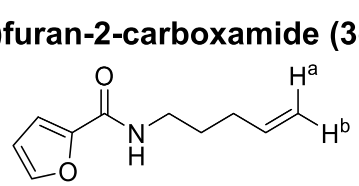

Prepared according to the general procedure for step 3 using pent-4-en-1-amine (**S3a**, 12 mmol, 1.0 equiv) in DCM (30 mL). Et_3N (3.4 mL, 24 mmol, 2.0 equiv). 2-Furoyl chloride (1.2 mL, 12 mmol, 1.0 equiv). The crude was purified using flash chromatography (silica gel, 85:15 petrol/EtOAc) to afford the product as a yellow oil (0.80 g, 36% yield).

IR: 678 (w, b), 749 (s), 911 (m), 1242 (w), 1376 (w), 1475 (m), 1639 (s), 2932 (m), 3076 (w), 3297 (m, b) cm^{-1}.
^{1}H NMR ($CDCl_3$, 400 MHz): δ 1.70 (2H, quint, *J* = 7.3 Hz, CH_2), 2.14 (2H, q, *J* = 7.2 Hz, CH_2), 3.43 (2H, q, *J* = 6.7 Hz, CH_2), 5.00 (1H, dd, *J* = 10.2, 1.5 Hz, H^b), 5.06 (1H, dq, *J* = 17.2, 1.7 Hz, H^a), 5.82 (1H, ddt, *J* = 17.2, 10.3, 6.6 Hz, CH), 6.40 (1H, br s, NH), 6.48 (1H, dd, *J* = 3.5, 1.7 Hz, furan), 7.09 (1H, d, *J* = 3.5 Hz, furan), 7.41 (1H, d, *J* = 1.0 Hz, furan).
^{13}C NMR ($CDCl_3$, 100 MHz): δ 28.9, 31.2, 38.8, 112.2, 114.1, 115.4, 137.8, 143.8, 148.3, 158.5.
HRMS (ESI-TOF) m/z: $[M+H]^+$ Cald for $C_{10}H_{14}NO_2^+$ 180.1019; Found 180.1024.

Synthesis of *N*-(2,2-dimethylpent-4-en-1-yl)benzamide (3p)

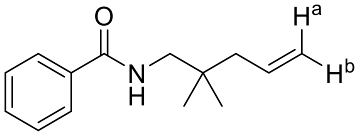

Prepared according to the general procedure for step 3 using 2,2-dimethylpent-4-en-1-amine[13] (20 mmol, 1.0 equiv) in DCM (50 mL). Et_3N (5.6 mL, 40 mmol, 2.0 equiv) and benzoyl chloride (2.3 mL, 20 mmol, 1.0 equiv). The crude was purified using flash chromatography (silica gel, 90:10 petrol/EtOAc) to afford the product as a yellow oil (0.14 g, 3% yield). Data matched the literature values.[14]
IR: 913 (m), 1204 (w), 1367 (m), 1431 (w), 1489 (w), 1579 (w), 1639 (m), 2913 (w), 3328 (m, b) cm^{-1}.
^{1}H NMR ($CDCl_3$, 400 MHz): δ 0.97 (6H, s, Me), 2.06 (2H, d, *J* = 7.5 Hz, CH_2), 3.31 (2H, d, *J* = 6.4 Hz, CH_2), 5.05-5.12 (2H, m, H^a+H^b), 5.89 (1H, ddt, *J* = 16.7, 7.5, 7.4, Hz, CH), 6.23 (1H, br s, NH), 7.43 (2H, t, *J* = 7.3 Hz, Ar), 7.49 (1H, t, *J* = 7.3 Hz, Ar), 7.75 (2H, d, *J* = 7.8 Hz, Ar).
^{13}C NMR ($CDCl_3$, 100 MHz): δ 25.2, 35.2, 45.0, 49.5, 117.7, 126.9, 128.7, 131.5, 135.1, 135.2, 167.7.
HRMS (ESI-TOF) m/z: $[M+H]^+$ Cald for $C_{14}H_{19}NO$ 218.1539; Found 218.1547.

Synthesis of *N*-((4*Z*)-hex-4-en-1-yl)benzamide (3q)

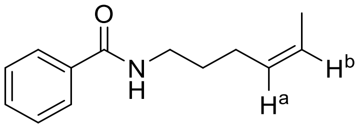

Prepared according to the general procedure for step 3 using (*Z*)-hex-4-en-1-amine (**S3o**, 14 mmol, 1.0 equiv) in DCM (30 mL). Et_3N (3.9 mL, 28 mmol, 2.0 equiv) and benzoyl chloride (1.6 mL, 14 mmol, 1.0 equiv). The crude was purified using flash chromatography (silica gel, 90:10 petrol/EtOAc) to afford the product as a pale-yellow oil (1.46 g, 52% yield).
IR: 691 (s, b), 1369 (w), 1435 (w), 1489 (m), 1602 (m), 1634 (m), 2932 (w), 3310 (w, b) cm^{-1}.
^{1}H NMR ($CDCl_3$, 400 MHz): δ 1.61 (3H, d, *J* = 6.7 Hz, Me), 1.68 (2H, quint, *J* = 7.3 Hz, CH_2), 2.14 (2H, q, *J* = 7.2 Hz, CH_2), 3.45 (2H, q, *J* = 6.7 Hz, CH_2), 5.36-5.55 (2H, m, H^a+H^b), 6.35 (1H, br s, NH), 7.40 (2H, t, *J* = 7.4 Hz, Ar), 7.47 (1H, t, *J* = 7.3 Hz, Ar), 7.75 (2H, d, *J* = 8.0 Hz, Ar).

^{13}C NMR ($CDCl_3$, 100 MHz): δ 12.9, 24.5, 29.5, 39.9, 125.0, 126.9, 128.6, 129.6, 131.4, 134.9, 167.6.
HRMS (ESI-TOF) m/z: $[M+H]^+$ Cald for $C_{13}H_{18}NO^+$ 204.1383; Found 204.1384.

Synthesis of *N*-(6-methylhept-5-en-2-yl)benzamide (3r)

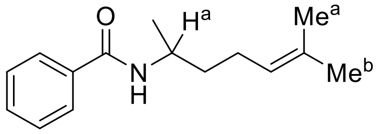

Prepared according to the general procedure for step 3 using 6-methylhept-5-en-2-amine (**S3p**, 12 mmol, 1.0 equiv) in DCM (30 mL). Et_3N (3.2 mL, 23 mmol, 2.0 equiv) and benzoyl chloride (1.3 mL, 11.6 mmol, 1.0 equiv). The crude was purified using flash chromatography (silica gel, 90:10 petrol/EtOAc) to afford the product as an orange powder (0.18 g, 7% yield).
M.p.: 84-87 °C.
IR: 664 (m, b), 746 (w), 893 (w), 1278 (w), 1352 (m), 1450 (m), 1492 (m), 1602 (m), 1629 (m), 2922 (m), 3302 (w, b) cm^{-1}.
^1H NMR ($CDCl_3$, 400 MHz): δ 1.24 (3H, d, *J* = 6.6 Hz, Me), 1.56-1.66 (6H, m, Me^a+CH_2), 1.68 (3H, d, *J* = 0.9 Hz, Me^b), 2.05-2.13 (2H, m, CH_2), 4.21 (1H, ddt, *J* = 13.3, 12.7, 6.7 Hz, H^a), 5.14 (1H, tt, *J* = 7.2, 1.4 Hz, CH), 5.96 (1H, br d, *J* = 7.2 Hz, NH), 7.42 (2H, t, *J* = 7.4 Hz, Ar), 7.48 (1H, t, 7.3 Hz, Ar), 7.74 (1H, d, *J* = 7.8 Hz, Ar).
^{13}C NMR ($CDCl_3$, 100 MHz): δ 17.8, 21.1, 24.8, 25.9, 37.0, 45.8, 123.9, 126.9, 128.7, 131.4, 132.4, 135.2, 166.9.
HRMS (ESI-TOF) m/z: $[M+H]^+$ Cald for $C_{15}H_{22}NO^+$ 232.1696; Found 232.1703.

General procedure for the cyclization reaction: synthesis of (2-(hydroxymethyl)pyrrolidin-1-yl)(phenyl)methanone (7a)

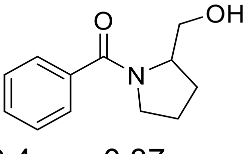

N-(Pent-4-en-1-yl)benzamide (**3a**, 69.4 mg, 0.37 mmol, 1.0 equiv) was dissolved in MeCN (3.0 mL) at room temperature. TFA (42 µL, 0.55 mmol, 1.5 equiv) was added followed by Selectfluor (260 mg, 0.73 mmol, 2.0 equiv). After 5 minutes, 1-iodo-2,4-dimethoxybenzene (145 mg, 0.55 mmol, 1.5 equiv) was added and the mixture was left stirring for 48 hours. The resulting mixture was basified with aqueous NaOH solution (2 M, 2.0 mL), stirred for 10 minutes, then transferred to a separating funnel containing a saturated aqueous solution of sodium thiosulfate pentahydrate (5.0 mL). After vigorous shaking, the organic layer was extracted three times with EtOAc (3 × 10 mL). The organic extracts were combined, dried using Na_2SO_4, filtered, and concentrated under vacuum. The crude was purified by flash chromatography (silica gel, 35:65 petrol/EtOAc) to afford the product as a brown oil (51.3 mg, 68% yield). Data matched the literature values.[15]
IR: 700 (s), 1027 (m), 1421 (s), 1598 (s), 2970 (m), 3378 (w, b) cm^{-1}.
^1H NMR ($CDCl_3$, 400 MHz): δ 1.58-1.78 (2H, m, CH_2), 1.80-1.90 (1H, m, CH_2), 2.08-2.18 (1H, m, CH_2), 3.40-3.52 (2H, m, CH_2), 3.67-3.81 (2H, m, CH_2OH), 4.32-4.41 (1H, m, CH), 4.94 (1H, br s, OH), 7.35-7.42 (3H, m, Ar), 7.48 (2H, d, *J* = 6.5 Hz, Ar).

^{13}C NMR ($CDCl_3$, 100 MHz): δ 25.0 (CH), 28.5 (CH), 51.2 (CH_2), 61.4 (CH), 67.0 (CH_2), 127.1 (CH), 128.4 (CH), 130.2 (CH), 136.7 (C), 172.2 (C).
HRMS (ESI-TOF) m/z: $[M+H]^+$ Cald for $C_{12}H_{16}NO_2^+$ 206.1176; Found 206.1175.

Synthesis of (4-chlorophenyl)(2-(hydroxymethyl)pyrrolidin-1-yl)methanone (7b)

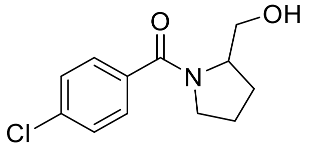

Prepared according to the general procedure for the cyclization reaction using 4-chloro-*N*-(pent-4-en-1-yl)benzamide (**3b**, 83 mg, 0.37 mmol, 1.0 equiv). The crude was purified using flash chromatography (silica gel, 40:60 petrol/EtOAc) to afford the product as a brown oil (49 mg, 55% yield). Data matched the literature values.[16]
IR: 756 (m), 1014 (m), 1422 (s), 1595 (s), 2969 (w), 3398 (w, b) cm^{-1}.
^1H NMR ($CDCl_3$, 400 MHz): δ 1.60-1.80 (2H, m, CH_2), 1.83-1.92 (1H, m, CH_2), 2.08-2.19 (1H, m, CH_2), 3.38-3.52 (2H, m, CH_2), 3.64-3.84 (2H, m, CH_2OH), 4.29-4.39 (1H, m, CH), 4.73 (1H, br s, OH), 7.36 (2H, d, J = 8.4 Hz, Ar), 7.44 (2H, d, J = 8.4 Hz, Ar).
^{13}C NMR ($CDCl_3$, 150 MHz): δ 25.1, 28.4, 51.2, 61.5, 66.8, 128.7, 135.0, 136.3, 171.0.
HRMS (ESI-TOF) m/z: $[M+H]^+$ Cald for $C_{12}H_{15}ClNO_2^+$ 240.0786; Found 240.0783.

Synthesis of (4-bromophenyl)(2-(hydroxymethyl)pyrrolidin-1-yl)methanone (7c)

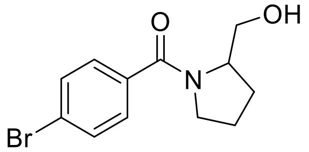

Prepared according to the general procedure for the cyclization reaction using 4-bromo-*N*-(pent-4-en-1-yl)benzamide (**3c**, 81 mg, 0.30 mmol, 1.0 equiv). The crude was purified using flash chromatography (silica gel, 40:60 petrol/EtOAc) to afford the product as a brown oil (47 mg, 55% yield). Data matched the literature values.[7]
IR: 754 (m), 1047 (m), 1422 (s), 1589 (s), 2970 (w), 3362 (w, b) cm^{-1}.
^1H NMR ($CDCl_3$, 400 MHz): δ 1.60-1.79 (2H, m, CH_2), 1.82-1.91 (1H, m, CH_2), 2.08-2.17 (1H, m, CH_2), 3.40-3.47 (1H, m, CH_2), 3.65-3.73 (1H, m, CH_2OH), 3.73-3.80 (1H, m, CH_2OH), 4.29-4.38 (1H, m, CH), 4.73 (1H, br s, OH), 7.36 (2H, d, J = 8.2 Hz, Ar), 7.52 (2H, d, J = 8.2 Hz, Ar).
^{13}C NMR ($CDCl_3$, 100 MHz): δ 25.0, 28.4, 51.2, 61.4, 66.6, 124.6, 128.8, 131.6, 135.5, 171.0.
HRMS (ESI-TOF) m/z: $[M+H]^+$ Cald for $C_{12}H_{15}NO_2^+$ 284.0281; Found 284.0290.

Synthesis of [1,1'-biphenyl]-4-yl(2-(hydroxymethyl)pyrrolidin-1-yl)methanone (7d)

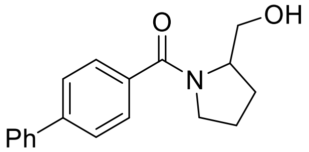

Prepared according to the general procedure for the cyclization reaction using *N*-(pent-4-en-1-yl)[1,1'-biphenyl]-4-carboxamide (**3d**, 71 mg, 0.27 mmol, 1.0 equiv). The crude was purified using flash chromatography (silica gel, 40:60 petrol/EtOAc) to afford the product as a brown oil (43 mg, 57% yield). Data matched the literature values.[7]

IR: 747 (s), 1031 (m), 1426 (s), 1599 (s), 2970 (w), 3346 (w, b) cm^{-1}.

^1H NMR ($CDCl_3$, 400 MHz): δ 1.61-1.82 (2H, m, CH_2), 1.84-1.95 (1H, m, CH_2), 2.10-2.25 (1H, m, CH_2), 3.48-3.63 (2H, m, CH_2), 3.70-3.89 (2H, m, CH_2OH), 4.35-4.50 (1H, m, CH), 4.95 (1H, br d, *J* = 5.0 Hz, OH), 7.37 (1H, t, *J* = 7.3 Hz, Ar), 7.45 (2H, t, *J* = 7.5 Hz, Ar), 7.54-7.66 (6H, m, Ar).

^{13}C NMR ($CDCl_3$, 100 MHz): δ 25.2, 28.6, 51.3, 61.7 67.3, 127.1, 127.2, 127.7, 128.0, 129.0, 135.4, 140.2, 143.2, 172.1.

HRMS (ESI-TOF) m/z: $[M+H]^+$ Cald for $C_{18}H_{20}NO_2^+$ 282.1489; Found 282.1498.

Synthesis of (2-(hydroxymethyl)pyrrolidin-1-yl)(4-methoxyphenyl)methanone (7e)

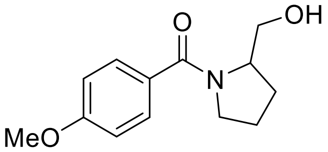

Prepared according to the general procedure for the cyclization reaction using 4-methoxy-*N*-(pent-4-en-1-yl)benzamide (**3e**, 75 mg, 0.34 mmol, 1.0 equiv). The crude was purified using flash chromatography (silica gel, 35:65 petrol/EtOAc) to afford the product as a brown oil (36 mg, 45% yield). Data matched the literature values.[7]

IR: 727 (m), 1026 (s), 1422 (s), 1600 (s), 2970 (w), 3394 (w, b) cm^{-1}.

^1H NMR ($CDCl_3$, 400 MHz): δ 1.55-1.78 (2H, m, CH_2), 1.80-1.90 (1H, m, CH_2), 2.06-2.18 (1H, m, CH_2), 3.45-3.60 (2H, m, CH_2), 3.65-3.83 (5H, m, CH_2OH+OMe), 4.32-4.41 (1H, m, CH), 4.98 (1H, br s, OH), 6.88 (2H, d, *J* = 8.5 Hz, Ar), 7.47 (2H, d, *J* = 8.5 Hz, Ar).

^{13}C NMR ($CDCl_3$, 100 MHz): δ 25.2, 28.5, 51.4, 55.4, 61.5, 67.2, 113.6, 128.7, 129.2, 161.1, 172.0.

HRMS (ESI-TOF) m/z: $[M+H]^+$ Cald for $C_{13}H_{18}NO_3^+$ 236.1281; Found 236.1290.

Synthesis of (2-(hydroxymethyl)pyrrolidin-1-yl)(2-methylphenyl)methanone (7f)

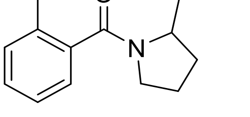

Prepared according to the general procedure for the cyclization reaction using *N*-(pent-4-en-1-yl)[1,1'-biphenyl]-4-carboxamide (**3f**, 72 mg, 0.35 mmol, 1.0 equiv). The crude was purified using flash chromatography (silica gel, 35:65 petrol/EtOAc) to afford the product as a brown oil (24 mg, 32% yield). Data matched the literature values.[17]

IR: 728 (s), 1030 (m), 1421 (s), 1595 (s), 2980 (m), 3367 (w, b) cm^{-1}.

^{1}H NMR ($CDCl_3$, 400 MHz): δ 1.58-1.90 (3H, m, CH_2), 2.11-2.21 (1H, m, CH_2), 2.32 (3H, s, Me), 3.13-3.26 (2H, m, CH_2), 3.70-3.81 (2H, m, CH_2OH), 4.34-4.42 (1H, m, CH), 5.11 (1H, br s, OH), 7.16-7.30 (4H, m, Ar).
^{13}C NMR ($CDCl_3$, 100 MHz): δ 18.9, 24.7, 28.7, 50.0, 61.2, 67.4, 125.4, 126.1, 129.2, 130.6, 133.6, 137.3, 172.5.
HRMS (ESI-TOF) m/z: $[M+H]^+$ Cald for $C_{13}H_{18}NO_2^+$ 220.1332; Found 220.1331.

Synthesis of (2-fluorophenyl)(2-(hydroxymethyl)pyrrolidin-1-yl)methanone (7g)

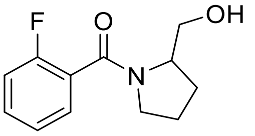

Prepared according to the general procedure for the cyclization reaction using 2-fluoro-*N*-(pent-4-en-1-yl)benzamide (**3g**, 83 mg, 0.40 mmol, 1.0 equiv). The crude was purified using flash chromatography (silica gel, 40:60 petrol/EtOAc) to afford the product as a brown oil (37 mg, 42% yield).
IR: 754 (m), 1050 (m), 1425 (s), 1608 (s), 2958 (w), 3382 (w, b) cm^{-1}.
^{1}H NMR ($CDCl_3$, 400 MHz): δ 1.62-1.81 (2H, m, CH_2), 1.82-1.91 (1H, m, CH_2), 2.11-2.20 (1H, m, CH_2), 3.31-3.42 (2H, m, CH_2), 3.70-3.81 (2H, m, CH_2OH), 4.30-4.39 (1H, m, CH), 4.76 (1H, br s, OH), 7.09 (1H, t, J = 9.2 Hz, Ar), 7.19 (1H, t, J = 7.4 Hz, Ar), 7.35-7.43 (2H, m, Ar).
^{13}C NMR ($CDCl_3$, 100 MHz): δ 24.6, 28.6, 49.5 (d, J = 3.7 Hz), 61.6, 66.7, 116.1 (d, J = 21.4 Hz), 124.7 (d, J = 3.4 Hz), 125.2 (d, J = 17.4 Hz), 128.8 (d, J = 3.6 Hz), 131.7 (d, J = 8.1 Hz), 158.2 (d, J = 248 Hz), 167.6.
^{19}F ($CDCl_3$, 376 MHz): -114.9.
HRMS (ESI-TOF) m/z: $[M+H]^+$ Cald for $C_{12}H_{15}FNO_2^+$ 224.1081; Found 224.1090.

Synthesis of (2-(hydroxymethyl)pyrrolidin-1-yl)(2-methoxyphenyl)methanone (7h)

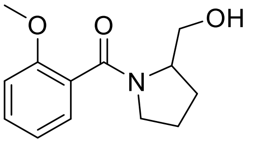

Prepared according to the general procedure for the cyclization reaction using 2-methoxy-*N*-(pent-4-en-1-yl)benzamide (**3h**, 68 mg, 0.31 mmol, 1.0 equiv). The crude was purified using flash chromatography (silica gel, 35:65 petrol/EtOAc) to afford the product as a brown oil (39 mg, 53% yield).
IR: 726 (s), 1021 (m), 1437 (s), 1597 (s), 2979 (m), 3398 (w, b) cm^{-1}.
^{1}H NMR ($CDCl_3$, 400 MHz): δ 1.59-1.87 (3H, m, CH_2), 2.09-2.19 (1H, m, CH_2), 3.19-3.31 (2H, m, CH_2), 3.65-3.73 (1H, m, CH_2OH), 3.77-3.85 (4H, m, CH_2OH+OMe), 4.28-4.37 (1H, m, CH), 4.97 (1H, br s, OH), 6.91 (1H, d, J = 8.4 Hz, Ar), 6.97 (1H, t, J = 7.4 Hz, Ar), 7.25 (1H, t, J = 7.7 Hz, Ar), 7.34 (1H, t, J = 7.9 Hz, Ar).
^{13}C NMR ($CDCl_3$, 100 MHz): δ 24.5, 28.7, 49.4, 55.8, 61.2, 66.8, 111.2, 121.0, 126.8, 127.7, 130.8, 155.1.
HRMS (ESI-TOF) m/z: $[M+H]^+$ Cald for $C_{13}H_{18}NO_3^+$ 236.1281; Found 236.1292.

Synthesis of 1-(2-(hydroxymethyl)pyrrolidin-1-yl)-2-phenylethan-1-one (7k)

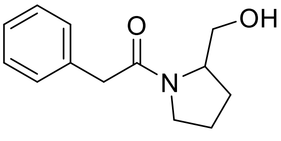

Prepared according to the general procedure for the cyclization reaction using *N*-(pent-4-en-1-yl)(phenyl)acetamide (**3k**, 83 mg, 0.41 mmol, 1.0 equiv). The crude was purified using flash chromatography (silica gel, 35:65 petrol/EtOAc) to afford the product as a brown oil (19 mg, 22% yield).

IR: 720 (m), 1029 (m), 1427 (m), 1614 (m), 2979 (w), 3362 (w, b) cm^{-1}.

^{1}H NMR ($CDCl_3$, 400 MHz): δ 1.52-1.62 (1H, m, CH_2), 1.76-1.96 (3H, m, CH_2), 1.97-2.08 (1H, m, CH_2), 3.39-3.47 (1H, m, CH_2), 3.52-3.68 (3H, m, CH_2+CH_2OH), 3.69 (2H, s, ArCH_2), 4.19-4.27 (1H, m, CH), 5.03 (1H, br s, OH), 7.23-7.28 (3H, m, Ar), 7.30-7.36 (2H, m, Ar).

^{13}C NMR ($CDCl_3$, 100 MHz): δ 24.5, 28.4, 42.5, 48.5, 61.6, 67.4, 127.1, 128.8, 129.0, 134.4, 172.5.

HRMS (ESI-TOF) m/z: $[M+H]^+$ Cald for $C_{13}H_{18}NO_2^+$ 220.1332; Found 220.1341.

Synthesis of cyclopropyl(2-(hydroxymethyl)pyrrolidin-1-yl)methanone (7m)

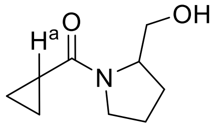

Prepared according to the general procedure for the cyclization reaction using *N*-(pent-4-en-1-yl)cyclopropanecarboxamide (**3m**, 71 mg, 0.47 mmol, 1.0 equiv). The crude was purified using flash chromatography (silica gel, 40:60 petrol/EtOAc) to afford the product as a brown oil (40 mg, 50% yield).

IR: 739 (m), 1030 (m), 1438 (s), 1602 (s), 2950 (w), 3374 (w, b) cm^{-1}.

^{1}H NMR ($CDCl_3$, 400 MHz): δ 0.78-0.85 (2H, m, cycloprop), 0.98-1.08 (2H, m, cycloprop), 1.58-1.68 (2H, m, CH_2), 1.86-2.08 (3H, m, CH_2+H^a), 3.52-3.79 (4H, m, CH_2+CH_2OH), 4.18-4.27 (1H, m, CH), 5.21 (1H, dd, J = 7.4, 1.6 Hz, OH).

^{13}C NMR ($CDCl_3$, 100 MHz): δ 8.0, 8.5, 12.9, 24.5, 28.5, 48.3, 61.6, 68.0, 175.2.

HRMS (ESI-TOF) m/z: $[M+H]^+$ Cald for $C_9H_{16}NO_2^+$ 170.1176; Found 170.1183.

Synthesis of cyclohexyl(2-(hydroxymethyl)pyrrolidin-1-yl)methanone (7n)

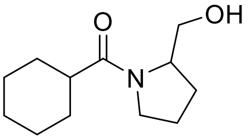

Prepared according to the general procedure for the cyclization reaction using *N*-(pent-4-en-1-yl)cyclohexanecarboxamide (**3n**, 76 mg, 0.39 mmol, 1.0 equiv). The crude was purified using flash chromatography (silica gel, 50:50 petrol/EtOAc) to afford the product as a yellow oil (22 mg, 26% yield).

IR: 1050 (m), 1435 (m), 1606 (m), 2924 (m), 3363 (w, b) cm^{-1}.

^{1}H NMR ($CDCl_3$, 400 MHz): δ 1.17-1.31 (4H, m, cyclohex), 1.43-1.60 (3H, m, cyclohex+CH_2), 1.63-1.70 (1H, m, CH_2), 1.73-1.82 (2H, m, cyclohex+CH_2), 1.82-2.07 (4H,

m, cyclohex), 2.34 (1H, tt, *J* = 3.1, 11.7 Hz, cyclohexCH), 3.43-3.64 (4H, m, CH_2+CH_2OH), 4.15-4.23 (1H, m, CH), 5.24 (1H, br s, OH).
^{13}C NMR ($CDCl_3$, 100 MHz): δ 24.6, 25.81, 25.83, 25.9, 28.3, 28.6, 29.4, 43.2, 47.8, 61.2, 67.8, 177.8.
HRMS (ESI-TOF) m/z: $[M+H]^+$ Cald for $C_{12}H_{22}NO_2^+$ 212.1645; Found 212.1653.

Synthesis of furan-2-yl(2-(hydroxymethyl)pyrrolidin-1-yl)methanone (7o)

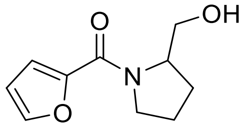

Prepared according to the general procedure for the cyclization reaction using *N*-(pent-4-en-1-yl)furan-2-carboxamide (**3o**, 73 mg, 0.41 mmol, 1.0 equiv). The crude was purified using flash chromatography (silica gel, 35:65 petrol/EtOAc) to afford the product as a brown oil (50 mg, 63% yield).
IR: 750 (s), 1027 (s), 1422 (s), 1594 (s), 2947 (w), 3351 (w, b) cm^{-1}.
^1H NMR ($CDCl_3$, 400 MHz): δ 1.61-1.72 (1H, m, CH_2), 1.81-1.92 (1H, m, CH_2), 1.92-2.09 (2H, m, CH_2), 3.64-3.73 (2H, m, CH_2OH), 3.77-3.85 (1H, m, CH_2), 3.91-4.00 (1H, m, CH_2), 4.36-4.44 (1H, m, CH), 4.78 (1H, br s, OH), 6.47 (1H, d, *J* = 1.6 Hz, furan), 7.07 (1H, d, *J* = 3.0 Hz, furan), 7.50 (1H, s, furan).
^{13}C NMR ($CDCl_3$, 100 MHz): δ 25.0, 27.8, 49.2, 62.3, 66.9, 111.6, 117.0, 144.7, 148.2, 160.4.
HRMS (ESI-TOF) m/z: $[M+H]^+$ Cald for $C_{10}H_{14}NO_3^+$ 196.0968; Found 196.0966.

Synthesis of (2-(hydroxymethyl)-4,4-dimethylpyrrolidin-1-yl)(phenyl)methanone (7p)

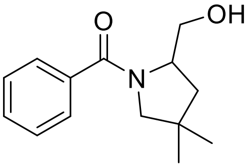

Prepared according to the general procedure for the cyclization reaction using *N*-(2,2-dimethylpent-4-en-1-yl)benzamide (**3p**, 198 mg, 0.91 mmol, 1.0 equiv). The crude was purified using flash chromatography (silica gel, 40:60 petrol/EtOAc) to afford the product as a brown oil (20 mg, 10% yield).
IR: 698 (m), 1028 (m), 1426 (m), 1600 (m), 2959 (w), 3347 (w, b) cm^{-1}.
^1H NMR ($CDCl_3$, 400 MHz): δ 0.96 (3H, s, Me), 1.05 (3H, s, Me), 1.43 (1H, t, *J* = 11.6 Hz, CH_2), 1.89 (1H, ddd, *J* = 12.6, 7.4, 1.8Hz, CH_2), 3.18 (1H, dd, *J* = 10.7, 1.5 Hz, CH_2), 3.27 (1H, d, *J* = 10.7 Hz, CH_2), 3.69-3.81 (2H, m, CH_2OH), 4.45-4.54 (1H, m, CH), 4.98 (1H, br s, OH), 7.38-7.44 (3H, m, Ar), 7.46-7.50 (2H, m, Ar).
^{13}C NMR ($CDCl_3$, 100 MHz): δ 25.4, 25.6, 37.7, 42.2, 61.3, 63.4, 67.5, 127.2, 128.5, 130.3, 136.6, 172.7.
HRMS (ESI-TOF) m/z: $[M+H]^+$ Cald for $C_{14}H_{20}NO_2^+$ 234.1489; Found 234.1499.

Synthesis of (2-(1-hydroxyethyl)pyrrolidin-1-yl)(phenyl)methanone (7q)

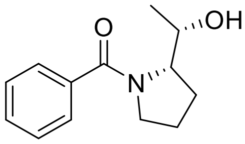

Prepared according to the general procedure for the cyclization reaction using *N*-((4*Z*)-hex-4-en-1-yl)benzamide (**3q**, 72 mg, 0.35 mmol, 1.0 equiv). The crude was purified using flash chromatography (silica gel, 40:60 petrol/EtOAc) to afford the product as a brown oil (26 mg, 34% yield). Only one diastereomer observed.
IR: 699 (m), 1026 (m), 1420 (s), 1599 (s), 2970 (w), 3381 (w, b) cm^{-1}.
^1H NMR ($CDCl_3$, 400 MHz): δ 1.17 (3H, d, *J* = 6.3 Hz, Me), 1.61-1.77 (2H, m, CH_2), 1.81-1.90 (1H, m, CH_2), 2.09-2.20 (1H, m, CH_2), 3.38-3.47 (1H, m, CH_2), 3.49-3.57 (1H, m, CH_2), 4.06 (1H, d, *J* = 5.7 Hz, CH), 4.39 (1H, t, *J* = 7.8 Hz, CHOH), 4.79 (1H, br s, OH), 7.37-7.43 (3H, H, Ar), 7.50 (2H, *J* = 6.6 Hz, Ar).
^{13}C NMR ($CDCl_3$, 100 MHz): δ 17.5, 25.2, 28.0, 52.1, 64.8, 69.3, 127.2, 128.4, 130.3, 136.8, 171.9.
HRMS (ESI-TOF) m/z: $[M+H]^+$ Cald for $C_{13}H_{18}NO_2^+$ 220.1332; Found 220.1340.

Synthesis of *N*-(6-(acetylamino)-5-fluoro-6-methylheptan-2-yl)benzamide (15)

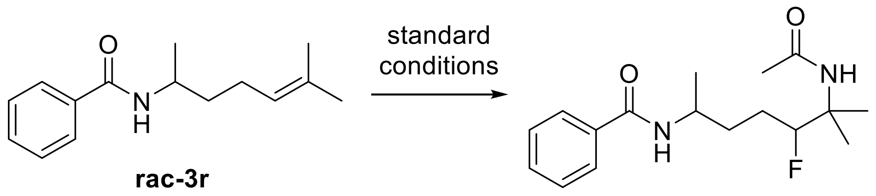

15 (56%, 1:1 d.r.)
(one regioisomer)

Prepared according to the general procedure for the cyclization reaction using *N*-(6-methylhept-5-en-2-yl)benzamide (**3r**, 75 mg, 0.32 mmol, 1.0 equiv). The crude was purified using flash chromatography (silica gel, 35:65 petrol/EtOAc) to afford the product as a brown oil (56 mg, 56% yield). The product is a 1:1 mixture of diastereomers.
IR: 1028 (m), 1451 (m), 1634 (m), 2972 (w), 3305 (w, b) cm^{-1}.
^1H NMR ($CDCl_3$, 400 MHz): δ 1.22 (3H, d, *J* = 5.6 Hz, Me), 1.22 (3H, d, *J* = 5.6 Hz, Me), 4.27 (2x3H, d, *J* = 5.6 Hz, Me), 1.29 (3H, s, Me), 1.32 (3H, s, Me), 1.50-1.73 (2x4H, m, CH_2), 1.83 (3H, s, Me), 1.86 (3H, s, Me), 4.10-4.30 (2x1H, m, C*H*N), 4.68-4.77 (1H, m, C*H*F), 4.80-4.89 (1H, m, C*H*F), 5.76 (1H, s, NH), 5.91 (1H, s, NH), 6.33-6.41 (2x1H, m, NH), 7.34-7.41 (2x2H, m, Ar), 7.41-7.50 (2x1H, m, Ar), 7.70-7.77 (2x2H, m, Ar).
^{13}C NMR ($CDCl_3$, 100 MHz): δ 21.4, 21.6, 22.0 (d, *J* = 3.3 Hz), 22.5 (d, *J* = 3.0 Hz), 22.6 (d, *J* = 3.5 Hz), 22.8 (d, *J* = 4.1 Hz), 24.37, 24.44, 26.1 (d, *J* = 21 Hz), 26.5 (d, *J* = 21 Hz), 33.6 (d, *J* = 1.7 Hz), 34.0 (d, *J* = 2.2 Hz), 45.1, 45.9, 56.2 (d, *J* = 21 Hz), 56.3 (d, *J* = 20 Hz), 96.0 (d, *J* = 175 Hz), 96.7 (d, *J* = 175 Hz), 126.9 (2x2C), 128.6, 128.7, 131.5, 131.6, 134.7, 134.9, 167.2, 167.4, 170.07, 170.12.
^{19}F ($CDCl_3$, 376 MHz): -193.8, -193.2.
HRMS (ESI-TOF) m/z: $[M+H]^+$ Cald for $C_{17}H_{26}FN_2O_2^+$ 309.1973; Found 309.1968.

Cartesian coordinates and energies of calculated structures

8

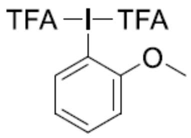

C -0.000004 1.568477 -0.539663

C -0.000022 2.465623 0.543921

C 0.000005 2.000779 -1.862347

C -0.000031 3.838184 0.254964

C -0.000003 3.370447 -2.132954

H 0.000020 1.281853 -2.674148

C -0.000021 4.275885 -1.071080

H -0.000043 4.562876 1.059898

H 0.000003 3.718646 -3.160003

H -0.000027 5.343182 -1.270565

O 2.201147 -0.222484 -0.184127

C 2.803245 -1.359379 0.020475

C 4.352072 -1.199825 0.007068

O 2.277486 -2.443260 0.203918

F 4.747053 -0.324951 0.958231

F 4.769709 -0.731585 -1.189615

F 4.963552 -2.368315 0.235023

I 0.000007 -0.498674 -0.137704

O -2.201133 -0.222507 -0.184159

C -4.352055 -1.199853 0.007054

F -4.963531 -2.368358 0.234943

F -4.769695 -0.731546 -1.189600

F -4.747037 -0.325035 0.958269

C -2.803227 -1.359400 0.020462

O -2.277464 -2.443276 0.203927

O -0.000032 1.938191 1.788154

C -0.000063 2.812646 2.919788

H -0.898601 3.439108 2.929620

H -0.000078 2.158193 3.790738

H 0.898465 3.439122 2.929658

Zero-point correction = 0.178345 (Hartree/Particle)

Thermal correction to Energy = 0.202063

Thermal correction to Enthalpy = 0.203007

Thermal correction to Gibbs Free Energy = 0.117439

SCF Done: E(RB3LYP) = -1409.97140056 Hartree

3a

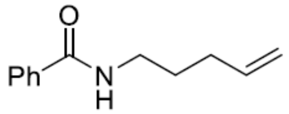

C 0.49522 -0.92112 0.09276

C -1.6235 -1.40535 -1.1189

C -5.31559 -0.12613 -1.2968

C -2.81342 -0.43586 -1.04999

C -4.15804 -1.07594 -1.44093

H -5.48708 0.26706 -0.29266

H -1.6813 -2.14181 -0.31356

H -2.60338 0.40868 -1.71902

H -1.64432 -1.94808 -2.07194

H -2.88348 -0.01498 -0.03643

H -4.32787 -1.9537 -0.80223

H -4.10375 -1.44659 -2.47157

N -0.32717 -0.74602 -0.98744

H 0.03694 -0.25644 -1.79108

O 0.62702 -2.01025 0.65197

C -6.12421 0.26498 -2.28661

H -6.94444 0.95584 -2.11369

H -5.99542 -0.09876 -3.30391

C 1.24665 0.29615 0.56691

C 2.41155 0.08095 1.31766

C 0.82919 1.61283 0.31784

C 3.15845 1.16022 1.78893

H 2.71029 -0.94142 1.52316

C 1.57274 2.69365 0.79757

H -0.09441 1.81034 -0.21918

C 2.74222 2.46972 1.52849

H 4.06308 0.98079 2.3626

H 1.2333 3.70781 0.60814

H 3.32091 3.31055 1.89987

Zero-point correction = 0.245807 (Hartree/Particle)

Thermal correction to Energy = 0.259812

Thermal correction to Enthalpy = 0.260756

Thermal correction to Gibbs Free Energy = 0.202185

SCF(Done): E(RB3LYP): -596.331553629 Hartree

TS[8-9]

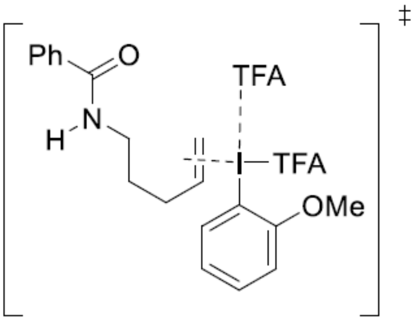

C -1.71301 2.06039 1.60314

H -2.61314 1.4932 1.367

C -1.49709 3.33669 0.83413

H -2.29361 4.05089 1.09662

H -0.55125 3.77893 1.15807
C -0.88434 1.58866 2.54814
H 0.02313 2.12049 2.82129
H -1.11105 0.67268 3.087
C -0.19931 -1.50337 0.82227
C 0.05606 -2.31956 1.94932
C -2.30384 -2.6202 0.43144
C -0.88322 -3.30379 2.28959
C -2.04775 -3.4442 1.53133
H -3.21571 -2.73127 -0.14483
H -0.71406 -3.95132 3.14141
H -2.76657 -4.20831 1.81295
O 2.07699 -1.48591 -1.15492
C 3.13476 -2.17159 -0.75138
C 3.60463 -3.11411 -1.9073
O 3.68289 -2.16477 0.32215
F 2.71489 -4.12325 -2.05275
F 4.79778 -3.64796 -1.61967
F 3.70019 -2.46352 -3.0781
C -1.37495 -1.643 0.07585
H -1.01199 2.19946 -0.96419
H -0.81815 3.93109 -1.13161
C -1.46802 3.15824 -0.7037
N -3.86618 2.35933 -0.97986
H -4.72522 2.75806 -0.63197
C -3.87408 1.04191 -1.32781
O -2.93813 0.52078 -1.94316
C -5.08536 0.24474 -0.92387
C -5.86984 0.55446 0.19783
C -5.42083 -0.86922 -1.70794
C -6.97748 -0.23226 0.52327
H -5.60556 1.38766 0.84324
C -6.53379 -1.64698 -1.38866

H -4.79994 -1.10592 -2.56587

C -7.31502 -1.33021 -0.27224

H -7.57091 0.00899 1.4002

H -6.79354 -2.49869 -2.01062

H -8.17965 -1.93788 -0.02175

H -2.65722 3.13697 -2.49957

H -3.21841 4.2932 -1.28183

C -2.81651 3.28483 -1.42522

I 1.24508 -0.08035 0.22003

O 1.67682 2.44417 0.86679

C 2.66576 2.53947 0.08416

C 3.3534 3.93774 0.05727

F 4.44187 3.96411 -0.72838

F 2.48834 4.87673 -0.40749

F 3.73126 4.31716 1.3002

O 3.11874 1.64381 -0.65937

H -1.57678 -0.9919 -0.77043

O 1.19822 -2.08625 2.62904

C 1.58117 -2.95769 3.69345

H 2.55568 -2.59929 4.02283

H 1.66956 -3.99056 3.33968

H 0.8669 -2.90297 4.52284

Zero-point correction = 0.424395 (Hartree/Particle)

Thermal correction to Energy = 0.463471

Thermal correction to Enthalpy = 0.464416

Thermal correction to Gibbs Free Energy = 0.338895

SCF Done: E(RB3LYP) = -2006.27254238 Hartree

Imaginary frequency= -51.06 cm^{-1}

9

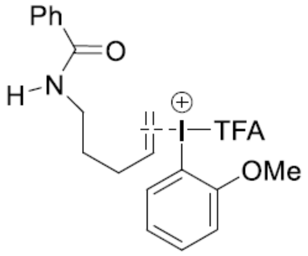

C 1.29369 -1.33116 0.10995

H 1.59497 -0.65708 -0.68765

C 1.51879 -2.79165 -0.11672

H 1.20066 -3.36601 0.76147

C 0.7389 -0.78786 1.24173

H 0.52486 -1.40642 2.11015

H 0.74647 0.28635 1.39205

C -1.44919 1.2283 -0.42502

C -1.59216 2.18695 0.6014

C -1.15046 2.91892 -2.1015

C -1.5211 3.5385 0.22811

C -1.30552 3.8889 -1.10467

H -0.98295 3.20606 -3.1335

H -1.63036 4.31173 0.9788

H -1.2544 4.941 -1.36683

O -3.72194 -0.75241 -0.72123

C -4.6835 -0.57585 0.17177

C -6.07597 -0.60382 -0.54039

O -4.56953 -0.42603 1.36452

F -6.15725 0.41159 -1.42291

F -7.0578 -0.47497 0.35252

F -6.23857 -1.76326 -1.20227

C -1.22334 1.57084 -1.76055

I -1.69964 -0.7988 0.07391

H -1.11791 0.80276 -2.51833

O -1.76478 1.73333 1.85495

C -2.09436 2.65777 2.90927

H -2.27144 2.0377 3.78628

H -3.00226 3.21396 2.65957

H -1.2612 3.34173 3.09711

C 2.98844 -3.12614 -0.47829

H 3.01867 -4.16735 -0.81384

H 3.29917 -2.51641 -1.33203

C 3.98889 -2.91385 0.6913

H 3.47193 -2.46865 1.54874

H 4.4013 -3.8697 1.0208

N 5.11742 -2.05711 0.3419

H 6.05013 -2.42901 0.4351

C 4.94632 -0.71494 0.18607

O 3.80659 -0.22304 0.24371

C 6.15503 0.12735 -0.07123

C 7.39359 -0.40268 -0.46977

C 6.0261 1.51535 0.09402

C 8.48359 0.44037 -0.68654

H 7.51934 -1.46631 -0.65245

C 7.11804 2.35555 -0.11691

H 5.06407 1.91561 0.39403

C 8.34975 1.82 -0.50528

H 9.43347 0.02084 -1.00238

H 7.00996 3.42682 0.02194

H 9.20028 2.47391 -0.6713

H 0.89094 -3.11547 -0.96057

Zero-point correction = 0.398225 (Hartree/Particle)

Thermal correction to Energy = 0.429813

Thermal correction to Enthalpy = 0.430758

Thermal correction to Gibbs Free Energy = 0.325847

SCF Done: E(RB3LYP) = -1479.83351702 Hartree

TS[9-10]

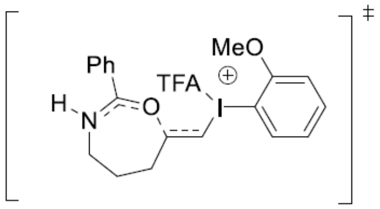

C 4.85643 0.08058 -0.5201

C 3.86485 1.58585 -2.25404

C 1.6285 0.18542 -0.63183

C 2.93605 2.32447 -1.2681

C 1.57919 1.63735 -0.98225

H 1.92727 -0.50581 -1.41387

H 3.29662 0.89371 -2.89038

H 2.7078 3.3151 -1.6734

H 4.33072 2.3086 -2.92731

H 3.46231 2.48555 -0.32407

H 0.96436 1.71552 -1.89279

H 1.0598 2.19399 -0.19683

N 4.97214 0.87516 -1.60546

H 5.872 0.90961 -2.06267

O 3.74958 -0.14682 0.0245

C 1.00725 -0.35611 0.50093

H 1.23471 -1.38972 0.74891

H 0.79963 0.29092 1.34915

I -1.32184 -0.76923 -0.31962

C -1.90739 1.21908 0.0879

C -2.30322 2.03506 -0.9718

C -1.99404 1.61647 1.43573

C -2.81115 3.30238 -0.68691

C -2.51443 2.89382 1.69639

C -2.91567 3.71754 0.64427

H -3.13639 3.94676 -1.49618

H -2.60934 3.23863 2.71905

H -3.31982 4.69892 0.87186

C 6.10203 -0.52516 0.03396

C 5.98912 -1.71713 0.76664

C 7.36647 0.06357 -0.13976

C 7.12674 -2.32519 1.29443

H 5.00868 -2.15725 0.91042

C 8.50099 -0.54373 0.39816

H 7.47429 1.01406 -0.65483

C 8.3837 -1.74135 1.10953

H 7.03411 -3.25269 1.85055

H 9.47289 -0.07781 0.27066

H 9.26862 -2.21348 1.52486

O -3.42417 -1.19891 -0.97407

C -4.3029 -1.23182 -0.00278

O -4.11492 -1.03411 1.18076

C -5.72916 -1.54716 -0.55862

F -6.61576 -1.65298 0.43528

F -6.12799 -0.55339 -1.38219

F -5.7254 -2.69945 -1.25471

H -2.25009 1.68052 -1.99538

O -1.54971 0.75419 2.37115

C -1.92673 0.95367 3.74692

H -3.01376 1.03154 3.83333

H -1.57731 0.06584 4.27165

H -1.43863 1.84236 4.15878

Zero-point correction = 0.398904 (Hartree/Particle)

Thermal correction to Energy = 0.429287

Thermal correction to Enthalpy = 0.430231

Thermal correction to Gibbs Free Energy = 0.329718

SCF Done: E(RB3LYP) = -1479.82962603 Hartree

Imaginary frequency= -64.42 cm^{-1}

TS[9-11]

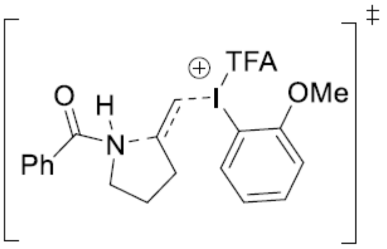

C -1.74335 -1.43442 -0.57096

H -1.99244 -1.64832 0.46795

C -1.39823 -2.63693 -1.3983

H -1.08385 -2.33162 -2.40323

H -0.54063 -3.13737 -0.92402

C -1.20576 -0.1412 -0.83862

H -1.03895 0.12522 -1.88078

H -1.54849 0.6758 -0.20839

I 1.14792 -0.29757 -0.06924

C 1.20577 1.81398 0.02362

C 1.42985 2.53012 -1.16732

C 1.19662 3.82749 1.33494

C 1.53616 3.92586 -1.07392

C 1.42076 4.55784 0.16549

H 1.11957 4.32642 2.29462

H 1.71341 4.51724 -1.96394

H 1.51185 5.63845 0.21309

O 3.33659 -0.13121 0.57107

C 4.04054 -0.21322 -0.51505

C 5.57115 -0.08709 -0.24215

O 3.61036 -0.36484 -1.65132

F 6.27515 -0.2182 -1.37001

F 5.97217 -1.03737 0.62596

F 5.84391 1.1203 0.29507

C 1.09082 2.43807 1.26477

H 0.94387 1.85356 2.16638

H -2.76151 -4.05207 -0.4684

H -2.40812 -4.41617 -2.16151

C -2.58839 -3.60489 -1.45133

N -3.7922 -1.50341 -1.0992

H -3.93148 -0.68306 -1.68051

C -4.53467 -1.46431 0.1349

O -4.70633 -2.51072 0.73196

C -4.98434 -0.1386 0.62896

C -4.85003 1.06305 -0.09258

C -5.60008 -0.11411 1.8948

C -5.31859 2.2616 0.44412

H -4.39678 1.09318 -1.07959

C -6.06513 1.08499 2.42699

H -5.7076 -1.04509 2.44044

C -5.92541 2.27434 1.70355

H -5.21921 3.18162 -0.12279

H -6.54051 1.09322 3.40249

H -6.29401 3.20815 2.11666

H -4.74334 -3.31136 -1.66072

H -3.76887 -2.51971 -2.92378

C -3.81113 -2.77934 -1.86254

O 1.51577 1.82256 -2.31738

C 1.9362 2.48923 -3.51908

H 2.91083 2.96496 -3.37476

H 2.01761 1.70534 -4.27087

H 1.19416 3.22937 -3.83553

Zero-point correction = 0.399081 (Hartree/Particle)

Thermal correction to Energy = 0.429286

Thermal correction to Enthalpy = 0.430230

Thermal correction to Gibbs Free Energy = 0.329111

SCF Done: E(RB3LYP) = -1479.82658287 Hartree

Imaginary frequency= -170.91 cm^{-1}

10

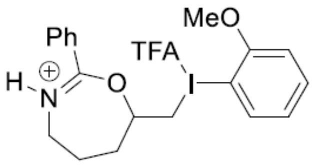

C 5.02142 0.16231 -0.49328

C 4.03556 1.6686 -2.23236

C 1.81006 0.26428 -0.60402

C 3.10888 2.40807 -1.24582

C 1.75659 1.71746 -0.94958

H 2.10695 -0.42351 -1.38991

H 3.46517 0.98294 -2.87379

H 2.8756 3.39572 -1.65551

H 4.50759 2.39206 -2.90065

H 3.63956 2.57642 -0.30542

H 1.13317 1.79598 -1.85441

H 1.24213 2.27094 -0.1587

N 5.13743 0.94852 -1.58411

H 6.03683 0.97501 -2.04298

O 3.91555 -0.05724 0.05825

C 1.17994 -0.28214 0.5233

H 1.40784 -1.31643 0.76826

H 0.9749 0.36166 1.37468

I -1.14666 -0.69019 -0.29346

C -1.73246 1.29836 0.11384

C -2.12902 2.11389 -0.94588

C -1.81967 1.69582 1.46153

C -2.63785 3.38094 -0.6612

C -2.34098 2.97282 1.72207

C -2.74272 3.79622 0.66989

H -2.96368 4.02489 -1.47057

H -2.43632 3.31758 2.74472

H -3.14767 4.77729 0.89737

C 6.26656 -0.44469 0.06037

C 6.15389 -1.63604 0.79412

C 7.53116 0.14346 -0.11515

C 7.29197 -2.24427 1.32081

H 5.17353 -2.0758 0.93961

C 8.66601 -0.46396 0.4219

H 7.63854 1.09369 -0.63079

C 8.54899 -1.66112 1.1341

H 7.19958 -3.17139 1.8776

H 9.63795 0.00156 0.29314

H 9.43415 -2.13341 1.54874

O -3.25185 -1.1172 -0.94927

C -4.13004 -1.15076 0.02197

O -3.94202 -0.95354 1.20579

C -5.55651 -1.46656 -0.53303

F -6.44294 -1.57247 0.46115

F -5.95624 -0.47322 -1.3568

F -5.55293 -2.61911 -1.22889

H -2.07606 1.75907 -1.96938

O -1.37501 0.83385 2.3972

C -1.75405 1.03241 3.7725

H -2.84128 1.1092 3.85748

H -1.40452 0.14471 4.29738

H -1.26736 1.9214 4.18543

Zero-point correction = 0.402266 (Hartree/Particle)

Thermal correction to Energy = 0.432218

Thermal correction to Enthalpy = 0.433162

Thermal correction to Gibbs Free Energy = 0.333758

SCF Done: E(RB3LYP) = -1479.84721816 Hartree

11

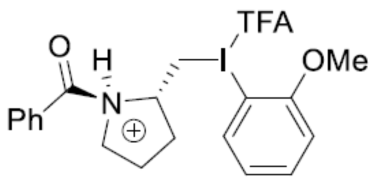

C 2.08785 -1.76064 0.00492

H 2.03411 -1.21267 -0.93854

C 1.90146 -3.26004 -0.21203

H 1.706 -3.75745 0.74613

H 1.03645 -3.43538 -0.8576

C 1.26336 -1.11347 1.07524

H 1.2659 -1.66387 2.0198

H 1.46575 -0.05458 1.23391

I -1.06553 -1.05659 0.56201

C -0.74639 0.50729 -0.83302

C -0.44803 1.78326 -0.32635

C -0.83733 1.32538 -3.08821

C -0.35836 2.83999 -1.24468

C -0.55211 2.60535 -2.60692

H -0.99947 1.15411 -4.14675

H -0.14688 3.84309 -0.89337

H -0.4853 3.43921 -3.29872

O -3.30334 -0.92456 -0.01655

C -3.90466 0.14412 0.40552

C -5.41217 0.13801 0.0078

O -3.42789 1.08863 1.01764

F -6.05745 1.20503 0.49719

F -6.02768 -0.97266 0.46448

F -5.53111 0.15806 -1.34091

C -0.94427 0.26152 -2.18956

H -1.21471 -0.72861 -2.53977

H 3.29188 -3.44019 -1.87604

H 3.28662 -4.85691 -0.81343

C 3.20237 -3.76777 -0.83618

N 3.767 -1.70282 0.2986

H 3.88747 -1.46932 1.28429

C 4.45429 -0.66504 -0.57712

O 4.68423 -1.00276 -1.71177

C 4.73229 0.65474 0.00667

C 4.61294 0.9669 1.37563

C 5.14999 1.65436 -0.89867

C 4.89605 2.25505 1.82472

H 4.31593 0.22617 2.11296

C 5.42058 2.93975 -0.44427

H 5.248 1.40376 -1.94905

C 5.29226 3.24242 0.91655

H 4.81186 2.48777 2.88105

H 5.73418 3.70521 -1.14641

H 5.50848 4.24533 1.27149

H 5.25648 -3.03986 -0.47647

H 4.3978 -3.60869 0.98104

C 4.28667 -3.11195 0.01417

O -0.23773 1.8967 1.00517

C -0.40918 3.18257 1.62607

H -1.38047 3.60549 1.35627

H -0.37767 2.99275 2.69833

H 0.40219 3.86351 1.34814

Zero-point correction = 0.401673 (Hartree/Particle)

Thermal correction to Energy = 0.431988

Thermal correction to Enthalpy = 0.432932

Thermal correction to Gibbs Free Energy = 0.330865

SCF Done: E(RB3LYP) = -1479.83676517 Hartree

12

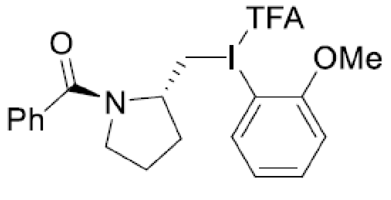

C -1.51763 -1.40908 0.44972

H -1.41329 -0.68666 1.26171

C -1.0716 -2.79757 0.9141

H -0.99126 -3.48418 0.06134

H -0.09173 -2.75074 1.39467

C -0.9324 -0.8748 -0.8352

H -1.00033 -1.59119 -1.66009

H -1.34784 0.08882 -1.13479

I 1.31732 -0.48546 -0.63028

C 0.94985 1.55649 -0.16531

C 1.16002 2.51112 -1.15588

C 0.42048 3.26386 1.44074

C 0.98738 3.86229 -0.8464

C 0.62086 4.22697 0.4494

H 0.14282 3.5732 2.44109

H 1.15774 4.61522 -1.6081

H 0.49481 5.27506 0.70215

O 3.55552 0.2749 -0.36008

C 4.29346 -0.76739 -0.51662

C 5.81388 -0.4681 -0.35428

O 3.91446 -1.91168 -0.76623

F 6.21474 0.45545 -1.25676

F 6.56119 -1.56694 -0.52503

F 6.06815 0.02224 0.88153

C 0.58777 1.90335 1.14627

H -2.06597 -2.73488 2.84459

H -2.17951 -4.31623 2.06374

C -2.17683 -3.23944 1.88019

N -3.07135 -1.60036 0.30611

C -3.86724 -0.33228 0.65313

O -3.52601 0.23761 1.65642

C -4.96159 0.03177 -0.25114

C -5.37026 -0.74295 -1.35698

C -5.64037 1.23472 0.04199

C -6.43121 -0.31905 -2.15238

H -4.89719 -1.68774 -1.61055

C -6.69539 1.65297 -0.75997

H -5.32447 1.82256 0.89669

C -7.09135 0.87848 -1.85714

H -6.74509 -0.92097 -2.99849

H -7.21155 2.57973 -0.53242

H -7.91692 1.20598 -2.48125

H -4.25292 -2.46784 1.88801

H -3.88617 -3.55908 0.53377

C -3.47578 -2.79544 1.19723

H 1.48064 2.21405 -2.14826

O 0.4083 0.89462 2.04277

C 0.33323 1.22328 3.43898

H 0.34481 0.26891 3.96489

H 1.20029 1.81777 3.74192

H -0.59413 1.75859 3.66763

Zero-point correction = 0.388844 (Hartree/Particle)

Thermal correction to Energy = 0.418807

Thermal correction to Enthalpy = 0.419751

Thermal correction to Gibbs Free Energy = 0.319675

SCF Done: E(RB3LYP) = -1479.49475645 Hartree

TS[12-13]

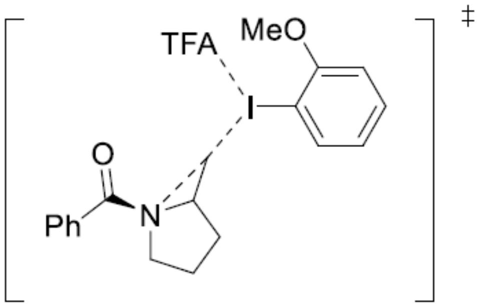

C 0.96983 -0.75281 -1.83137

H 0.97099 0.27727 -2.19043

C 0.9439 -1.87153 -2.88845

H 0.09767 -2.54331 -2.70963

H 0.83142 -1.46089 -3.89429

I -2.49507 -0.69937 -1.14688

C -3.05617 -1.21904 0.85261

C -3.82769 -2.36188 1.06882

C -3.04192 -0.71273 3.19286

C -4.20689 -2.67208 2.37885

C -3.81518 -1.84977 3.43892

H -2.73654 -0.06852 4.01198

H -4.81045 -3.55606 2.56319

H -4.11537 -2.09397 4.45356

O -0.42511 1.63143 0.75073

C -0.51481 2.38133 -0.26373

C -0.8976 3.85852 0.08623

O -0.37117 2.10508 -1.46748

F -0.89839 4.68458 -0.98068

F -0.0522 4.39661 1.00259

F -2.15073 3.9132 0.62642

C -2.65354 -0.37782 1.88988

H -2.04479 0.50362 1.69722

H 3.06244 -2.14909 -3.32578

H 2.23072 -3.6712 -2.97576

C 2.28739 -2.61271 -2.70653

N 2.13164 -1.03285 -0.94277

C 3.01582 0.04278 -0.58978

O 2.92682 1.11881 -1.14339

C 4.01237 -0.24116 0.48329

C 3.78397 -1.17939 1.502

C 5.18864 0.52537 0.49669

C 4.72683 -1.35442 2.51558

H 2.8589 -1.74618 1.52312

C 6.13507 0.33669 1.50181

H 5.33873 1.26761 -0.28027

C 5.90582 -0.60337 2.51215

H 4.53722 -2.06771 3.31192

H 7.0457 0.92783 1.504

H 6.63959 -0.74303 3.30057

H 3.6938 -2.46529 -1.00071

H 2.11091 -3.13474 -0.58584

C 2.62799 -2.40828 -1.22301

C 0.17319 -0.90353 -0.59432

H 0.04819 -0.01482 0.05128

H 0.01668 -1.90501 -0.2033

O -4.22749 -3.17368 0.09162

C -4.97656 -4.29598 0.42634

H -5.22806 -4.87523 -0.43744

H -5.87327 -3.97085 0.91122

H -4.40244 -4.89453 1.10237

Zero-point correction = 0.386878 (Hartree/Particle)

Thermal correction to Energy = 0.416760

Thermal correction to Enthalpy = 0.417704

Thermal correction to Gibbs Free Energy = 0.318013

SCF Done: E(RB3LYP) = -1479.46950749 Hartree

Imaginary frequency= -254.56 cm^{-1}

13

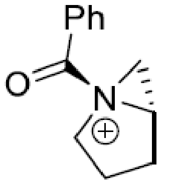

C 2.38567 0.15507 -1.06911

H 2.26113 1.04283 -1.70149

C 3.65486 -0.10833 -0.27013

H 4.09952 -1.06684 -0.55991

H 4.39602 0.67151 -0.45635

H 3.22446 0.89415 1.61387

H 3.8228 -0.75642 1.84435

C 3.19905 -0.12178 1.20868

N 1.20912 -0.10535 -0.14729

C 0.06022 0.89351 -0.03776

O 0.37453 2.04542 0.09112

C -1.29718 0.32219 -0.00944

C -1.53903 -1.05935 0.0656

C -2.378 1.22121 -0.03022

C -2.84815 -1.53585 0.12789

H -0.71807 -1.7674 0.07205

C -3.68137 0.73894 0.02249

H -2.17725 2.2838 -0.10955

C -3.91862 -0.63825 0.10227

H -3.03271 -2.60392 0.18299

H -4.51442 1.434 -0.01083

H -4.93763 -1.01187 0.13803

H 1.1242 -0.21088 1.97194

H 1.66294 -1.70282 1.17394

C 1.74561 -0.6125 1.17014

C 1.43871 -0.946 -1.37228

H 0.71321 -0.70303 -2.14942

H 1.68514 -1.98212 -1.15627

Zero-point correction = 0.238493 (Hartree/Particle)

Thermal correction to Energy = 0.250232

Thermal correction to Enthalpy = 0.251177

Thermal correction to Gibbs Free Energy = 0.199739

SCF Done: E(RB3LYP) = -595.452695757 Hartree

TS[13-14]

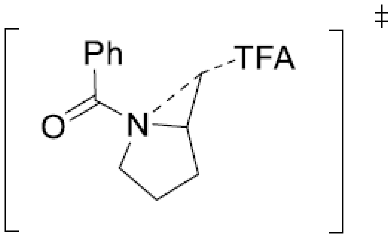

C 0.21166 1.93727 0.50731

H 0.24422 2.01994 1.59437

C 0.0869 3.24009 -0.30183

H -0.78257 3.19271 -0.9658

H -0.04768 4.10089 0.35685

O -2.72712 0.54701 0.41535

C -3.10817 -0.56942 -0.03832

C -4.65438 -0.7914 0.07018

O -2.43806 -1.50133 -0.51788

F -5.07944 -1.93112 -0.51468

F -5.35458 0.22641 -0.49359

F -5.0377 -0.85555 1.37947

H 2.1716 3.83796 -0.52991

H 1.28405 3.85295 -2.06112

C 1.40006 3.32267 -1.11166

N 1.27537 1.16138 -0.03811

C 2.23014 0.58905 0.8722

O 2.31091 0.99354 2.0129

C 3.07909 -0.51285 0.33376

C 2.65323 -1.36116 -0.70032

C 4.32194 -0.73656 0.94753

C 3.46652 -2.41397 -1.12087

H 1.67665 -1.22142 -1.152

C 5.13819 -1.77952 0.51427

H 4.62597 -0.0908 1.76462

C 4.712 -2.61929 -0.52025

H 3.12468 -3.07893 -1.9082

H 6.10106 -1.94429 0.98801

H 5.34473 -3.43783 -0.85066

H 2.87523 1.7081 -1.46737

H 1.27586 1.40123 -2.15649

C 1.80623 1.85554 -1.31198

C -0.31866 0.53468 -0.05712

H -0.3819 -0.31175 0.65094

H -0.50827 0.39942 -1.11762

Zero-point correction = 0.264695 (Hartree/Particle)

Thermal correction to Energy = 0.283716

Thermal correction to Enthalpy = 0.284660

Thermal correction to Gibbs Free Energy = 0.212184

SCF Done: E(RB3LYP) = -1121.88063434 Hartree

Imaginary frequency= -54.28 cm^{-1}

14

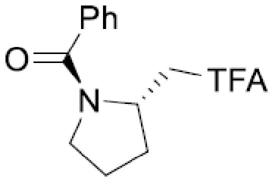

C 0.21993 0.83048 -0.43067

H 0.31488 0.57367 -1.4921

C 0.55422 2.31378 -0.17637

H 0.95439 2.43977 0.83744

H 1.3035 2.68679 -0.87776

O 2.44023 0.00696 -0.07866

C 3.35885 -0.71191 0.55436

C 4.75653 -0.44933 -0.06867

O 3.1872 -1.4715 1.47584

F 5.6926 -1.17674 0.5502

F 5.0935 0.8552 0.04429

F 4.76638 -0.77033 -1.37975

H -1.0687 3.17147 -1.34942

H -0.82265 3.9983 0.19828

C -0.80279 3.0229 -0.29653

N -1.206 0.70902 -0.04023

C -1.9205 -0.36442 -0.52299

O -1.37714 -1.22774 -1.21633

C -3.37284 -0.48197 -0.15671

C -3.85993 -0.18509 1.12398

C -4.24933 -1.00281 -1.12141

C -5.20767 -0.39052 1.4305

H -3.18661 0.18235 1.89207

C -5.59809 -1.18656 -0.82095

H -3.85424 -1.26592 -2.09723

C -6.08047 -0.88119 0.45652

H -5.5719 -0.17262 2.4302

H -6.27215 -1.57735 -1.57751

H -7.12923 -1.0355 0.69324

H -2.79446 2.12134 -0.00016

H -1.76517 2.13347 1.44295

C -1.76626 2.02224 0.35079

C 1.06502 -0.15521 0.37328

H 0.75906 -1.18538 0.1846

H 1.02052 0.04906 1.44671

Zero-point correction = 0.268103 (Hartree/Particle)

Thermal correction to Energy = 0.287045

Thermal correction to Enthalpy = 0.287989

Thermal correction to Gibbs Free Energy = 0.216407

SCF Done: E(RB3LYP) = -1121.95686024 Hartree

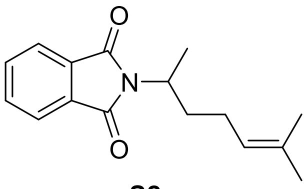

S2p

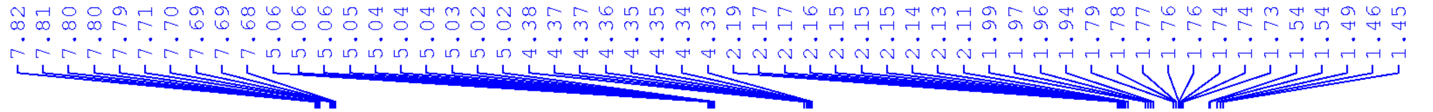
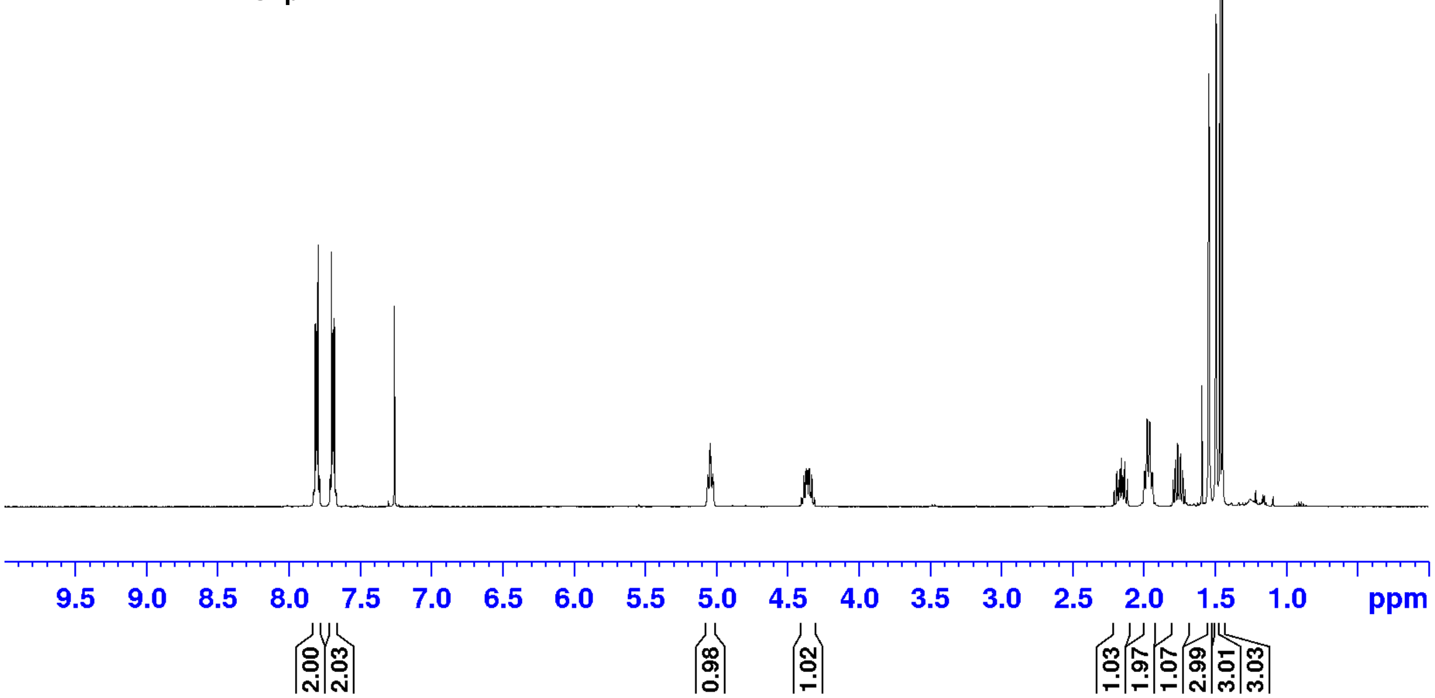


```
Current Data Parameters
NAME                 KK-518
EXPNO                    11
PROCNO                    1

F2 - Acquisition Parameters
Date_              20221204
Time                  11.30 h
INSTRUM               spect
PROBHD     Z108618_0435 (
PULPROG                zg30
TD                    65536
SOLVENT               CDCl3
NS                       16
DS                        2
SWH                8223.685 Hz
FIDRES             0.250967 Hz
AQ                3.9845889 sec
RG                   147.88
DW                   60.800 usec
DE                    10.50 usec
TE                    298.1 K
D1               2.00000000 sec
TD0                       1
SFO1            400.1324710 MHz
NUC1                     1H
P0                     3.67 usec
P1                    11.00 usec
PLW1            28.00000000 W

F2 - Processing parameters
SI                    32768
SF              400.1300104 MHz
WDW                      EM
SSB                       0
LB                     0.30 Hz
GB                        0
PC                     1.50
```

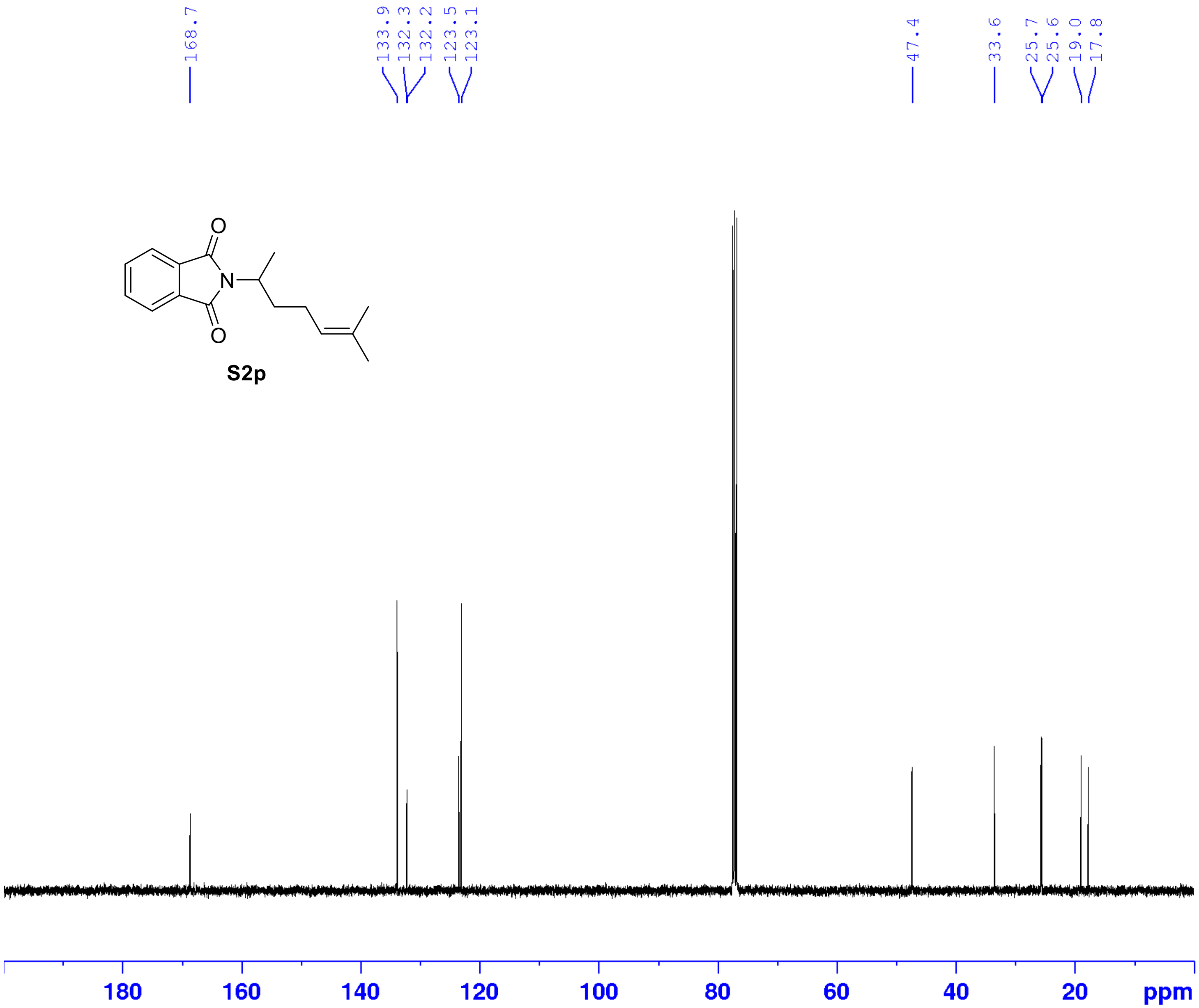

BRUKER

Current Data Parameters
NAME KK-518
EXPNO 12
PROCNO 1

F2 - Acquisition Parameters
Date_ 20221204
Time 11.49 h
INSTRUM spect
PROBHD Z108618_0435 (
PULPROG zgpg30
TD 65536
SOLVENT CDCl3
NS 256
DS 4
SWH 24038.461 Hz
FIDRES 0.733596 Hz
AQ 1.3631488 sec
RG 181.72
DW 20.800 usec
DE 8.63 usec
TE 298.2 K
D1 2.00000000 sec
D11 0.03000000 sec
TD0 8
SFO1 100.6228303 MHz
NUC1 13C
P0 2.83 usec
P1 8.50 usec
PLW1 73.00000000 W
SFO2 400.1316005 MHz
NUC2 1H
CPDPRG[2 waltz16
PCPD2 90.00 usec
PLW2 28.00000000 W
PLW12 0.41826999 W
PLW13 0.21039000 W

F2 - Processing parameters
SI 65536
SF 100.6127552 MHz
WDW EM
SSB 0
LB 1.00 Hz
GB 0
PC 1.40

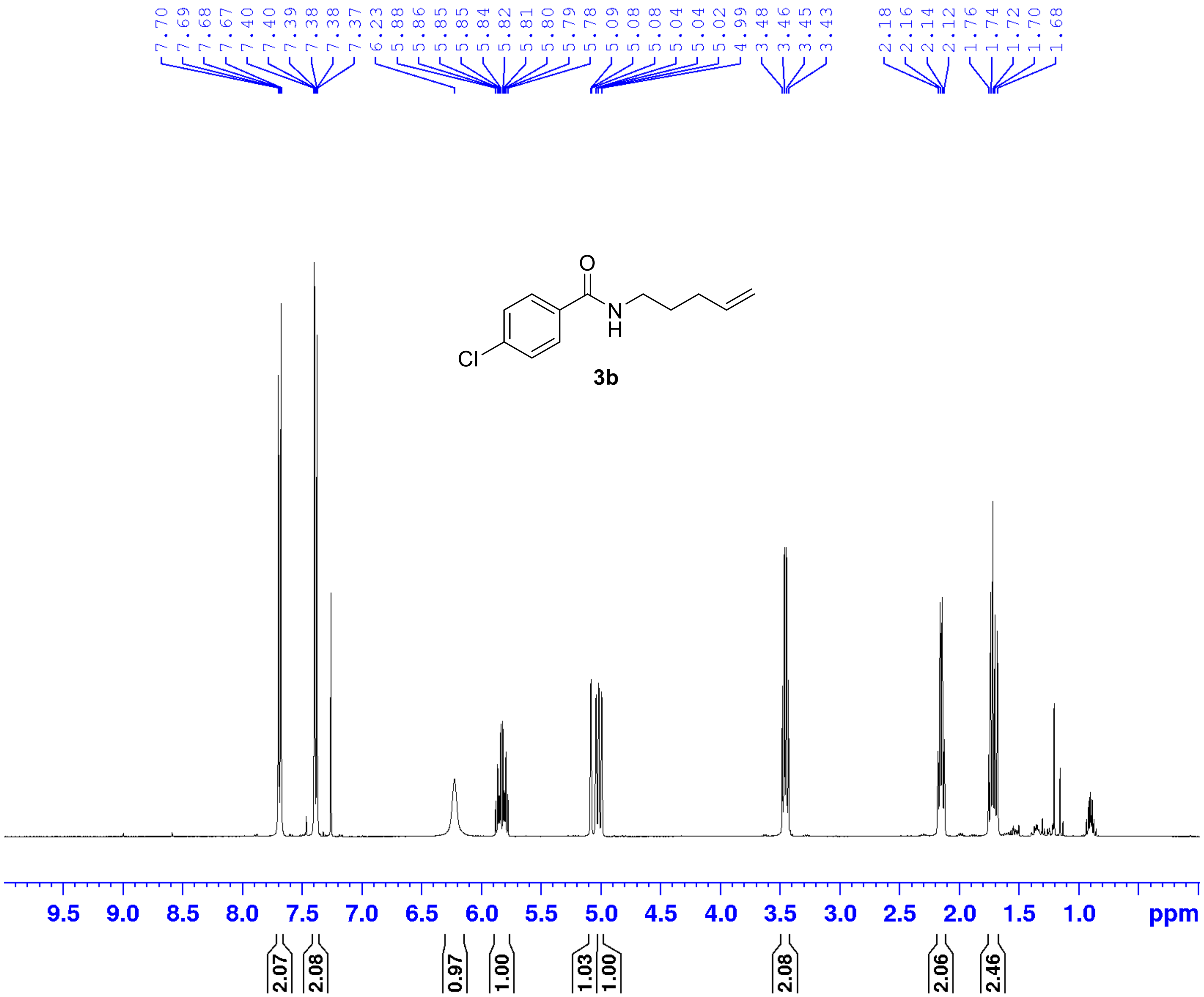


```
Current Data Parameters
NAME                KK-480
EXPNO                   60
PROCNO                   1

F2 - Acquisition Parameters
Date_             20221019
Time                 10.47 h
INSTRUM              spect
PROBHD    Z116098_0048 (
PULPROG               zg30
TD                   65536
SOLVENT              CDCl3
NS                      16
DS                       2
SWH               8223.685 Hz
FIDRES            0.250967 Hz
AQ               3.9845889 sec
RG                  104.33
DW                  60.800 usec
DE                   10.80 usec
TE                   298.1 K
D1              2.00000000 sec
TD0                      1
SFO1           400.1324710 MHz
NUC1                    1H
P0                    3.08 usec
P1                    9.25 usec
PLW1           24.00000000 W

F2 - Processing parameters
SI                   32768
SF             400.1300098 MHz
WDW                     EM
SSB                      0
LB                    0.30 Hz
GB                       0
PC                    1.50
```

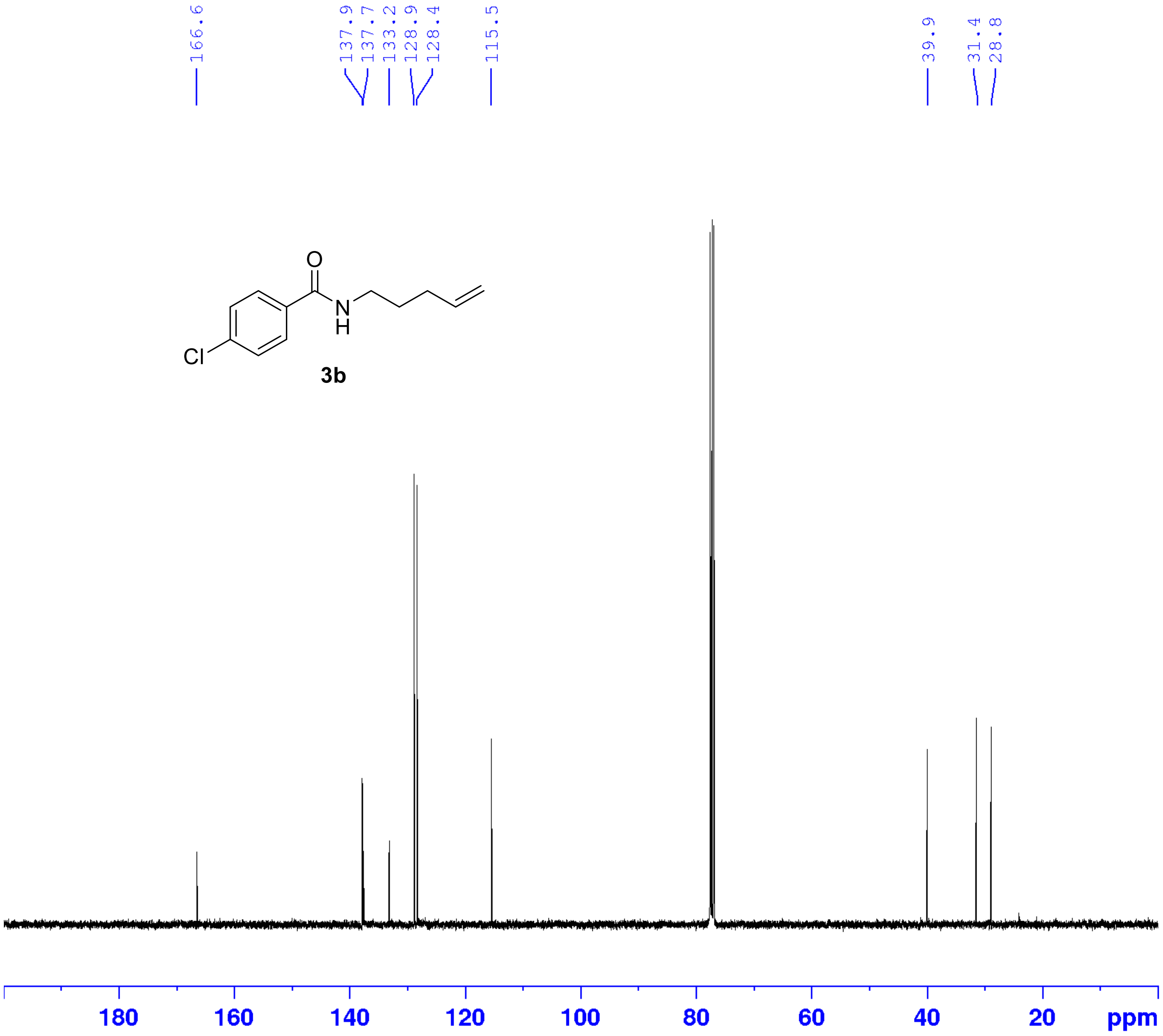

Current Data Parameters
NAME KK-480
EXPNO 62
PROCNO 1

F2 - Acquisition Parameters
Date_ 20221020
Time 0.23 h
INSTRUM spect
PROBHD Z116098_0048 (
PULPROG zgpg30
TD 65536
SOLVENT CDCl3
NS 512
DS 4
SWH 24038.461 Hz
FIDRES 0.733596 Hz
AQ 1.3631488 sec
RG 181.72
DW 20.800 usec
DE 8.54 usec
TE 298.2 K
D1 2.00000000 sec
D11 0.03000000 sec
TD0 8
SFO1 100.6228303 MHz
NUC1 13C
P0 3.00 usec
P1 9.00 usec
PLW1 77.00000000 W
SFO2 400.1316005 MHz
NUC2 1H
CPDPRG[2 waltz16
PCPD2 90.00 usec
PLW2 24.00000000 W
PLW12 0.25352001 W
PLW13 0.12751999 W

F2 - Processing parameters
SI 65536
SF 100.6127561 MHz
WDW EM
SSB 0
LB 1.00 Hz
GB 0
PC 1.40

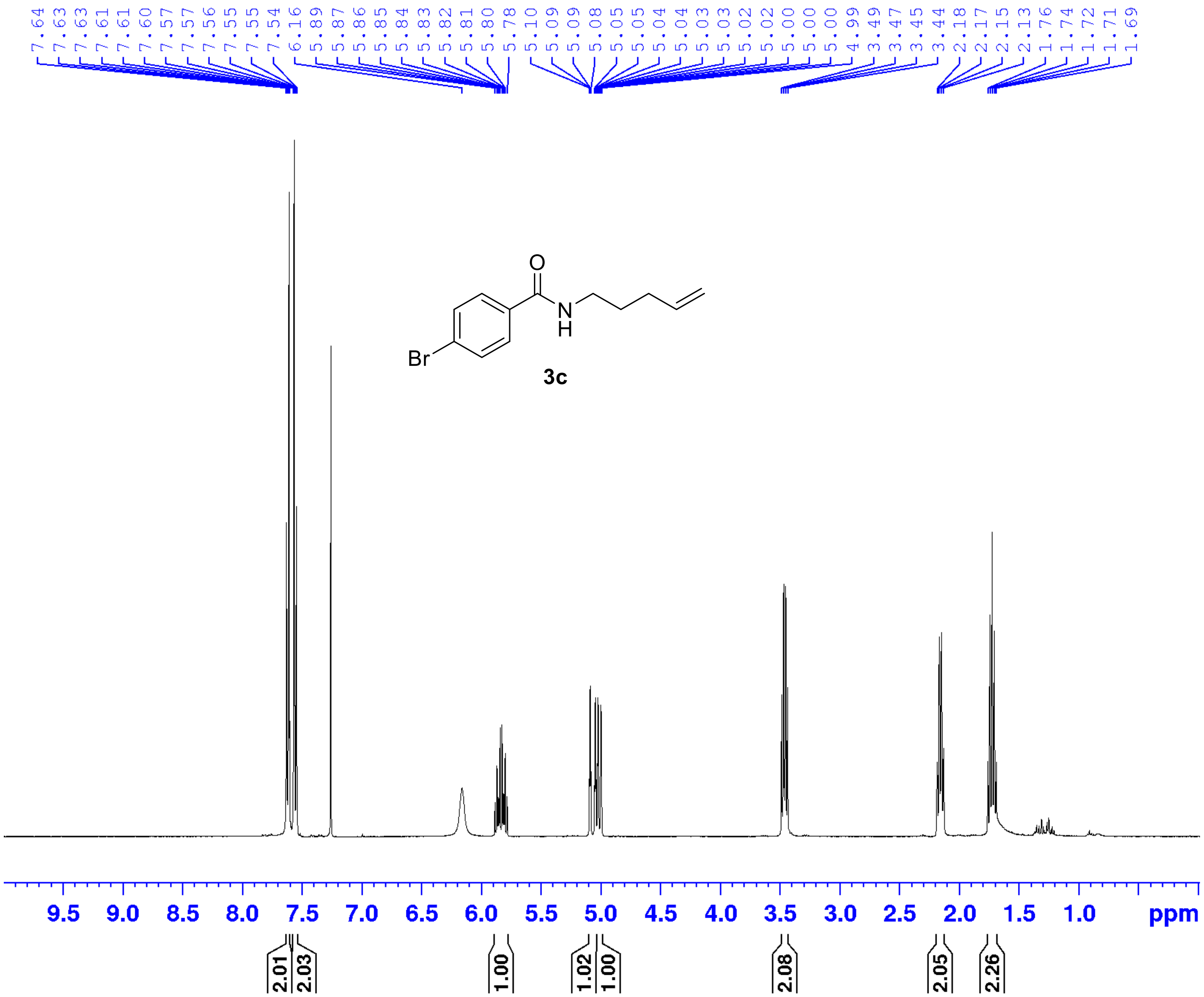

Current Data Parameters
NAME KK-559
EXPNO 10
PROCNO 1

F2 - Acquisition Parameters
Date_ 20230628
Time 10.58 h
INSTRUM spect
PROBHD Z116098_0048 (
PULPROG zg30
TD 65536
SOLVENT CDCl3
NS 16
DS 2
SWH 8223.685 Hz
FIDRES 0.250967 Hz
AQ 3.9845889 sec
RG 147.88
DW 60.800 usec
DE 10.80 usec
TE 298.1 K
D1 2.00000000 sec
TD0 1
SFO1 400.1324710 MHz
NUC1 1H
P0 3.08 usec
P1 9.25 usec
PLW1 24.00000000 W

F2 - Processing parameters
SI 32768
SF 400.1300103 MHz
WDW EM
SSB 0
LB 0.30 Hz
GB 0
PC 1.50

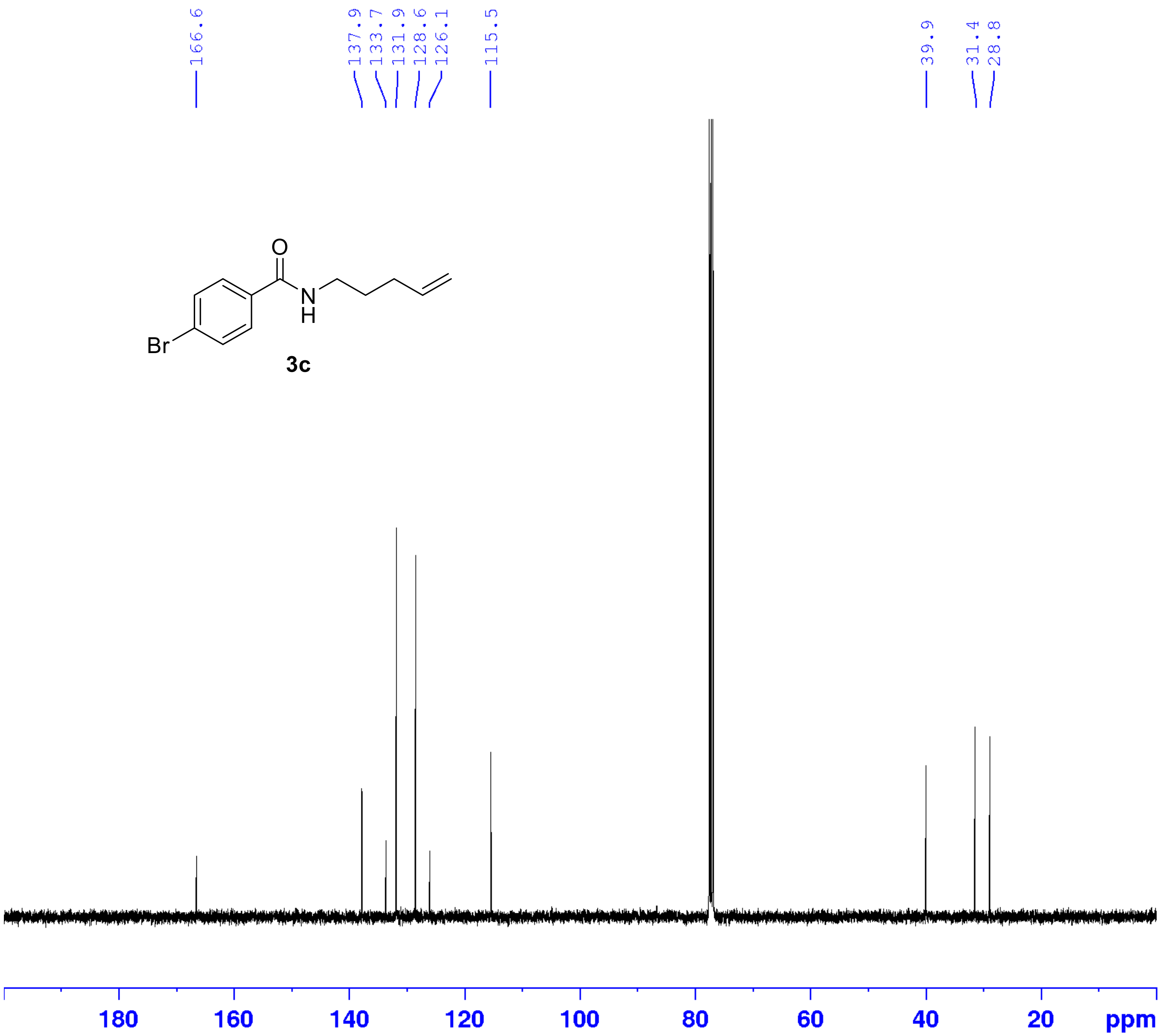

Current Data Parameters

NAME	KK-559
EXPNO	12
PROCNO	1

F2 - Acquisition Parameters

Date_	20230628
Time	19.18 h
INSTRUM	spect
PROBHD	Z116098_0048 (
PULPROG	zgpg30
TD	65536
SOLVENT	CDCl3
NS	512
DS	4
SWH	24038.461 Hz
FIDRES	0.733596 Hz
AQ	1.3631488 sec
RG	181.72
DW	20.800 usec
DE	8.54 usec
TE	298.2 K
D1	2.00000000 sec
D11	0.03000000 sec
TD0	8
SFO1	100.6228303 MHz
NUC1	13C
P0	3.00 usec
P1	9.00 usec
PLW1	77.00000000 W
SFO2	400.1316005 MHz
NUC2	1H
CPDPRG[2	waltz16
PCPD2	90.00 usec
PLW2	24.00000000 W
PLW12	0.25352001 W
PLW13	0.12751999 W

F2 - Processing parameters

SI	65536
SF	100.6127561 MHz
WDW	EM
SSB	0
LB	1.00 Hz
GB	0
PC	1.40

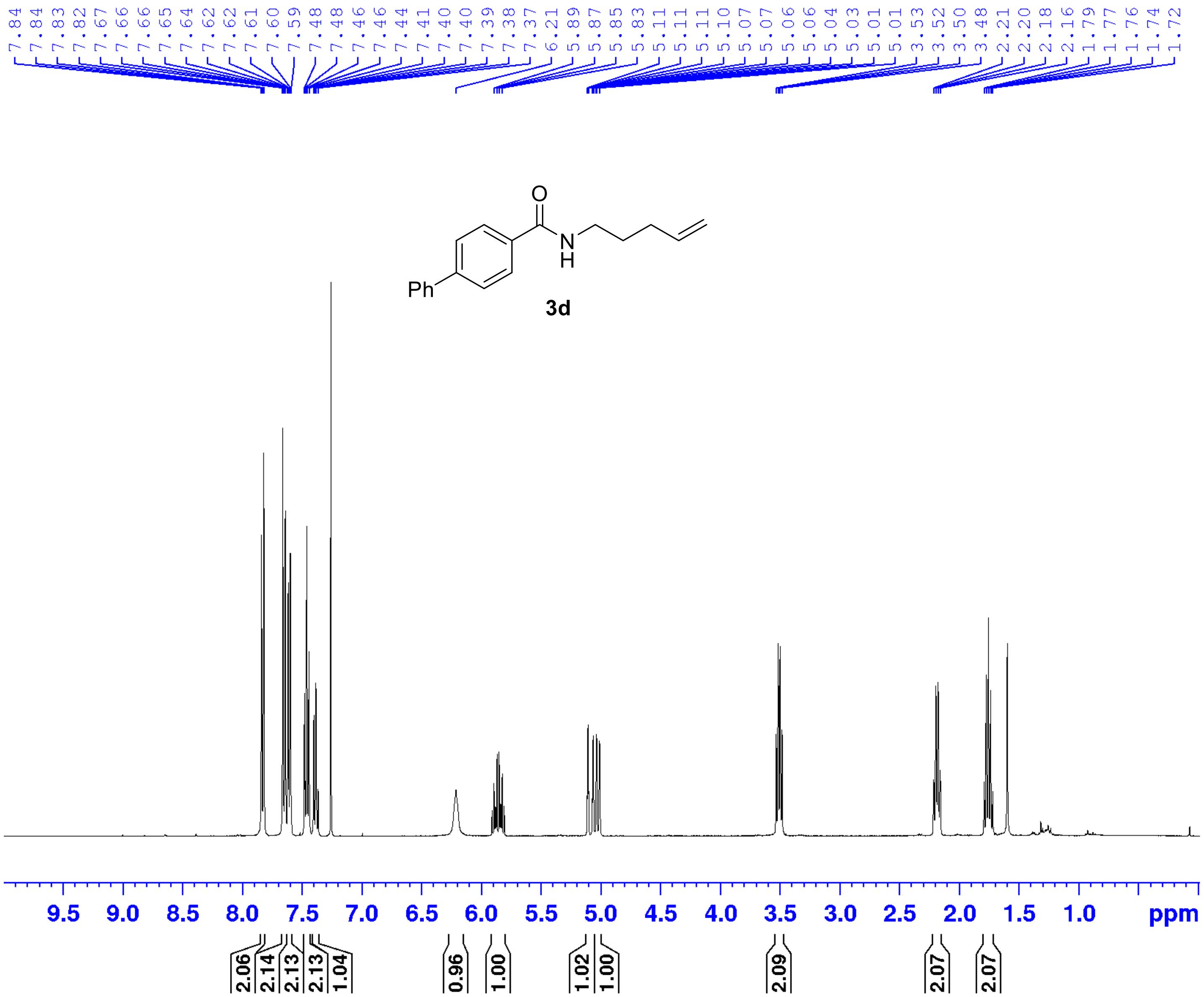

3d

Current Data Parameters
NAME KK-503
EXPNO 10
PROCNO 1

F2 - Acquisition Parameters
Date_ 20221026
Time 10.10 h
INSTRUM spect
PROBHD Z116098_0048 (
PULPROG zg30
TD 65536
SOLVENT CDCl3
NS 16
DS 2
SWH 8223.685 Hz
FIDRES 0.250967 Hz
AQ 3.9845889 sec
RG 147.88
DW 60.800 usec
DE 10.80 usec
TE 298.2 K
D1 2.00000000 sec
TD0 1
SFO1 400.1324710 MHz
NUC1 1H
P0 3.08 usec
P1 9.25 usec
PLW1 24.00000000 W

F2 - Processing parameters
SI 32768
SF 400.1300101 MHz
WDW EM
SSB 0
LB 0.30 Hz
GB 0
PC 1.50

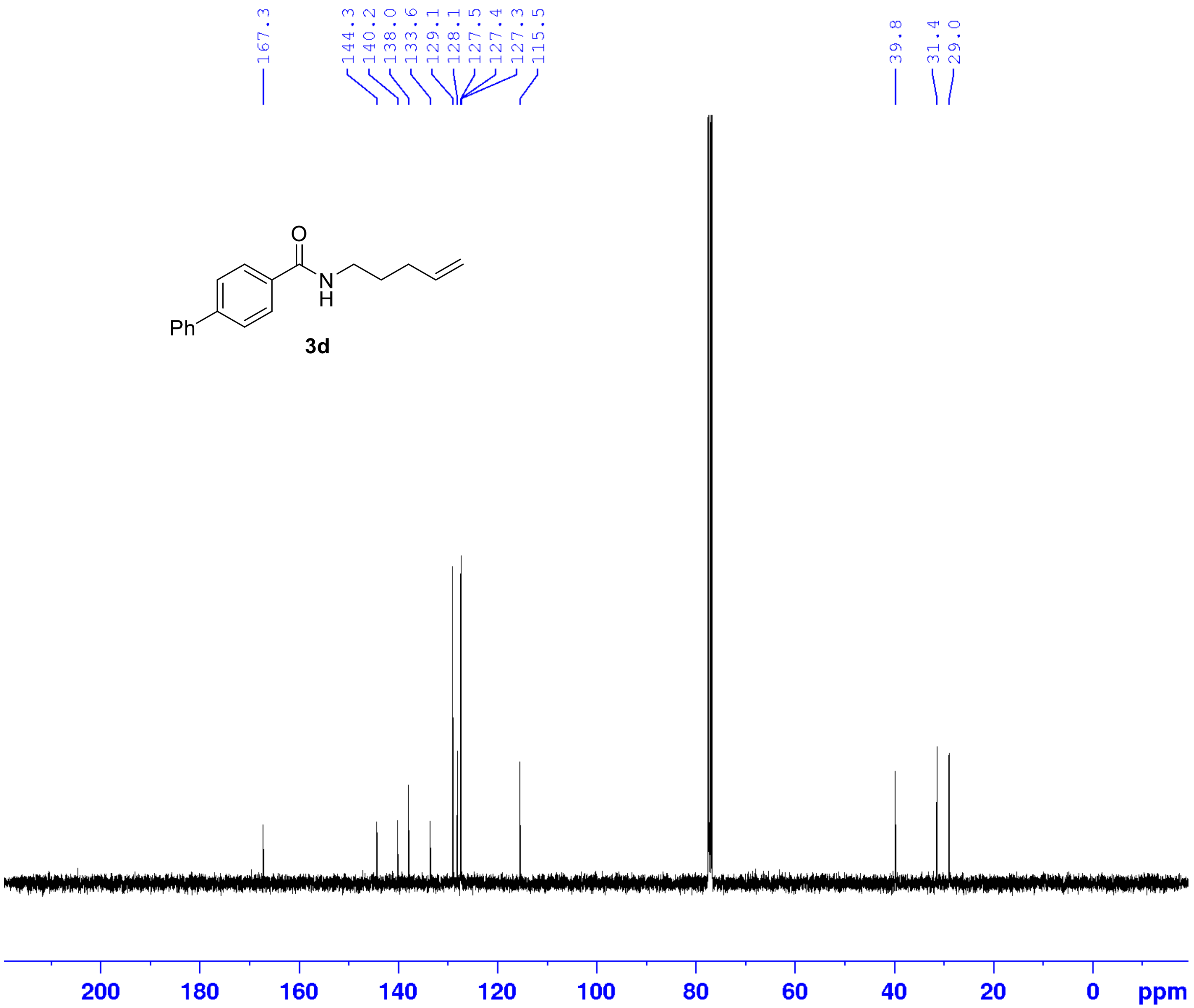

BRUKER

```
Current Data Parameters
NAME                 KK-503
EXPNO                    21
PROCNO                    1

F2 - Acquisition Parameters
Date_              20221026
Time                  20.24 h
INSTRUM               spect
PROBHD    Z116098_0048 (
PULPROG             zgpg30
TD                    65536
SOLVENT               CDCl3
NS                      512
DS                        4
SWH               24038.461 Hz
FIDRES             0.733596 Hz
AQ                1.3631488 sec
RG                   181.72
DW                   20.800 usec
DE                     8.54 usec
TE                    298.1 K
D1               2.00000000 sec
D11              0.03000000 sec
TD0                       8
SFO1            100.6228303 MHz
NUC1                    13C
P0                     3.00 usec
P1                     9.00 usec
PLW1            77.00000000 W
SFO2            400.1316005 MHz
NUC2                     1H
CPDPRG[2            waltz16
PCPD2                 90.00 usec
PLW2            24.00000000 W
PLW12            0.25352001 W
PLW13            0.12751999 W

F2 - Processing parameters
SI                    65536
SF              100.6127556 MHz
WDW                      EM
SSB                       0
LB                     1.00 Hz
GB                        0
PC                     1.40
```

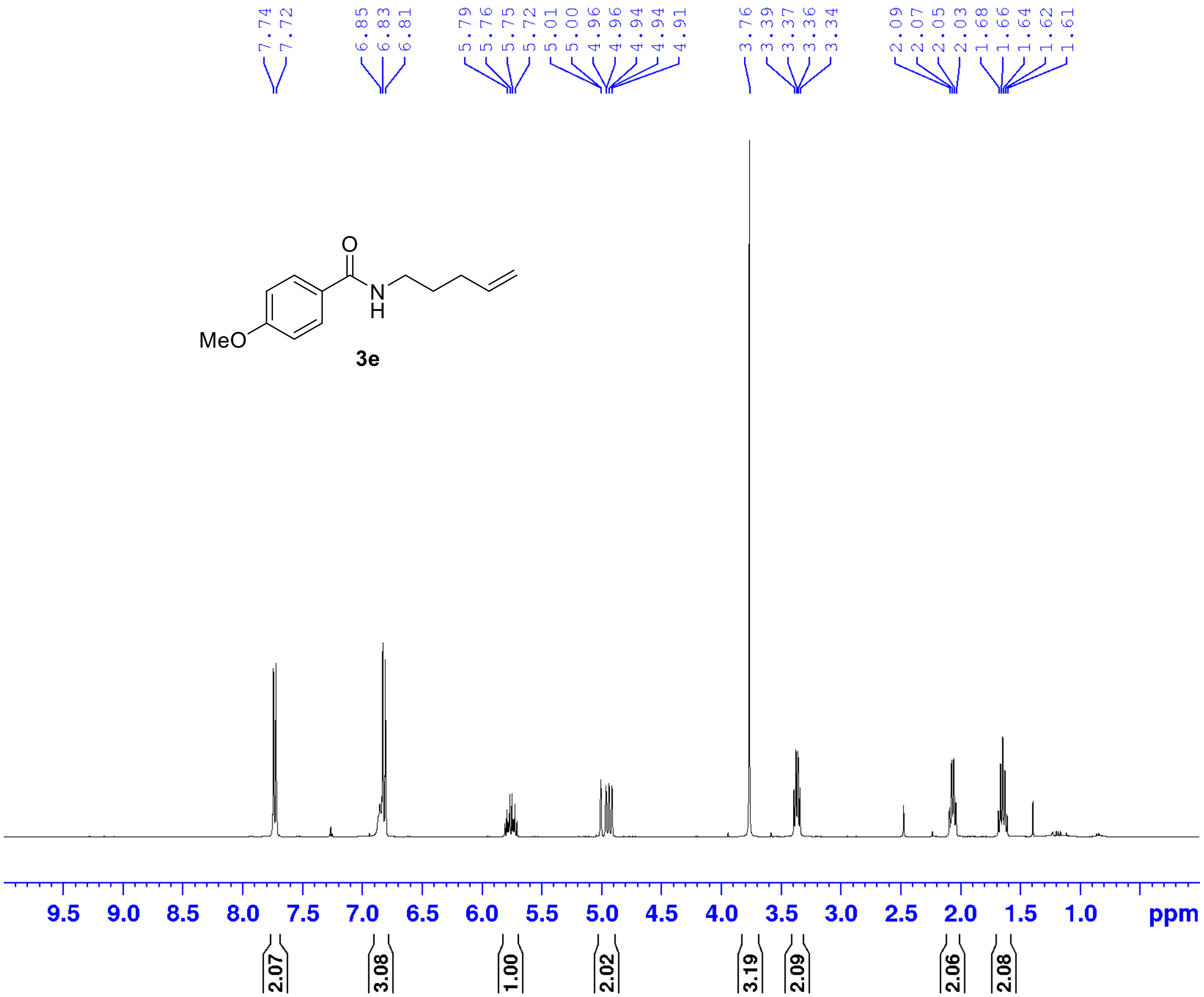

BRUKER

Current Data Parameters
NAME KK-505
EXPNO 13
PROCNO 1

F2 - Acquisition Parameters
Date_ 20240319
Time 12.19 h
INSTRUM spect
PROBHD Z116098_0048 (
PULPROG zg30
TD 65536
SOLVENT CDCl3
NS 16
DS 2
SWH 8223.685 Hz
FIDRES 0.250967 Hz
AQ 3.9845889 sec
RG 17.7
DW 60.800 usec
DE 10.80 usec
TE 298.2 K
D1 2.00000000 sec
TD0 1
SFO1 400.1324710 MHz
NUC1 1H
P0 3.08 usec
P1 9.25 usec
PLW1 24.00000000 W

F2 - Processing parameters
SI 32768
SF 400.1300087 MHz
WDW EM
SSB 0
LB 0.30 Hz
GB 0
PC 1.50

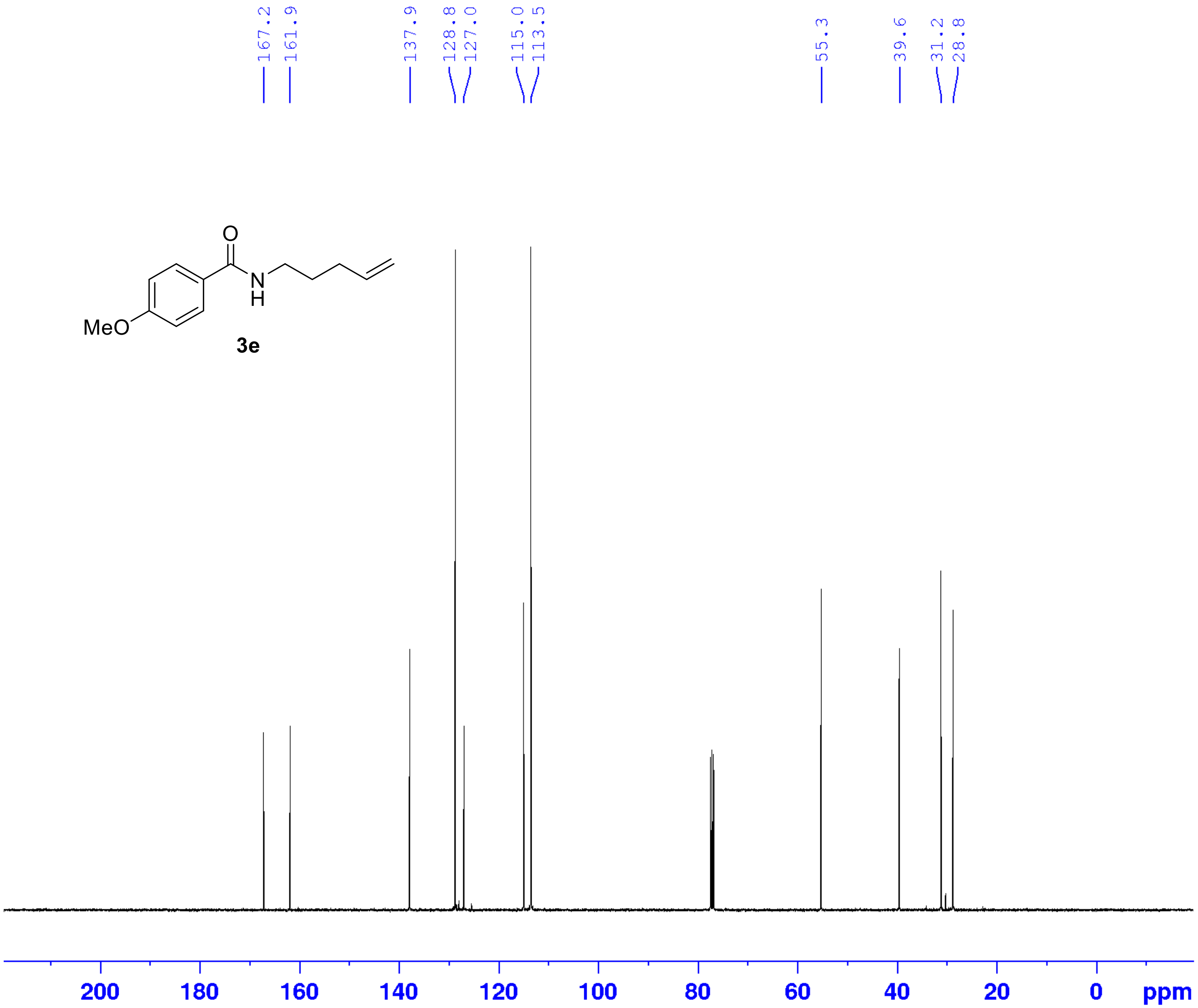

3e

BRUKER

Current Data Parameters	
NAME	KK-505
EXPNO	20
PROCNO	1
F2 - Acquisition Parameters	
Date_	20240319
Time	14.24 h
INSTRUM	spect
PROBHD	Z116098_0048 (
PULPROG	zgpg30
TD	65536
SOLVENT	CDCl3
NS	256
DS	4
SWH	24038.461 Hz
FIDRES	0.733596 Hz
AQ	1.3631488 sec
RG	181.72
DW	20.800 usec
DE	8.54 usec
TE	298.1 K
D1	2.00000000 sec
D11	0.03000000 sec
TD0	8
SFO1	100.6228303 MHz
NUC1	13C
P0	3.00 usec
P1	9.00 usec
PLW1	77.00000000 W
SFO2	400.1316005 MHz
NUC2	1H
CPDPRG[2	waltz16
PCPD2	90.00 usec
PLW2	24.00000000 W
PLW12	0.25352001 W
PLW13	0.12751999 W
F2 - Processing parameters	
SI	65536
SF	100.6127720 MHz
WDW	EM
SSB	0
LB	1.00 Hz
GB	0
PC	1.40

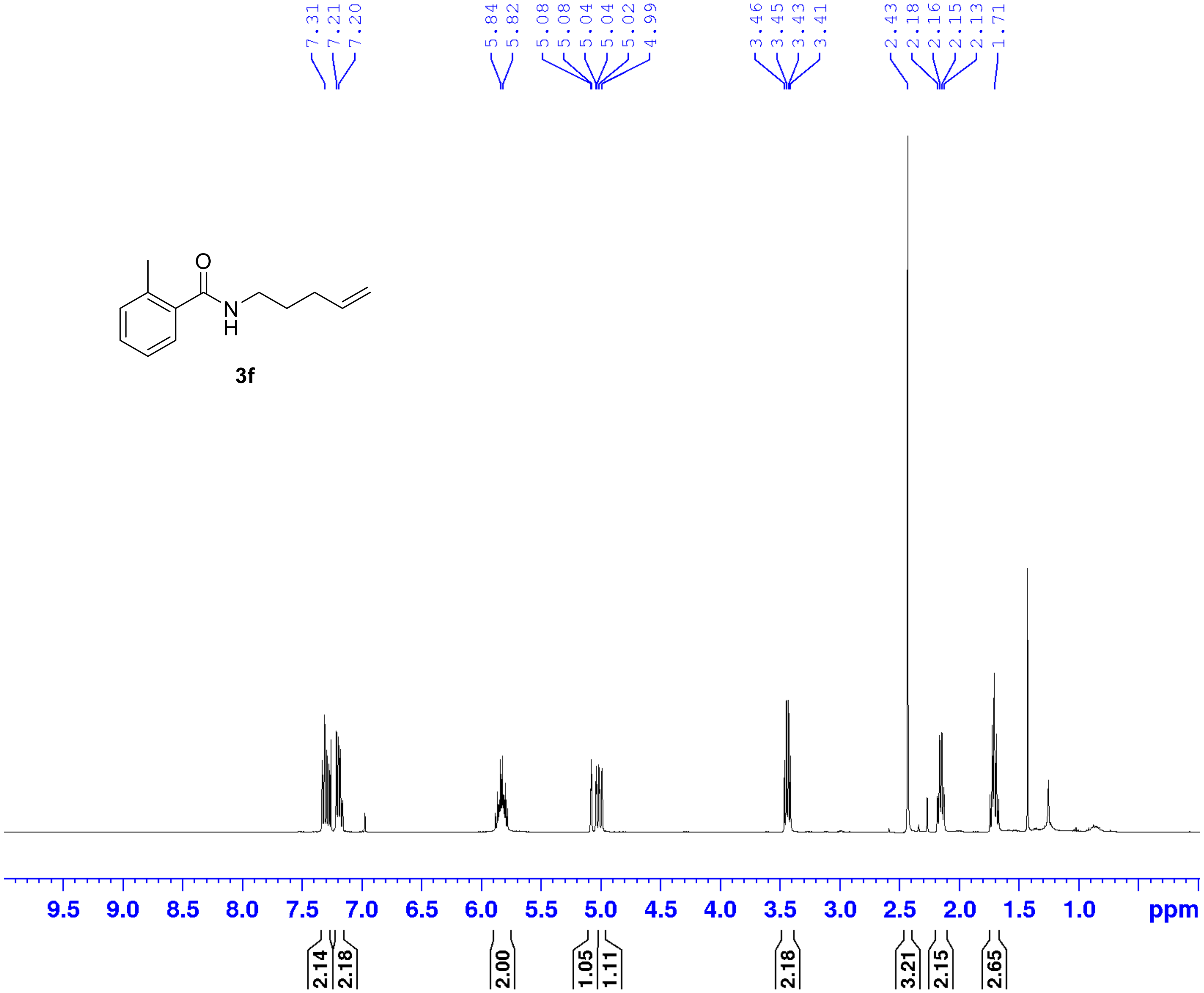

3f

BRUKER

Current Data Parameters
NAME KK-490
EXPNO 50
PROCNO 1

F2 - Acquisition Parameters
Date_ 20240320
Time 11.08 h
INSTRUM spect
PROBHD Z116098_0048 (
PULPROG zg30
TD 65536
SOLVENT CDCl3
NS 16
DS 2
SWH 8223.685 Hz
FIDRES 0.250967 Hz
AQ 3.9845889 sec
RG 56.49
DW 60.800 usec
DE 10.80 usec
TE 298.1 K
D1 2.00000000 sec
TD0 1
SFO1 400.1324710 MHz
NUC1 1H
P0 3.08 usec
P1 9.25 usec
PLW1 24.00000000 W

F2 - Processing parameters
SI 32768
SF 400.1300103 MHz
WDW EM
SSB 0
LB 0.30 Hz
GB 0
PC 1.50

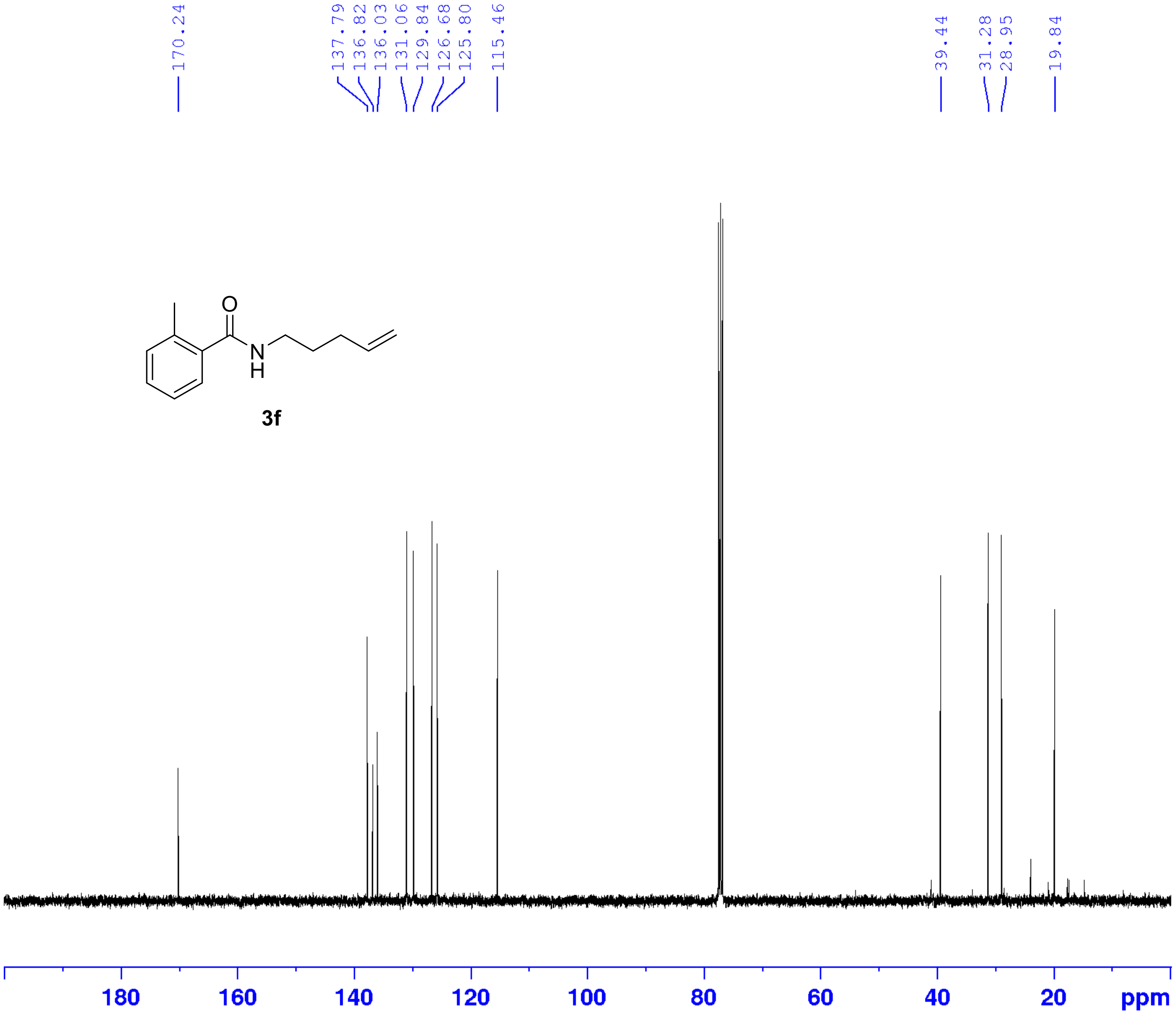

3f

Current Data Parameters		
NAME	KK-490	
EXPNO	41	
PROCNO	1	
F2 - Acquisition Parameters		
Date_	20221022	
Time	0.33	h
INSTRUM	spect	
PROBHD	Z116098_0048 (	
PULPROG	zgpg30	
TD	65536	
SOLVENT	CDCl3	
NS	256	
DS	4	
SWH	24038.461	Hz
FIDRES	0.733596	Hz
AQ	1.3631488	sec
RG	181.72	
DW	20.800	usec
DE	8.54	usec
TE	298.1	K
D1	2.00000000	sec
D11	0.03000000	sec
TD0	8	
SFO1	100.6228303	MHz
NUC1	13C	
P0	3.00	usec
P1	9.00	usec
PLW1	77.00000000	W
SFO2	400.1316005	MHz
NUC2	1H	
CPDPRG[2	waltz16	
PCPD2	90.00	usec
PLW2	24.00000000	W
PLW12	0.25352001	W
PLW13	0.12751999	W
F2 - Processing parameters		
SI	65536	
SF	100.6127588	MHz
WDW	EM	
SSB	0	
LB	1.00	Hz
GB	0	
PC	1.40	

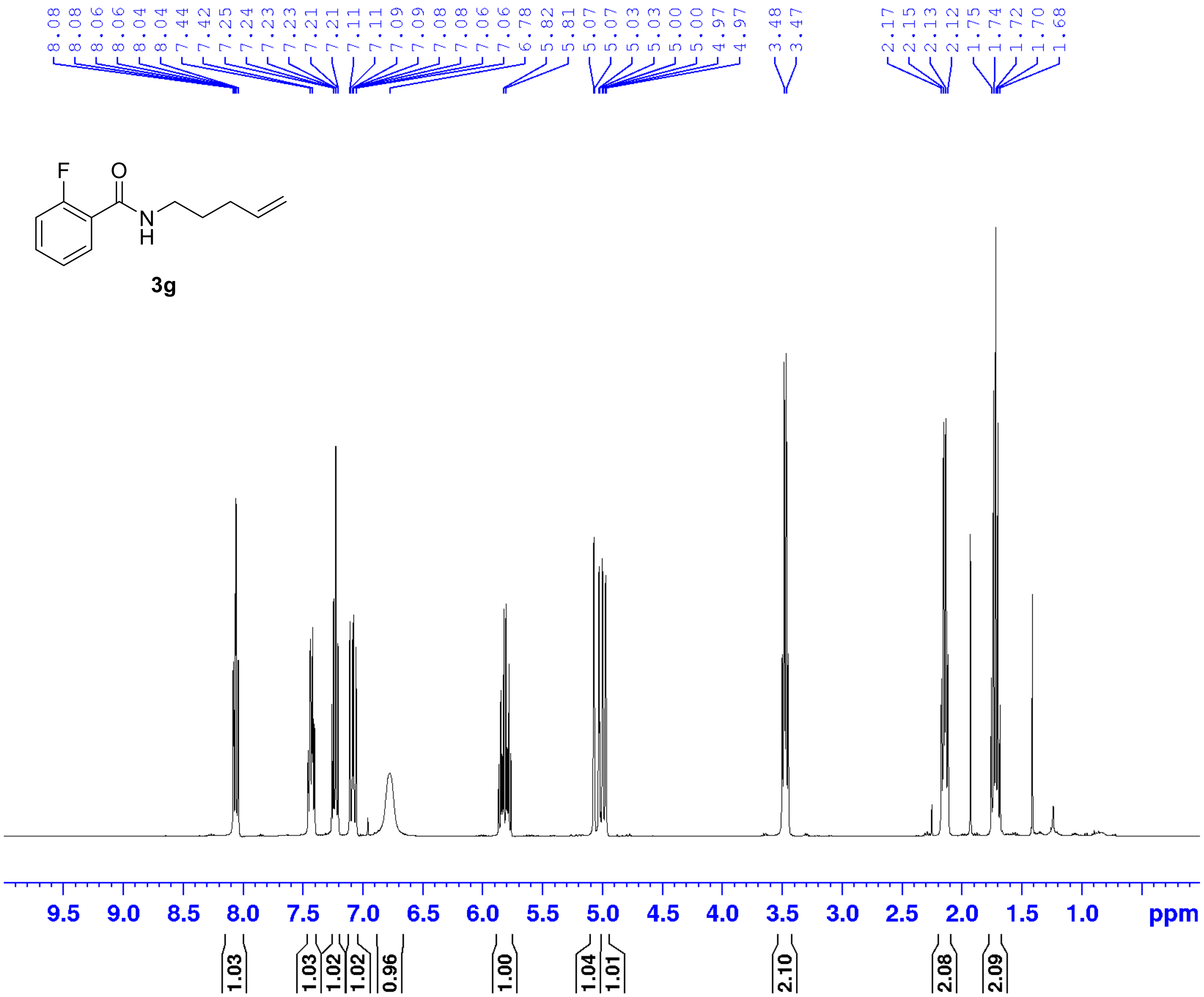

3g

Current Data Parameters

NAME	KK-523
EXPNO	50
PROCNO	1

F2 - Acquisition Parameters

Date_	20240320
Time	11.14 h
INSTRUM	spect
PROBHD	Z116098_0048 (
PULPROG	zg30
TD	65536
SOLVENT	CDCl3
NS	16
DS	2
SWH	8223.685 Hz
FIDRES	0.250967 Hz
AQ	3.9845889 sec
RG	35.7
DW	60.800 usec
DE	10.80 usec
TE	298.2 K
D1	2.00000000 sec
TD0	1
SFO1	400.1324710 MHz
NUC1	1H
P0	3.08 usec
P1	9.25 usec
PLW1	24.00000000 W

F2 - Processing parameters

SI	32768
SF	400.1300101 MHz
WDW	EM
SSB	0
LB	0.30 Hz
GB	0
PC	1.50

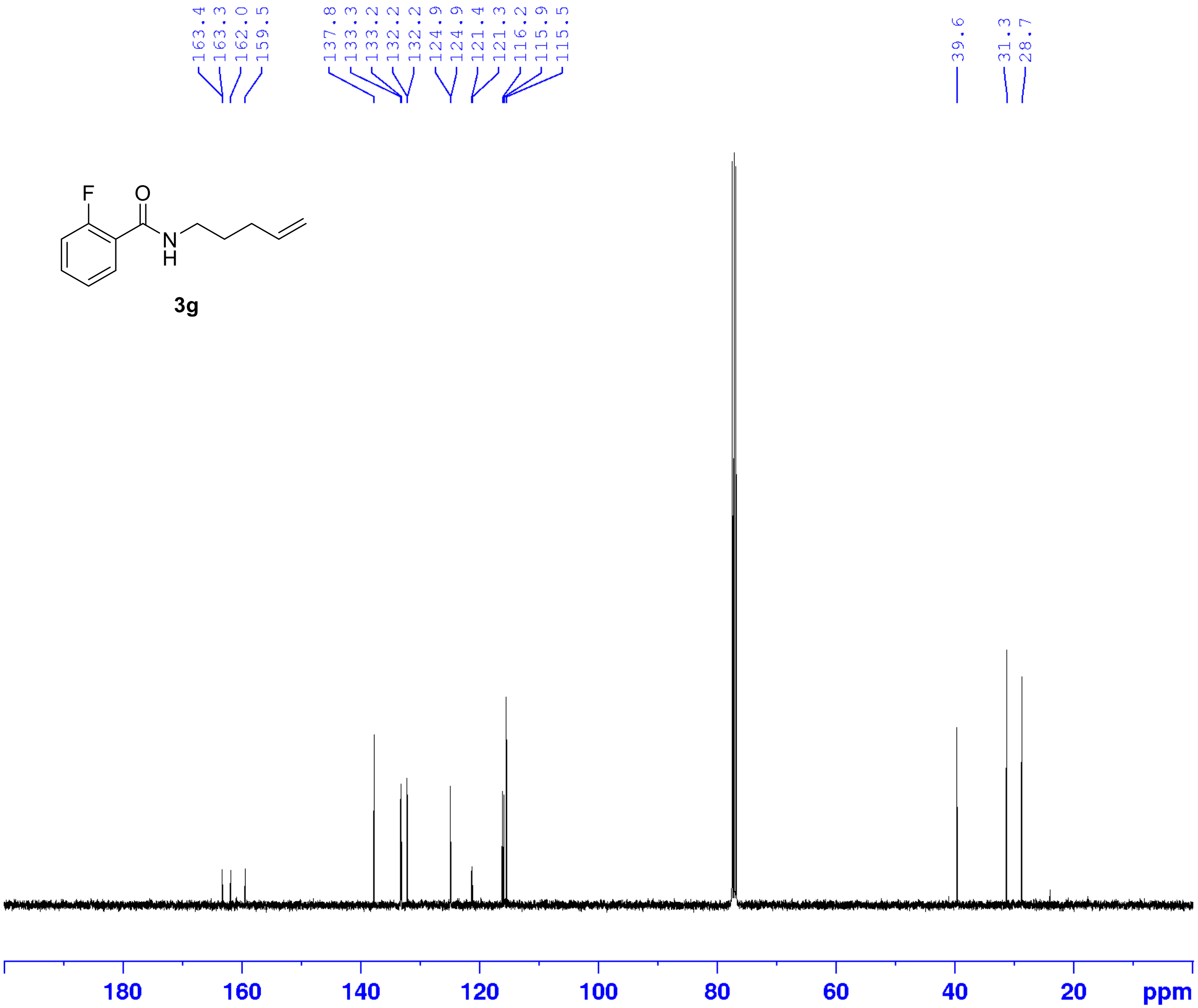

3g


```
Current Data Parameters
NAME                KK-523
EXPNO                   43
PROCNO                   1

F2 - Acquisition Parameters
Date_             20230626
Time                 19.19 h
INSTRUM              spect
PROBHD    Z116098_0048 (
PULPROG            zgpg30
TD                   65536
SOLVENT              CDCl3
NS                     512
DS                       4
SWH              24038.461 Hz
FIDRES            0.733596 Hz
AQ               1.3631488 sec
RG                  181.72
DW                  20.800 usec
DE                    8.54 usec
TE                   298.2 K
D1              2.00000000 sec
D11             0.03000000 sec
TD0                      8
SFO1           100.6228303 MHz
NUC1                   13C
P0                    3.00 usec
P1                    9.00 usec
PLW1           77.00000000 W
SFO2           400.1316005 MHz
NUC2                    1H
CPDPRG[2           waltz16
PCPD2                90.00 usec
PLW2           24.00000000 W
PLW12           0.25352001 W
PLW13           0.12751999 W

F2 - Processing parameters
SI                   65536
SF             100.6127559 MHz
WDW                     EM
SSB                      0
LB                    1.00 Hz
GB                       0
PC                    1.40
```

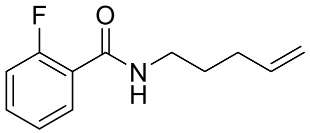

3g

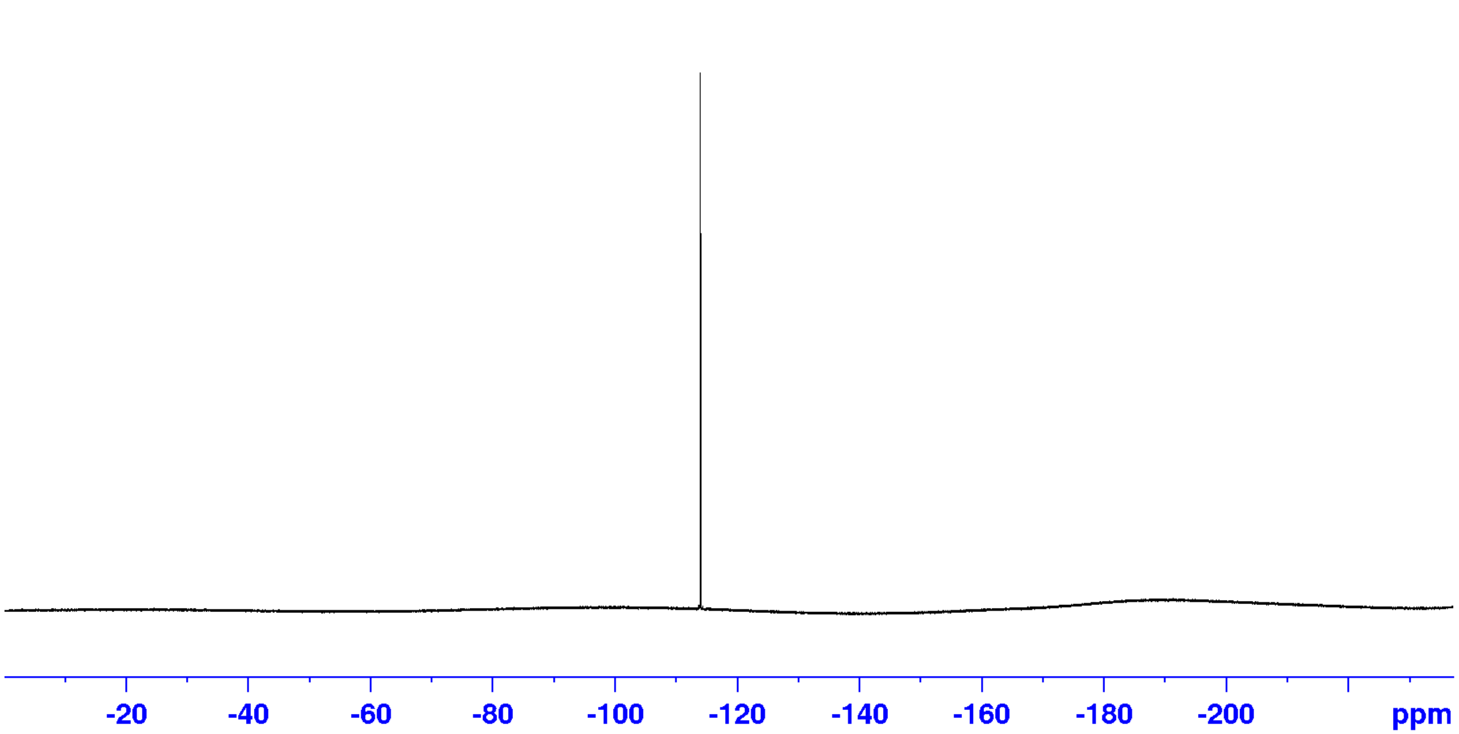

Current Data Parameters		
NAME	KK-523	
EXPNO	41	
PROCNO	1	
F2 - Acquisition Parameters		
Date_	20230626	
Time	11.15	h
INSTRUM	spect	
PROBHD	Z116098_0048 (	
PULPROG	zg	
TD	262144	
SOLVENT	CDCl3	
NS	16	
DS	0	
SWH	89285.711	Hz
FIDRES	0.681196	Hz
AQ	1.4680064	sec
RG	181.72	
DW	5.600	usec
DE	7.11	usec
TE	298.1	K
D1	4.00000000	sec
TD0	1	
SFO1	376.4536869	MHz
NUC1	19F	
P1	14.00	usec
PLW1	20.00000000	W
F2 - Processing parameters		
SI	262144	
SF	376.4983660	MHz
WDW	EM	
SSB	0	
LB	0.50	Hz
GB	0	
PC	1.00	

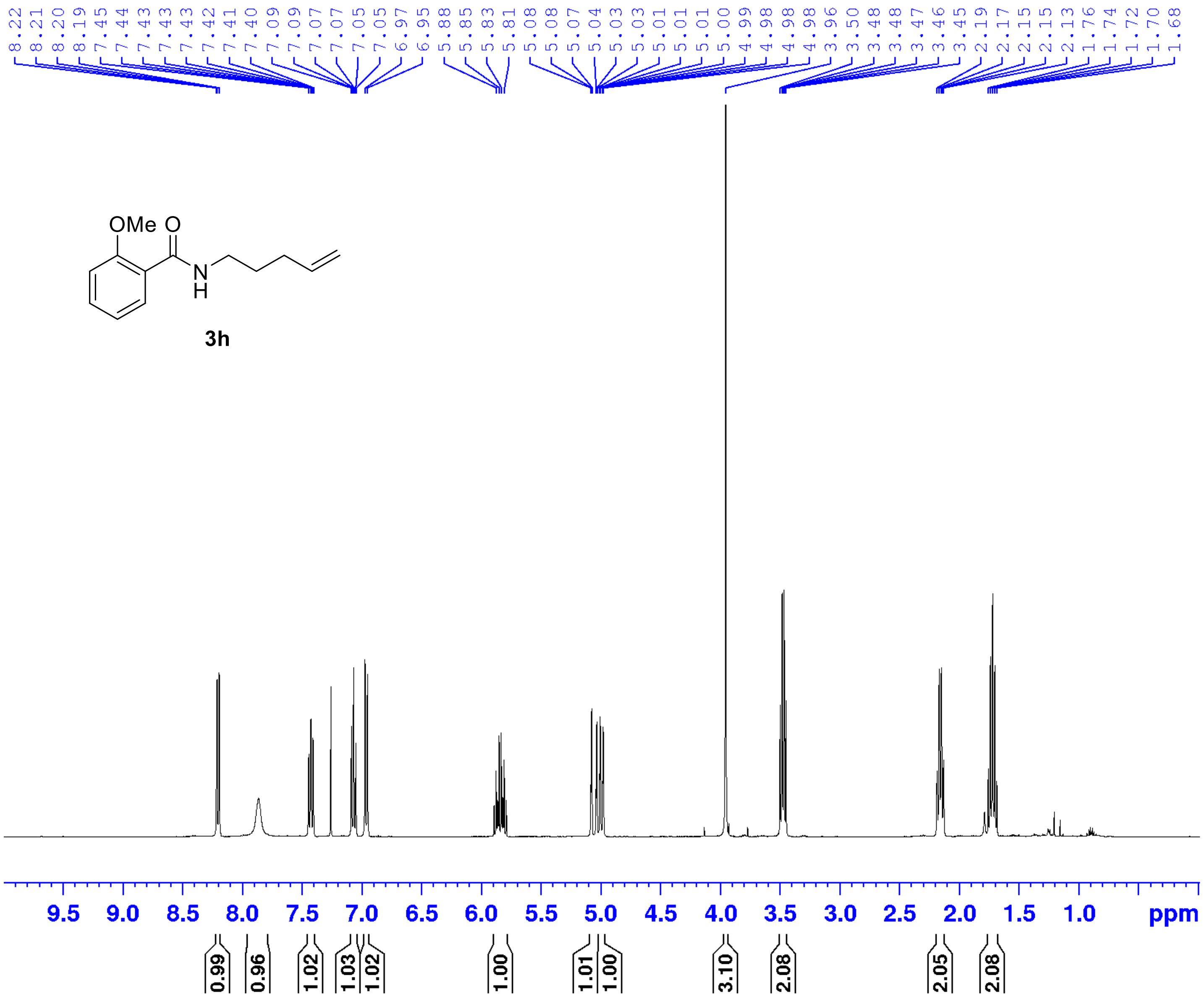


```
Current Data Parameters
NAME                KK-582
EXPNO                   10
PROCNO                   1

F2 - Acquisition Parameters
Date_             20230713
Time                 11.19 h
INSTRUM              spect
PROBHD    Z116098_0048 (
PULPROG               zg30
TD                   65536
SOLVENT              CDCl3
NS                      16
DS                       2
SWH               8223.685 Hz
FIDRES            0.250967 Hz
AQ               3.9845889 sec
RG                   50.36
DW                  60.800 usec
DE                   10.80 usec
TE                   298.2 K
D1              2.00000000 sec
TD0                      1
SFO1           400.1324710 MHz
NUC1                    1H
P0                    3.08 usec
P1                    9.25 usec
PLW1           24.00000000 W

F2 - Processing parameters
SI                   32768
SF             400.1300101 MHz
WDW                     EM
SSB                      0
LB                    0.30 Hz
GB                       0
PC                    1.50
```

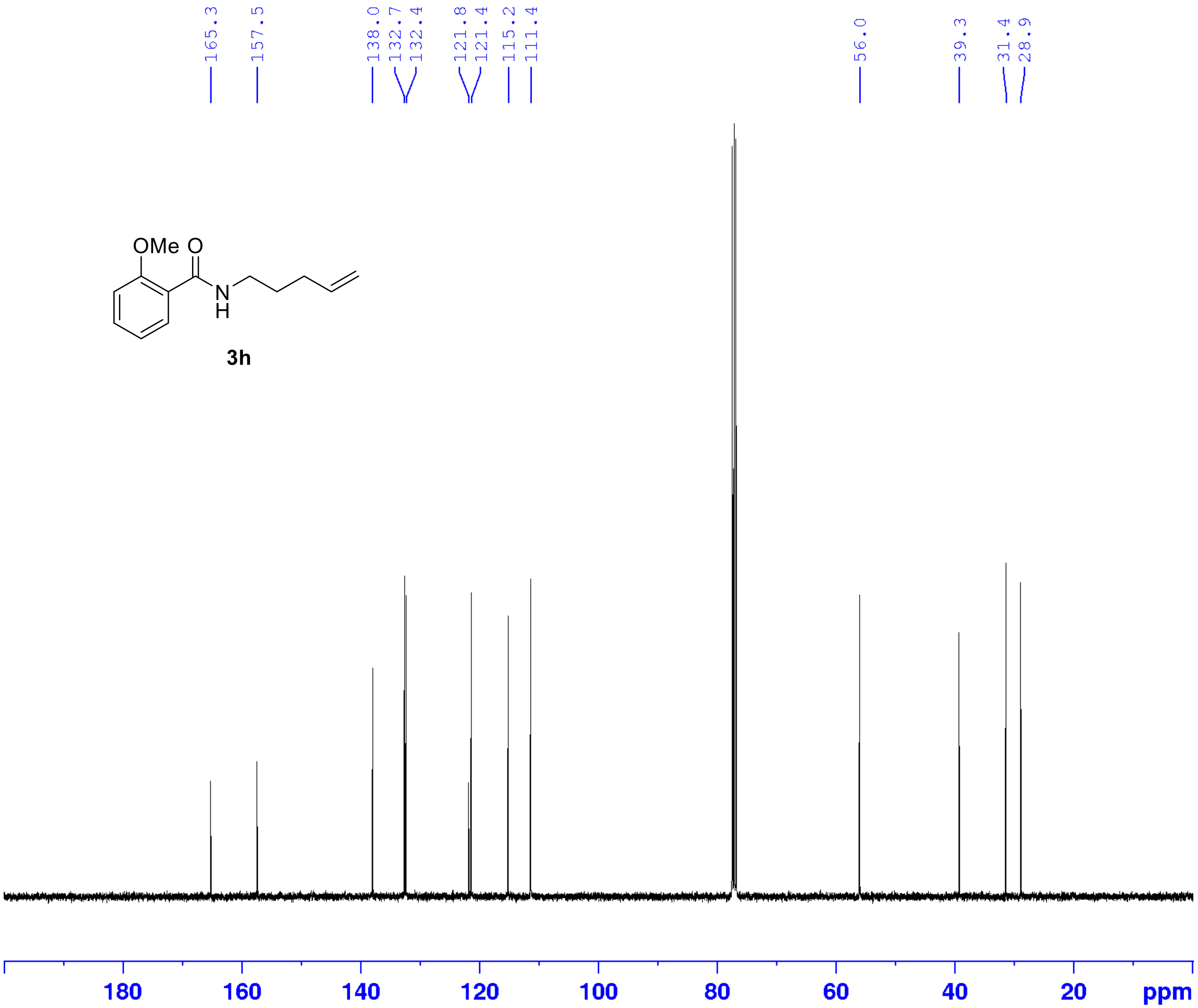

3h

```
Current Data Parameters
NAME                KK-582
EXPNO                   12
PROCNO                   1

F2 - Acquisition Parameters
Date_             20230713
Time                 19.19 h
INSTRUM              spect
PROBHD   Z116098_0048 (
PULPROG             zgpg30
TD                   65536
SOLVENT              CDCl3
NS                     512
DS                       4
SWH              24038.461 Hz
FIDRES            0.733596 Hz
AQ               1.3631488 sec
RG                  181.72
DW                  20.800 usec
DE                    8.54 usec
TE                   298.2 K
D1              2.00000000 sec
D11             0.03000000 sec
TD0                      8
SFO1           100.6228303 MHz
NUC1                   13C
P0                    3.00 usec
P1                    9.00 usec
PLW1           77.00000000 W
SFO2           400.1316005 MHz
NUC2                    1H
CPDPRG[2           waltz16
PCPD2                90.00 usec
PLW2           24.00000000 W
PLW12           0.25352001 W
PLW13           0.12751999 W

F2 - Processing parameters
SI                   65536
SF             100.6127585 MHz
WDW                     EM
SSB                      0
LB                    1.00 Hz
GB                       0
PC                    1.40
```

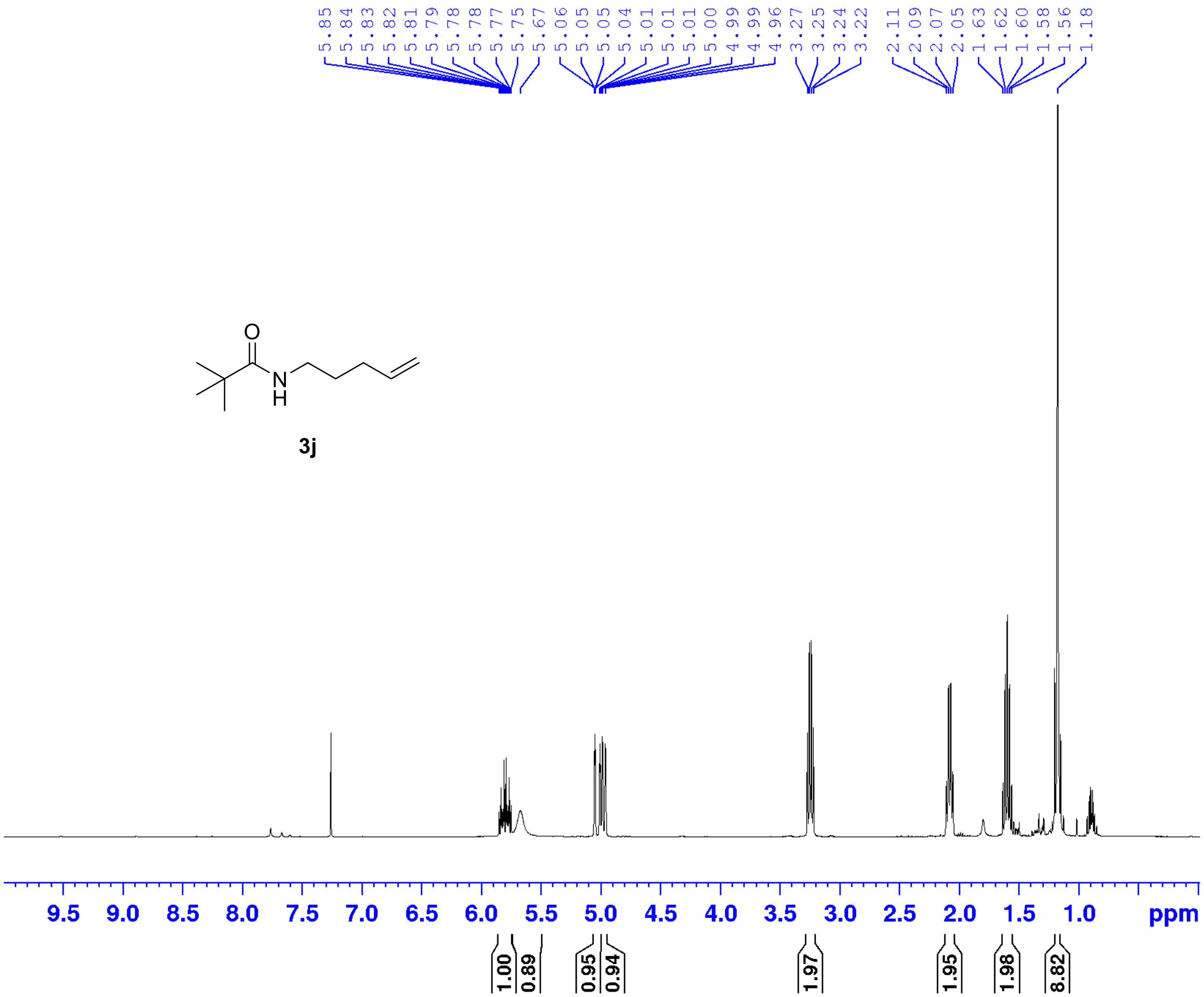

Current Data Parameters
NAME KK-495
EXPNO 40
PROCNO 1

F2 - Acquisition Parameters
Date_ 20221019
Time 10.30 h
INSTRUM spect
PROBHD Z116098_0048 (
PULPROG zg30
TD 65536
SOLVENT CDCl3
NS 16
DS 2
SWH 8223.685 Hz
FIDRES 0.250967 Hz
AQ 3.9845889 sec
RG 50.36
DW 60.800 usec
DE 10.80 usec
TE 298.2 K
D1 2.00000000 sec
TD0 1
SFO1 400.1324710 MHz
NUC1 1H
P0 3.08 usec
P1 9.25 usec
PLW1 24.00000000 W

F2 - Processing parameters
SI 32768
SF 400.1300096 MHz
WDW EM
SSB 0
LB 0.30 Hz
GB 0
PC 1.50

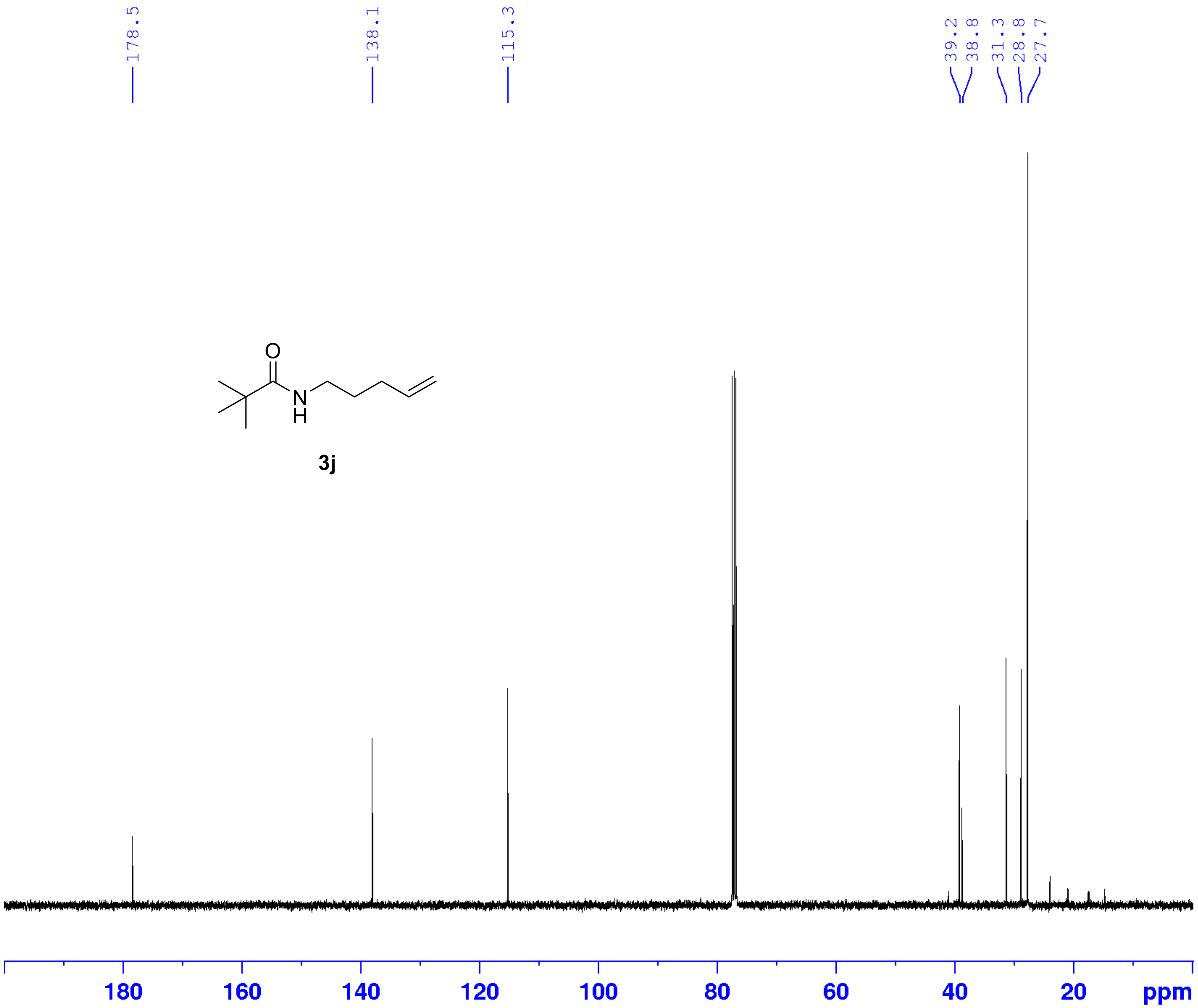

3j

Current Data Parameters

NAME	KK-495
EXPNO	41
PROCNO	1

F2 - Acquisition Parameters

Date_	20221019
Time	21.29 h
INSTRUM	spect
PROBHD	Z116098_0048 (
PULPROG	zgpg30
TD	65536
SOLVENT	CDCl3
NS	256
DS	4
SWH	24038.461 Hz
FIDRES	0.733596 Hz
AQ	1.3631488 sec
RG	181.72
DW	20.800 usec
DE	8.54 usec
TE	298.1 K
D1	2.00000000 sec
D11	0.03000000 sec
TD0	8
SFO1	100.6228303 MHz
NUC1	13C
P0	3.00 usec
P1	9.00 usec
PLW1	77.00000000 W
SFO2	400.1316005 MHz
NUC2	1H
CPDPRG[2	waltz16
PCPD2	90.00 usec
PLW2	24.00000000 W
PLW12	0.25352001 W
PLW13	0.12751999 W

F2 - Processing parameters

SI	65536
SF	100.6127569 MHz
WDW	EM
SSB	0
LB	1.00 Hz
GB	0
PC	1.40

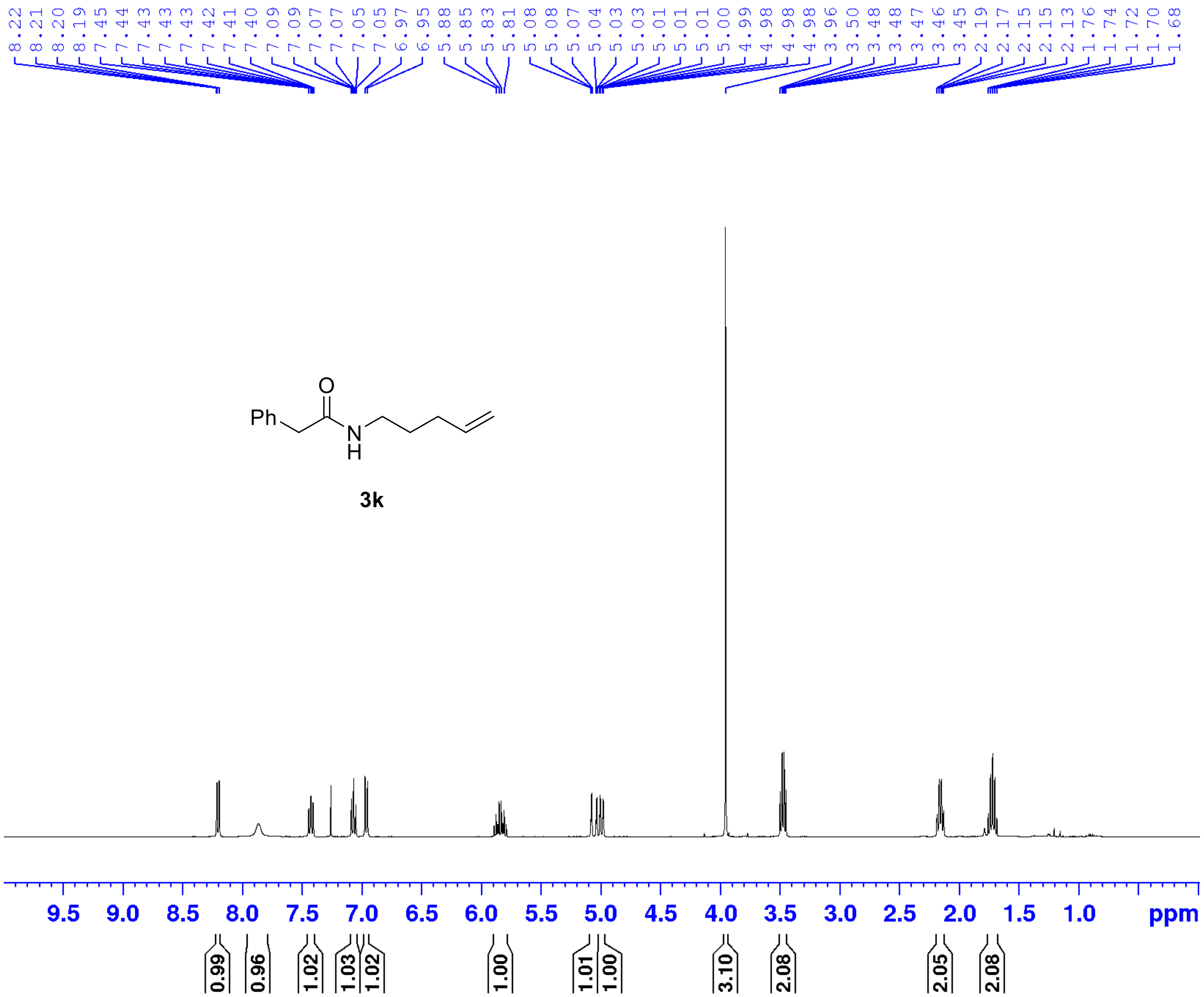

3k

```
Current Data Parameters
NAME               KK-582
EXPNO                  10
PROCNO                  1

F2 - Acquisition Parameters
Date_            20230713
Time                11.19 h
INSTRUM             spect
PROBHD    Z116098_0048 (
PULPROG              zg30
TD                  65536
SOLVENT             CDCl3
NS                     16
DS                      2
SWH              8223.685 Hz
FIDRES           0.250967 Hz
AQ              3.9845889 sec
RG                  50.36
DW                 60.800 usec
DE                  10.80 usec
TE                  298.2 K
D1             2.00000000 sec
TD0                     1
SFO1          400.1324710 MHz
NUC1                   1H
P0                   3.08 usec
P1                   9.25 usec
PLW1          24.00000000 W

F2 - Processing parameters
SI                  32768
SF            400.1300101 MHz
WDW                    EM
SSB                     0
LB                   0.30 Hz
GB                      0
PC                   1.50
```

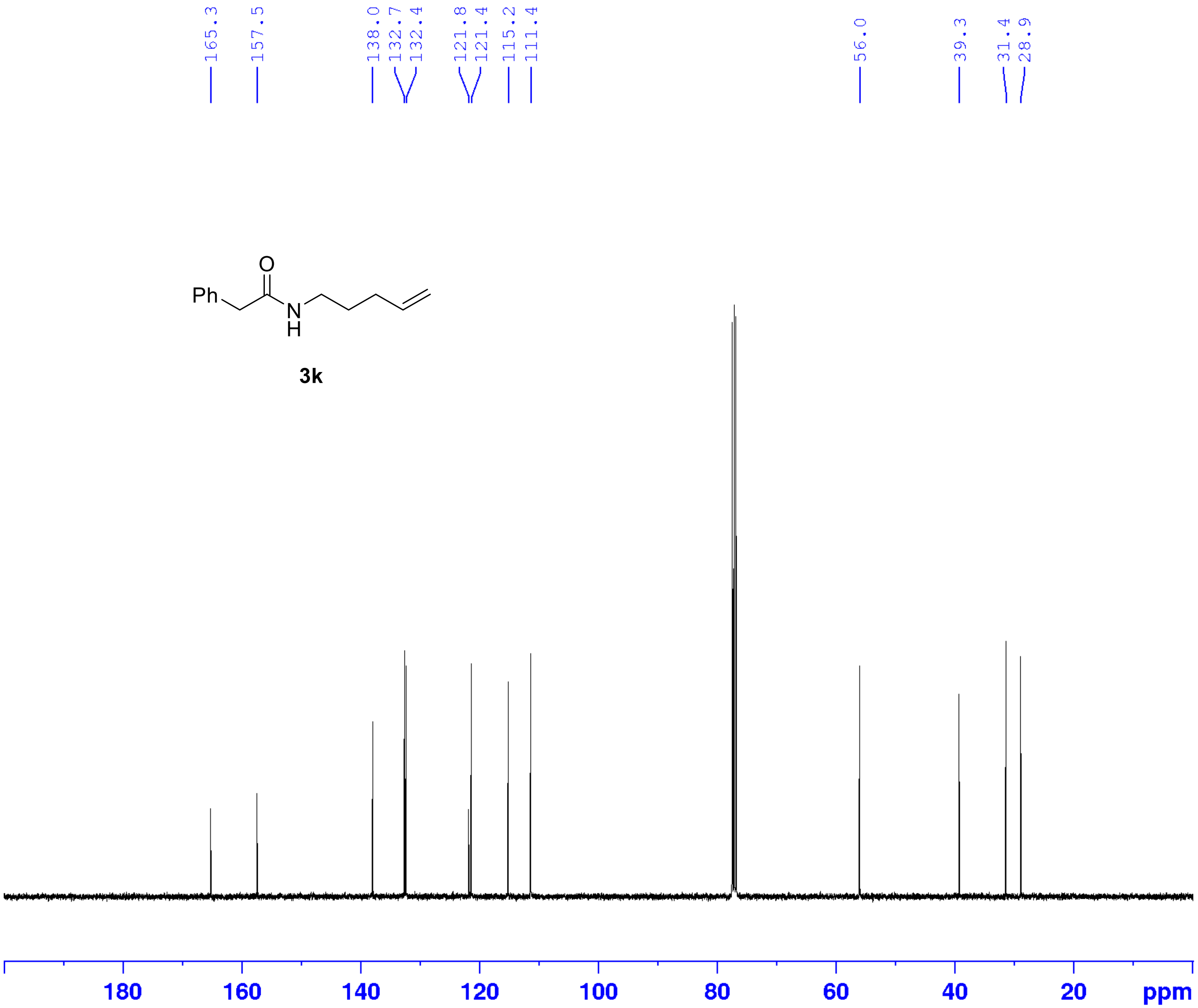

Current Data Parameters

NAME	KK-582
EXPNO	12
PROCNO	1

F2 - Acquisition Parameters

Date_	20230713
Time	19.19 h
INSTRUM	spect
PROBHD	Z116098_0048 (
PULPROG	zgpg30
TD	65536
SOLVENT	CDCl3
NS	512
DS	4
SWH	24038.461 Hz
FIDRES	0.733596 Hz
AQ	1.3631488 sec
RG	181.72
DW	20.800 usec
DE	8.54 usec
TE	298.2 K
D1	2.00000000 sec
D11	0.03000000 sec
TD0	8
SFO1	100.6228303 MHz
NUC1	13C
P0	3.00 usec
P1	9.00 usec
PLW1	77.00000000 W
SFO2	400.1316005 MHz
NUC2	1H
CPDPRG[2	waltz16
PCPD2	90.00 usec
PLW2	24.00000000 W
PLW12	0.25352001 W
PLW13	0.12751999 W

F2 - Processing parameters

SI	65536
SF	100.6127585 MHz
WDW	EM
SSB	0
LB	1.00 Hz
GB	0
PC	1.40

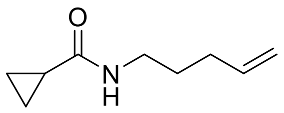

3m

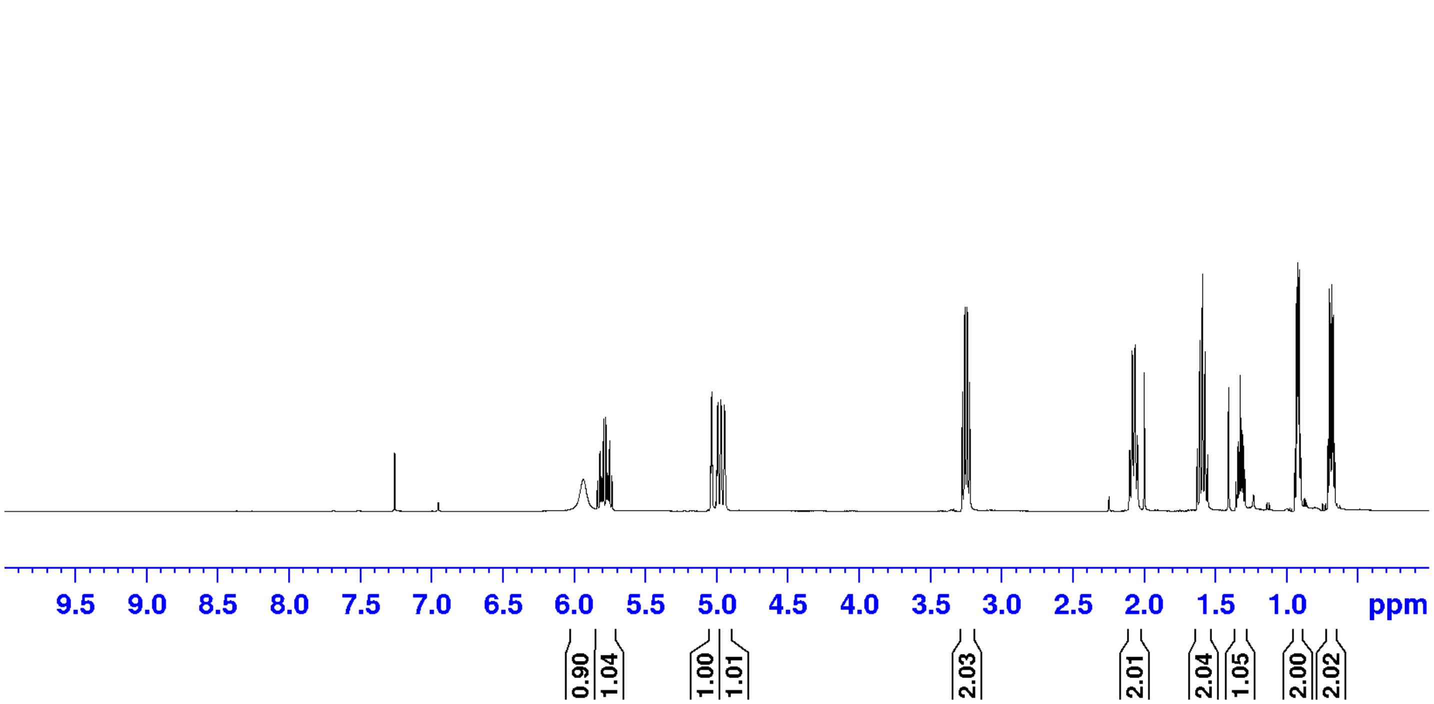

Current Data Parameters
NAME KK-498
EXPNO 30
PROCNO 1

F2 - Acquisition Parameters
Date_ 20240320
Time 11.04 h
INSTRUM spect
PROBHD Z116098_0048 (
PULPROG zg30
TD 65536
SOLVENT CDCl3
NS 16
DS 2
SWH 8223.685 Hz
FIDRES 0.250967 Hz
AQ 3.9845889 sec
RG 35.7
DW 60.800 usec
DE 10.80 usec
TE 298.1 K
D1 2.00000000 sec
TD0 1
SFO1 400.1324710 MHz
NUC1 1H
P0 3.08 usec
P1 9.25 usec
PLW1 24.00000000 W

F2 - Processing parameters
SI 32768
SF 400.1300104 MHz
WDW EM
SSB 0
LB 0.30 Hz
GB 0
PC 1.50

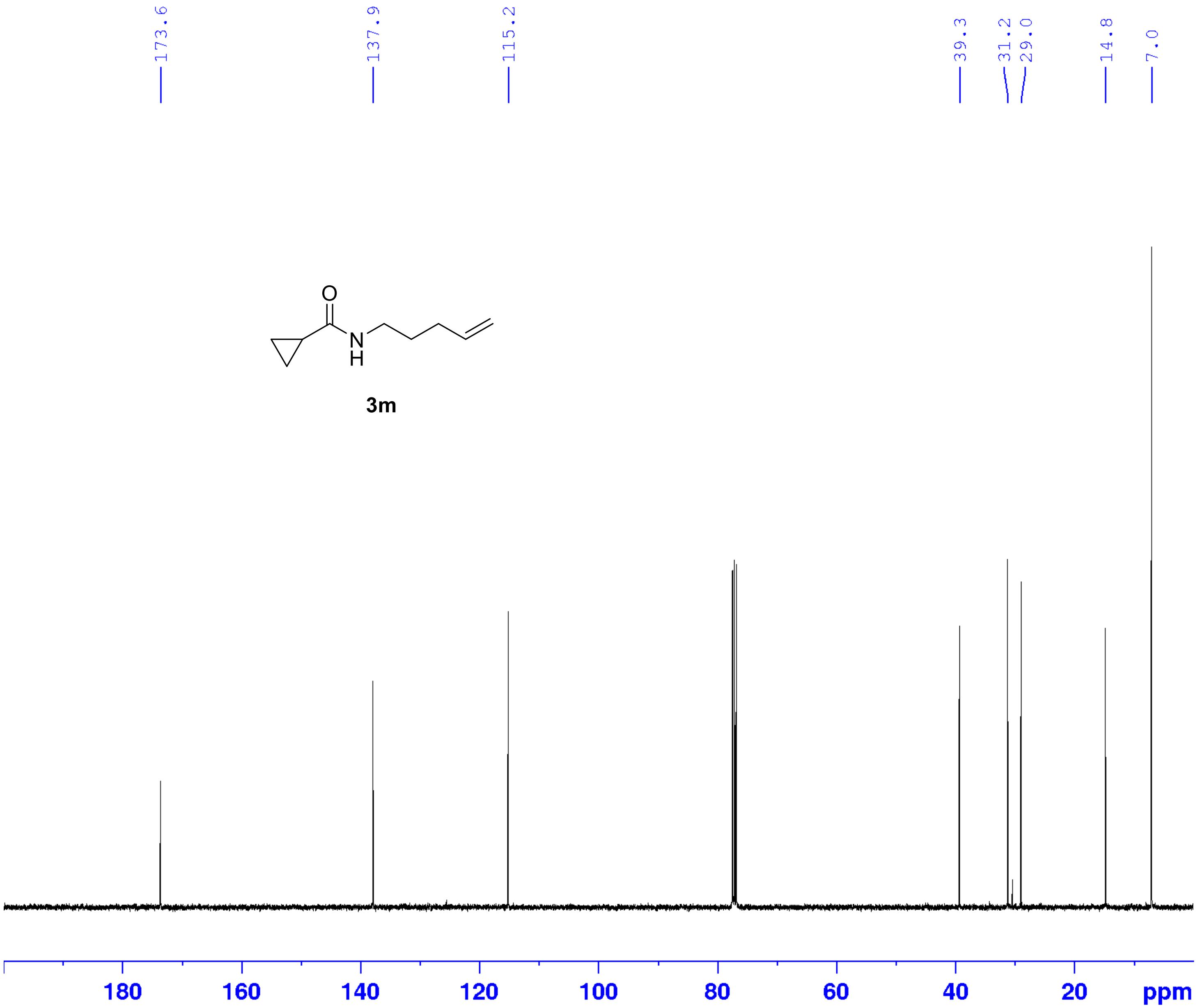

Current Data Parameters
NAME KK-498
EXPNO 31
PROCNO 1

F2 - Acquisition Parameters
Date_ 20240320
Time 21.56 h
INSTRUM spect
PROBHD Z116098_0048 (
PULPROG zgpg30
TD 65536
SOLVENT CDCl3
NS 256
DS 4
SWH 24038.461 Hz
FIDRES 0.733596 Hz
AQ 1.3631488 sec
RG 181.72
DW 20.800 usec
DE 8.54 usec
TE 298.1 K
D1 2.00000000 sec
D11 0.03000000 sec
TD0 8
SFO1 100.6228303 MHz
NUC1 13C
P0 3.00 usec
P1 9.00 usec
PLW1 77.00000000 W
SFO2 400.1316005 MHz
NUC2 1H
CPDPRG[2 waltz16
PCPD2 90.00 usec
PLW2 24.00000000 W
PLW12 0.25352001 W
PLW13 0.12751999 W

F2 - Processing parameters
SI 65536
SF 100.6127597 MHz
WDW EM
SSB 0
LB 1.00 Hz
GB 0
PC 1.40

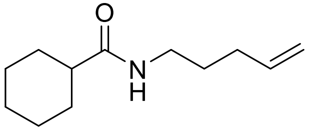

3n

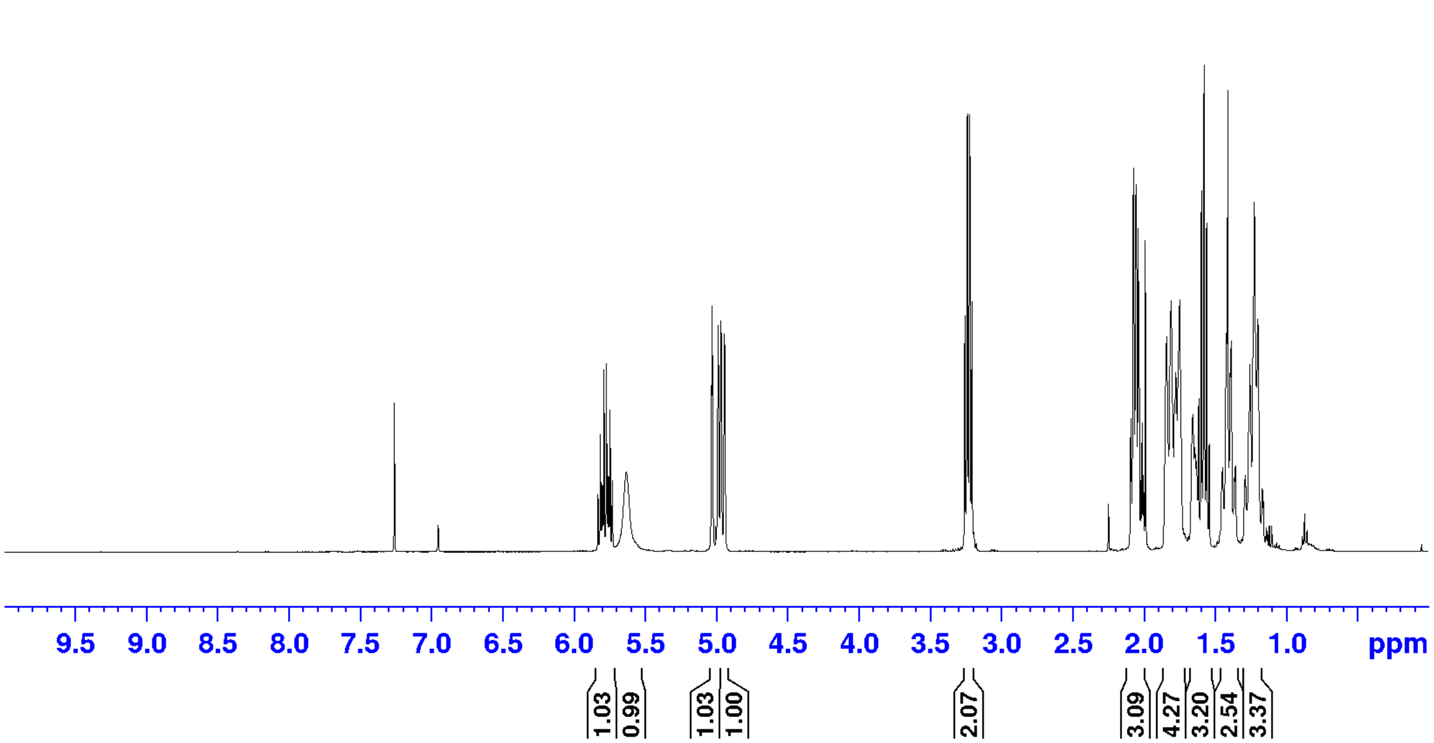


```
Current Data Parameters
NAME                 KK-560
EXPNO                    30
PROCNO                    1

F2 - Acquisition Parameters
Date_              20240320
Time                  12.55 h
INSTRUM               spect
PROBHD    Z116098_0048 (
PULPROG                zg30
TD                    65536
SOLVENT               CDCl3
NS                       16
DS                        2
SWH                8223.685 Hz
FIDRES             0.250967 Hz
AQ                3.9845889 sec
RG                     35.7
DW                   60.800 usec
DE                    10.80 usec
TE                    298.1 K
D1               2.00000000 sec
TD0                       1
SFO1            400.1324710 MHz
NUC1                     1H
P0                     3.08 usec
P1                     9.25 usec
PLW1            24.00000000 W

F2 - Processing parameters
SI                    32768
SF              400.1300103 MHz
WDW                      EM
SSB                       0
LB                     0.30 Hz
GB                        0
PC                     1.50
```

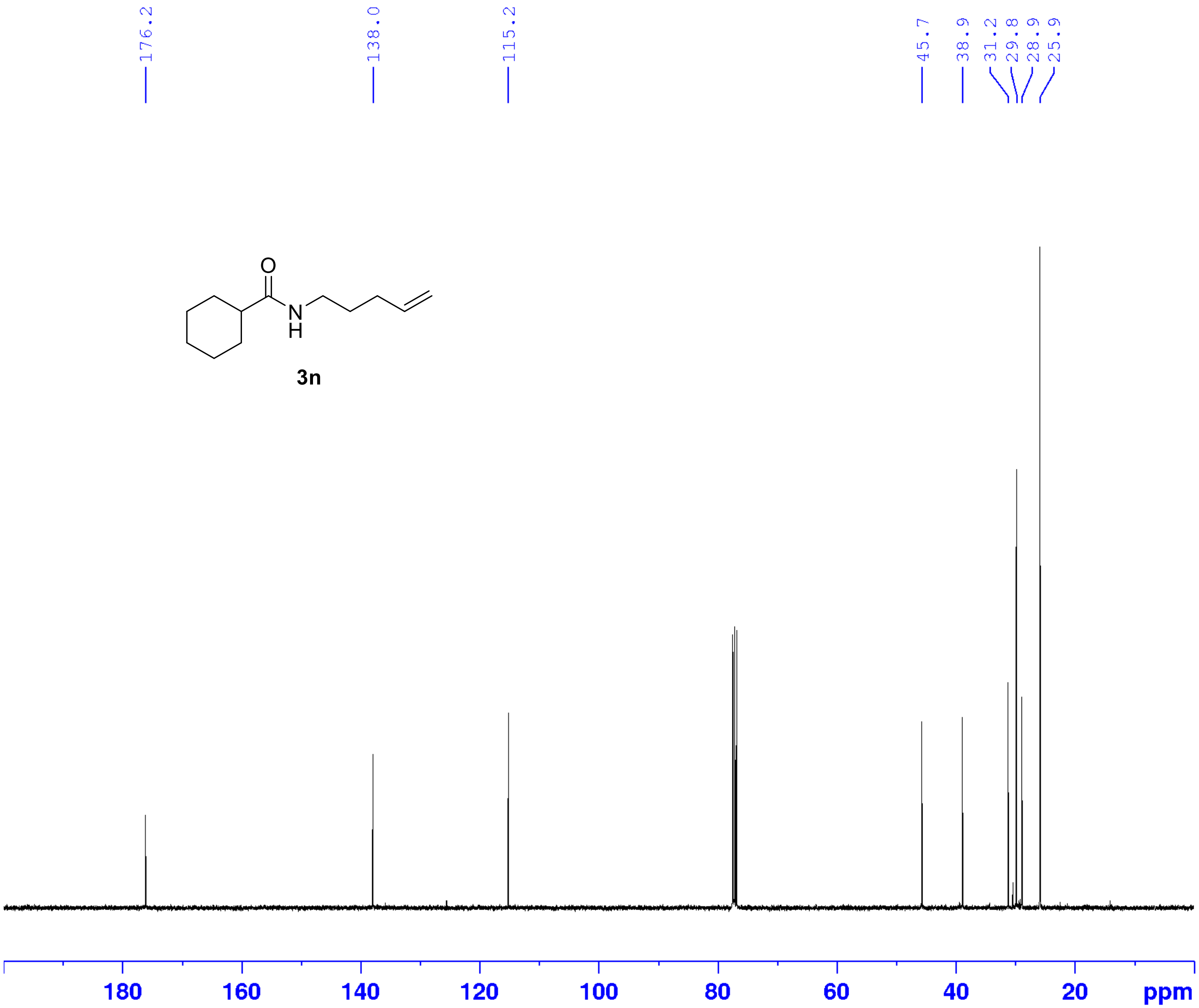

3n

```
Current Data Parameters
NAME              KK-560
EXPNO                 31
PROCNO                 1

F2 - Acquisition Parameters
Date_           20240320
Time               22.13 h
INSTRUM            spect
PROBHD    Z116098_0048 (
PULPROG          zgpg30
TD                 65536
SOLVENT            CDCl3
NS                   256
DS                     4
SWH            24038.461 Hz
FIDRES          0.733596 Hz
AQ             1.3631488 sec
RG                181.72
DW                20.800 usec
DE                  8.54 usec
TE                 298.2 K
D1            2.00000000 sec
D11           0.03000000 sec
TD0                    8
SFO1         100.6228303 MHz
NUC1                 13C
P0                  3.00 usec
P1                  9.00 usec
PLW1         77.00000000 W
SFO2         400.1316005 MHz
NUC2                  1H
CPDPRG[2         waltz16
PCPD2              90.00 usec
PLW2         24.00000000 W
PLW12         0.25352001 W
PLW13         0.12751999 W

F2 - Processing parameters
SI                 65536
SF           100.6127592 MHz
WDW                   EM
SSB                    0
LB                  1.00 Hz
GB                     0
PC                  1.40
```

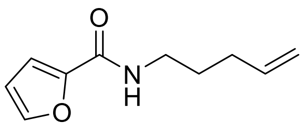

3o

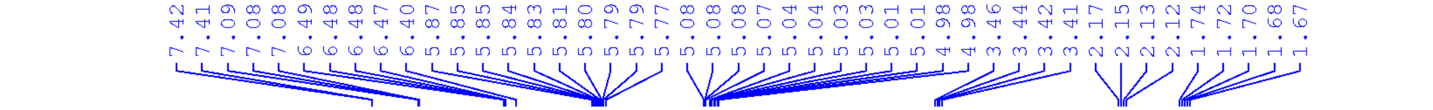

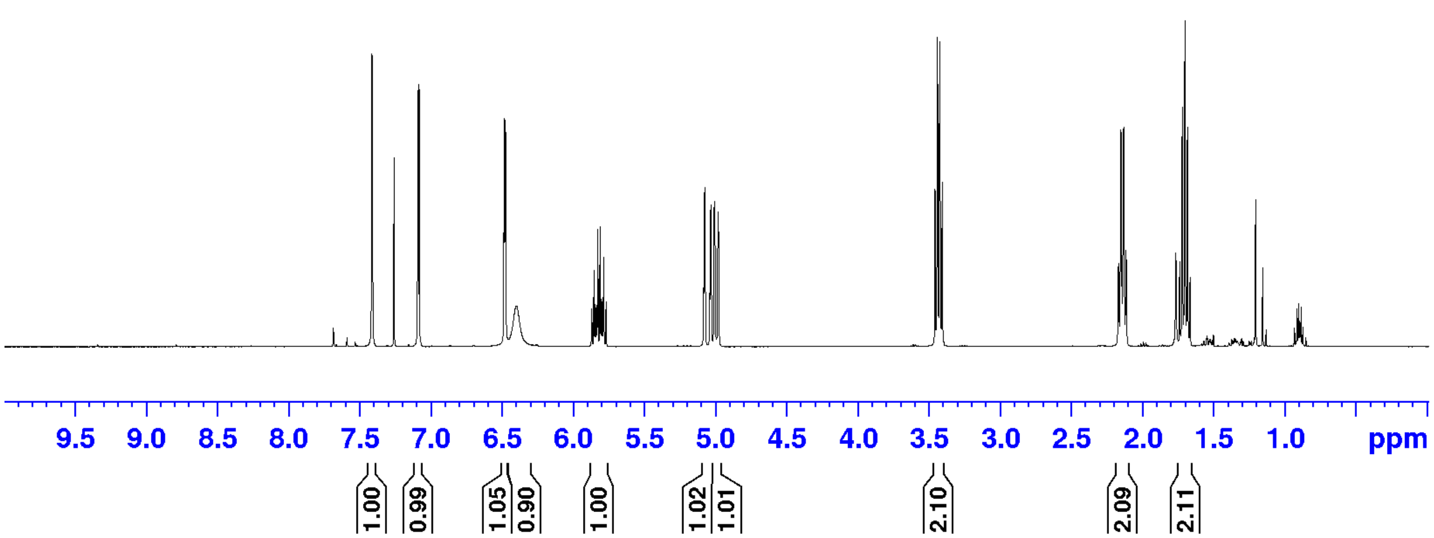

Current Data Parameters

NAME	KK-481
EXPNO	30
PROCNO	1

F2 - Acquisition Parameters

Date_	20221019
Time	10.40 h
INSTRUM	spect
PROBHD	Z116098_0048 (
PULPROG	zg30
TD	65536
SOLVENT	CDCl3
NS	16
DS	2
SWH	8223.685 Hz
FIDRES	0.250967 Hz
AQ	3.9845889 sec
RG	92.46
DW	60.800 usec
DE	10.80 usec
TE	298.1 K
D1	2.00000000 sec
TD0	1
SFO1	400.1324710 MHz
NUC1	1H
P0	3.08 usec
P1	9.25 usec
PLW1	24.00000000 W

F2 - Processing parameters

SI	32768
SF	400.1300095 MHz
WDW	EM
SSB	0
LB	0.30 Hz
GB	0
PC	1.50

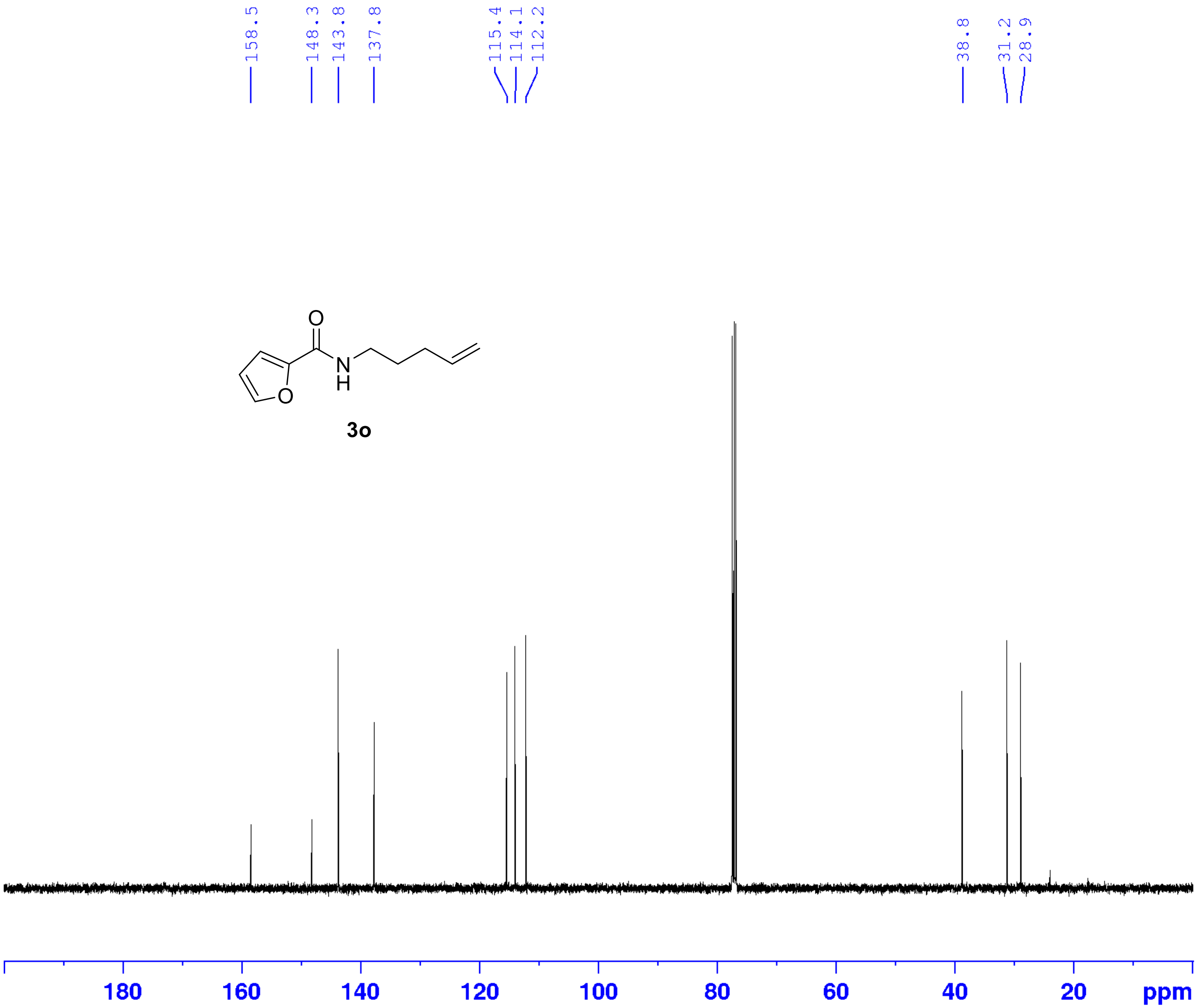

Current Data Parameters
NAME KK-481
EXPNO 31
PROCNO 1

F2 - Acquisition Parameters
Date_ 20221019
Time 23.04 h
INSTRUM spect
PROBHD Z116098_0048 (
PULPROG zgpg30
TD 65536
SOLVENT CDCl3
NS 256
DS 4
SWH 24038.461 Hz
FIDRES 0.733596 Hz
AQ 1.3631488 sec
RG 181.72
DW 20.800 usec
DE 8.54 usec
TE 298.1 K
D1 2.00000000 sec
D11 0.03000000 sec
TD0 8
SFO1 100.6228303 MHz
NUC1 13C
P0 3.00 usec
P1 9.00 usec
PLW1 77.00000000 W
SFO2 400.1316005 MHz
NUC2 1H
CPDPRG[2 waltz16
PCPD2 90.00 usec
PLW2 24.00000000 W
PLW12 0.25352001 W
PLW13 0.12751999 W

F2 - Processing parameters
SI 65536
SF 100.6127572 MHz
WDW EM
SSB 0
LB 1.00 Hz
GB 0
PC 1.40

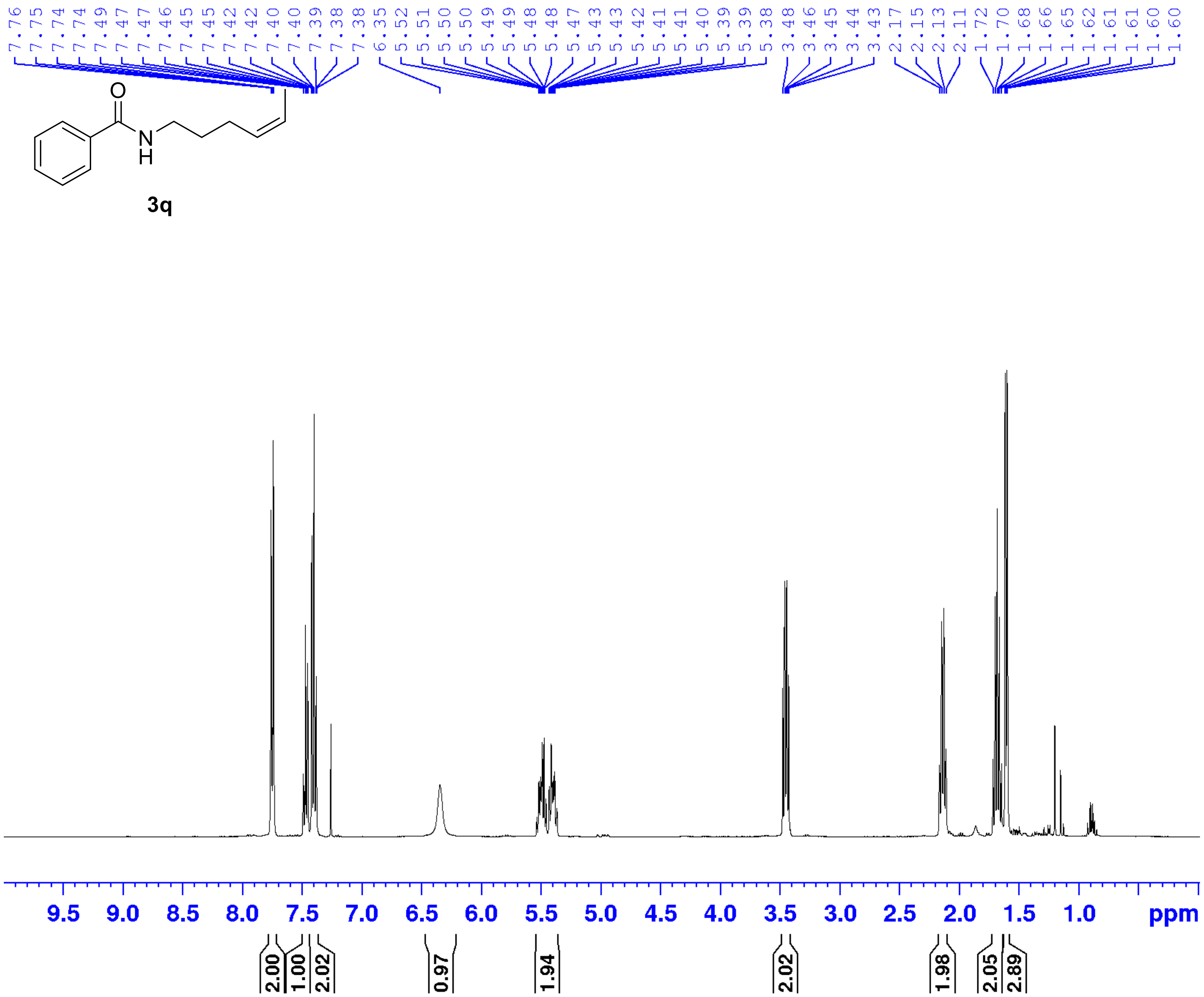

BRUKER

Current Data Parameters
NAME KK-513
EXPNO 10
PROCNO 1

F2 - Acquisition Parameters
Date_ 20221106
Time 13.39 h
INSTRUM spect
PROBHD Z116098_0048 (
PULPROG zg30
TD 65536
SOLVENT CDCl3
NS 16
DS 2
SWH 8223.685 Hz
FIDRES 0.250967 Hz
AQ 3.9845889 sec
RG 46.39
DW 60.800 usec
DE 10.80 usec
TE 298.1 K
D1 2.00000000 sec
TD0 1
SFO1 400.1324710 MHz
NUC1 1H
P0 3.08 usec
P1 9.25 usec
PLW1 24.00000000 W

F2 - Processing parameters
SI 32768
SF 400.1300097 MHz
WDW EM
SSB 0
LB 0.30 Hz
GB 0
PC 1.50

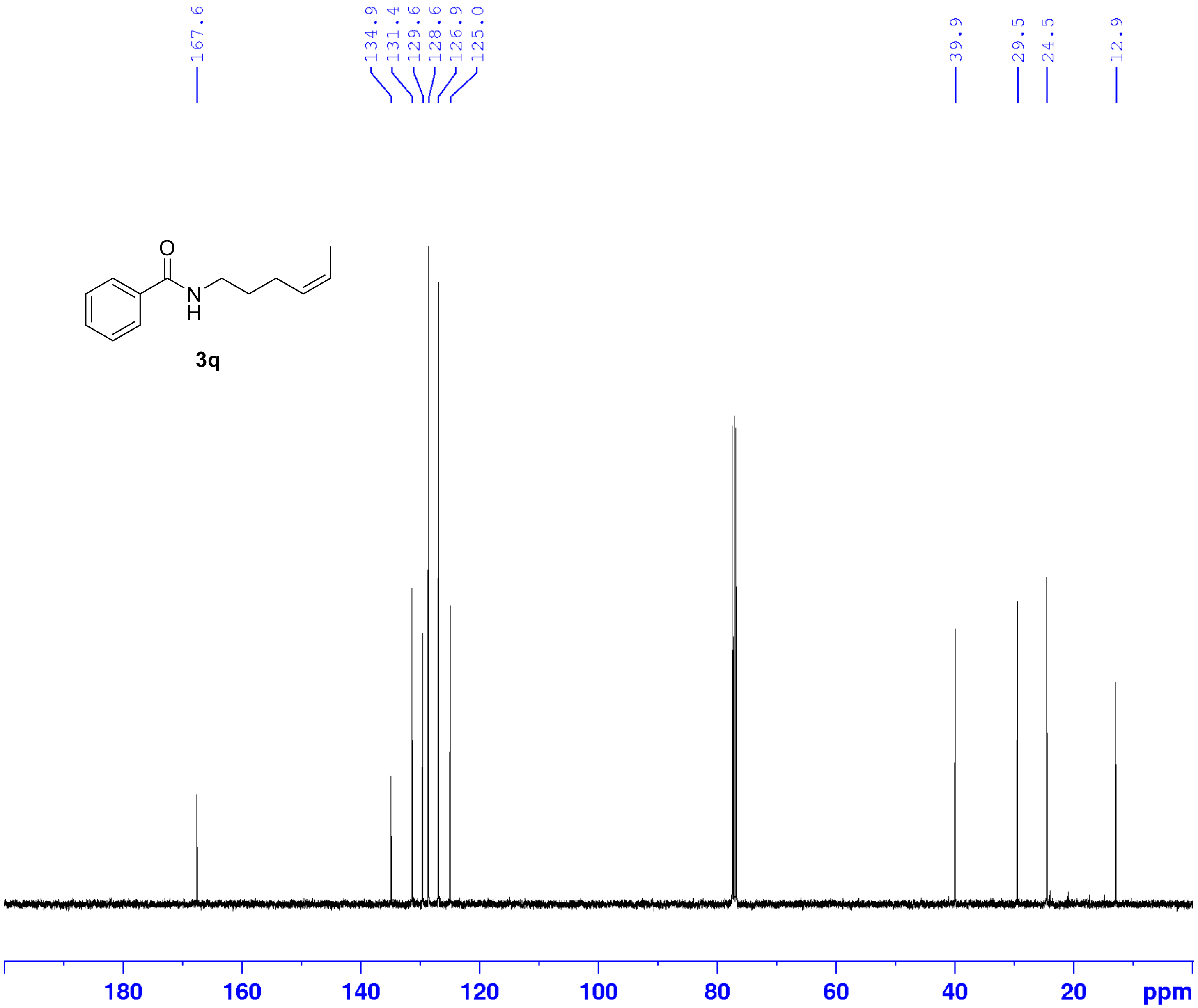


```
Current Data Parameters
NAME               KK-513
EXPNO                  11
PROCNO                  1

F2 - Acquisition Parameters
Date_            20221106
Time                14.49 h
INSTRUM             spect
PROBHD    Z116098_0048 (
PULPROG           zgpg30
TD                  65536
SOLVENT             CDCl3
NS                    256
DS                      4
SWH             24038.461 Hz
FIDRES           0.733596 Hz
AQ              1.3631488 sec
RG                 181.72
DW                 20.800 usec
DE                   8.54 usec
TE                  298.1 K
D1             2.00000000 sec
D11            0.03000000 sec
TD0                     8
SFO1          100.6228303 MHz
NUC1                  13C
P0                   3.00 usec
P1                   9.00 usec
PLW1          77.00000000 W
SFO2          400.1316005 MHz
NUC2                   1H
CPDPRG[2          waltz16
PCPD2               90.00 usec
PLW2          24.00000000 W
PLW12          0.25352001 W
PLW13          0.12751999 W

F2 - Processing parameters
SI                  65536
SF            100.6127599 MHz
WDW                    EM
SSB                     0
LB                   1.00 Hz
GB                      0
PC                   1.40
```

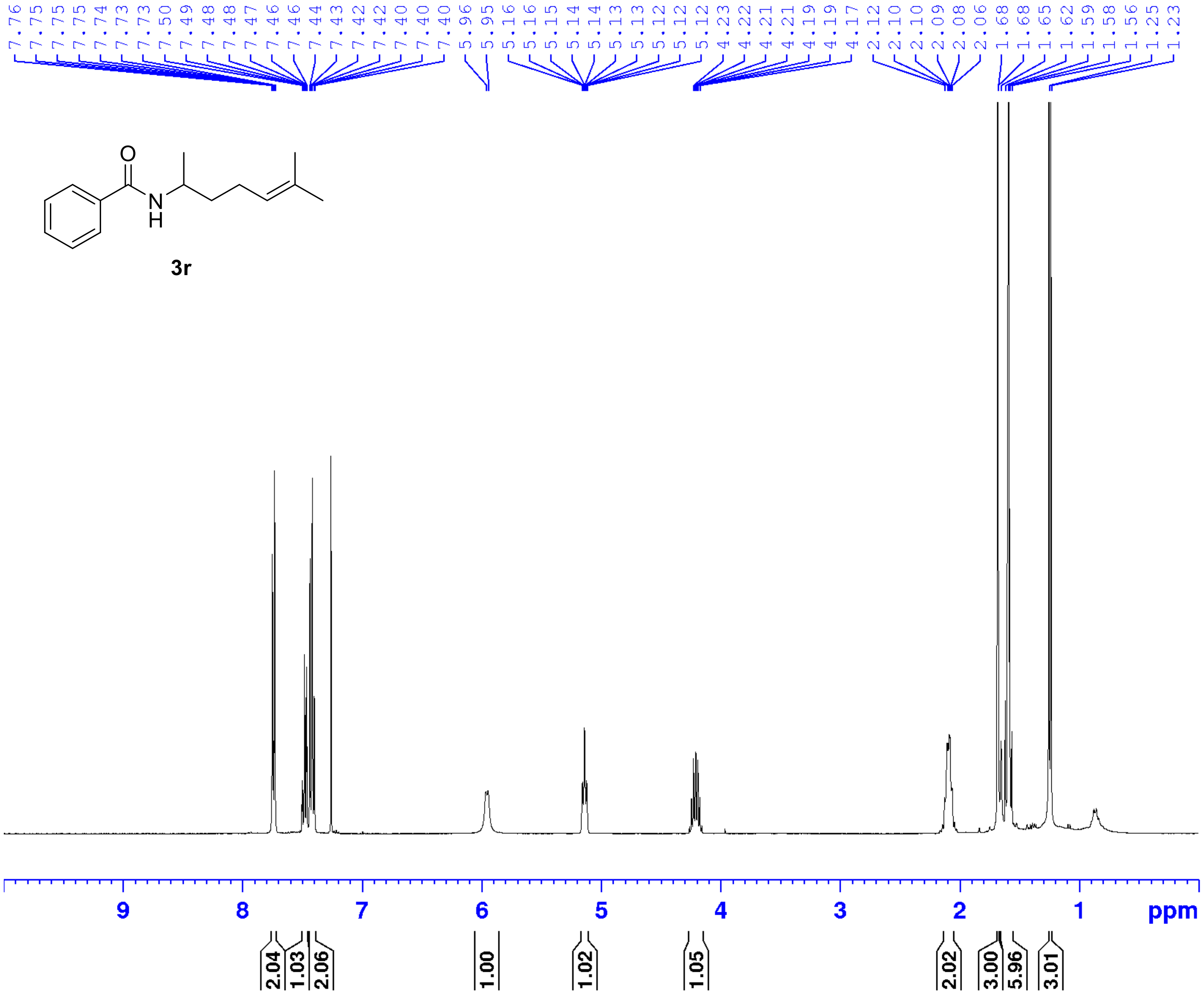

Current Data Parameters
NAME KK-589
EXPNO 10
PROCNO 1

F2 - Acquisition Parameters
Date_ 20230719
Time 12.06 h
INSTRUM spect
PROBHD Z116098_0048 (
PULPROG zg30
TD 65536
SOLVENT CDCl3
NS 16
DS 2
SWH 8223.685 Hz
FIDRES 0.250967 Hz
AQ 3.9845889 sec
RG 92.46
DW 60.800 usec
DE 10.80 usec
TE 298.1 K
D1 2.00000000 sec
TD0 1
SFO1 400.1324710 MHz
NUC1 1H
P0 3.08 usec
P1 9.25 usec
PLW1 24.00000000 W

F2 - Processing parameters
SI 32768
SF 400.1300101 MHz
WDW EM
SSB 0
LB 0.30 Hz
GB 0
PC 1.50

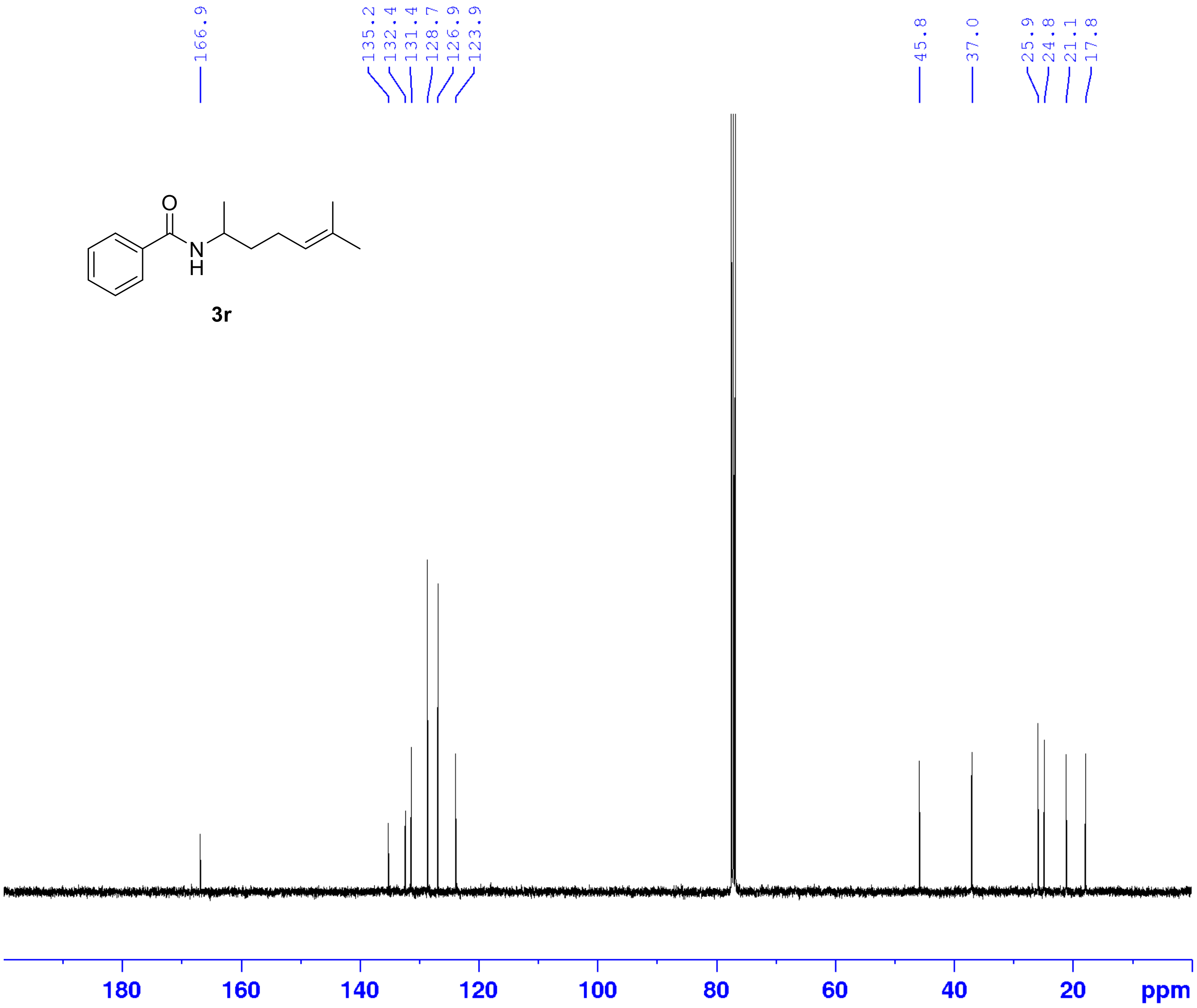

3r

Current Data Parameters
NAME KK-589
EXPNO 12
PROCNO 1

F2 - Acquisition Parameters
Date_ 20230719
Time 19.19 h
INSTRUM spect
PROBHD Z116098_0048 (
PULPROG zgpg30
TD 65536
SOLVENT CDCl3
NS 512
DS 4
SWH 24038.461 Hz
FIDRES 0.733596 Hz
AQ 1.3631488 sec
RG 181.72
DW 20.800 usec
DE 8.54 usec
TE 298.1 K
D1 2.00000000 sec
D11 0.03000000 sec
TD0 8
SFO1 100.6228303 MHz
NUC1 13C
P0 3.00 usec
P1 9.00 usec
PLW1 77.00000000 W
SFO2 400.1316005 MHz
NUC2 1H
CPDPRG[2 waltz16
PCPD2 90.00 usec
PLW2 24.00000000 W
PLW12 0.25352001 W
PLW13 0.12751999 W

F2 - Processing parameters
SI 65536
SF 100.6127561 MHz
WDW EM
SSB 0
LB 1.00 Hz
GB 0
PC 1.40

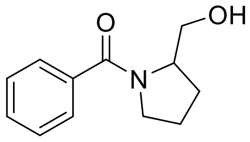

7a

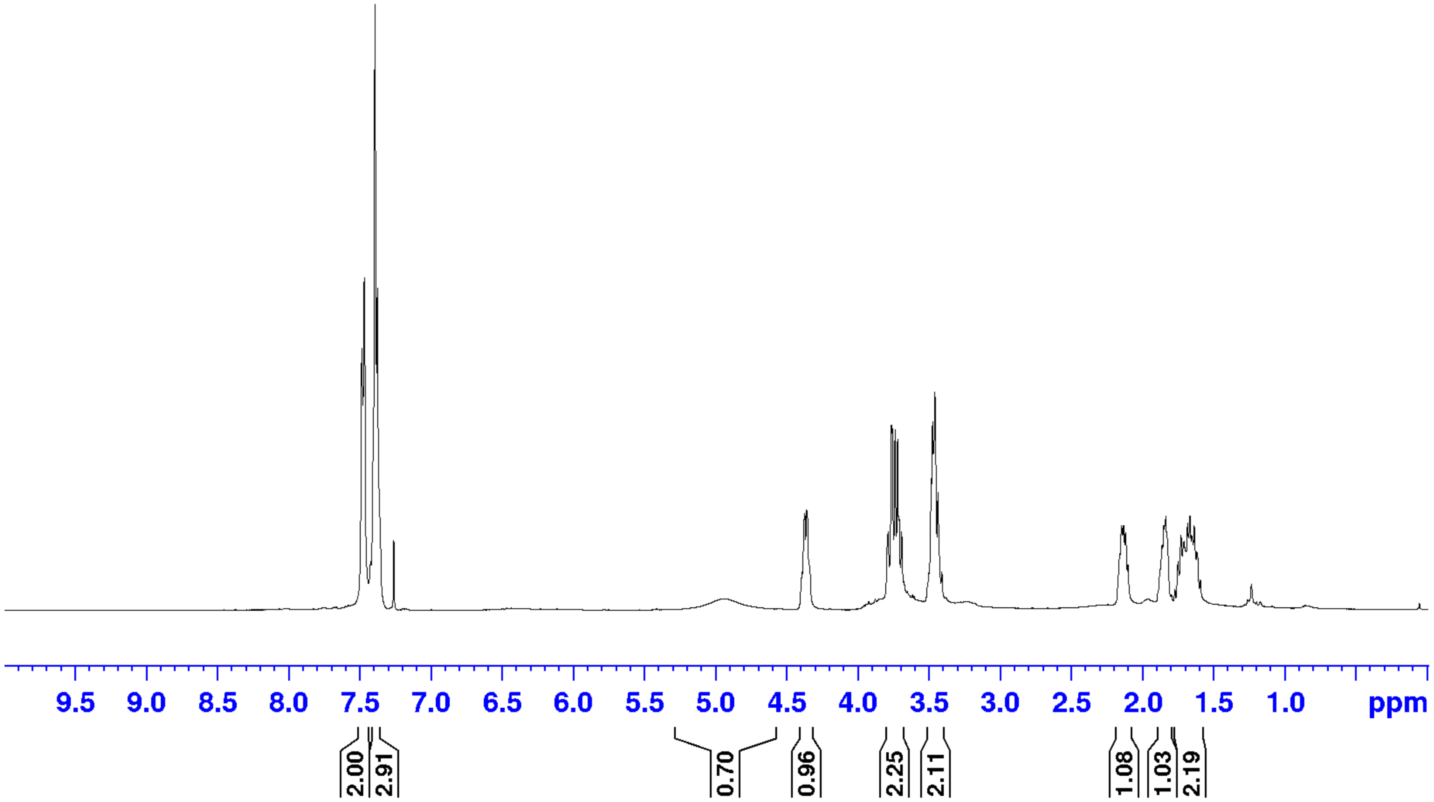

BRUKER

Current Data Parameters

NAME	KK-584
EXPNO	10
PROCNO	1

F2 - Acquisition Parameters

Date_	20230711
Time	12.13 h
INSTRUM	spect
PROBHD	Z116098_0048 (
PULPROG	zg30
TD	65536
SOLVENT	CDCl3
NS	16
DS	2
SWH	8223.685 Hz
FIDRES	0.250967 Hz
AQ	3.9845889 sec
RG	35.7
DW	60.800 usec
DE	10.80 usec
TE	298.1 K
D1	2.00000000 sec
TD0	1
SFO1	400.1324710 MHz
NUC1	1H
P0	3.08 usec
P1	9.25 usec
PLW1	24.00000000 W

F2 - Processing parameters

SI	32768
SF	400.1300102 MHz
WDW	EM
SSB	0
LB	0.30 Hz
GB	0
PC	1.50

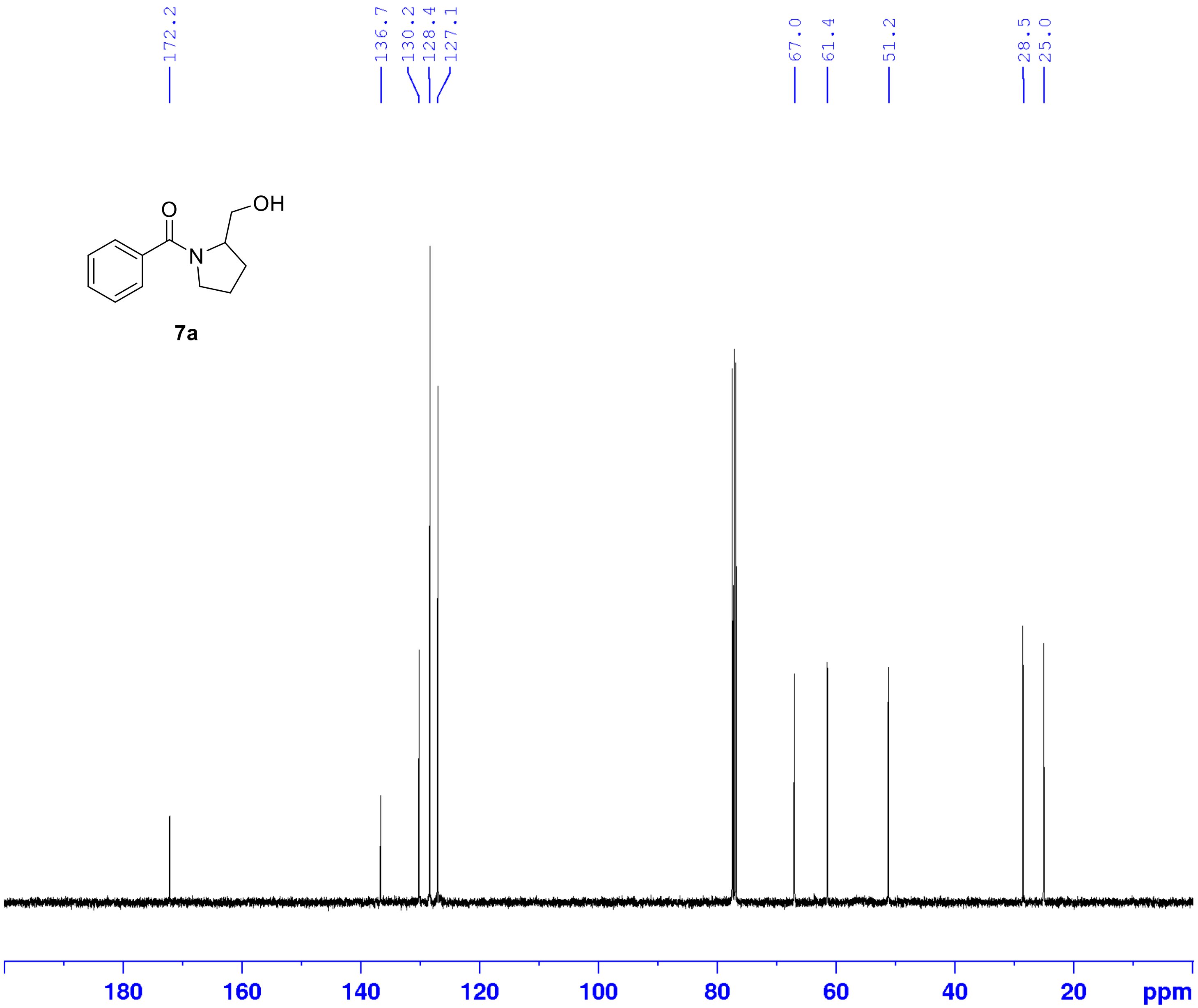

7a


```
Current Data Parameters
NAME                KK-584
EXPNO                   20
PROCNO                   1

F2 - Acquisition Parameters
Date_             20230711
Time                 19.38 h
INSTRUM              spect
PROBHD    Z116098_0048 (
PULPROG            zgpg30
TD                  65536
SOLVENT             CDCl3
NS                    256
DS                      4
SWH             24038.461 Hz
FIDRES           0.733596 Hz
AQ              1.3631488 sec
RG                 181.72
DW                 20.800 usec
DE                   8.54 usec
TE                  298.1 K
D1             2.00000000 sec
D11            0.03000000 sec
TD0                     8
SFO1          100.6228303 MHz
NUC1                  13C
P0                   3.00 usec
P1                   9.00 usec
PLW1          77.00000000 W
SFO2          400.1316005 MHz
NUC2                   1H
CPDPRG[2          waltz16
PCPD2               90.00 usec
PLW2          24.00000000 W
PLW12          0.25352001 W
PLW13          0.12751999 W

F2 - Processing parameters
SI                  65536
SF            100.6127646 MHz
WDW                    EM
SSB                     0
LB                   1.00 Hz
GB                      0
PC                   1.40
```

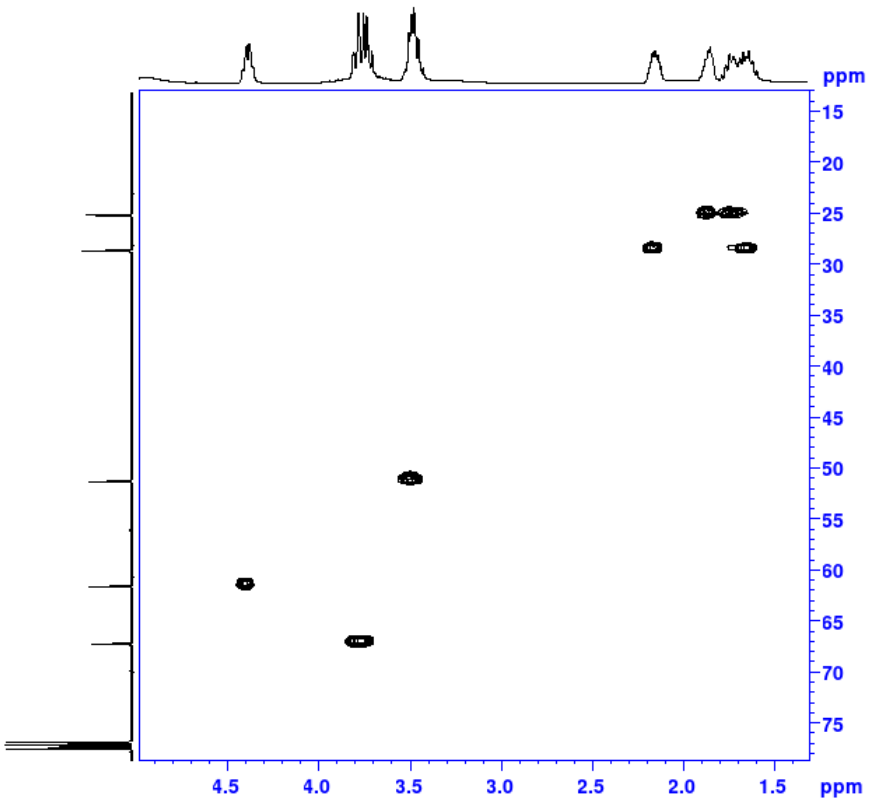
ppm
15
20
25
30
35
40
45
50
55
60
65
70
75
4.5
4.0
3.5
3.0
2.5
2.0
1.5
ppm

HSQC

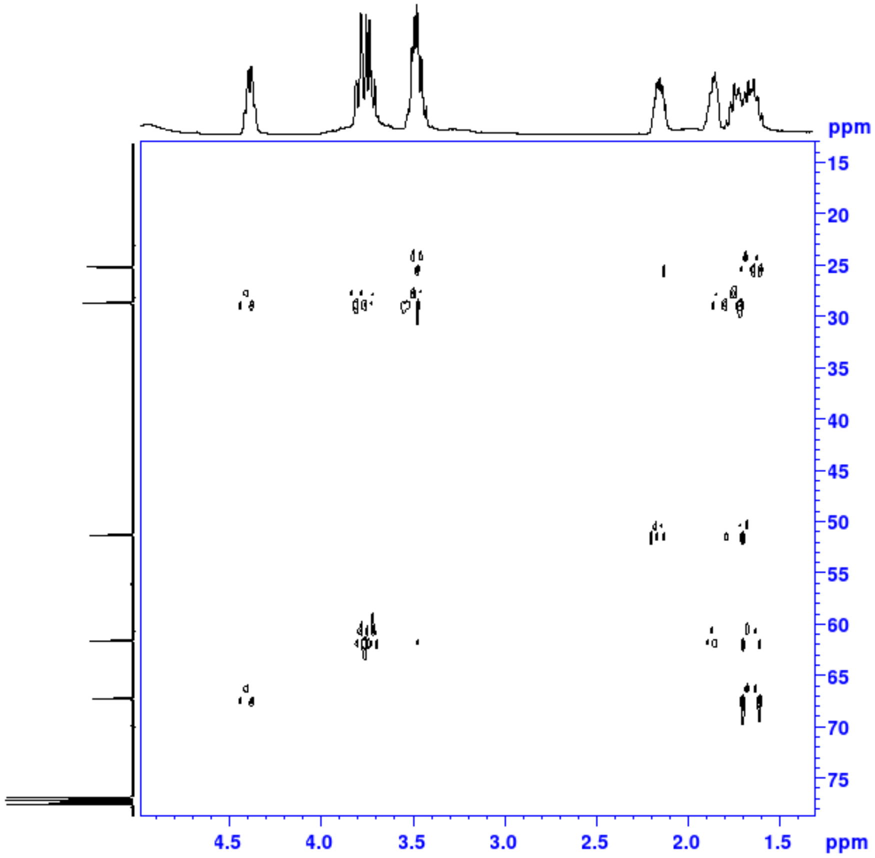
ppm
15
20
25
30
35
40
45
50
55
60
65
70
75
4.5
4.0
3.5
3.0
2.5
2.0
1.5
ppm

HMBC

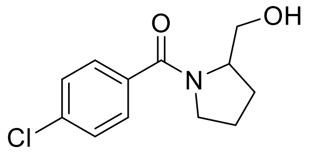

7b

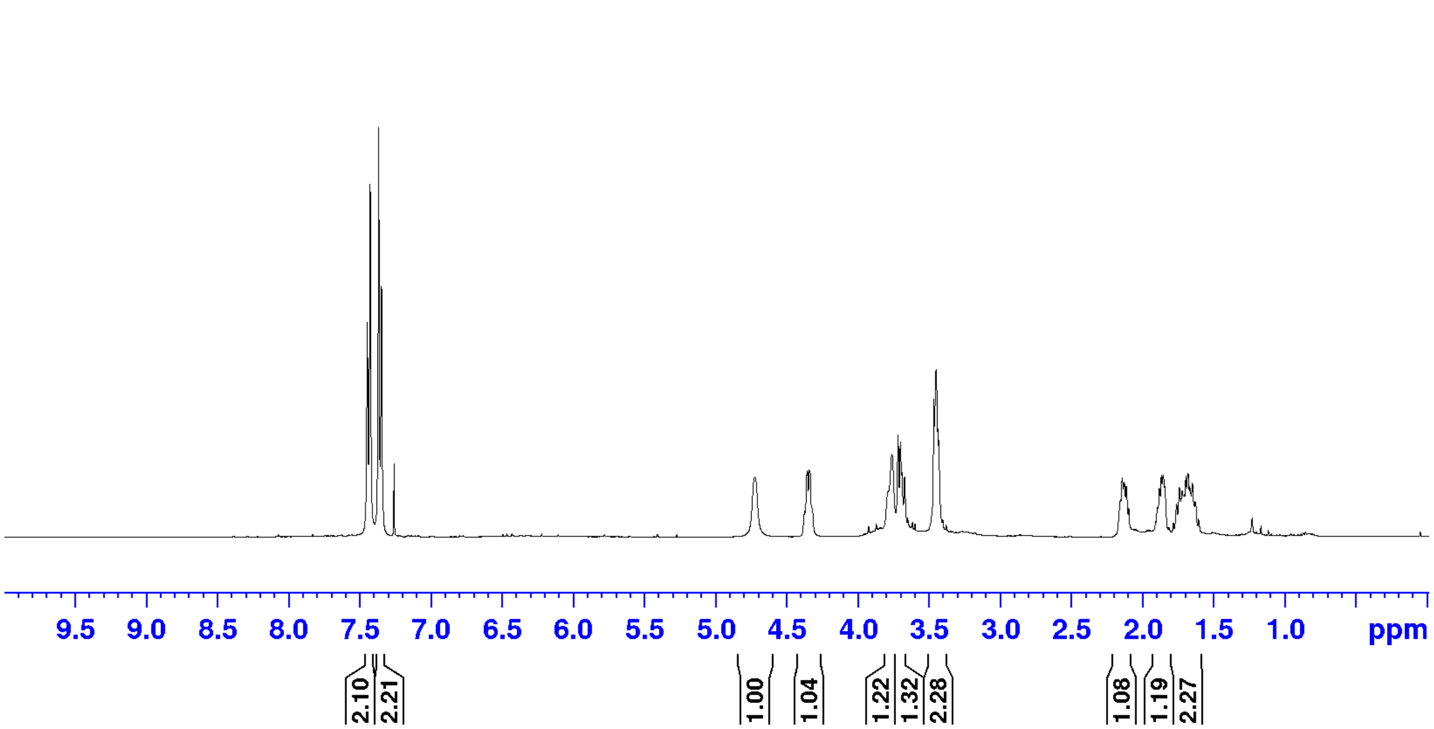


```
Current Data Parameters
NAME                KK-660
EXPNO                   20
PROCNO                   1

F2 - Acquisition Parameters
Date_             20240328
Time                 10.37 h
INSTRUM              spect
PROBHD    Z116098_0048 (
PULPROG               zg30
TD                   65536
SOLVENT              CDCl3
NS                      16
DS                       2
SWH               8223.685 Hz
FIDRES            0.250967 Hz
AQ               3.9845889 sec
RG                   40.16
DW                  60.800 usec
DE                   10.80 usec
TE                   298.1 K
D1              2.00000000 sec
TD0                      1
SFO1           400.1324710 MHz
NUC1                    1H
P0                    3.08 usec
P1                    9.25 usec
PLW1           24.00000000 W

F2 - Processing parameters
SI                   32768
SF             400.1300102 MHz
WDW                     EM
SSB                      0
LB                    0.30 Hz
GB                       0
PC                    1.50
```

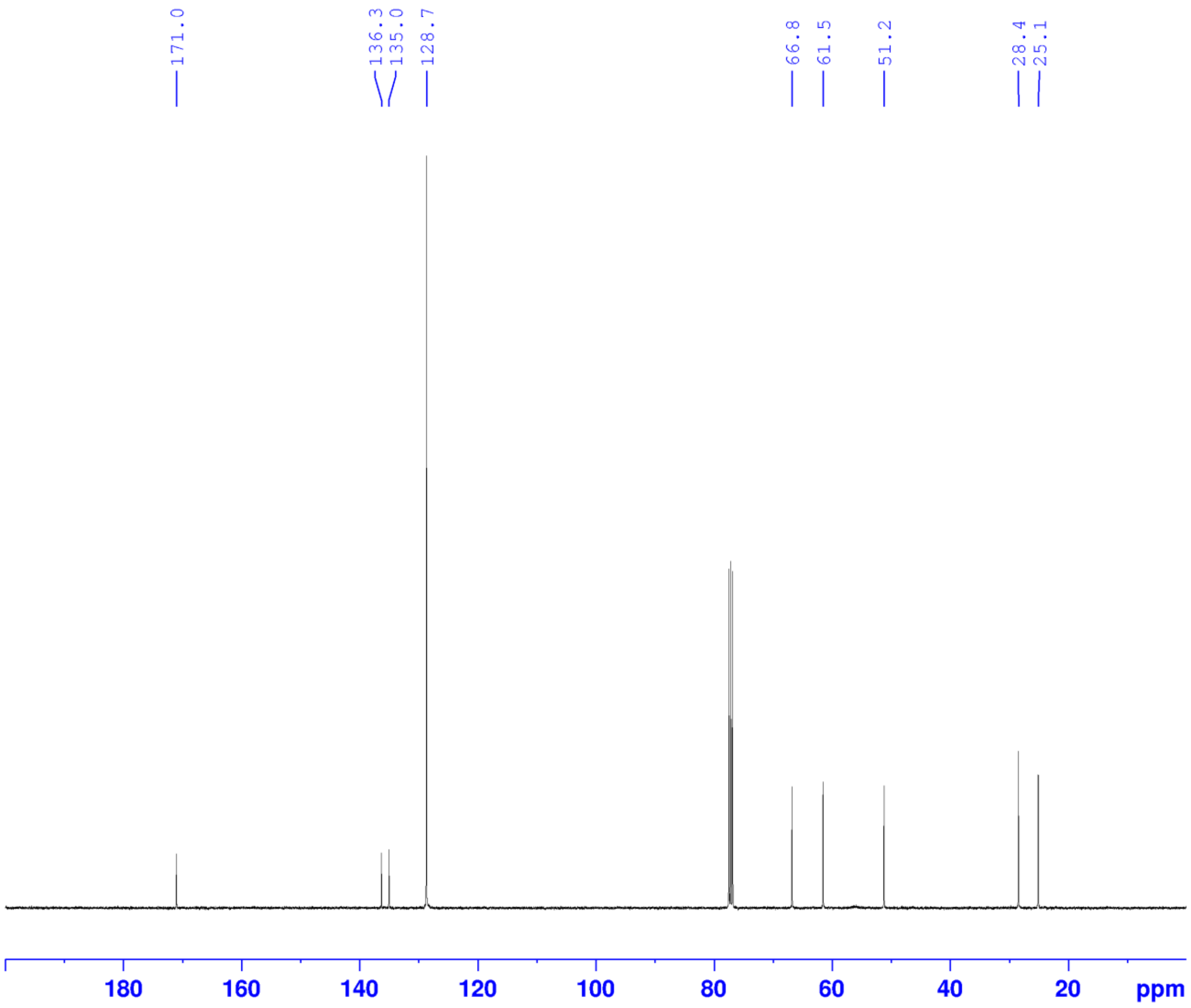

BRUKER

Current Data Parameters

NAME	KK-660
EXPNO	23
PROCNO	1

F2 - Acquisition Parameters

Date_	20240328
Time	21.15 h
INSTRUM	spect
PROBHD	Z116098_0048 (
PULPROG	zgpg30
TD	65536
SOLVENT	CDCl3
NS	1024
DS	4
SWH	24038.461 Hz
FIDRES	0.733596 Hz
AQ	1.3631488 sec
RG	181.72
DW	20.800 usec
DE	8.54 usec
TE	298.2 K
D1	2.00000000 sec
D11	0.03000000 sec
TD0	8
SFO1	100.6228303 MHz
NUC1	13C
P0	3.00 usec
P1	9.00 usec
PLW1	77.00000000 W
SFO2	400.1316005 MHz
NUC2	1H
CPDPRG[2	waltz16
PCPD2	90.00 usec
PLW2	24.00000000 W
PLW12	0.25352001 W
PLW13	0.12751999 W

F2 - Processing parameters

SI	65536
SF	100.6127635 MHz
WDW	EM
SSB	0
LB	1.00 Hz
GB	0
PC	1.40

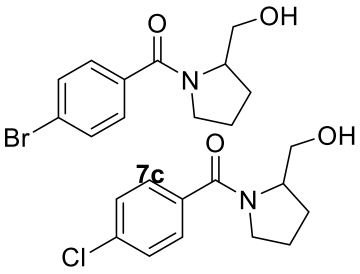

7c

7b

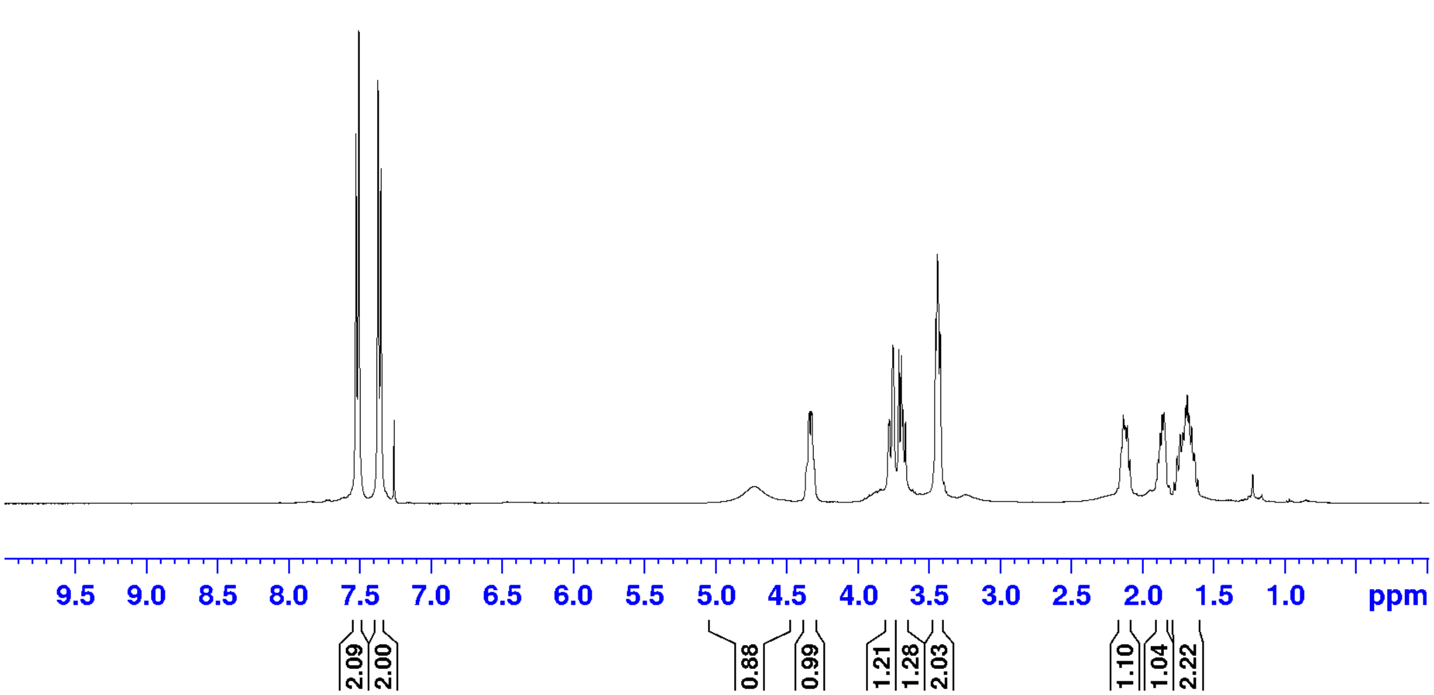

Current Data Parameters
NAME KK-607
EXPNO 10
PROCNO 1

F2 - Acquisition Parameters
Date_ 20230904
Time 14.33 h
INSTRUM spect
PROBHD Z116098_0048 (
PULPROG zg30
TD 65536
SOLVENT CDCl3
NS 16
DS 2
SWH 8223.685 Hz
FIDRES 0.250967 Hz
AQ 3.9845889 sec
RG 35.7
DW 60.800 usec
DE 10.80 usec
TE 298.2 K
D1 2.00000000 sec
TD0 1
SFO1 400.1324710 MHz
NUC1 1H
P0 3.08 usec
P1 9.25 usec
PLW1 24.00000000 W

F2 - Processing parameters
SI 32768
SF 400.1300102 MHz
WDW EM
SSB 0
LB 0.30 Hz
GB 0
PC 1.50

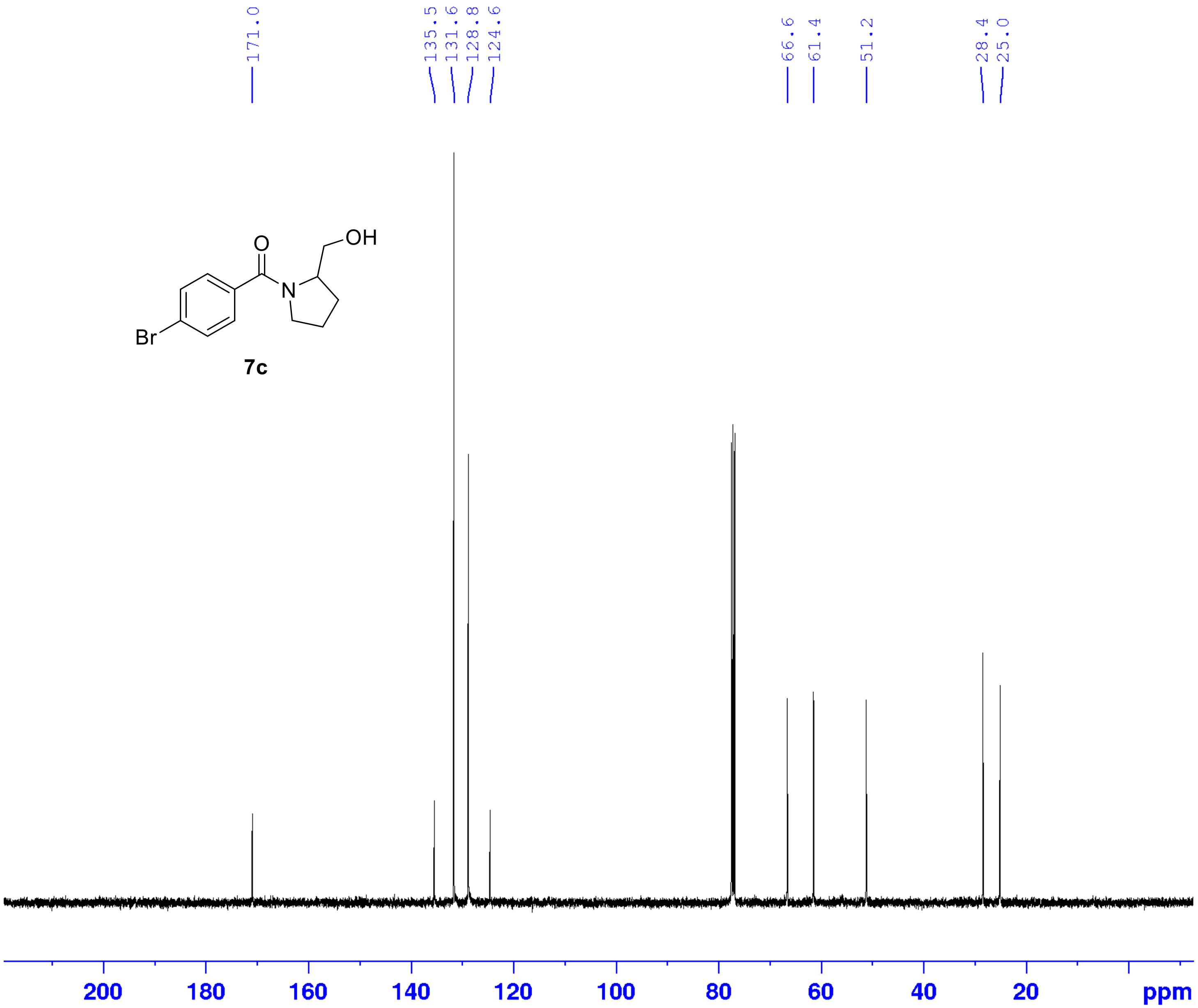

Current Data Parameters
NAME KK-607
EXPNO 20
PROCNO 1

F2 - Acquisition Parameters
Date_ 20230906
Time 20.21 h
INSTRUM spect
PROBHD Z116098_0048 (
PULPROG zgpg30
TD 65536
SOLVENT CDCl3
NS 256
DS 4
SWH 24038.461 Hz
FIDRES 0.733596 Hz
AQ 1.3631488 sec
RG 181.72
DW 20.800 usec
DE 8.54 usec
TE 298.1 K
D1 2.00000000 sec
D11 0.03000000 sec
TD0 8
SFO1 100.6228303 MHz
NUC1 13C
P0 3.00 usec
P1 9.00 usec
PLW1 77.00000000 W
SFO2 400.1316005 MHz
NUC2 1H
CPDPRG[2 waltz16
PCPD2 90.00 usec
PLW2 24.00000000 W
PLW12 0.25352001 W
PLW13 0.12751999 W

F2 - Processing parameters
SI 65536
SF 100.6127654 MHz
WDW EM
SSB 0
LB 1.00 Hz
GB 0
PC 1.40

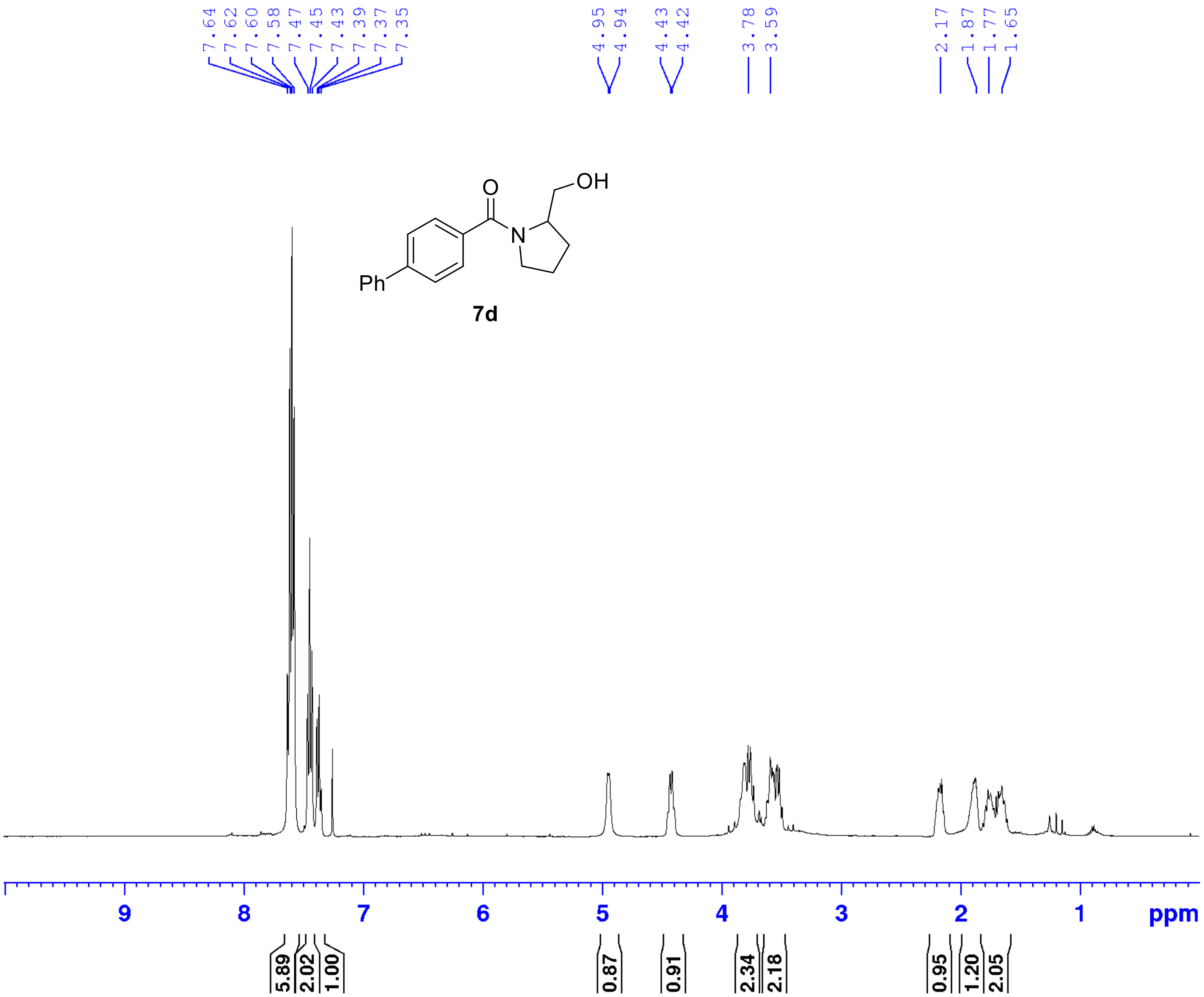


```
Current Data Parameters
NAME                 KK-661
EXPNO                    20
PROCNO                    1

F2 - Acquisition Parameters
Date_              20240328
Time                  15.36 h
INSTRUM               spect
PROBHD     Z116098_0048 (
PULPROG                zg30
TD                    65536
SOLVENT               CDCl3
NS                       16
DS                        2
SWH                8223.685 Hz
FIDRES             0.250967 Hz
AQ                3.9845889 sec
RG                    50.36
DW                   60.800 usec
DE                    10.80 usec
TE                    298.1 K
D1               2.00000000 sec
TD0                       1
SFO1            400.1324710 MHz
NUC1                     1H
P0                     3.08 usec
P1                     9.25 usec
PLW1            24.00000000 W

F2 - Processing parameters
SI                    32768
SF              400.1300107 MHz
WDW                      EM
SSB                       0
LB                     0.30 Hz
GB                        0
PC                     1.50
```

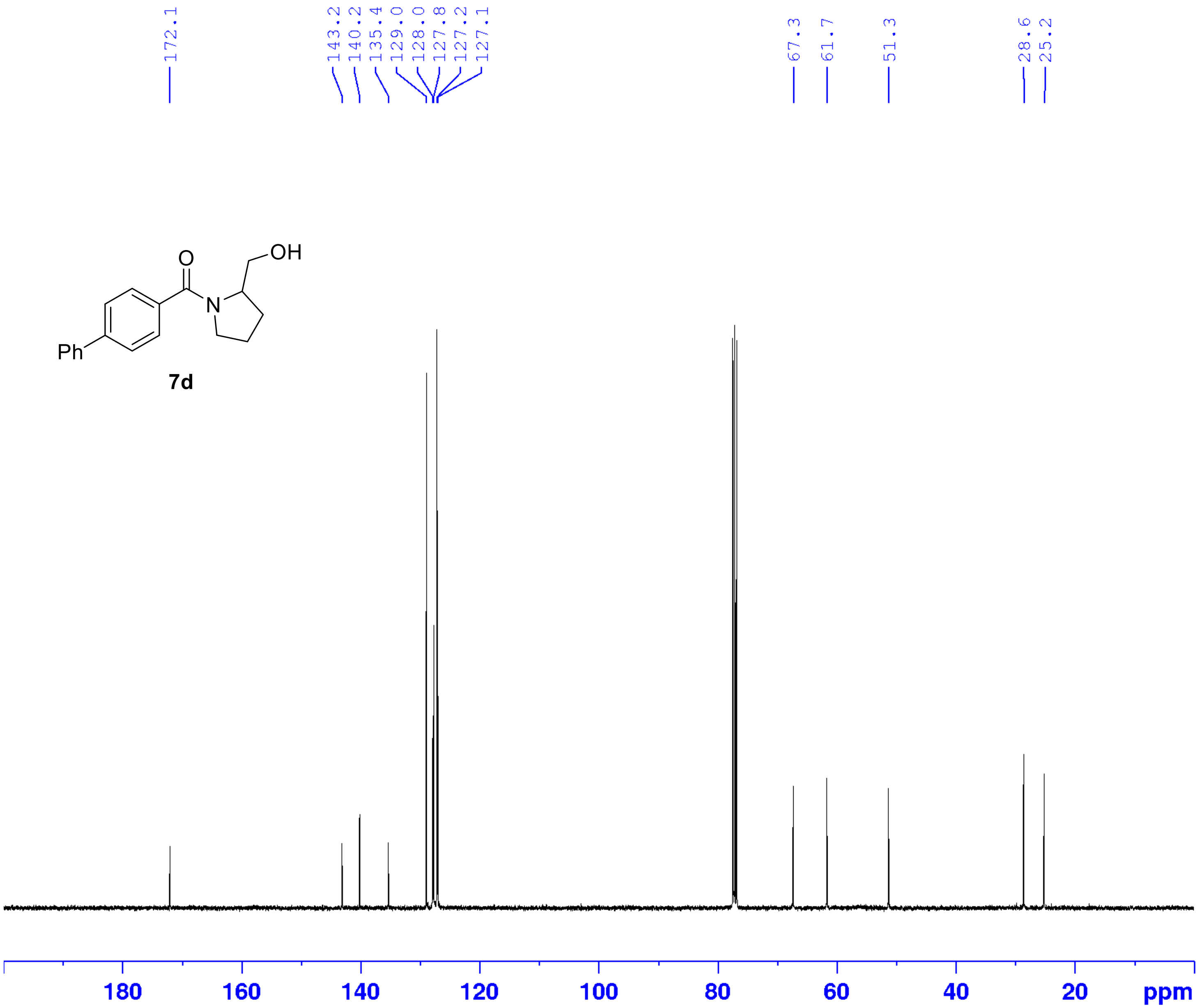

Current Data Parameters
NAME KK-661
EXPNO 23
PROCNO 1

F2 - Acquisition Parameters
Date_ 20240328
Time 22.38 h
INSTRUM spect
PROBHD Z116098_0048 (
PULPROG zgpg30
TD 65536
SOLVENT CDCl3
NS 1024
DS 4
SWH 24038.461 Hz
FIDRES 0.733596 Hz
AQ 1.3631488 sec
RG 181.72
DW 20.800 usec
DE 8.54 usec
TE 298.1 K
D1 2.00000000 sec
D11 0.03000000 sec
TD0 8
SFO1 100.6228303 MHz
NUC1 13C
P0 3.00 usec
P1 9.00 usec
PLW1 77.00000000 W
SFO2 400.1316005 MHz
NUC2 1H
CPDPRG[2 waltz16
PCPD2 90.00 usec
PLW2 24.00000000 W
PLW12 0.25352001 W
PLW13 0.12751999 W

F2 - Processing parameters
SI 65536
SF 100.6127622 MHz
WDW EM
SSB 0
LB 1.00 Hz
GB 0
PC 1.40

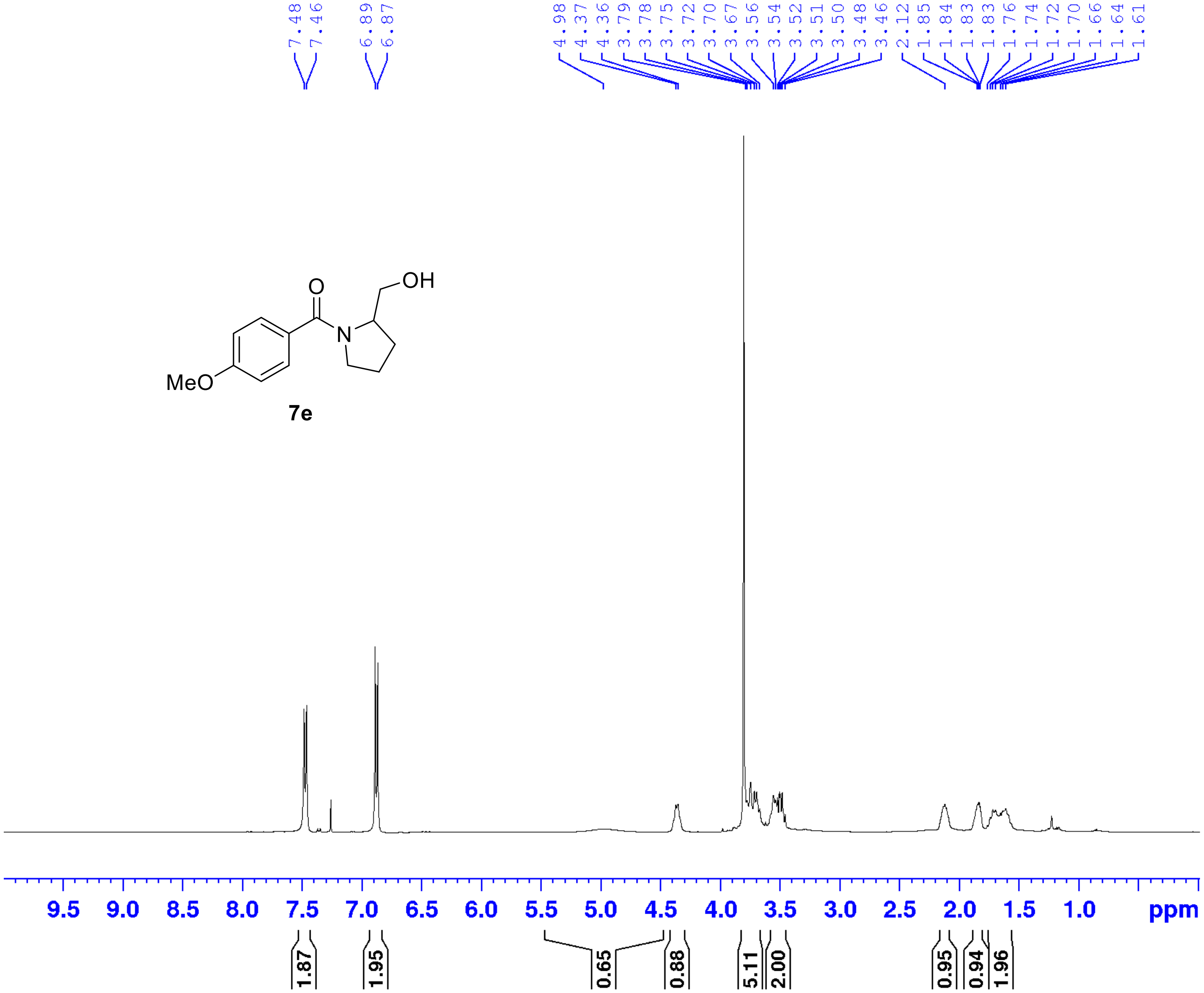

7e

```
Current Data Parameters
NAME                KK-606
EXPNO                   10
PROCNO                   1

F2 - Acquisition Parameters
Date_             20230904
Time                 14.38 h
INSTRUM              spect
PROBHD    Z116098_0048 (
PULPROG               zg30
TD                   65536
SOLVENT              CDCl3
NS                      16
DS                       2
SWH               8223.685 Hz
FIDRES            0.250967 Hz
AQ               3.9845889 sec
RG                   32.09
DW                  60.800 usec
DE                   10.80 usec
TE                   298.1 K
D1              2.00000000 sec
TD0                      1
SFO1           400.1324710 MHz
NUC1                    1H
P0                    3.08 usec
P1                    9.25 usec
PLW1           24.00000000 W

F2 - Processing parameters
SI                   32768
SF             400.1300102 MHz
WDW                     EM
SSB                      0
LB                    0.30 Hz
GB                       0
PC                    1.50
```

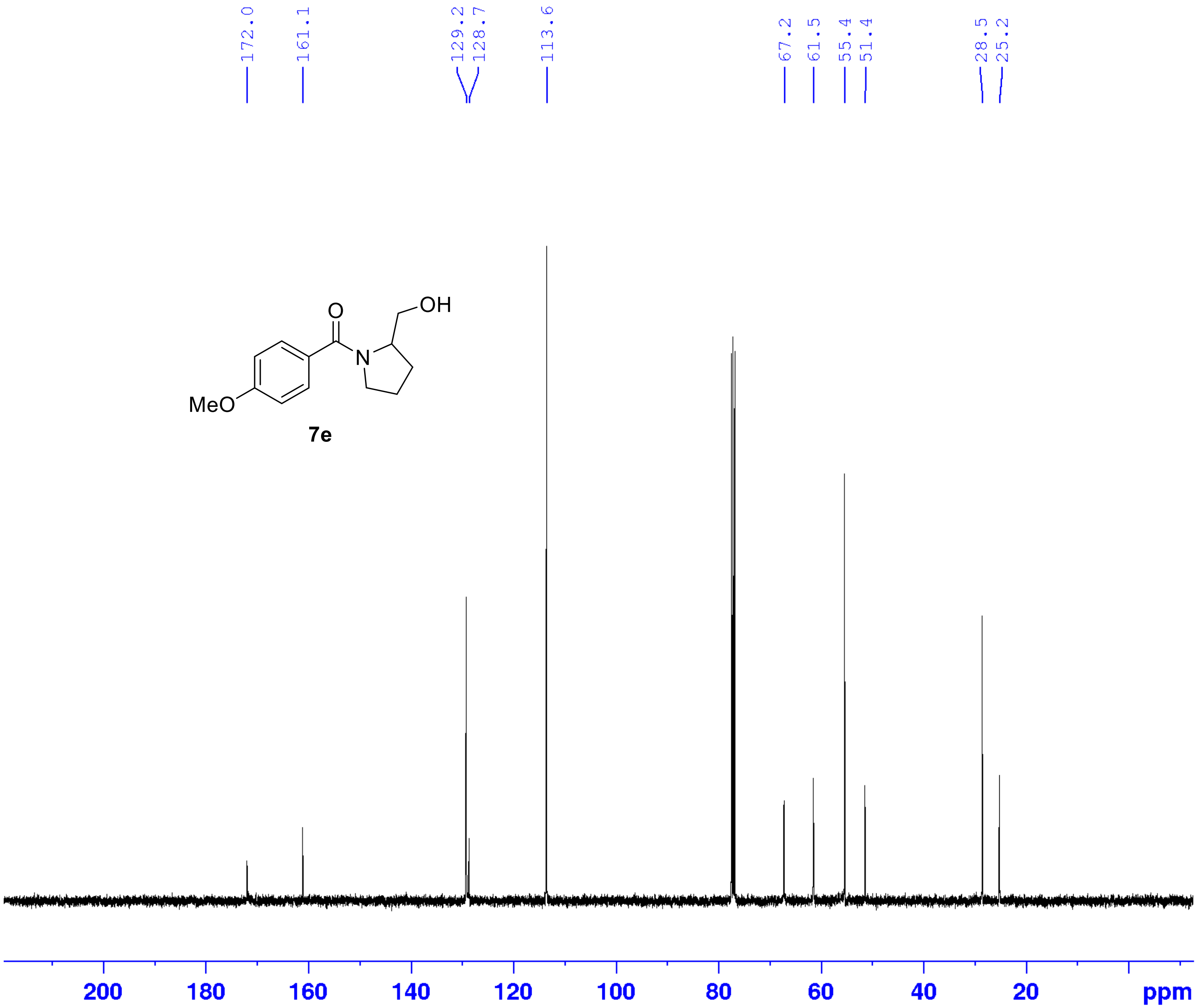

Current Data Parameters
NAME KK-606
EXPNO 11
PROCNO 1

F2 - Acquisition Parameters
Date_ 20230906
Time 19.26 h
INSTRUM spect
PROBHD Z116098_0048 (
PULPROG zgpg30
TD 65536
SOLVENT CDCl3
NS 256
DS 4
SWH 24038.461 Hz
FIDRES 0.733596 Hz
AQ 1.3631488 sec
RG 181.72
DW 20.800 usec
DE 8.54 usec
TE 298.1 K
D1 2.00000000 sec
D11 0.03000000 sec
TD0 8
SFO1 100.6228303 MHz
NUC1 13C
P0 3.00 usec
P1 9.00 usec
PLW1 77.00000000 W
SFO2 400.1316005 MHz
NUC2 1H
CPDPRG[2 waltz16
PCPD2 90.00 usec
PLW2 24.00000000 W
PLW12 0.25352001 W
PLW13 0.12751999 W

F2 - Processing parameters
SI 65536
SF 100.6127649 MHz
WDW EM
SSB 0
LB 1.00 Hz
GB 0
PC 1.40

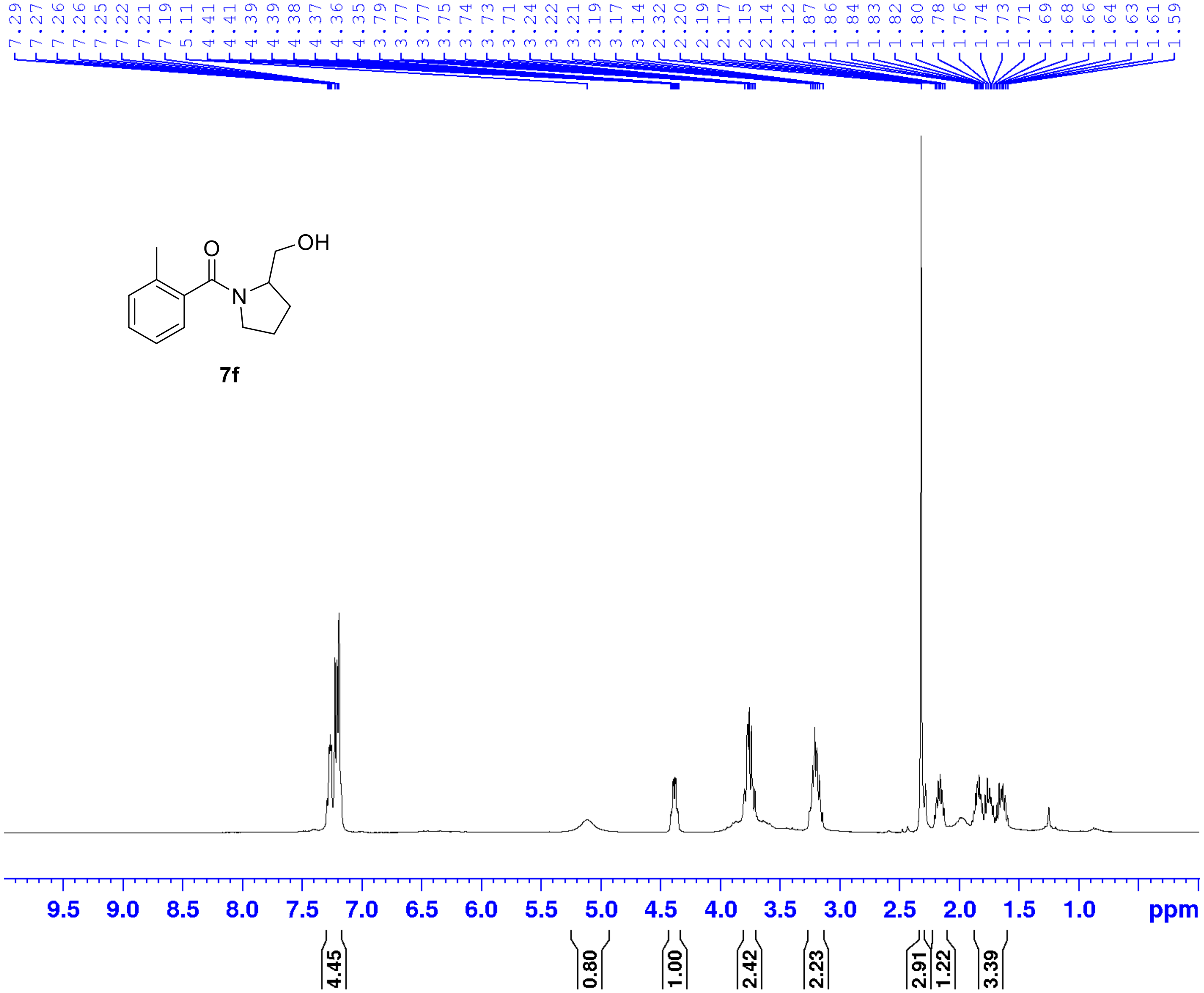

Current Data Parameters

NAME	KK-585
EXPNO	10
PROCNO	1

F2 - Acquisition Parameters

Date_	20230718
Time	14.20 h
INSTRUM	spect
PROBHD	Z116098_0048 (
PULPROG	zg30
TD	65536
SOLVENT	CDCl3
NS	16
DS	2
SWH	8223.685 Hz
FIDRES	0.250967 Hz
AQ	3.9845889 sec
RG	40.16
DW	60.800 usec
DE	10.80 usec
TE	298.2 K
D1	2.00000000 sec
TD0	1
SFO1	400.1324710 MHz
NUC1	1H
P0	3.08 usec
P1	9.25 usec
PLW1	24.00000000 W

F2 - Processing parameters

SI	32768
SF	400.1300087 MHz
WDW	EM
SSB	0
LB	0.30 Hz
GB	0
PC	1.50

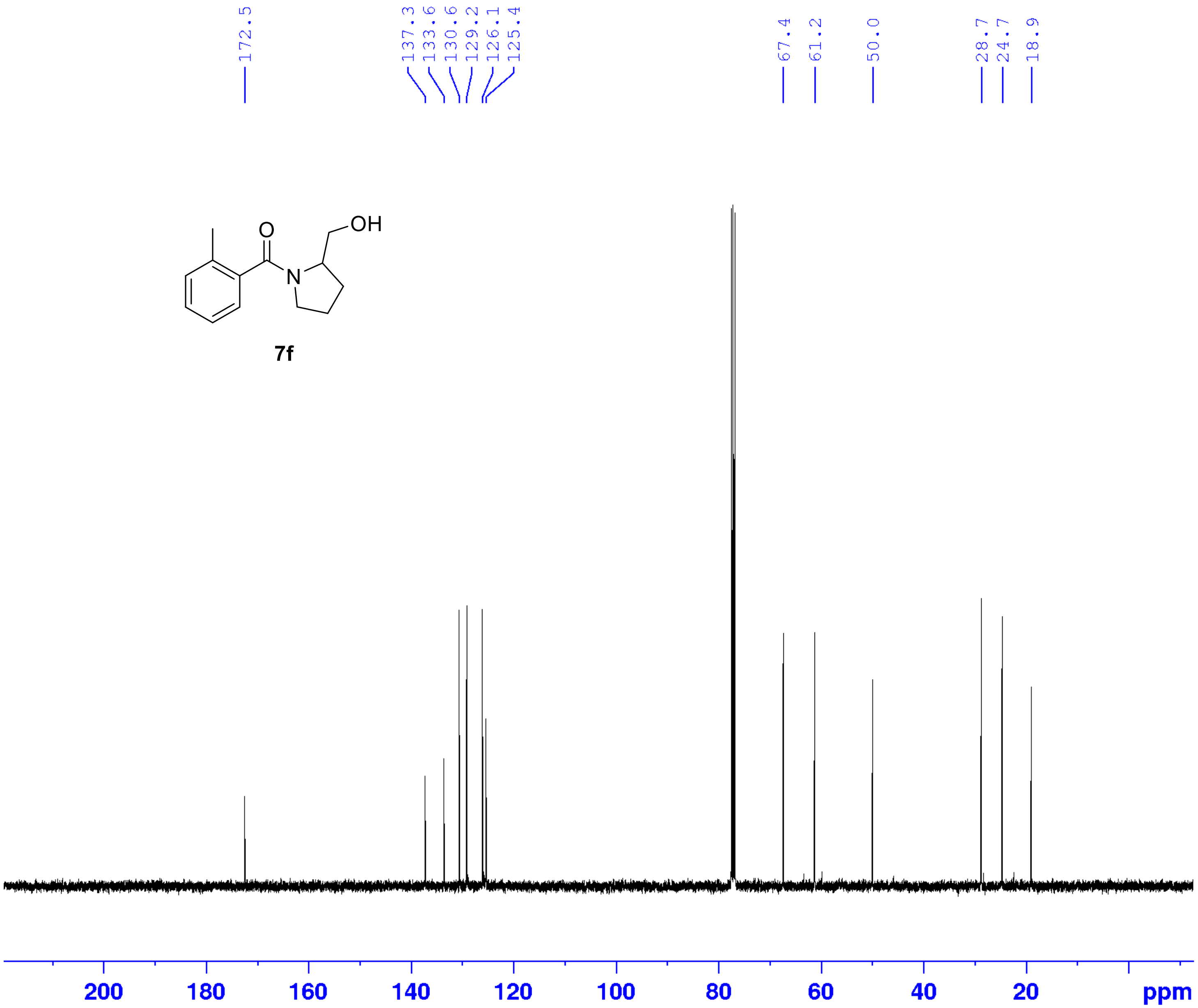

7f

Current Data Parameters
NAME KK-585
EXPNO 11
PROCNO 1

F2 - Acquisition Parameters

Date_	20230718
Time	20.30 h
INSTRUM	spect
PROBHD	Z116098_0048 (
PULPROG	zgpg30
TD	65536
SOLVENT	CDCl3
NS	256
DS	4
SWH	24038.461 Hz
FIDRES	0.733596 Hz
AQ	1.3631488 sec
RG	181.72
DW	20.800 usec
DE	8.54 usec
TE	298.1 K
D1	2.00000000 sec
D11	0.03000000 sec
TD0	8
SFO1	100.6228303 MHz
NUC1	13C
P0	3.00 usec
P1	9.00 usec
PLW1	77.00000000 W
SFO2	400.1316005 MHz
NUC2	1H
CPDPRG[2	waltz16
PCPD2	90.00 usec
PLW2	24.00000000 W
PLW12	0.25352001 W
PLW13	0.12751999 W

F2 - Processing parameters

SI	65536
SF	100.6127602 MHz
WDW	EM
SSB	0
LB	1.00 Hz
GB	0
PC	1.40

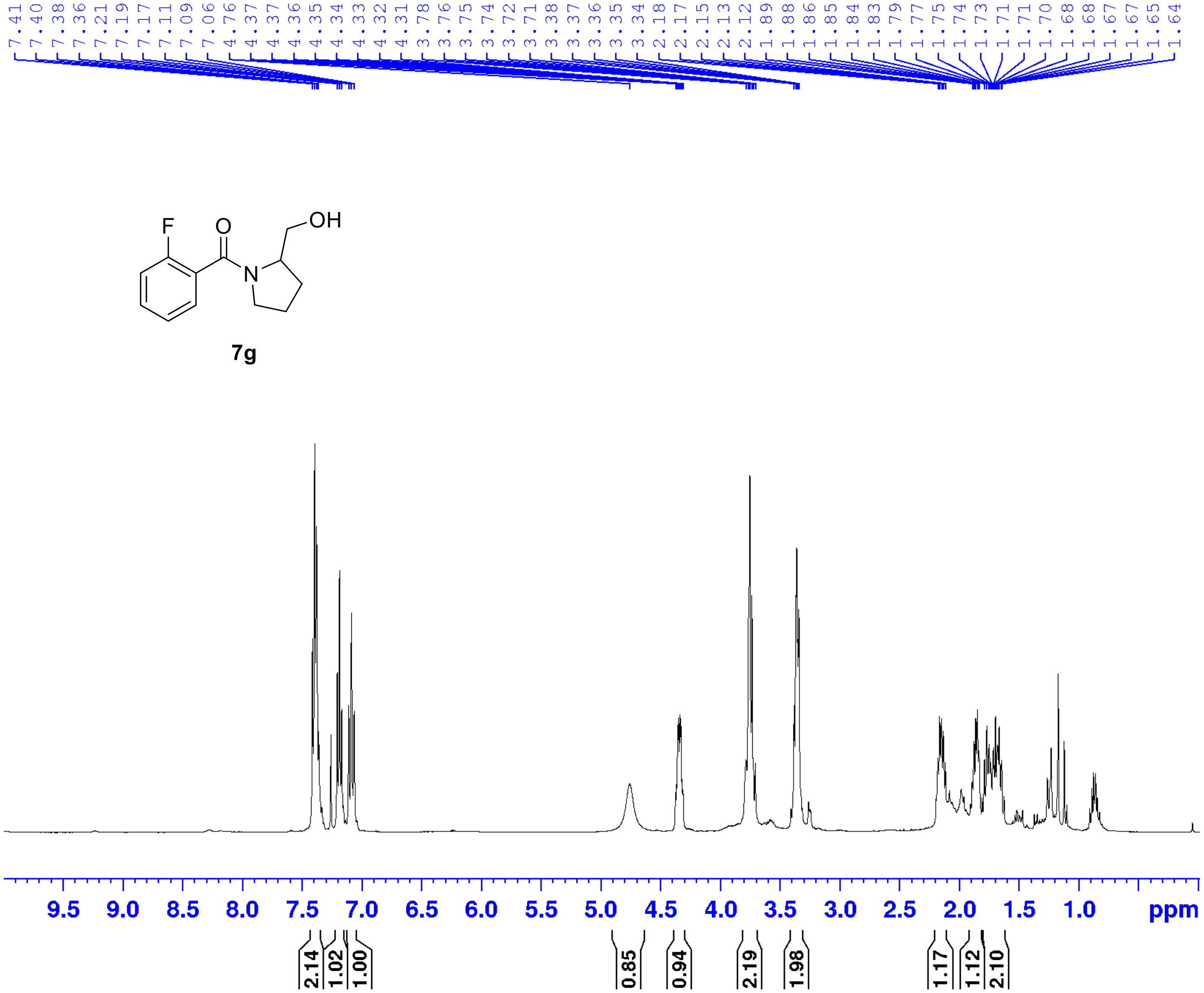

BRUKER

Current Data Parameters
NAME KK-604
EXPNO 20
PROCNO 1

F2 - Acquisition Parameters
Date_ 20230910
Time 12.21 h
INSTRUM spect
PROBHD Z116098_0048 (
PULPROG zg30
TD 65536
SOLVENT CDCl3
NS 16
DS 2
SWH 8223.685 Hz
FIDRES 0.250967 Hz
AQ 3.9845889 sec
RG 40.16
DW 60.800 usec
DE 10.80 usec
TE 298.2 K
D1 2.00000000 sec
TD0 1
SFO1 400.1324710 MHz
NUC1 1H
P0 3.08 usec
P1 9.25 usec
PLW1 24.00000000 W

F2 - Processing parameters
SI 32768
SF 400.1300105 MHz
WDW EM
SSB 0
LB 0.30 Hz
GB 0
PC 1.50

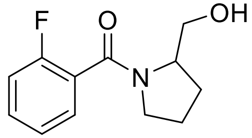

7g

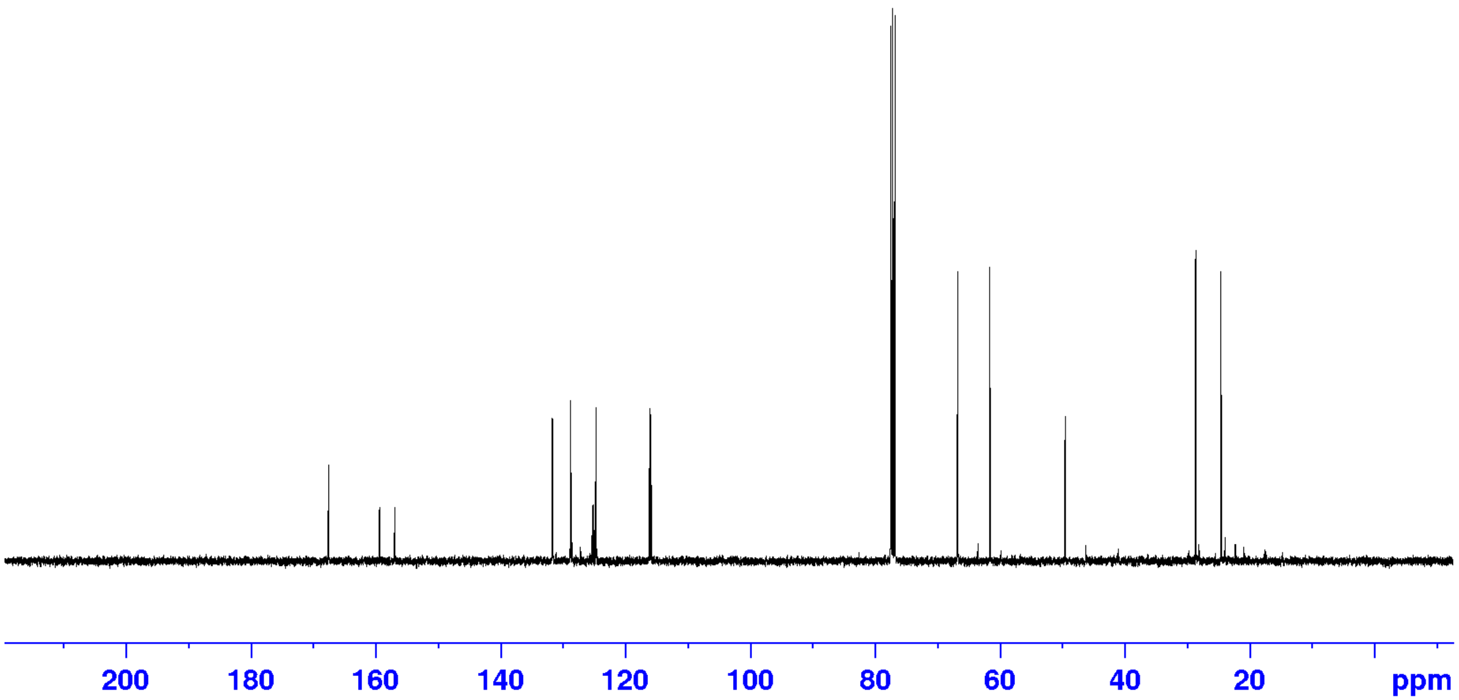

BRUKER

Current Data Parameters

NAME	KK-604
EXPNO	21
PROCNO	1

F2 - Acquisition Parameters

Date_	20230910
Time	13.13 h
INSTRUM	spect
PROBHD	Z116098_0048 (
PULPROG	zgpg30
TD	65536
SOLVENT	CDCl3
NS	256
DS	4
SWH	24038.461 Hz
FIDRES	0.733596 Hz
AQ	1.3631488 sec
RG	181.72
DW	20.800 usec
DE	8.54 usec
TE	298.2 K
D1	2.00000000 sec
D11	0.03000000 sec
TD0	8
SFO1	100.6228303 MHz
NUC1	13C
P0	3.00 usec
P1	9.00 usec
PLW1	77.00000000 W
SFO2	400.1316005 MHz
NUC2	1H
CPDPRG[2	waltz16
PCPD2	90.00 usec
PLW2	24.00000000 W
PLW12	0.25352001 W
PLW13	0.12751999 W

F2 - Processing parameters

SI	65536
SF	100.6127615 MHz
WDW	EM
SSB	0
LB	1.00 Hz
GB	0
PC	1.40

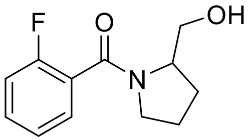

7g

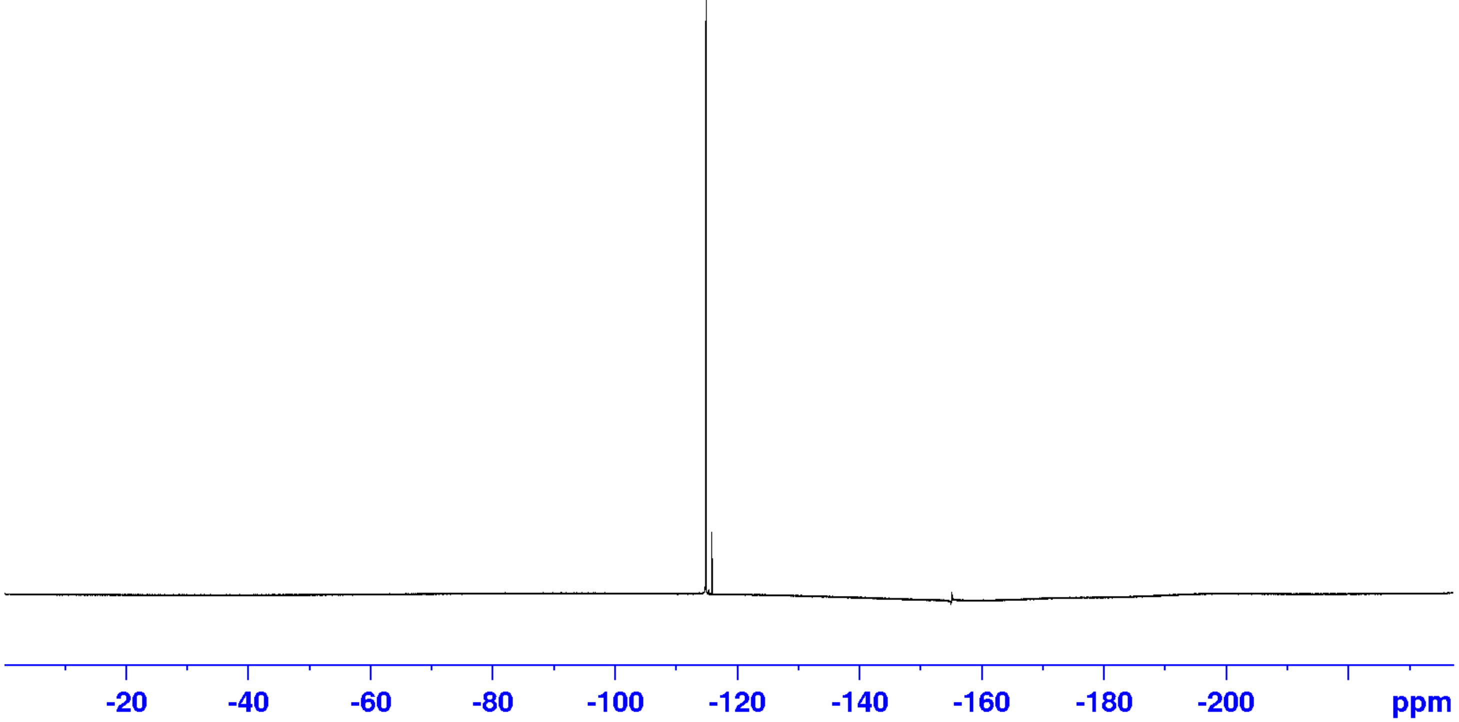

Current Data Parameters
NAME KK-604
EXPNO 22
PROCNO 1

F2 - Acquisition Parameters
Date_ 20230910
Time 14.38 h
INSTRUM spect
PROBHD Z116098_0048 (
PULPROG zg
TD 262144
SOLVENT CDCl3
NS 16
DS 0
SWH 89285.711 Hz
FIDRES 0.681196 Hz
AQ 1.4680064 sec
RG 181.72
DW 5.600 usec
DE 7.11 usec
TE 298.1 K
D1 4.00000000 sec
TD0 1
SFO1 376.4536869 MHz
NUC1 19F
P1 14.00 usec
PLW1 20.00000000 W

F2 - Processing parameters
SI 262144
SF 376.4983660 MHz
WDW EM
SSB 0
LB 0.50 Hz
GB 0
PC 1.00

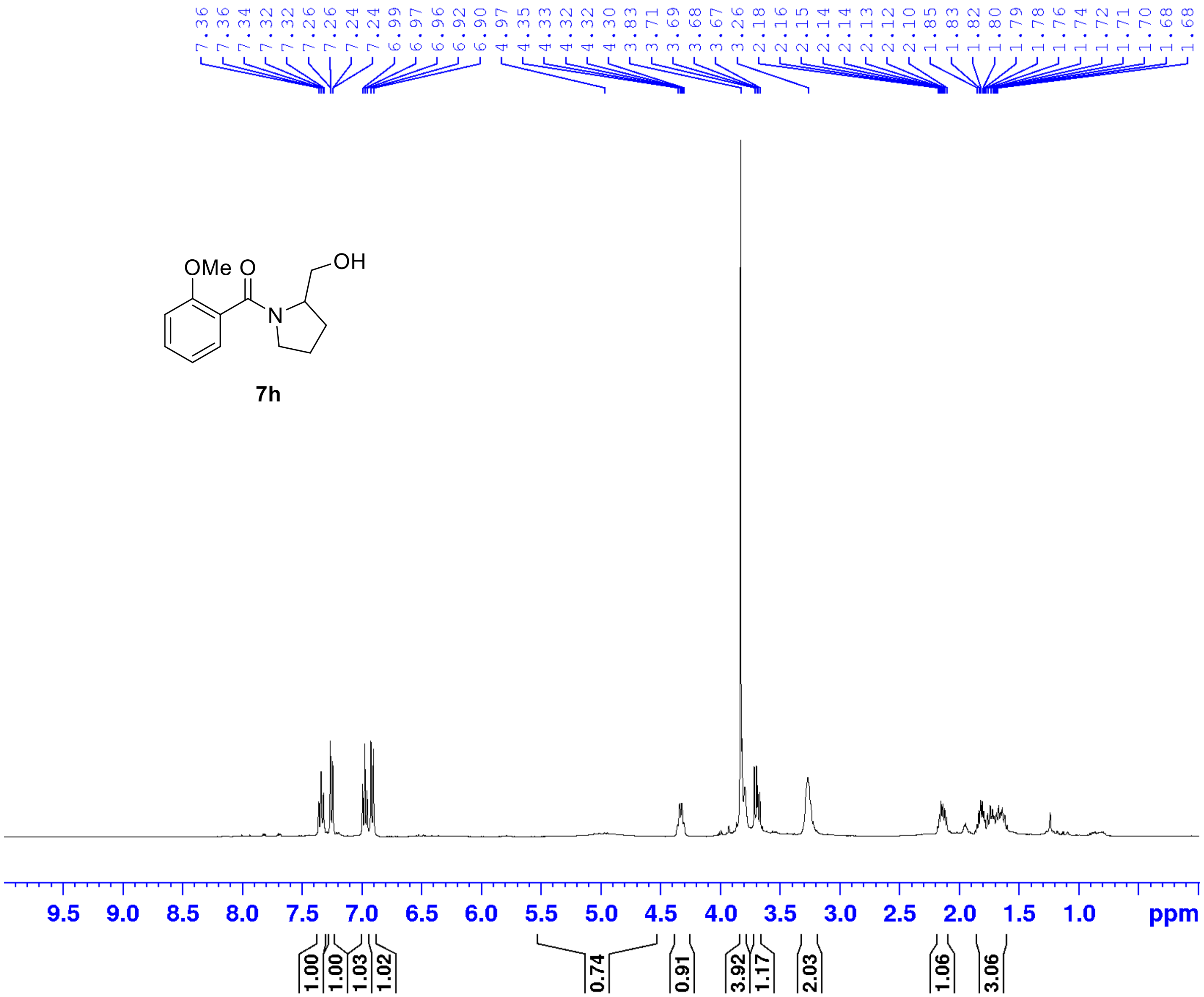

Current Data Parameters
NAME KK-611
EXPNO 10
PROCNO 1

F2 - Acquisition Parameters
Date_ 20230904
Time 14.43 h
INSTRUM spect
PROBHD Z116098_0048 (
PULPROG zg30
TD 65536
SOLVENT CDCl3
NS 16
DS 2
SWH 8223.685 Hz
FIDRES 0.250967 Hz
AQ 3.9845889 sec
RG 35.7
DW 60.800 usec
DE 10.80 usec
TE 298.1 K
D1 2.00000000 sec
TD0 1
SFO1 400.1324710 MHz
NUC1 1H
P0 3.08 usec
P1 9.25 usec
PLW1 24.00000000 W

F2 - Processing parameters
SI 32768
SF 400.1300087 MHz
WDW EM
SSB 0
LB 0.30 Hz
GB 0
PC 1.50

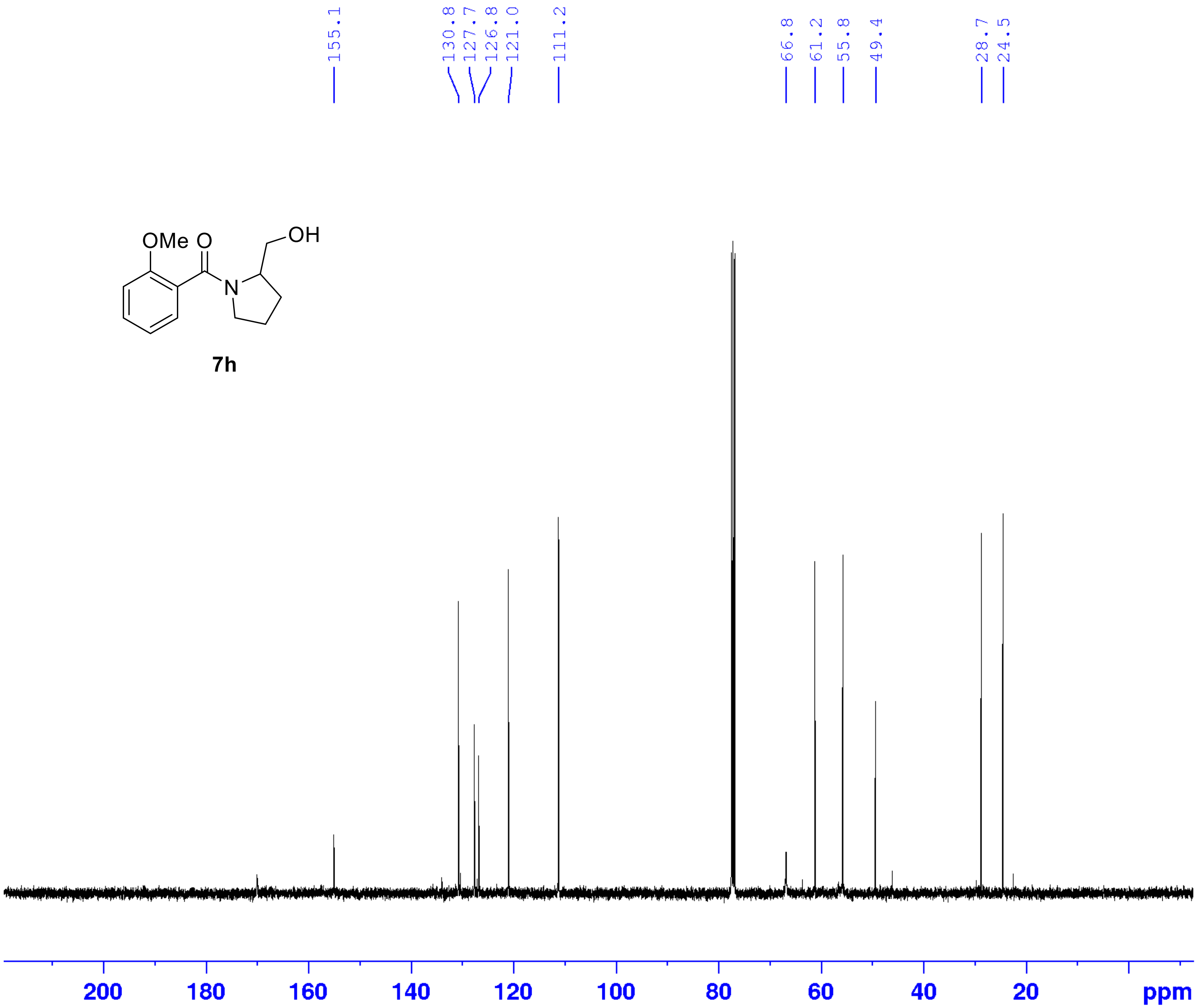


```
Current Data Parameters
NAME                KK-611
EXPNO                   11
PROCNO                   1

F2 - Acquisition Parameters
Date_             20230906
Time                 20.58 h
INSTRUM              spect
PROBHD    Z116098_0048 (
PULPROG            zgpg30
TD                   65536
SOLVENT              CDCl3
NS                     256
DS                       4
SWH              24038.461 Hz
FIDRES            0.733596 Hz
AQ               1.3631488 sec
RG                  181.72
DW                  20.800 usec
DE                    8.54 usec
TE                   298.1 K
D1              2.00000000 sec
D11             0.03000000 sec
TD0                      8
SFO1           100.6228303 MHz
NUC1                   13C
P0                    3.00 usec
P1                    9.00 usec
PLW1           77.00000000 W
SFO2           400.1316005 MHz
NUC2                    1H
CPDPRG[2           waltz16
PCPD2                90.00 usec
PLW2           24.00000000 W
PLW12           0.25352001 W
PLW13           0.12751999 W

F2 - Processing parameters
SI                   65536
SF             100.6127641 MHz
WDW                     EM
SSB                      0
LB                    1.00 Hz
GB                       0
PC                    1.40
```

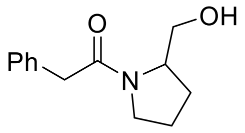

7k

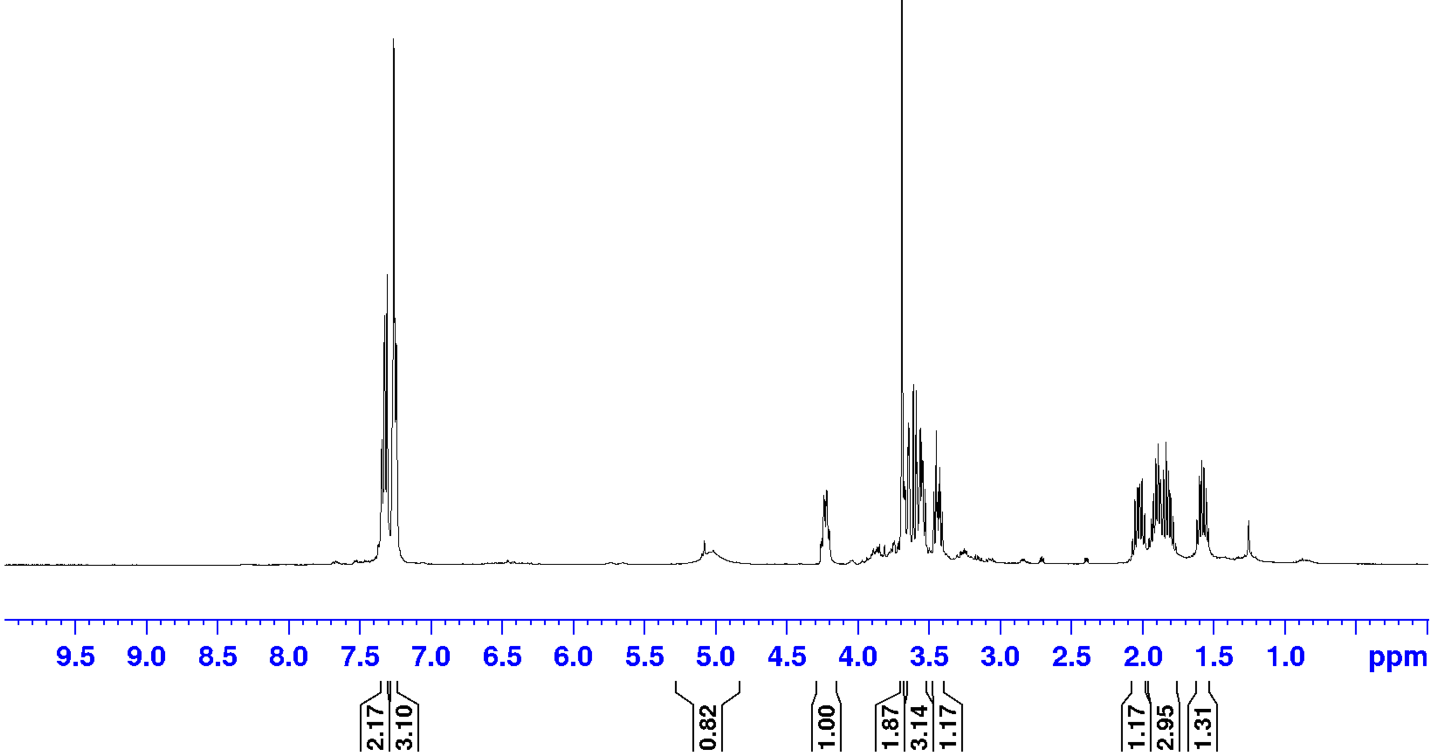


```
Current Data Parameters
NAME                KK-609
EXPNO                   10
PROCNO                   1

F2 - Acquisition Parameters
Date_             20230904
Time                 14.26 h
INSTRUM              spect
PROBHD     Z116098_0048 (
PULPROG               zg30
TD                   65536
SOLVENT              CDCl3
NS                      16
DS                       2
SWH               8223.685 Hz
FIDRES            0.250967 Hz
AQ               3.9845889 sec
RG                   46.39
DW                  60.800 usec
DE                   10.80 usec
TE                   298.1 K
D1              2.00000000 sec
TD0                      1
SFO1           400.1324710 MHz
NUC1                    1H
P0                    3.08 usec
P1                    9.25 usec
PLW1           24.00000000 W

F2 - Processing parameters
SI                   32768
SF             400.1300102 MHz
WDW                     EM
SSB                      0
LB                    0.30 Hz
GB                       0
PC                    1.50
```

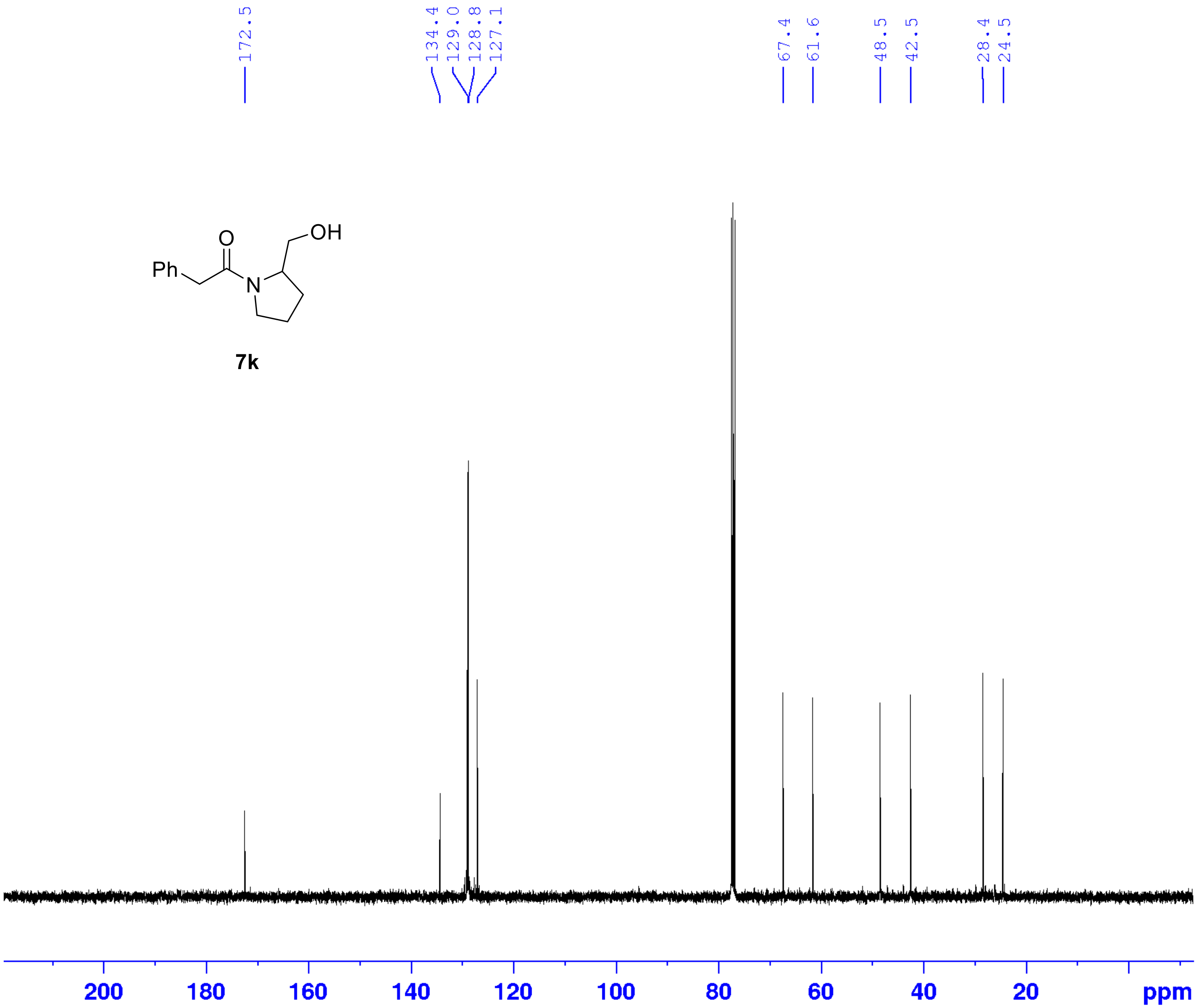

7k

Current Data Parameters
NAME KK-609
EXPNO 11
PROCNO 1

F2 - Acquisition Parameters
Date_ 20230906
Time 9.50 h
INSTRUM spect
PROBHD Z116098_0048 (
PULPROG zgpg30
TD 65536
SOLVENT CDCl3
NS 256
DS 4
SWH 24038.461 Hz
FIDRES 0.733596 Hz
AQ 1.3631488 sec
RG 181.72
DW 20.800 usec
DE 8.54 usec
TE 298.1 K
D1 2.00000000 sec
D11 0.03000000 sec
TD0 8
SFO1 100.6228303 MHz
NUC1 13C
P0 3.00 usec
P1 9.00 usec
PLW1 77.00000000 W
SFO2 400.1316005 MHz
NUC2 1H
CPDPRG[2 waltz16
PCPD2 90.00 usec
PLW2 24.00000000 W
PLW12 0.25352001 W
PLW13 0.12751999 W

F2 - Processing parameters
SI 65536
SF 100.6127599 MHz
WDW EM
SSB 0
LB 1.00 Hz
GB 0
PC 1.40

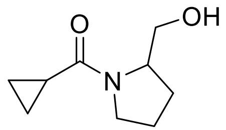

7m

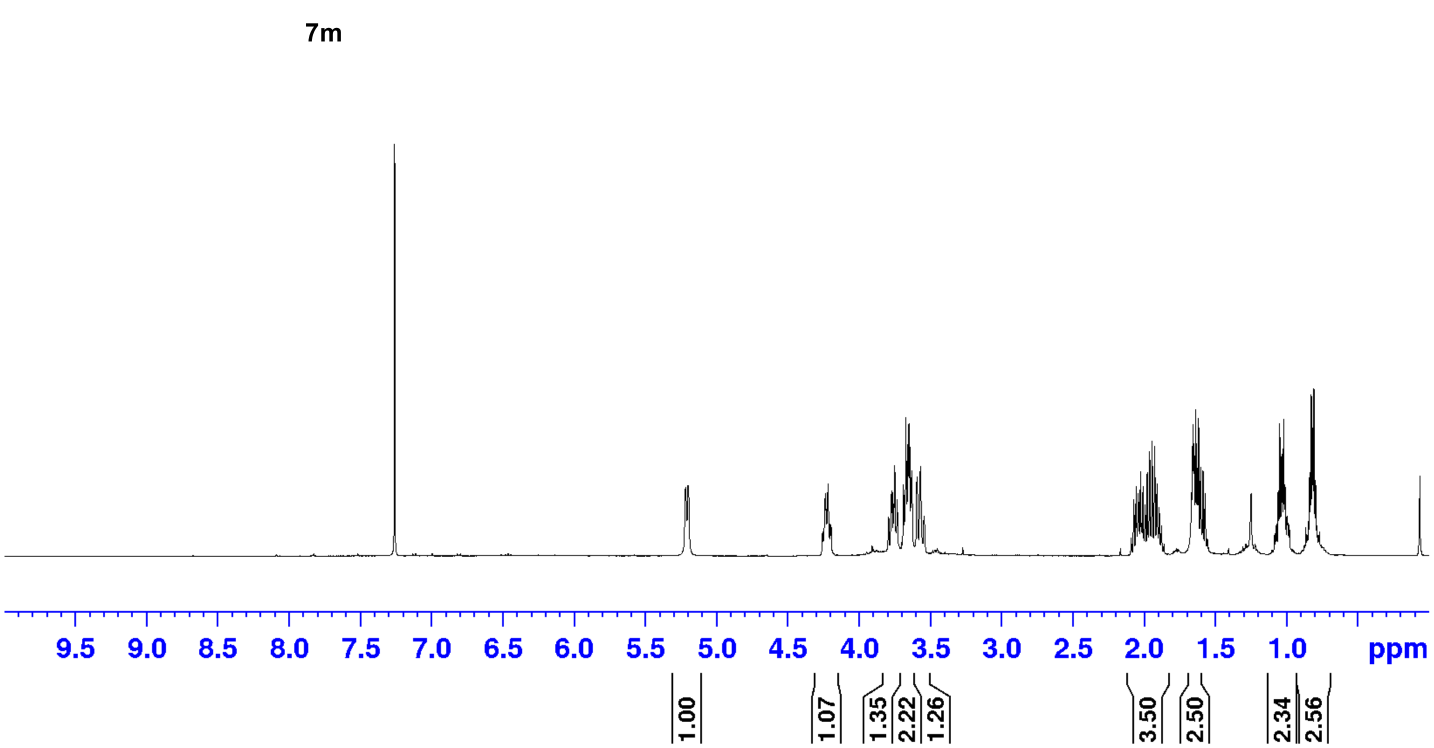

BRUKER

```
Current Data Parameters
NAME                KK-587
EXPNO                   31
PROCNO                   1

F2 - Acquisition Parameters
Date_             20240325
Time                 21.28 h
INSTRUM              spect
PROBHD    Z116098_0048 (
PULPROG               zg30
TD                   65536
SOLVENT              CDCl3
NS                      64
DS                       2
SWH               8223.685 Hz
FIDRES            0.250967 Hz
AQ               3.9845889 sec
RG                  134.04
DW                  60.800 usec
DE                   10.80 usec
TE                   298.2 K
D1              1.00000000 sec
TD0                      1
SFO1           400.1324710 MHz
NUC1                    1H
P0                    3.08 usec
P1                    9.25 usec
PLW1           24.00000000 W

F2 - Processing parameters
SI                   32768
SF             400.1300102 MHz
WDW                     EM
SSB                      0
LB                    0.30 Hz
GB                       0
PC                    1.50
```

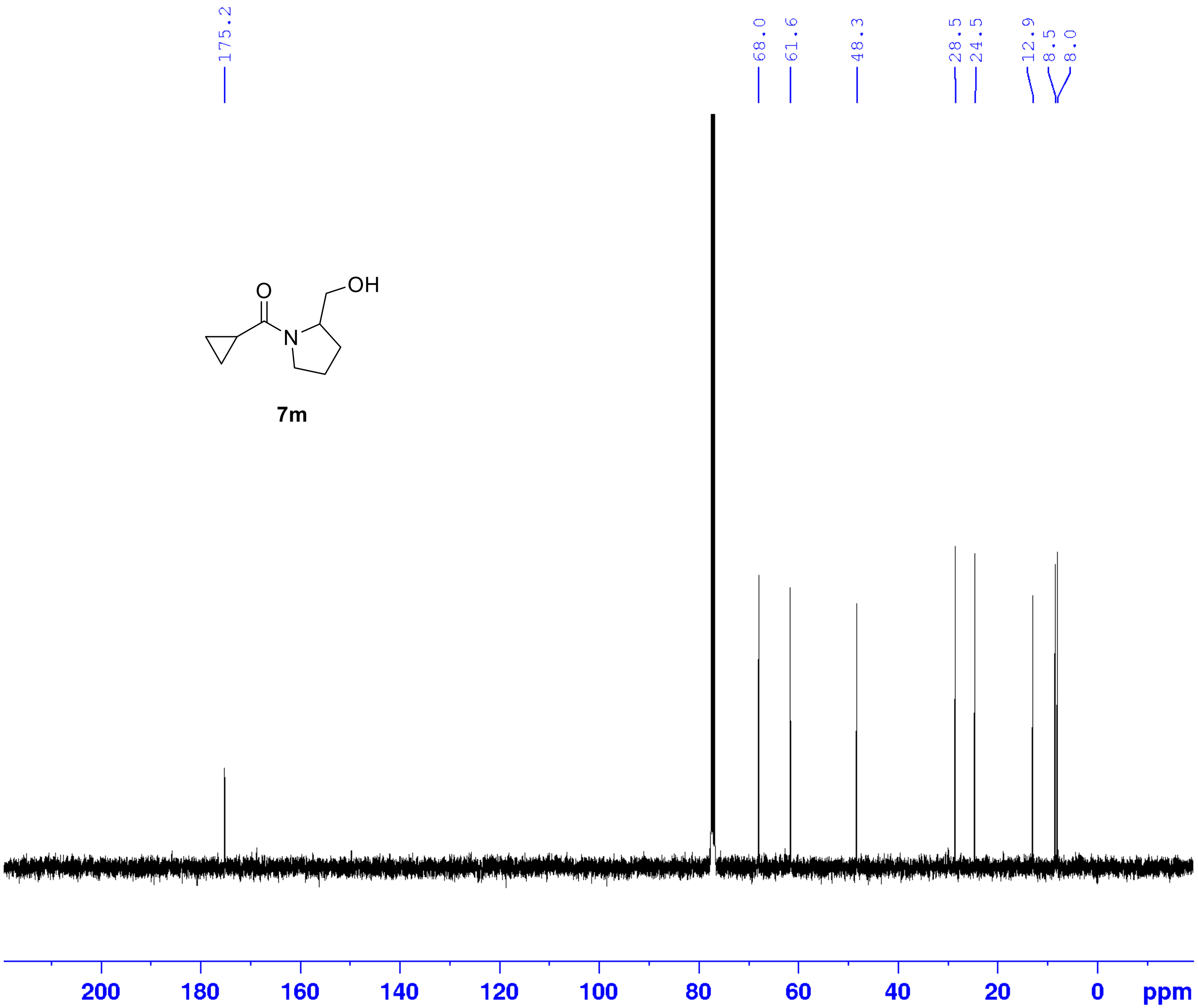

7m

Current Data Parameters	
NAME	KK-587
EXPNO	33
PROCNO	1
F2 - Acquisition Parameters	
Date_	20240325
Time	22.42 h
INSTRUM	spect
PROBHD	Z116098_0048 (
PULPROG	zgpg30
TD	65536
SOLVENT	CDCl3
NS	1024
DS	4
SWH	24038.461 Hz
FIDRES	0.733596 Hz
AQ	1.3631488 sec
RG	181.72
DW	20.800 usec
DE	8.54 usec
TE	298.1 K
D1	2.00000000 sec
D11	0.03000000 sec
TD0	8
SFO1	100.6228303 MHz
NUC1	13C
P0	3.00 usec
P1	9.00 usec
PLW1	77.00000000 W
SFO2	400.1316005 MHz
NUC2	1H
CPDPRG[2	waltz16
PCPD2	90.00 usec
PLW2	24.00000000 W
PLW12	0.25352001 W
PLW13	0.12751999 W
F2 - Processing parameters	
SI	65536
SF	100.6127559 MHz
WDW	EM
SSB	0
LB	1.00 Hz
GB	0
PC	1.40

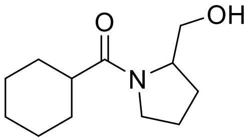

7n

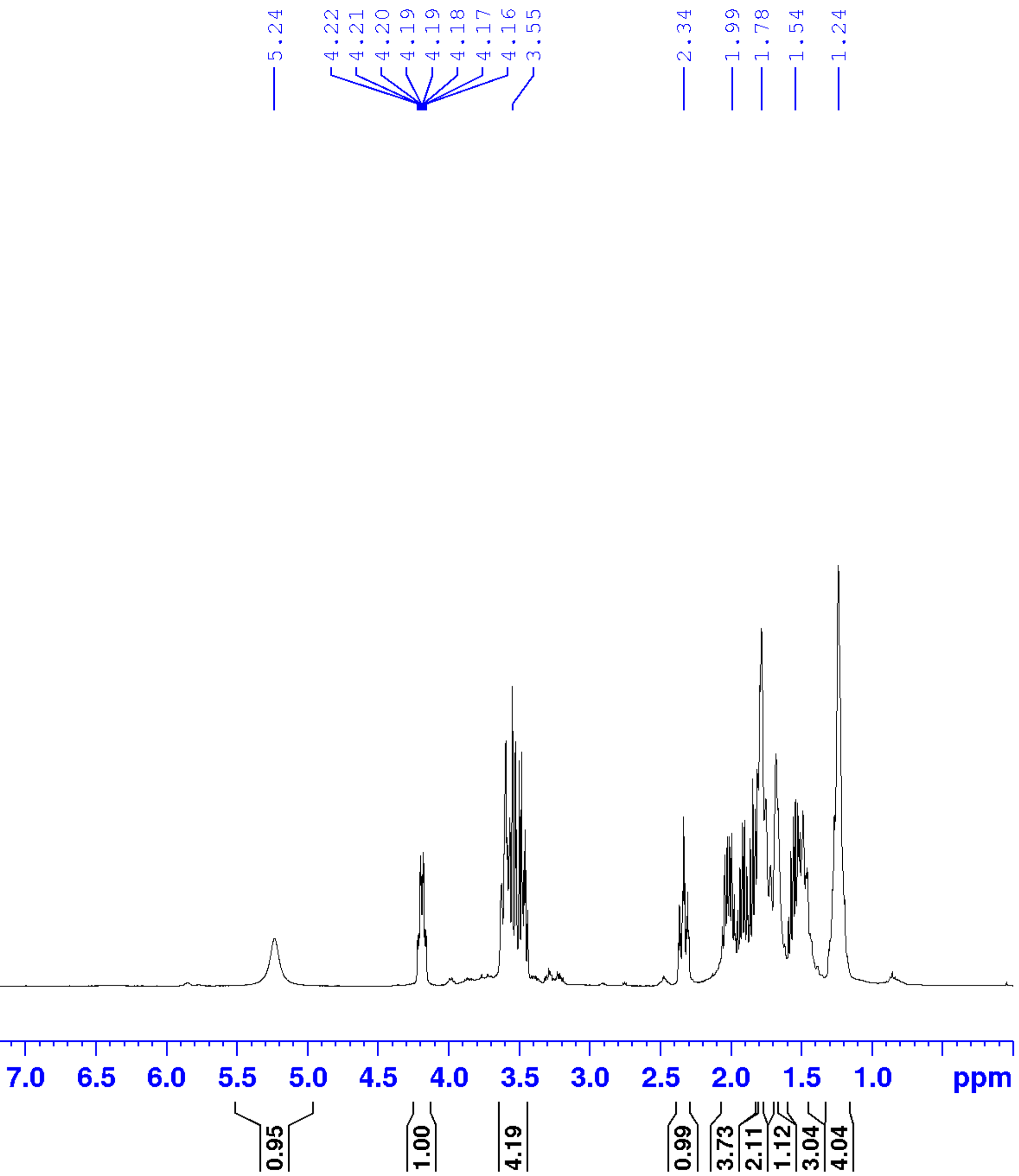

Current Data Parameters
NAME KK-608
EXPNO 10
PROCNO 1

F2 - Acquisition Parameters
Date_ 20230904
Time 15.00 h
INSTRUM spect
PROBHD Z116098_0048 (
PULPROG zg30
TD 65536
SOLVENT CDCl3
NS 16
DS 2
SWH 8223.685 Hz
FIDRES 0.250967 Hz
AQ 3.9845889 sec
RG 35.7
DW 60.800 usec
DE 10.80 usec
TE 298.2 K
D1 2.00000000 sec
TD0 1
SFO1 400.1324710 MHz
NUC1 1H
P0 3.08 usec
P1 9.25 usec
PLW1 24.00000000 W

F2 - Processing parameters
SI 32768
SF 400.1300103 MHz
WDW EM
SSB 0
LB 0.30 Hz
GB 0
PC 1.50

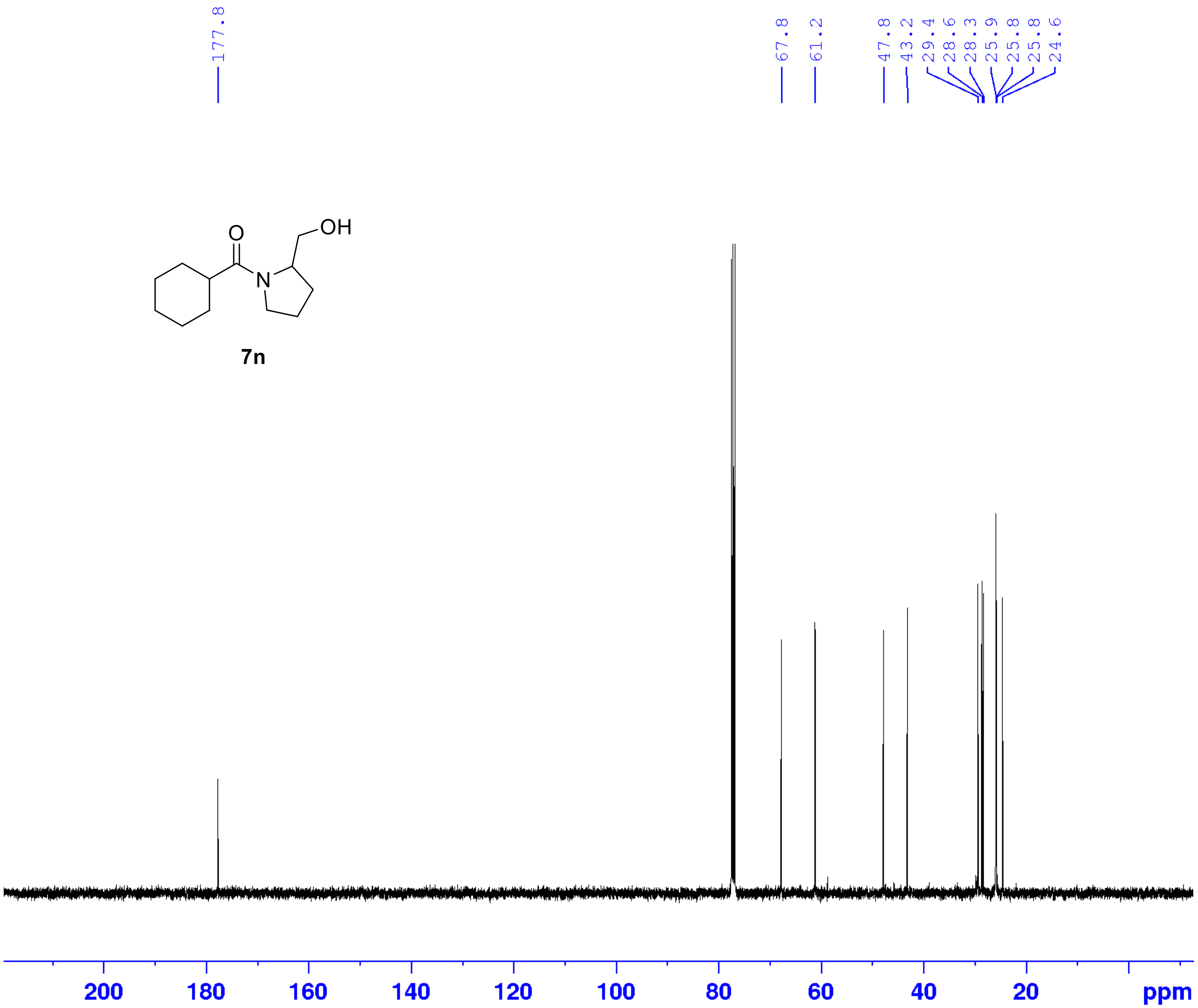

BRUKER

Current Data Parameters

NAME	KK-608
EXPNO	11
PROCNO	1

F2 - Acquisition Parameters

Date_	20230906
Time	20.40 h
INSTRUM	spect
PROBHD	Z116098_0048 (
PULPROG	zgpg30
TD	65536
SOLVENT	CDCl3
NS	256
DS	4
SWH	24038.461 Hz
FIDRES	0.733596 Hz
AQ	1.3631488 sec
RG	181.72
DW	20.800 usec
DE	8.54 usec
TE	298.2 K
D1	2.00000000 sec
D11	0.03000000 sec
TD0	8
SFO1	100.6228303 MHz
NUC1	13C
P0	3.00 usec
P1	9.00 usec
PLW1	77.00000000 W
SFO2	400.1316005 MHz
NUC2	1H
CPDPRG[2	waltz16
PCPD2	90.00 usec
PLW2	24.00000000 W
PLW12	0.25352001 W
PLW13	0.12751999 W

F2 - Processing parameters

SI	65536
SF	100.6127597 MHz
WDW	EM
SSB	0
LB	1.00 Hz
GB	0
PC	1.40

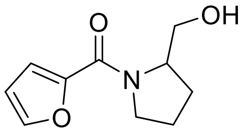

7o

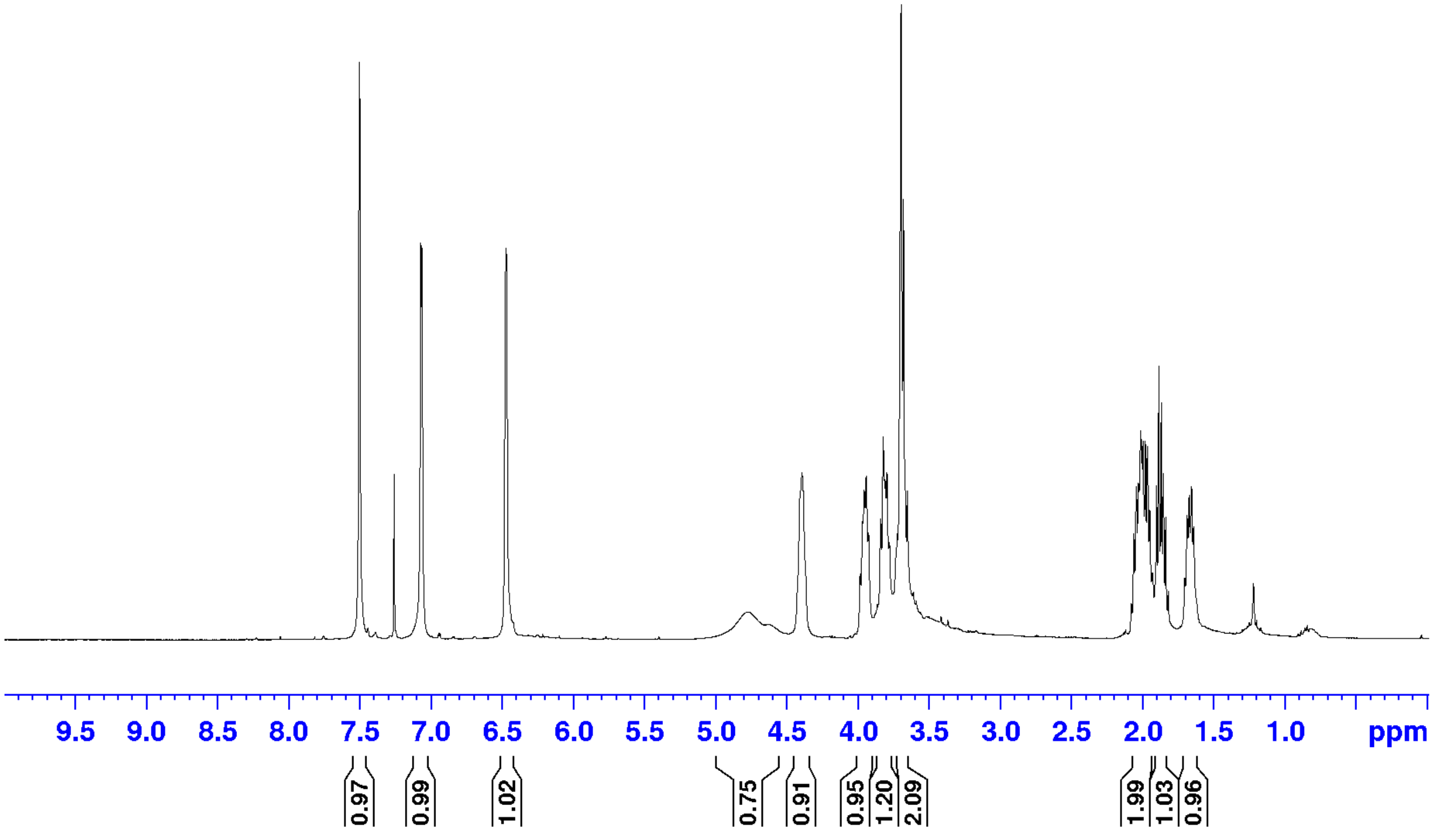

Current Data Parameters
NAME KK-574
EXPNO 30
PROCNO 1

F2 - Acquisition Parameters
Date_ 20230712
Time 12.58 h
INSTRUM spect
PROBHD Z116098_0048 (
PULPROG zg30
TD 65536
SOLVENT CDCl3
NS 16
DS 2
SWH 8223.685 Hz
FIDRES 0.250967 Hz
AQ 3.9845889 sec
RG 35.7
DW 60.800 usec
DE 10.80 usec
TE 298.2 K
D1 2.00000000 sec
TD0 1
SFO1 400.1324710 MHz
NUC1 1H
P0 3.08 usec
P1 9.25 usec
PLW1 24.00000000 W

F2 - Processing parameters
SI 32768
SF 400.1300106 MHz
WDW EM
SSB 0
LB 0.30 Hz
GB 0
PC 1.50

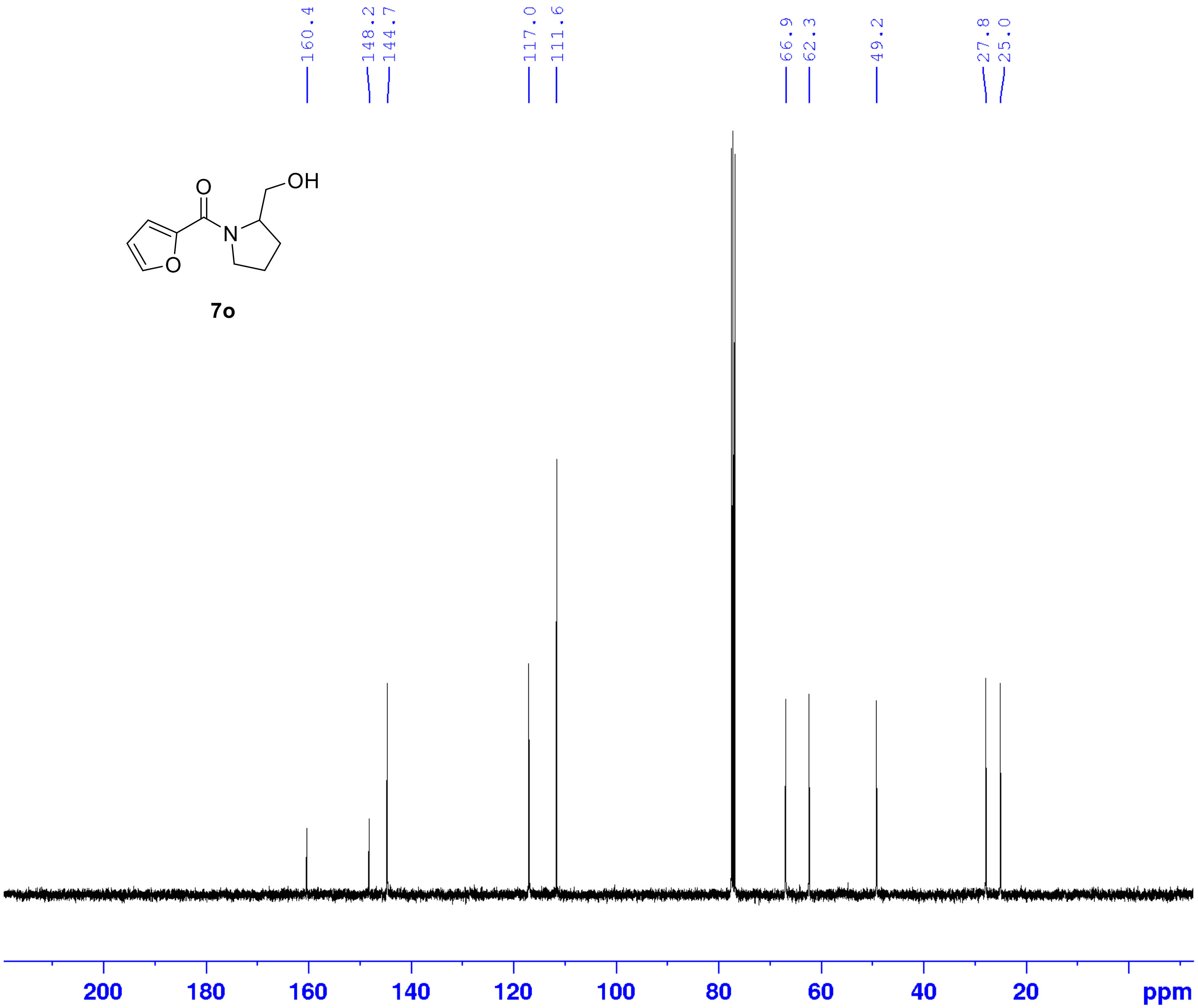

Current Data Parameters

NAME	KK-574
EXPNO	31
PROCNO	1

F2 - Acquisition Parameters

Date_	20230712
Time	13.23 h
INSTRUM	spect
PROBHD	Z116098_0048 (
PULPROG	zgpg30
TD	65536
SOLVENT	CDCl3
NS	256
DS	4
SWH	24038.461 Hz
FIDRES	0.733596 Hz
AQ	1.3631488 sec
RG	181.72
DW	20.800 usec
DE	8.54 usec
TE	298.1 K
D1	2.00000000 sec
D11	0.03000000 sec
TD0	8
SFO1	100.6228303 MHz
NUC1	13C
P0	3.00 usec
P1	9.00 usec
PLW1	77.00000000 W
SFO2	400.1316005 MHz
NUC2	1H
CPDPRG[2	waltz16
PCPD2	90.00 usec
PLW2	24.00000000 W
PLW12	0.25352001 W
PLW13	0.12751999 W

F2 - Processing parameters

SI	65536
SF	100.6127638 MHz
WDW	EM
SSB	0
LB	1.00 Hz
GB	0
PC	1.40

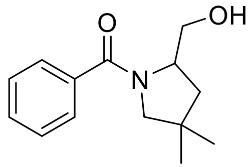

7p

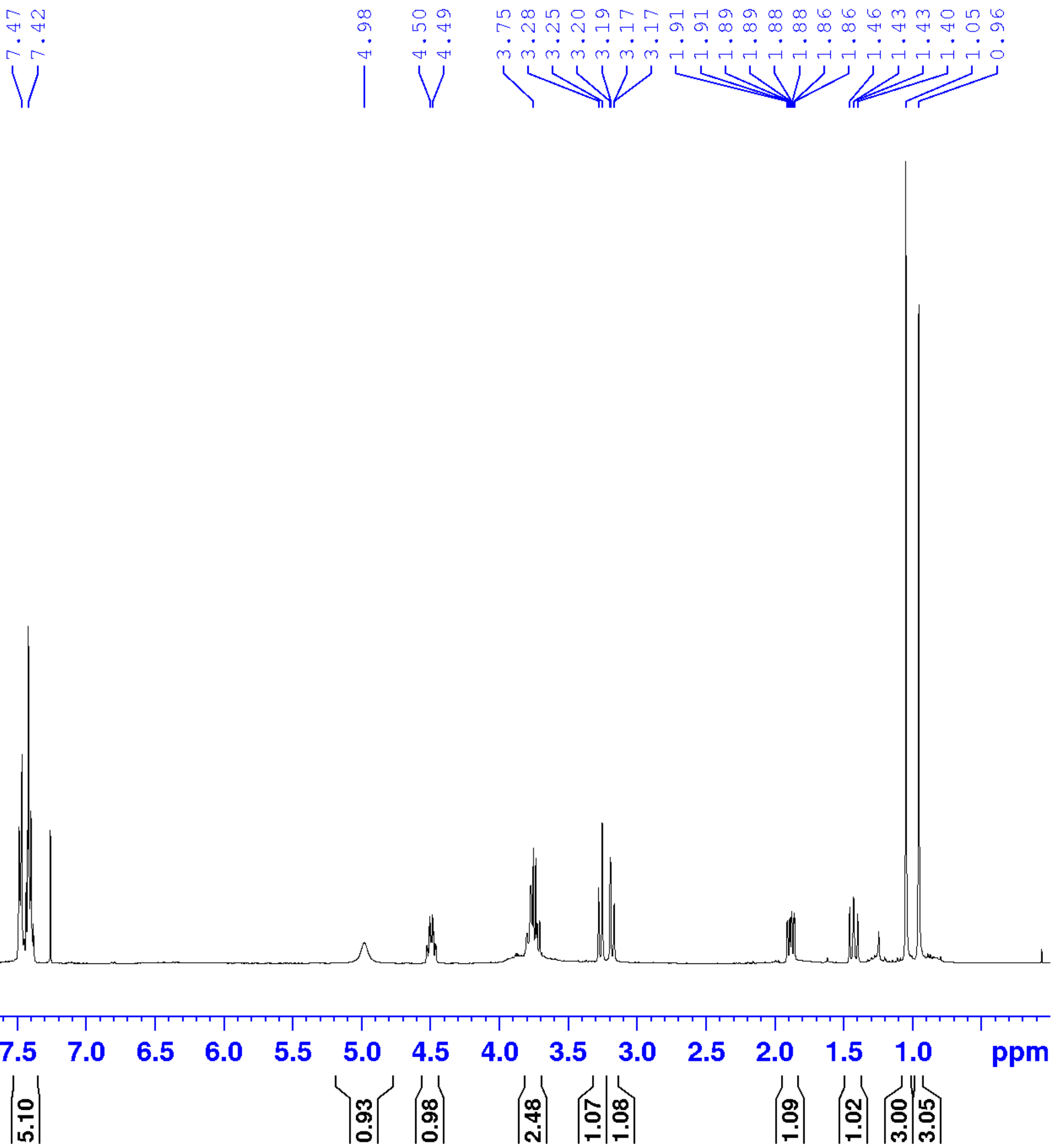


```
Current Data Parameters
NAME                KK-657
EXPNO                   41
PROCNO                   1

F2 - Acquisition Parameters
Date_             20240328
Time                 18.39 h
INSTRUM              spect
PROBHD     Z116098_0048 (
PULPROG               zg30
TD                   65536
SOLVENT              CDCl3
NS                      64
DS                       2
SWH               8223.685 Hz
FIDRES            0.250967 Hz
AQ               3.9845889 sec
RG                   65.91
DW                  60.800 usec
DE                   10.80 usec
TE                   298.1 K
D1              1.00000000 sec
TD0                      1
SFO1           400.1324710 MHz
NUC1                    1H
P0                    3.08 usec
P1                    9.25 usec
PLW1           24.00000000 W

F2 - Processing parameters
SI                   32768
SF             400.1300102 MHz
WDW                     EM
SSB                      0
LB                    0.30 Hz
GB                       0
PC                    1.50
```

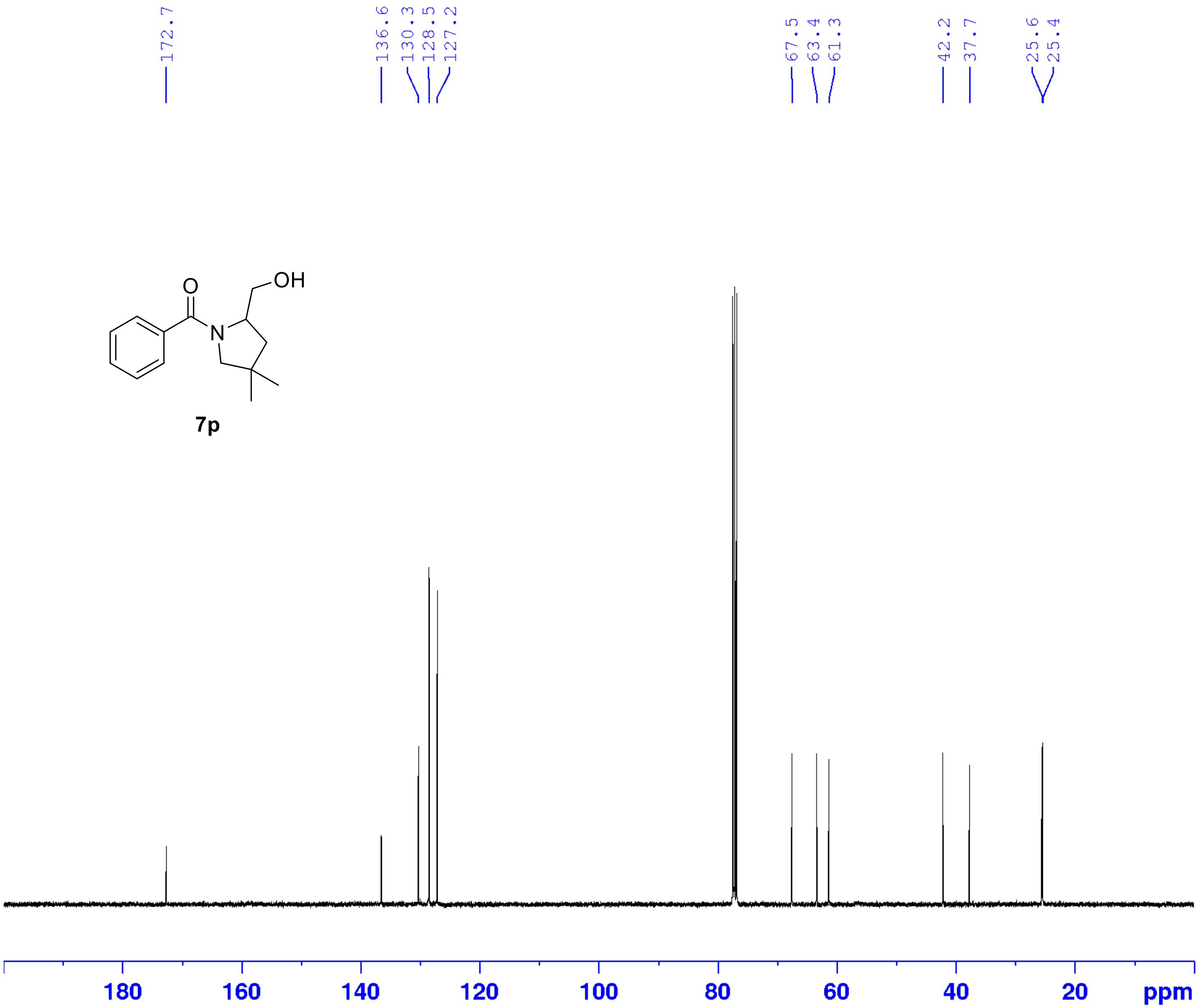


```
Current Data Parameters
NAME                KK-657
EXPNO                   43
PROCNO                   1

F2 - Acquisition Parameters
Date_             20240328
Time                 19.53 h
INSTRUM              spect
PROBHD      Z116098_0048 (
PULPROG            zgpg30
TD                   65536
SOLVENT              CDCl3
NS                    1024
DS                       4
SWH              24038.461 Hz
FIDRES            0.733596 Hz
AQ               1.3631488 sec
RG                  181.72
DW                  20.800 usec
DE                    8.54 usec
TE                   298.1 K
D1              2.00000000 sec
D11             0.03000000 sec
TD0                      8
SFO1           100.6228303 MHz
NUC1                   13C
P0                    3.00 usec
P1                    9.00 usec
PLW1           77.00000000 W
SFO2           400.1316005 MHz
NUC2                    1H
CPDPRG[2           waltz16
PCPD2                90.00 usec
PLW2           24.00000000 W
PLW12           0.25352001 W
PLW13           0.12751999 W

F2 - Processing parameters
SI                   65536
SF             100.6127582 MHz
WDW                     EM
SSB                      0
LB                    1.00 Hz
GB                       0
PC                    1.40
```

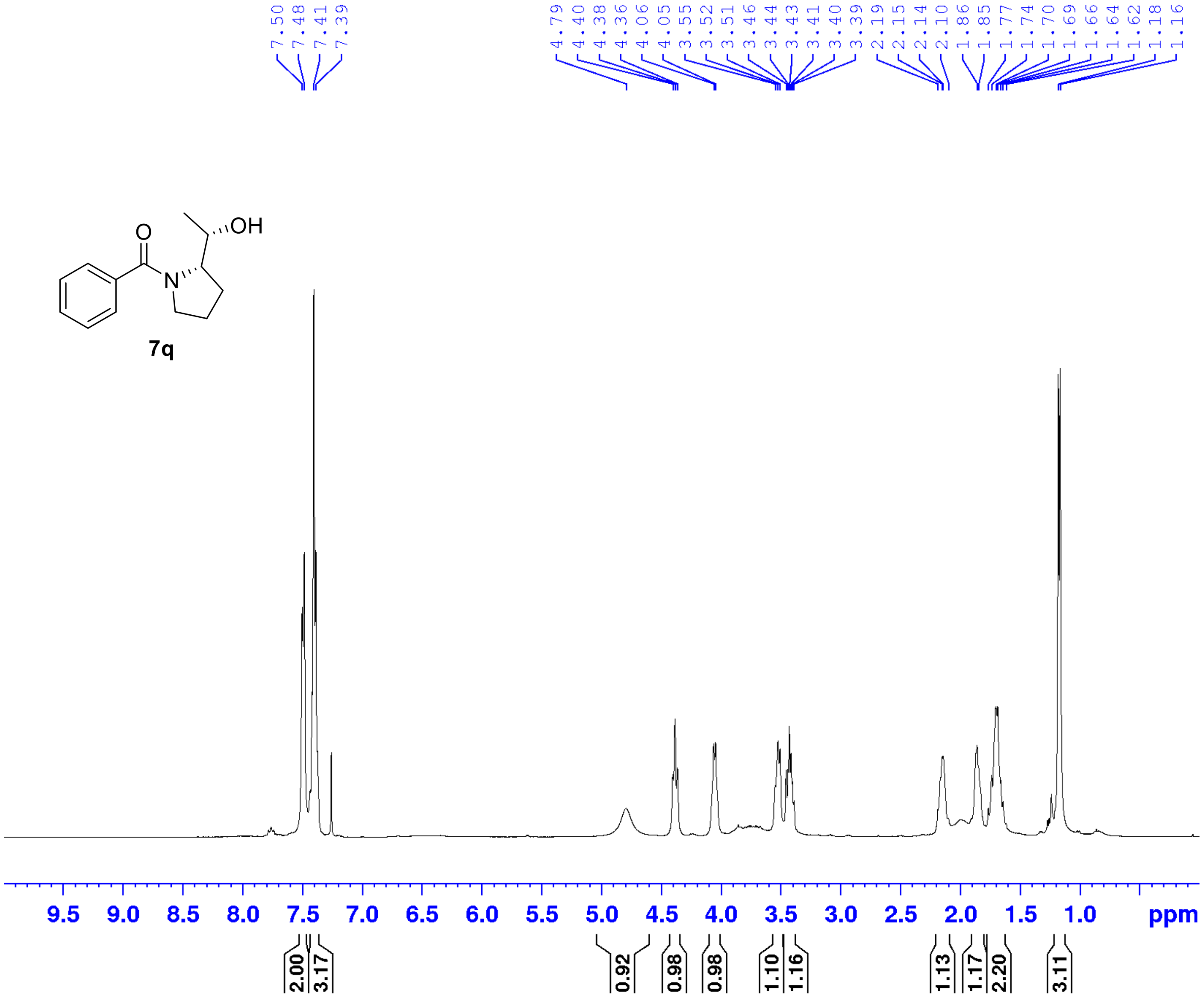

Current Data Parameters
NAME KK-596
EXPNO 20
PROCNO 1

F2 - Acquisition Parameters
Date_ 20230904
Time 12.59 h
INSTRUM spect
PROBHD Z116098_0048 (
PULPROG zg30
TD 65536
SOLVENT CDCl3
NS 16
DS 2
SWH 8223.685 Hz
FIDRES 0.250967 Hz
AQ 3.9845889 sec
RG 40.16
DW 60.800 usec
DE 10.80 usec
TE 298.1 K
D1 2.00000000 sec
TD0 1
SFO1 400.1324710 MHz
NUC1 1H
P0 3.08 usec
P1 9.25 usec
PLW1 24.00000000 W

F2 - Processing parameters
SI 32768
SF 400.1300108 MHz
WDW EM
SSB 0
LB 0.30 Hz
GB 0
PC 1.50

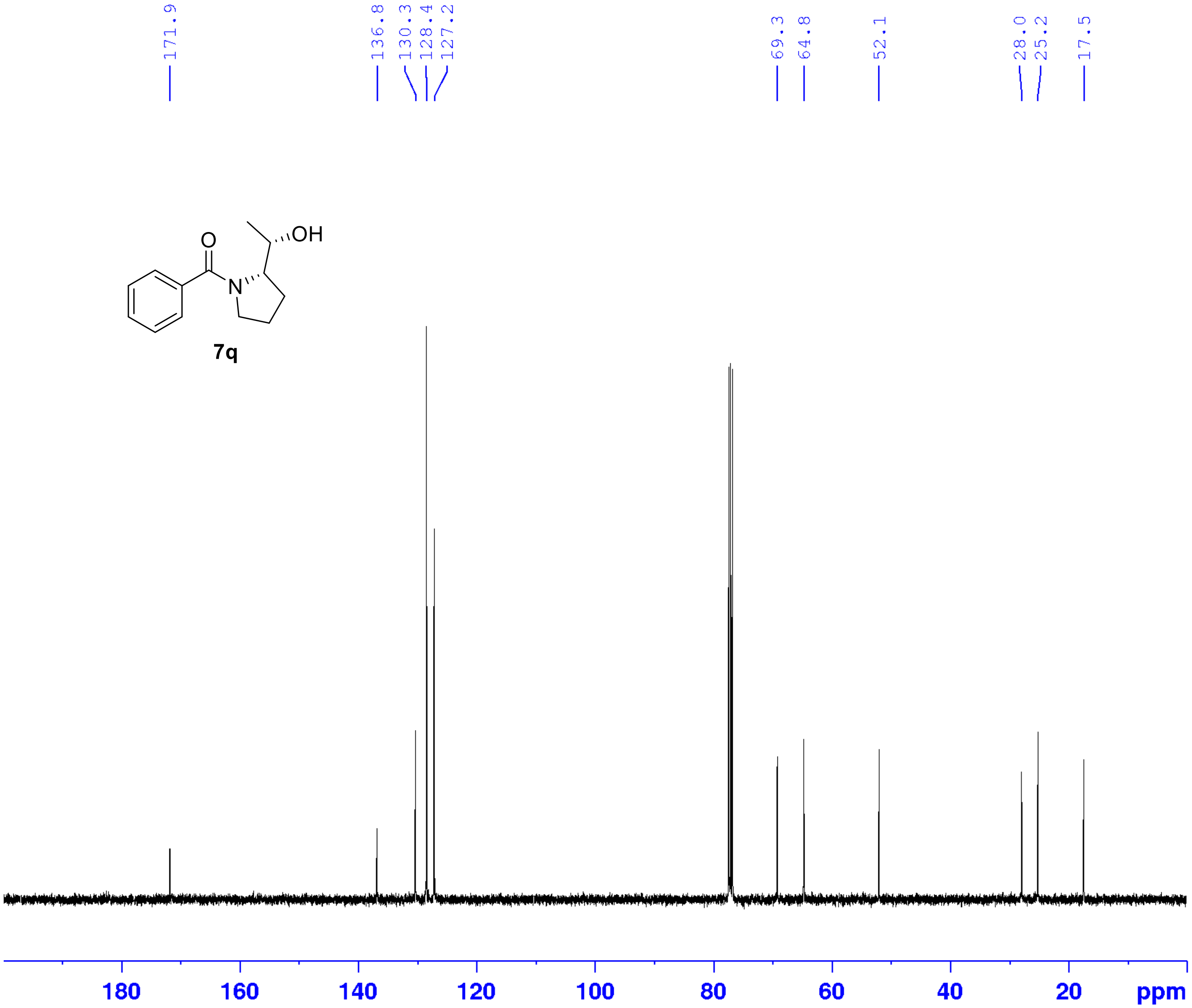

Current Data Parameters
NAME KK-596
EXPNO 21
PROCNO 1

F2 - Acquisition Parameters
Date_ 20230906
Time 20.01 h
INSTRUM spect
PROBHD Z116098_0048 (
PULPROG zgpg30
TD 65536
SOLVENT CDCl3
NS 256
DS 4
SWH 24038.461 Hz
FIDRES 0.733596 Hz
AQ 1.3631488 sec
RG 181.72
DW 20.800 usec
DE 8.54 usec
TE 298.1 K
D1 2.00000000 sec
D11 0.03000000 sec
TD0 8
SFO1 100.6228303 MHz
NUC1 13C
P0 3.00 usec
P1 9.00 usec
PLW1 77.00000000 W
SFO2 400.1316005 MHz
NUC2 1H
CPDPRG[2 waltz16
PCPD2 90.00 usec
PLW2 24.00000000 W
PLW12 0.25352001 W
PLW13 0.12751999 W

F2 - Processing parameters
SI 65536
SF 100.6127613 MHz
WDW EM
SSB 0
LB 1.00 Hz
GB 0
PC 1.40

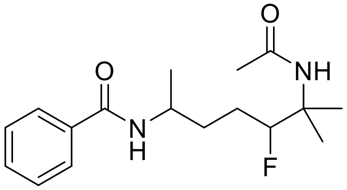

15

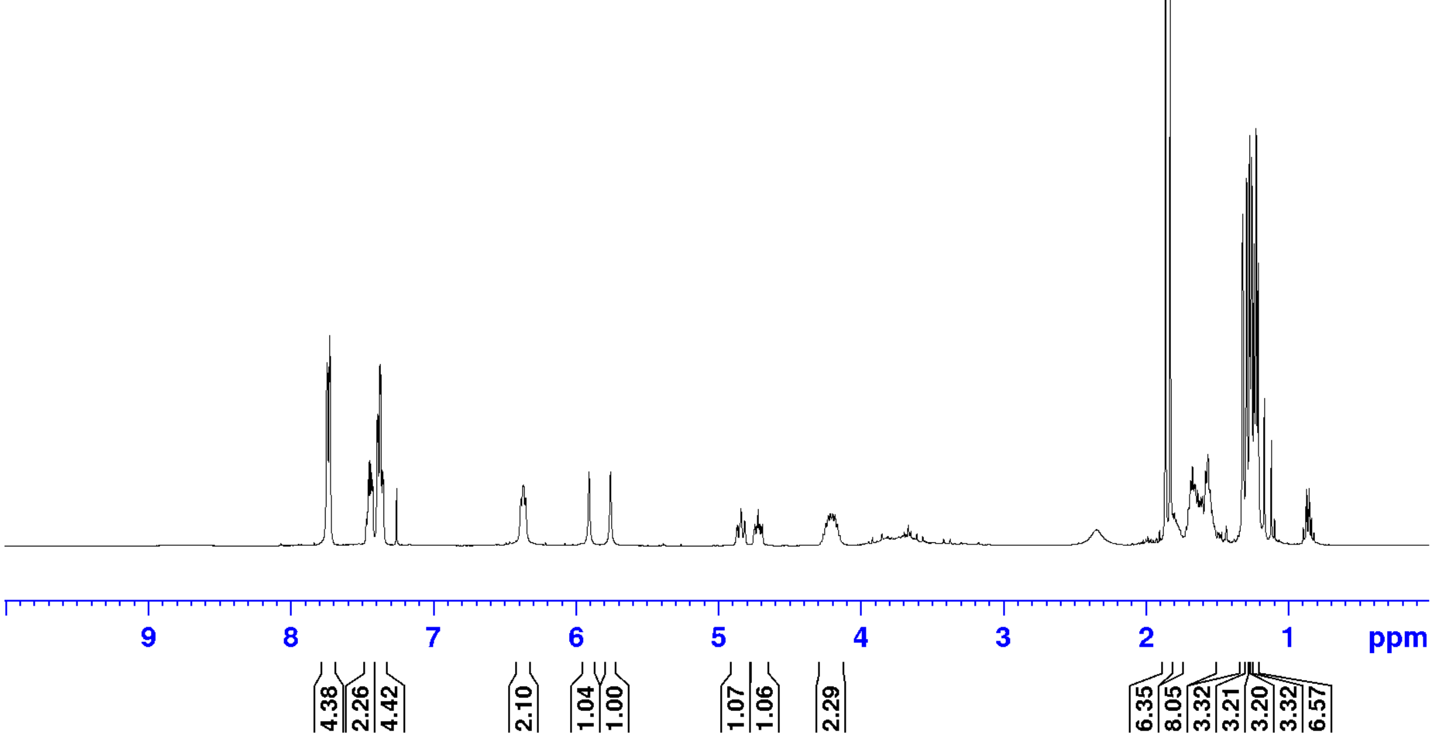


```
Current Data Parameters
NAME                KK-613
EXPNO                   10
PROCNO                   1

F2 - Acquisition Parameters
Date_             20230904
Time                 14.48 h
INSTRUM              spect
PROBHD    Z116098_0048 (
PULPROG               zg30
TD                   65536
SOLVENT              CDCl3
NS                      16
DS                       2
SWH               8223.685 Hz
FIDRES            0.250967 Hz
AQ               3.9845889 sec
RG                      25
DW                  60.800 usec
DE                   10.80 usec
TE                   298.1 K
D1              2.00000000 sec
TD0                      1
SFO1           400.1324710 MHz
NUC1                    1H
P0                    3.08 usec
P1                    9.25 usec
PLW1           24.00000000 W

F2 - Processing parameters
SI                   32768
SF             400.1300101 MHz
WDW                     EM
SSB                      0
LB                    0.30 Hz
GB                       0
PC                    1.50
```

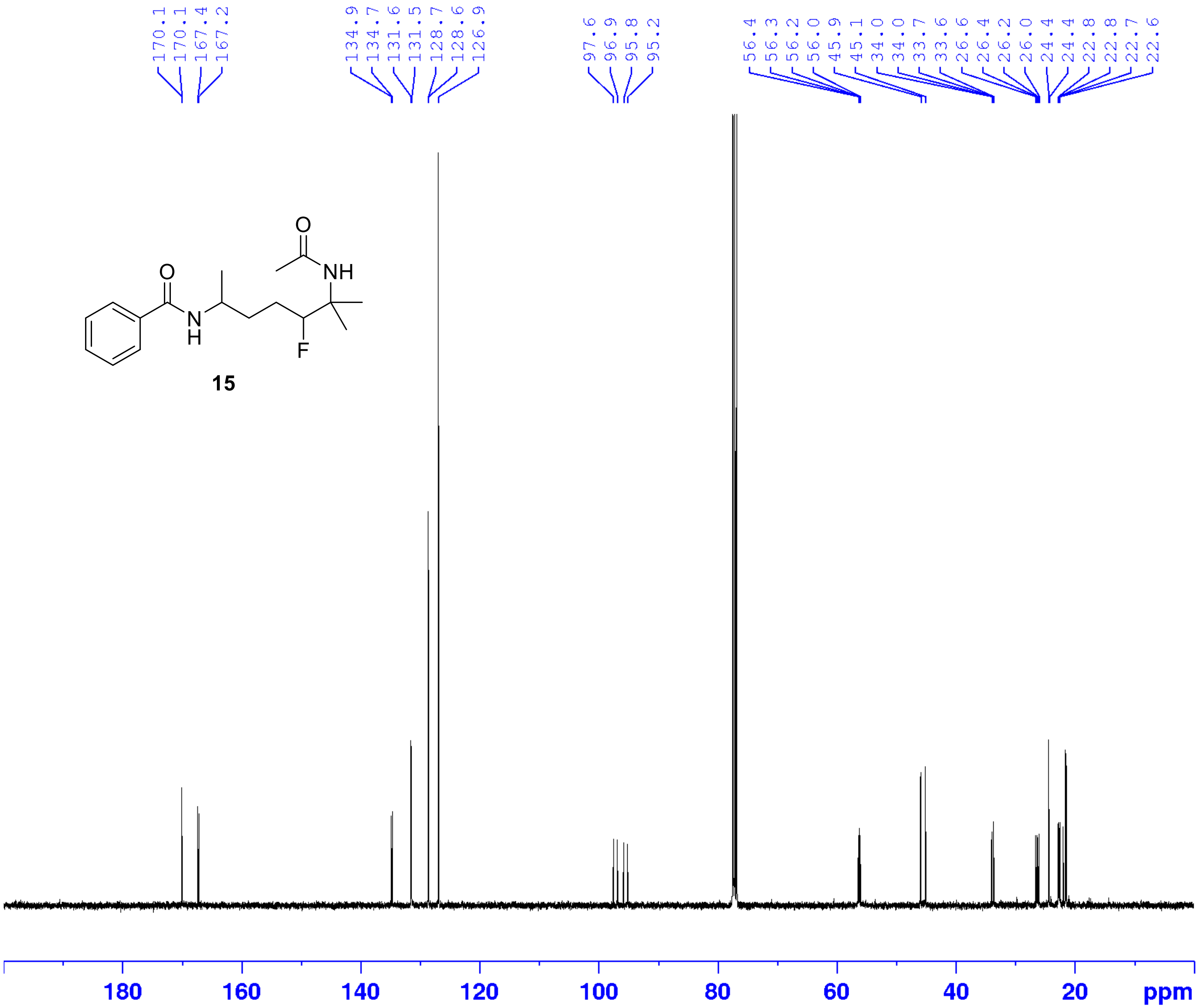

Current Data Parameters
NAME KK-613
EXPNO 23
PROCNO 1

F2 - Acquisition Parameters

Date_	20240322
Time	19.52 h
INSTRUM	spect
PROBHD	Z116098_0048 (
PULPROG	zgpg30
TD	65536
SOLVENT	CDCl3
NS	1024
DS	4
SWH	24038.461 Hz
FIDRES	0.733596 Hz
AQ	1.3631488 sec
RG	181.72
DW	20.800 usec
DE	8.54 usec
TE	298.2 K
D1	2.00000000 sec
D11	0.03000000 sec
TD0	8
SFO1	100.6228303 MHz
NUC1	13C
P0	3.00 usec
P1	9.00 usec
PLW1	77.00000000 W
SFO2	400.1316005 MHz
NUC2	1H
CPDPRG[2	waltz16
PCPD2	90.00 usec
PLW2	24.00000000 W
PLW12	0.25352001 W
PLW13	0.12751999 W

F2 - Processing parameters

SI	65536
SF	100.6127599 MHz
WDW	EM
SSB	0
LB	1.00 Hz
GB	0
PC	1.40

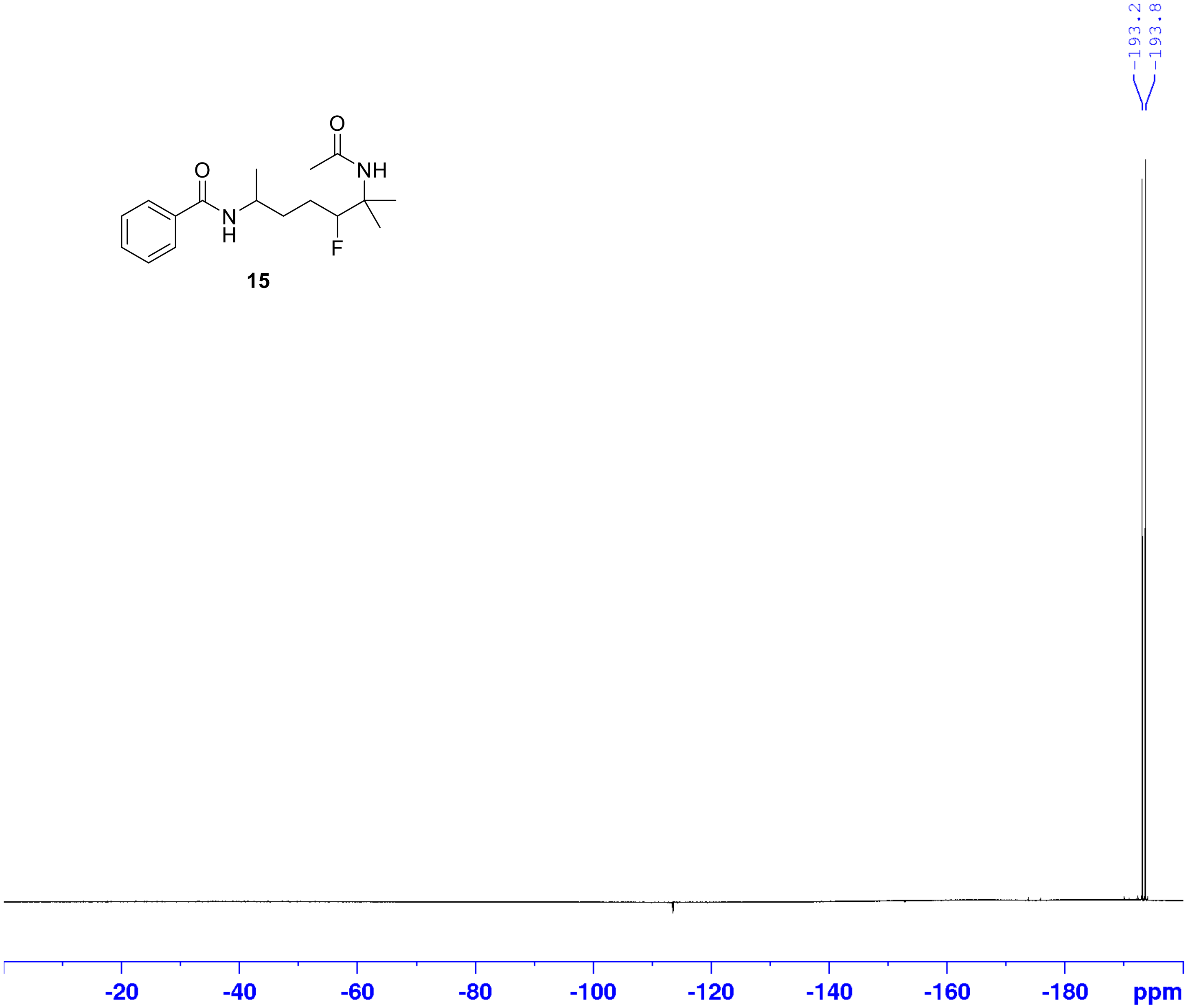


```
Current Data Parameters
NAME              KK-613
EXPNO                 31
PROCNO                 1

F2 - Acquisition Parameters
Date_           20240325
Time               22.48 h
INSTRUM            spect
PROBHD   Z116098_0048 (
PULPROG             zgig
TD                262144
SOLVENT            CDCl3
NS                    16
DS                     2
SWH            89285.711 Hz
FIDRES          0.681196 Hz
AQ             1.4680064 sec
RG                181.72
DW                 5.600 usec
DE                  7.11 usec
TE                 298.2 K
D1            2.00000000 sec
D11           0.03000000 sec
TD0                    1
SFO1         376.4607168 MHz
NUC1                 19F
P1                 14.00 usec
PLW1         20.00000000 W
SFO2         400.1316005 MHz
NUC2                  1H
CPDPRG[2         waltz16
PCPD2              90.00 usec
PLW2         24.00000000 W
PLW12         0.25352001 W

F2 - Processing parameters
SI                262144
SF           376.4983660 MHz
WDW                   EM
SSB                    0
LB                  0.30 Hz
GB                     0
PC                  1.00
```

[1] Gaussian 16, Revision C.01, Frisch, M. J.; Trucks, G. W.; Schlegel, H. B.; Scuseria, G. E.; Robb, M. A.; Cheeseman, J. R.; Scalmani, G.; Barone, V.; Petersson, G. A.; Nakatsuji, H.; Li, X.; Caricato, M.; Marenich, A. V.; Bloino, J.; Janesko, B. G.; Gomperts, R.; Mennucci, B.; Hratchian, H. P.; Ortiz, J. V.; Izmaylov, A. F.; Sonnenberg, J. L.; Williams-Young, D.; Ding, F.; Lipparini, F.; Egidi, F.; Goings, J.; Peng, B.; Petrone, A.; Henderson, T.; Ranasinghe, D.; Zakrzewski, V. G.; Gao, J.; Rega, N.; Zheng, G.; Liang, W.; Hada, M.; Ehara, M.; Toyota, K.; Fukuda, R.; Hasegawa, J.; Ishida, M.; Nakajima, T.; Honda, Y.; Kitao, O.; Nakai, H.; Vreven, T.; Throssell, K.; Montgomery, J. A., Jr.; Peralta, J. E.; Ogliaro, F.; Bearpark, M. J.; Heyd, J. J.; Brothers, E. N.; Kudin, K. N.; Staroverov, V. N.; Keith, T. A.; Kobayashi, R.; Normand, J.; Raghavachari, K.; Rendell, A. P.; Burant, J. C.; Iyengar, S. S.; Tomasi, J.; Cossi, M.; Millam, J. M.; Klene, M.; Adamo, C.; Cammi, R.; Ochterski, J. W.; Martin, R. L.; Morokuma, K.; Farkas, O.; Foresman, J. B.; Fox, D. J. 2016.
[2] GaussView, Version 5, Dennington, Roy; Keith, Todd A.; Millam, John M. Semichem Inc., Shawnee Mission, KS, 2016.
[3] (a) Becke, A. D. *J. Chem. Phys.* **1993**, *98*, 1372-1377. (b) Becke, A. D. *Phys. Rev. A*. **1988**, *38*, 3098. (c) Lee, C.; Yang, W.; Parr, R. G. *Phys. Rev. B: Condens. Matter* **1988**, *37*, 785.
[4] Hariharan, P. C.; Pople, J. A. *Theoret. Chim. Acta*. **1973**, *28*, 213-222.
[5] Igel-Mann, G.; Stoll, H., Preuss, H. *Mol. Phys*. **1988**, *65*, 1321-1328.
[6] (a) Gonzalez, C.; Schlegel, H. B. J. *Chem. Phys*. **1989**, *90*, 2154-2161. (b) Gonzalez, C.; Schlegel, H. B. J. *Phys. Chem*. **1990**, *94*, 5523-5527.
[7] Cossi, M.; Rega, N.; Scalmani, G.; Barone, V. *J. Comput. Chem*. **2003**, *24*, 669-681.
[8] E. M. Stang and M. C. White *J. Am. Chem. Soc*. **2011**, *133*, 14892–14895.
[9] D.-W. Gao, E. V. Vinogradova, S. K. Nimmagadda, J. M. Medina, Y. Xiao, R. M. Suciu, B. F. Cravatt, and K. M. Engle *J. Am. Chem. Soc.* **2018**, *140*, 8069–8073.
[10] Q. Hu, W-L. Yu, Y-C. Luo, X-Q. Hu, P-F. Xu *J. Org. Chem*., **2022**, *87*, 1493-1501.
[11] M. B. Bertrand and J. P. Wolfe *Tetrahedron* **2005**, *61*, 6447–6459.
[12] Du, W.; Gu, Q.; Li, Y.; Lin, Z.; Yang, D. *Org. Lett.* **2017**, *19*, 316-319.
[13] G. Zhang, L. Cui, Y. Wang, L. Zhang, *J. Am. Chem. Soc*. **2010**, *132*, 1474-1475.
[14] P. A. Sibbald and F. E. Michael *Org. Lett.* **2009**, *11*, 1147-1149.
[15] Y. Nishii, T. Hirai, S. Fernandez, P. Knochel, K. Mashima *Eur. J. Org. Chem.* **2017**, *34*, 5010-5014.
[16] Mao, K.; Lv, L.; Li, Z. *J. Org. Chem.* **2023**, *88*, 10137–10146.
[17] M. Besson, P. Gallezot, S. Neto and C. Pinel *Tetrahedron: Asymm.* **2000**, *11*, 1809-1818.
